# Ultrafast synthesis of zirconium-porphyrin framework nanocrystals from alkoxide precursors

**DOI:** 10.1016/j.xcrp.2024.102318

**Published:** 2024-12-18

**Authors:** Manuel Ceballos, Giulia Zampini, Oleg Semyonov, Samuel Funes-Hernando, José Manuel Vila-Fungueiriño, Sonia Martínez-Giménez, Sergio Tatay, Carlos Martí-Gastaldo, Thomas Devic, Beatriz Pelaz, Pablo del Pino

**Affiliations:** 1 Centro Singular de Investigación en Química Biolóxica e Materiais Moleculares (CiQUS), Departamento de Física de Partículas, Universidade de Santiago de Compostela, 15782 Santiago de Compostela, Spain; 2 Centro Singular de Investigación en Química Biolóxica e Materiais Moleculares (CiQUS), Universidade de Santiago de Compostela, 15782 Santiago de Compostela, Spain; 3 Centro Singular de Investigación en Química Biolóxica e Materiais Moleculares (CiQUS), Departamento de Química Física, Universidade de Santiago de Compostela, 15782 Santiago de Compostela, Spain; 4 Instituto de Ciencia Molecular (ICMol), Universitat de València, Catedrático José Beltrán-2, 46980 Paterna, Spain; 5 Nantes Université, CNRS, Institut des Matériaux de Nantes Jean Rouxel, IMN, 44000 Nantes, France; 6 Centro Singular de Investigación en Química Biolóxica e Materiais Moleculares (CiQUS), Departamento de Química Inorgánica, Universidade de Santiago de Compostela, 15782 Santiago de Compostela, Spain

**Keywords:** porphyrinic MOFs, Zr alkoxides, nanosized MOFs, nanoMOFs, in-flow synthesis, PCN-224, PCN-222, MOF-525, disordered PCN-224, dPCN-224, Zr_6_-oxo cluster

## Abstract

Porphyrinic metal-organic frameworks (MOFs) offer high surface areas and tunable catalytic and optoelectronic properties, making them versatile candidates for applications in phototherapy, drug delivery, photocatalysis, electronics, and energy storage. However, a key challenge for industrial integration is the rapid, cost-effective production of suitable sizes. This study introduces Zr(IV) alkoxides as metal precursors, achieving ultrafast (∼minutes) and high-yield (>90%) synthesis of three well-known Zr-based porphyrinic MOF nanocrystals: MOF-525, PCN-224, and PCN-222, each with distinct topologies. By adjusting linker-to-metal and modulator-to-metal ratios, we attain precise control over single-phase formation. Demonstrating alkoxides’ potential, we synthesized nanosized PCN-224 at room temperature within seconds using a continuous multifluidic method. This advancement greatly simplifies porphyrinic MOF production, enabling broader industrial and scientific applications.

## Highlights

• Zr(IV) alkoxides enable the fast, efficient synthesis of porphyrinic MOF nanocrystals
• MOF-525, PCN-224, and PCN-222 are synthesized in minutes with high yields and purity
• Tuning linker and modulator ratios allows precise control of crystal phases
• Room temperature continuous-flow synthesis supports scalable MOF production

## Graphical abstract

**Figure:**
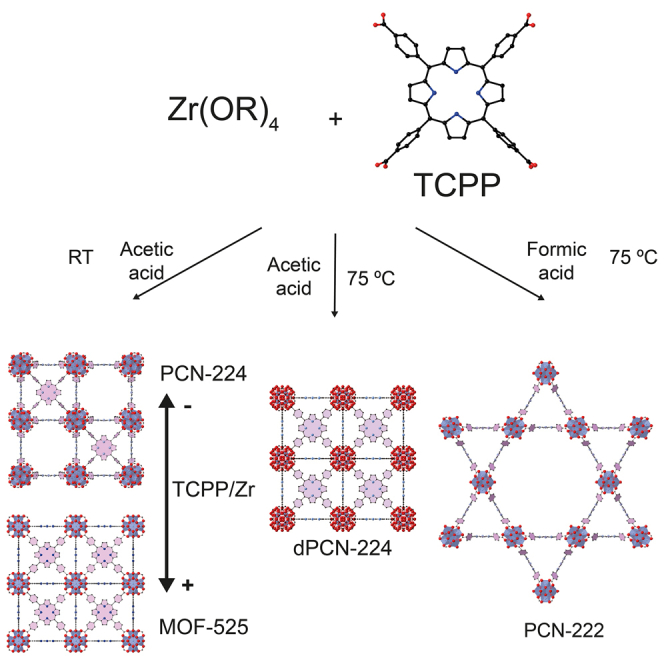

## Teaser

Ceballos et al. present a rapid and efficient method using Zr(IV) alkoxides to synthesize porphyrinic MOF nanocrystals, including MOF-525, PCN-224, and PCN-222, under mild conditions. This approach enables precise control over crystal phases and continuous synthesis at room temperature, supporting scalable MOF production with potential applications in technology and medicine.

## Introduction

Zirconium-based metal-organic frameworks (MOFs) are among the most promising microporous materials for a wide range of applications, from energy production and environmental remediation to biomedical uses in creating theragnostic nanocarriers.^1^^,^^2^^,^^3^ Most Zr-carboxylate MOFs rely on the robust secondary building unit (SBU) formed by the Zr_6_-oxo cluster, which can connect up to 12 carboxylate ligands, resulting generally in highly stable nets,^4^ where the SBU can be tuned to create missing linker defects or missing cluster defects, which directly impact the physicochemical properties.^5^ Over 7,600 Zr-based networks have been reported in the literature.^6^ One of the most prolific building blocks in the literature, tetrakis(4-carboxyphenyl)porphyrin (TCPP), serves as a tetratopic carboxylate ligand that, when combined with the Zr_6_-oxo cluster, has led to the renowned MOF networks MOF-525,^7^ PCN-222 (also known as MOF-545 or MMPF-6),^7^^,^^8^^,^^9^ and PCN-224,^10^ with connectivities of 12, 8, and 6, respectively. Other notable examples include the 8-connected (8-c) polymorphs PCN-225 and NU-902,^11^^,^^12^ as well as the 12-c PCN-223.^13^ The recent development of dPCN-224, a disordered variant of PCN-224, which incorporates the Zr_6_-oxo cluster in four spatial orientations,^14^ has elucidated the previously puzzling short and unrealistic Zr-Zr distance of 2.69 Å found in the Zr_8_O_6_ cluster in PCN-221.^15^ These porphyrinic frameworks exhibit exceptionally high surface areas (∼2,500 m^2^/g) and are remarkably versatile, presenting significant potential as nanozymes for myriad applications,^16^ including photocatalysis,^17^ energy production,^18^ photodynamic therapy,^19^ and drug delivery.^20^

A recent interlaboratory study, led by Lotsch’s group, highlighted challenges in achieving reproducible syntheses of pure PCN-222 and PCN-224.^21^ This study identified that the synthesis process is sensitive to factors that are not yet fully understood, with the major challenge being the identification and control of these variables. Interestingly, another recent study by the same group suggested that the amount of water used during synthesis directly affects the speciation and phase formation of Zr-porphyrinic MOFs.^22^

Although metal chlorides/oxochlorides have been the preferred metal sources for many years, the mechanism underlying the formation of the Zr_6_-oxo cluster remains not yet fully elucidated. Some reports indicate that dimethylformamide (DMF) and water are essential for forming an intermediate [ZrCl(OH)_2_(DMF)_2_]Cl. This intermediate then reacts with a carbonylated ligand to form the corresponding MOF.^23^ In more recent research, high-resolution mass spectrometry revealed that DMF undergoes partial hydrolysis, producing dimethylammonium species that subsequently exchange with zirconium atoms.^24^ Additionally, in this work, chloride ions from ZrCl_4_ were found to play a crucial role in the formation of zirconium chloroterephthalates, as demonstrated in the synthesis of UiO-66. This finding has sparked controversy, particularly regarding the use of other zirconium precursors, such as chloride-free alkoxides, which may impact the reaction pathway and the structure of the resulting MOFs.

While metal alkoxides have been extensively utilized in sol-gel synthesis due to their rapid hydrolysis, enabling the synthesis of catalytically active metal oxides such as Al_2_O_3_, Y_2_O_3_, ZrO_2_, or TiO_2_,^25^ their use as metal sources in Zr-MOF synthesis is more limited. They have been employed in the early synthesis of molecular oxo clusters like [Zr_6_(μ_3_-O)_4_(μ_3_-OH)_4_]^12+^ stabilized with various carboxylic acids.^26^^,^^27^^,^^28^ These clusters were next used as precursors to produce Zr-based MOFs that, because of cluster facile hydrolysis, could be prepared in mild conditions^29^ and with short reaction times while avoiding the formation of ordered defects and impurities^30^ and preventing the formation of polymorphs.^31^ The benefits of using alkoxides as metal sources also include high reaction yields, and the production of alcohols as byproducts rather than hydrochloric acid or chlorides may impact the reaction pathway and structure of the resulting MOFs.^24^ Moreover, their use helps to control the amount of water used during synthesis that has been shown to directly affect the speciation and phase formation of Zr-porphyrinic MOFs.^22^ Nonetheless, Zr alkoxides have scarcely been used for the direct synthesis of Zr-based MOFs.^32^^,^^33^^,^^34^^,^^35^ On top of that, alkoxide rapid hydrolysis makes them well suited for the synthesis of MOF nanocrystals (nanoMOFs) because it favors seeding during the crystallization process. These nanoMOFs often possess a larger outer surface area, which imparts distinct properties such as higher surface energy and a greater number of defects. These properties can significantly affect the flexibility, processability, and catalytic performance of the MOFs.^36^

In this work, we demonstrate that Zr alkoxide precursors facilitate the rapid and straightforward synthesis of nanosized MOF-525, PCN-224, and PCN-222 under mild synthetic conditions, crucially avoiding the mixing of crystalline phases. Most importantly, we show that our synthetic protocol can be adapted to the production of PCN-224 in continuous microflow reaction conditions.

**Scheme 1:**
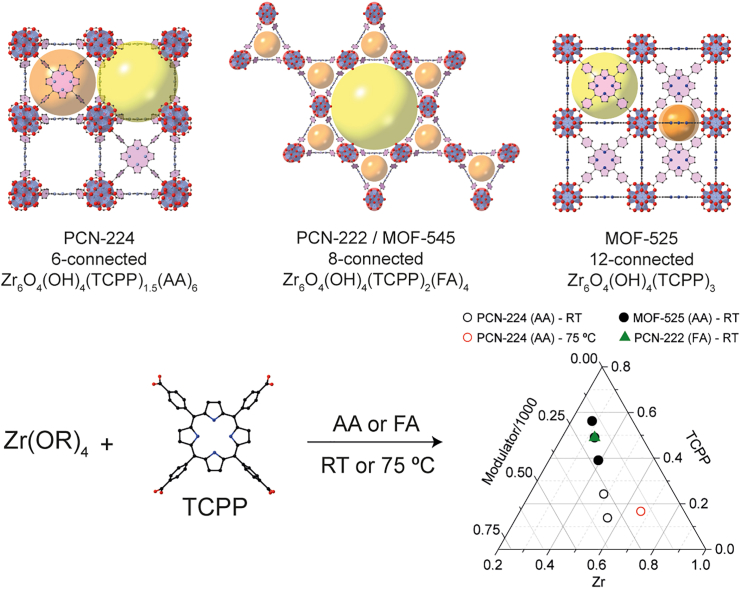
Variations in connectivity and organic linker arrangements in porphyrinic MOFs Top row: comparative structures of three different MOFs: PCN-224, PCN-222/MOF-545, and MOF-525, showing varying connectivity and organic linker arrangements. Bottom row: synthesis approach utilizing Zr-alkoxide precursors and TCPP. By adjusting the linker-to-metal (L/M) and modulator-to-metal (Mod/M) ratios, as well as the choice of modulator, we facilitate the formation of single phases of the depicted MOFs. These phases are further delineated in the tertiary diagram (bottom right).

As depicted in Scheme 1, the targeted synthesis of MOF-525, PCN-224, or PCN-222 can be accomplished by Zr alkoxide precursors by adjusting the linker-to-metal (L/M) and modulator-to-metal (Mod/M) ratios and changing the modulator from acetic acid (AA) to formic acid (FA) (Tables S1–S3). The ternary diagram shown in Scheme 1 provides a comprehensive overview of the synthetic conditions used to achieve pure phases of MOF-525, PCN-224, and PCN-222.

## Results and discussion

### Influence of linker-to-metal ratio at RT on MOF-525 and PCN-224 synthesis

This initial section explores the influence of the L/M ratio on the crystal phase of the attempted solids while keeping other potential reaction variables constant. We maintained a constant reaction time of 1 h at room temperature (RT) and used Zr(OEt)_4_ as the metal precursor and AA as the modulator. The Mod/M ratio was set to 560, inspired by previous work.^31^ To synthetize the porphyrinic framework with the lowest coordination connectivity, specifically the 6-c PCN-224, we initially set the L/M ratio to 0.25. This value aligns with the ideal formula of this MOF, Zr_6_O_4_(OH)_4_(TCPP)_1.5_(AA)_6_, with the anticipation that higher values would favor the formation of phases of higher connectivity, such as the 8-c PCN-222 or the 12-c MOF-525. We then gradually increased the L/M ratio up to 2.00, four times the amount necessary for maximal connectivity within the Zr_6_-oxo cluster of the canonical 12-c MOF-525. A comprehensive physicochemical characterization was conducted on the five purified solids, which were examined both as dried solids, to assess the coordination degree of the Zr_6_-oxo cluster and the potential presence of defects, and as colloidal dispersions.

**Figure 1:**
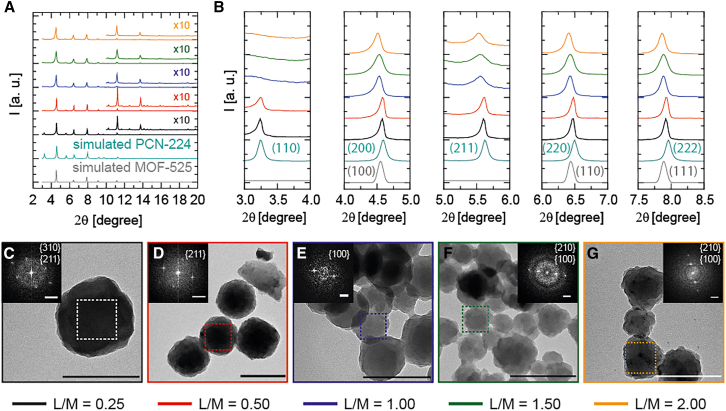
Structural characterization of nanoMOFs obtained by varying the L/M ratio at room temperature (A and B) PXRD diffractogram with the simulated patterns of PCN-224 and MOF-525 (A) and normalized first 5 diffraction peaks (B). (C–G) HR-TEM images of porphyrinic MOF nanoparticles changing the linker-to-metal (L/M) ratios (C) 0.25, (D) 0.50, (E) 1.00, (F) 1.50, and (G) 2.00 and their corresponding FFT, indexing some different crystallographic planes (PCN-224 for L/M ratios 0.25 and 0.50 and MOF-525 for L/M ratios 1.00, 1.50, and 2.00). Scale bars represent 200 nm for HR-TEM images and 0.5 nm^−1^ for FFT images.

**Figure 2:**
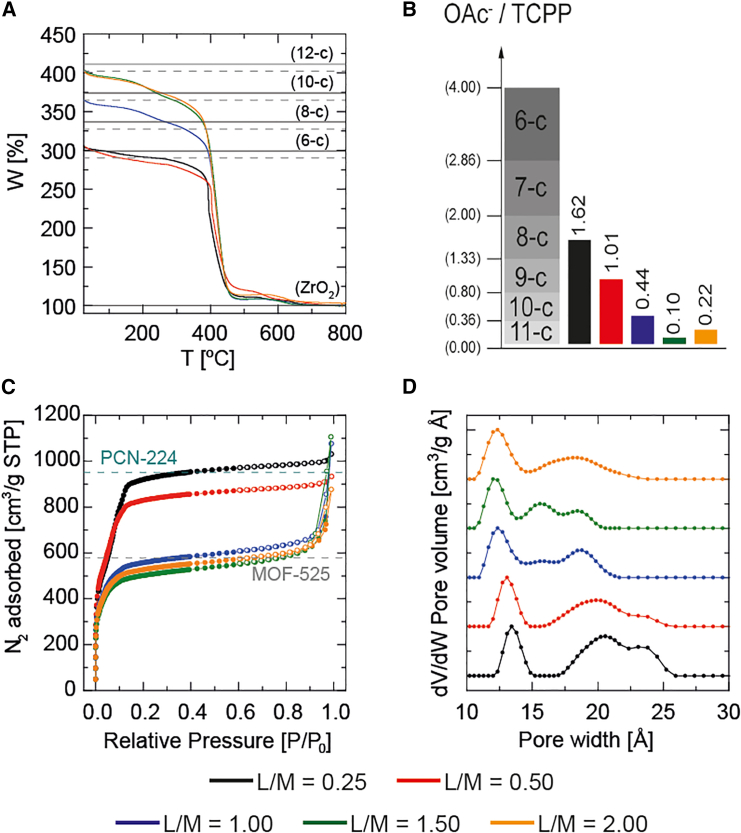
Characterization of nanoMOFs obtained by varying the L/M ratio at room temperature (A) Normalized TGA with the theoretical mass molar of Zr_6_-oxo cluster with different coordination degrees with TCPP and acetate molecules (dashed lines are the dehydrated cluster). (B) Quantification of TCPP molecules by ^1^H-NMR. (C) N_2_ adsorption isotherms at 77 K correspond to the expected values for ideal PCN-224 and MOF-525 phases calculated with Zeo++.^37^ (D) Pore size distribution using NLDFT model.

We confirmed the crystalline nature of the purified solids through powder X-ray diffraction (PXRD) analysis. Figures 1A and 1B display the PXRD diffractogram and its detailed magnification for the five solids studied, along with the simulated patterns for PCN-224 and MOF-525. Figure 1B presents close-up views of the initial five diffraction reflections. Notably, the absence of the (110) diffraction reflection and the broadening of the (211) diffraction reflection, with very low intensity visible only after normalization (Figure 2A), at two-theta angles of 3.2° and 5.6°, characteristic of PCN-224, are evident for L/M ratios of 1.00, 1.50, and 2.00, indicating the presence of MOF-525. To corroborate the absence of this reflection (110) for the higher L/M ratios, small-angle diffraction was performed (Figure S1). Overall, summarizing the PXRD data in Figures 1A and 1B, the L/M ratios in the stoichiometric condition for PCN-224 (L/M = 0.25) and the double (L/M = 0.50) suggest the formation of PCN-224 solids, while overpassing this L/M ratio, i.e., 1.00, 1.50, and 2.00, suggests the formation of MOF-525, compatible with Pawley refinement (Figure S2).

Figures 1C–1G display transmission electron microscopy (TEM) images of the five solids at L/M ratios ranging from 0.25 to 2.00. Average particle sizes for these ratios were measured as 527.7 ± 251.7, 193.0 ± 61.4, 105.0 ± 22.9, 97.1 ± 17.5, and 102.8 ± 16.3 nm, respectively (Figures S3 and S4) A trend is observed where an increase in L/M ratio leads to a decrease in both particle size and polydispersity, as well as a decrease in crystallite size calculated using the Scherrer equation (Equation S1; Table S4). Furthermore, high-resolution TEM (HR-TEM) images (Figures 1C–1G) confirm the stability of MOF structures under an 80 kV electron beam, with fast Fourier transform (FFT) analyses on designated regions of interest (dashed ROIs) providing critical d-spacing measurements that illustrate a transition to the MOF-525 phase at higher L/M ratios in agreement with the PXRD analysis results depicted in Figures 1A and 1B.

The thermogravimetric analysis (TGA) of the dried crystalline solids clearly demonstrated the high thermal stability of the samples, enduring temperatures up to 400°C (Figure 2A), as expected for such Zr frameworks (Figure S5).^13^ For lower L/M ratios (0.25 and 0.50), the analysis revealed an inorganic residue constituting ∼33% of the overall weight, attributable to residual ZrO_2_, which strongly agrees with the theoretical residue of the 6-c Zr_6_O_4_(OH)_4_(TCPP)_1.5_(OAc)_6_ of 33.4%, following thermal decomposition. For intermediate (1.00) and higher (1.50 and 2.00) L/M ratios, inorganic residues of around 27% and 25% were obtained, respectively. The latter being in full agreement with the theoretical value of the 12-c MOF-525 (Zr_6_O_4_(OH)_4_(TCPP)_3_), i.e., 24.3%.

The normalized TGA results illustrate that the two solids with the lower L/M ratio reflect the stoichiometric ratio expected for 6-c PCN-224, with the theoretical formula Zr_6_O_4_(OH)_4_(TCPP)_1.5_(OAc)_6_ indicating full coordination with modulator AA. At a moderate L/M ratio (1.00), the degree of coordination involved fewer than 10 TCPP molecules. This finding suggests a slightly defective variant of the canonical 12-c MOF-525, characterized by a few missing linker defects (12.8%). Finally, for the two higher L/M ratios (1.50 and 2.00), the 12-c expected for MOF-525 is attained. As far as we are aware, there appears to be limited literature providing TGA evidence for a nearly saturated 12-c porphyrinic MOF.^38^

To further validate the TCPP coordination extent of the Zr porphyrinic MOFs, quantification was carried out using ^1^H-nuclear magnetic resonance (^1^H-NMR). This process involved dissolving the solids in a deuterated aqueous solution of NaHCO_3_^39^ and incorporating an internal standard (methylsulfonylmethane)^40^ to establish a correlation between the proton signal areas and the quantities of TCPP molecules and acetate ions (OAc). This correlation facilitated the determination of the OAc/TCPP ratio (Figure 2B).

In cases of lower L/M ratios with a coordination index of 6, the OAc/TCPP ratios were lower than 4.00 (1.62 and 1.01 for L/M ratios 0.25 and 0.50, respectively). This is compatible with an excess of TCPP molecules, a coordination degree slightly over 8, or the loss of OAc ions in the cluster. At an L/M ratio of 1, the ^1^H-NMR quantification yielded a ratio of 0.44, closely approximating a coordination of 10. This implies the presence of 10 TCPP molecules for every 2 OAc molecules within a cluster. Furthermore, the two higher L/M ratios (1.50 and 2.00) yielded ratios of 0.10 and 0.22, respectively, being lower than the ratio of 0.36 within the range of 11–12 TCPP molecules per cluster. These results imply that the L/M ratio of 1.50 accommodates more than 11 TCPP molecules per cluster. Similarly, the L/M ratio of 2.00 exhibits an alignment with nearly 12 TCPP molecules per Zr_6_-oxo cluster. NMR spectra and quantification data are available in the supplemental information (Figure S6; Table S5).

N_2_ adsorption isotherms, measured at 77 K as shown in Figure 2C and detailed in Table S6, reveal the expected differences in N_2_ uptake among samples with varying L/M ratios, impacting directly in Brunauer-Emmett-Teller (BET) surface areas, which were calculated with BETSI analysis.^41^ Lower L/M ratios (0.50 and 0.25) result in adsorption isotherms and pore width distributions that align with those expected for PCN-224,^10^ while higher L/M ratios (1.00, 1.50, and 2.00) lead to a decreased N_2_ uptake, a reduction in BET surface areas, and a narrowing of pore size distribution that align with what is expected for MOF-525,^42^ as depicted in Figure 2D and elaborated on in Table S7 and Figure S7. Higher ratios feature a primary pore size centered around 12 Å (Figure S7). The N_2_ adsorption data thus support and complement the structural characterization previously discussed, underlining the impact of L/M ratio variations on the physicochemical properties of the MOFs.

The UV-visible (UV-vis) spectra of colloidal dispersions of the purified solids in MeOH reveal that at an L/M ratio of 0.25 (black line), the porphyrinic framework characteristic Soret band undergoes a red shift and notable broadening, with significant scattering observed in the four Q bands beyond 515 nm due to large particle sizes (Figure S8A). Increasing the L/M ratio to 0.50 (red line) narrows the Soret band and decreases Q-band scattering. Higher L/M ratios (1.00, 1.50, and 2.00) further narrow the Soret band and eliminate scattering in the 700–1,000 nm range, indicating reduced polydispersity and smaller nanoMOF sizes. Similarly, dynamic light scattering (DLS) data of colloidal dispersions of the purified solids presented in Figure S8B show a decrease in particle size and polydispersity with higher L/M ratios. Initial ratios (0.25 and 0.50) resulted in relatively large, polydisperse nanoMOFs, while higher ratios yielded smaller, highly monodisperse nanoMOFs. Details on hydrodynamic sizes and the corresponding polydispersity index (PdI) are provided in Table S8. Most notably, the chosen synthesis conditions achieved remarkably high reaction yields in terms of Zr consumed, ranging from ∼80% to nearly 100% for L/M ratios of 0.50 and 1.00, as determined by inductively coupled plasma optical emission spectroscopy (ICP-OES) measurements of Zr content post-purification of the solids (Figure S8C).

Vibrational spectroscopy techniques, including Fourier transform infrared spectroscopy (FTIR spectroscopy) and Raman, revealed no significant differences among the samples (Figures S9 and S10). However, differential scanning calorimetry (DSC) analysis produced two distinct sets of thermograms (Figure S11). Specifically, the samples with larger L/M ratios (1.00, 1.50, and 2.00) exhibited a single endothermic peak around 140°C. In contrast, the samples with lower L/M ratios (0.25 and 0.50) demonstrated three endothermic peaks, with the most intense peaks occurring at 260°C and 250°C, respectively. These findings align with results from previous characterization techniques, indicating that the samples with lower L/M ratios (0.25 and 0.50) differ from those with higher L/M ratios (1.00, 1.50, and 2.00).

The emission spectra of all prepared samples exhibit two bands when excited at both their Soret band (excitation at 420 nm) and Q band (excitation at 515 nm) regions: a first band located at ∼650 nm and a second band at around 720 nm, assigned to Q_00_ and Q_01_ transitions, respectively. The normalized emission spectra of the porphyrinic MOFs (Figure S12) demonstrate consistent emission behavior, regardless of excitation at the Soret or Q band. As displayed by TCPP emission, Q_01_ is typically less intense than the Q_00_ band because it involves vibrational excitation in the ground state, making it an overtone transition. However, as the ratio of the components (referring to the L/M ratio) increases, the intensity of the Q_01_ emission band (∼720 nm) over the Q_00_ emission band (∼650 nm) coherently increases. This suggests that higher L/M ratios enhance the electronic environment, favoring changes in oscillator strength due to an increased incorporation of coordinated TCPP molecules. This change potentially leads to enhanced electronic coupling, altered vibrational relaxation pathways, and variations in structural rigidity and symmetry, affecting the radiative transitions of the porphyrinic system. Moreover, the increase in TCPP coordination number from 6-c (L/M = 0.25) to 12-c (L/M = 2.00) results in a different spatial arrangement and orientation of the porphyrin units within the MOF framework. Such changes in dihedral angles and increased structural rigidity modify the electronic environment, leading to variations in the emission spectra akin to those observed in simpler porphyrin aggregates.

### Effect of temperature and alkoxide precursor variation on PCN-224 synthesis

Once confirmed that using the Zr(OEt)_4_ at RT enables the production of single-phase polydisperse (PdI > 0.3) PCN-224 and monodisperse (PdI < 0.1) MOF-525 nanocrystals, we decided to explore new synthetic conditions to reduce the polydispersity of nanosized PCN-224. Aiming to produce PCN-224 nanocrystals with sizes around 100 nm and low polydispersity, as in the previous section, we set the L/M ratio to 0.25, fixed the reaction time at 1 h, and used AA as the modulator. However, we substantially decreased the Mod/M ratio to 250, aiming to reduce the nanocrystal size, and varied the temperature (Figures S13 and S14). To this end, we explored five temperature settings ranging from RT to 120°C, the latter being widely used to produce MOFs based on the Zr_6_-oxo cluster. As anticipated from Figure S13, relatively monodisperse nanoparticles are obtained across the entire temperature range, with their size increasing as the temperature rises, with particle size ranging from 50 to 200 nm.

By examining the reaction yields in terms of Zr consumed (Figure S14A), we observed that when moving from RT to 75°C, the reaction yield moved from 50% to 85% of added Zr, as quantified by ICP-OES (Table S9). Additionally, we noted that increasing the temperature led to subtle changes in the optical properties. Specifically, there was a progressive broadening of the Soret band and an enhancement of the scattering baseline observed (Figure S14B). These changes are attributed to an increase in particle size and polydispersity, as qualitatively confirmed through scanning electron microscopy (SEM) (Figure S13) and DLS in Figure S14C and Table S10.

**Figure 3:**
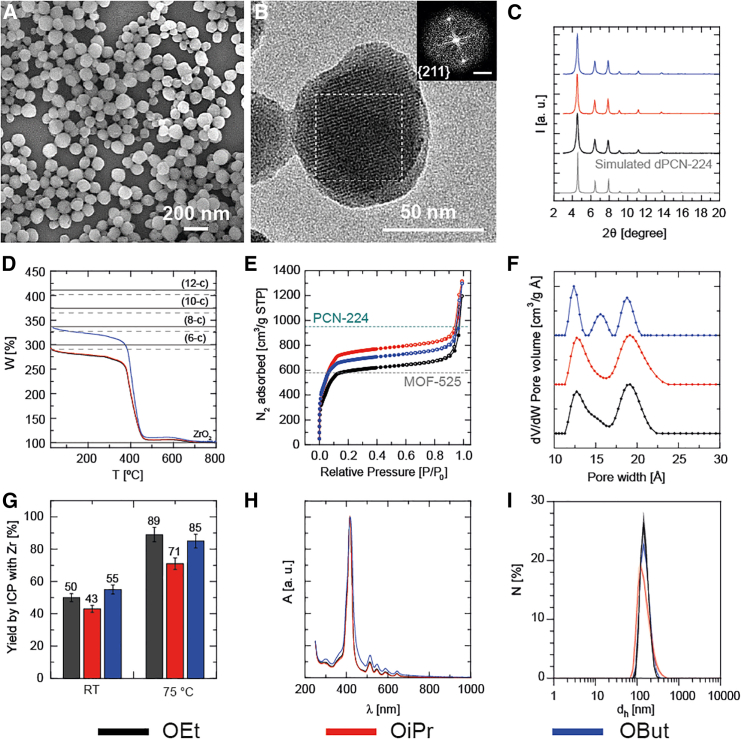
Characterization of nanosized PCN-224: Effect of temperature and alkoxide in the synthesis (A and B) Representative SEM (A) and HR-TEM (B) with FTT images of nanoMOFs synthesized starting from Zr(OEt)_4_ precursor at 75°C for 1 h in the presence of AA. (C) PXRD patterns. (D) Normalized TGA with the theoretical molar mass of Zr_6_-oxo cluster with different coordination degrees with TCPP and acetate molecules (dashed lines are the dehydrated cluster). (E) N_2_ adsorption isotherms at 77 K with the expected uptake values for ideal PCN-224 and MOF-525 phases calculated with Zeo++.^37^ (F) NLDFT pore size distribution by using different Zr precursors. (G) Graphical representation of the reaction yields evaluated through ICP-OES of 1 h reaction conducted at RT and 75°C. (H and I) UV-vis extinction spectra (H) and hydrodynamic diameters d_h_ (by number) (I). Scale in FFT represents 0.5 nm^−1^.

To go one step further and analyze the impact of different alkoxide precursors in the reaction that allow us to produce nanoMOFs (Figures 3A and 3B), we decided to fix the temperature at 75°C, keep the rest of the parameters unchanged, and employ other Zr precursors besides the ethoxy, such as Zr(OiPr)_4_ and Zr(OBut)_4_ (Figures S15 and S16; Table S11). We confirmed the crystalline structure of these solids through PXRD analysis, which revealed that the use of different Zr precursors led to the formation of virtually the same crystalline phase (Figure 3C). Moreover, HR-TEM confirmed the crystallinity of all nanoMOFs (Figure 3B) obtained, as can be seen with FFT analysis on designated ROIs (dashed ROI), providing a critical d-spacing of 16.528 Å compatible with the {211} family of planes for PCN-224, which are not present in MOF-525 or dPCN-224 due to the inherent symmetry in MOF-525 or forbidden reflections resulting from the disorder in the orientations of Zr_6_-oxo clusters in dPCN-224; these features were not observed in the PXRD pattern (Figure 3C). Although visual inspection of PXRD analysis does not unequivocally differentiate between PCN-224, MOF-525, and dPCN-224,^14^ our results align with the initial expectations of using an L/M ratio of 0.25 (Figure S17). Pawley refinement supports the presence of the PCN-224 phase, reinforcing the interpretation of the normalized TGA thermograms (Figure 3D). This is further evidenced by the exclusive presence of the {211} family of planes in PCN-224. Additionally, the absence of supercell peaks at 3.2° and 5.6° two-theta can be attributed to disorder in defects and/or variations in cluster orientation compared to the ordered structure of PCN-224, which gives the disordered phase dPCN-224 as a result.^14^

N_2_ adsorption isotherms of the materials revealed the microporosity of the nanoMOFs, with BET surface areas reaching values between 2,600 and 3,000 m^2^/g, closely aligning with expectations for PCN-224 (Figures 3E; Table S12).^10^ The pore size distribution analysis (Figure 3F), regardless of the alkoxy precursor used, revealed the presence of two primary pore sizes, centered at ∼13 and ∼19 Å (Figure S7; Table S7) with an extra pore size around 15 Å. This distribution is more closely aligned with the characteristics of PCN-224 rather than those of MOF-525. The presence of some degree of defectivity in our samples was confirmed by TGA, which nevertheless showed cluster connectivity indexes compatible with PCN-224 (6-c) but far from that of MOF-525 (12-c) (Figure 3D).

The 1 h reaction conducted at 75°C displayed high reaction yields (>70%) with all the alkoxide precursors (Figure 3G; Table S13).

In all cases, the characteristic optical signature of porphyrin nanoMOFs could be clearly observed (Figure 3H), with a dominating, very narrow Soret band (at ∼420 nm) that is significantly more intense compared to the weaker Q bands (500–630 nm), imparting to the colloidal solution a purple/violet color (Figure S14D) easily distinguishable with respect to the starting red TCPP solution. All the produced nanoMOFs presented superimposable extinction spectra, suggesting similar outcomes despite the different Zr precursors. The average d_h_ (hydrodynamic diameter by number distribution) was about 150 nm, with a relatively low PdI (<0.15), demonstrating the uniformity in particle d_h_ (Figure 3I; Table S14).

For completeness, we assessed the impact of temperature on the crystallinity and particle size of the nanoMOFs and conducted the reactions with the three different alkoxy precursors at RT (Figures S18 and S19; Table S15). Under these conditions, the 1 h reaction reached a yield of 50%, regardless of the alkoxide used (Figure 3G; Table S16). While the optical properties were comparable to those of particles synthesized at 75°C (Figure S20A), a notable reduction in d_h_ to ∼100 nm was observed (Figure S20B; Table S17). Most notably, the TGA thermograms closely resemble those obtained at 75°C, even when using Zr(OBut)_4_ (Figure S20C), which we tentatively associate with the presence of missing cluster defects. Moreover, the crystalline phase of the systems synthesized at RT was similar to that of the solids obtained at 75°C (Figure 3C), consistent with dPCN-224 (Figure S20D), but with considerably lower crystallinity as expected due to the low temperature. However, a pronounced decrease in BET surface areas was observed (Figures S20E and S20F).

Aiming to improve the reaction yield, the RT reaction was extended to 24 h. As anticipated, this adjustment led to an increase in reaction yields up to 70% (Table S16) without compromising the optical properties and crystalline phase of the synthesis conducted at 75°C (Figures S21A and S21B). Despite these adjustments, there was no significant enhancement in the BET surface areas, which remained indicative of a semi-condensed phase (Figures S21C and S21D; Table S18), echoing the outcomes observed with the 1 h of reaction.

### Effect of modulator on PCN-222 synthesis

Despite PCN-222 sometimes being found during zirconium halide synthesis of Zr-oxo MOFs, this phase was systematically absent during all our previous experiments. It has been previously reported that subtle changes, not only in reaction conditions but also in the modulator used, can lead to substantially different phase proportions in Zr-oxo MOFs.^43^ To demonstrate the capability of introducing an additional phase through minor modifications in the experimental setup, we substituted the AA modulator with FA and systematically varied the L/M (0.35–1.5) and Mod/L (100, 250, and 560) ratios, the temperature (25°C–75°C), and the Zr precursor (OEt, OiPr, or OBut).

Conducting the reaction at RT for 24 h with any of the Zr precursors at L/M = 0.35, the ideal L/M ratio for PCN-222, and Mod/M = 100 did not yield a solid product. However, at 75°C, solid particles were formed after 24 h (Figure S22A), with almost quantitative yields observed thereafter (Figure S22B; Table S19). The particles exhibited an elongated morphology (Figures S23 and S24; Table S20), suggesting a crystalline phase distinct from those observed in previous sections. Figure S22F presents PXRD diffractograms of Zr-porphyrinic nanocrystals synthesized with different Zr alkoxides and shows that the three samples comprise a phase mixture of MOF-525 and a partially formed phase PCN-222, as corroborated by Pawley refinement (Figure S25), which agrees with TGA showing a plateau slightly above the 8-c line (Figure S22E). Although this refinement does not allow for the quantification of each phase, it is clear that Zr(OEt)_4_ predominantly favors the formation of PCN-222, evident from the intensity of its reflections (Figure S25A) and the predominantly elongated particle morphology (Figure S23A). This contrasts with Zr(OiPr)_4_ and Zr(OBu)_4_, where PXRD patterns indicate a greater presence of the MOF-525 phase (Figures S25B and S25C), aligning with FE-SEM images that show more pseudospherical particles (Figures S23B and S23C), all of them with sizes around 150 nm (Table S21) and BET surface areas below the canonical PCN-222 phase (Table S22). In the case of Zr(OEt)_4_, with an increase in the L/M ratio, there was a gradual transition toward predominantly pseudospherical particles, indicating a loss of the elongated shape characteristic of PCN-222. Conversely, with a Mod/M ratio of 560 (Figure S26), an increase in the L/M ratio noticeably favored the formation of rod-like shaped particles (Figure S27). Particularly at an L/M ratio of 1.5, there was a marked shift toward the exclusive presence of elongated nanocrystals, highlighting a clear correlation between the Mod/M ratio, the L/M ratio, and the resulting particle morphology (Figure S27).

**Figure 4:**
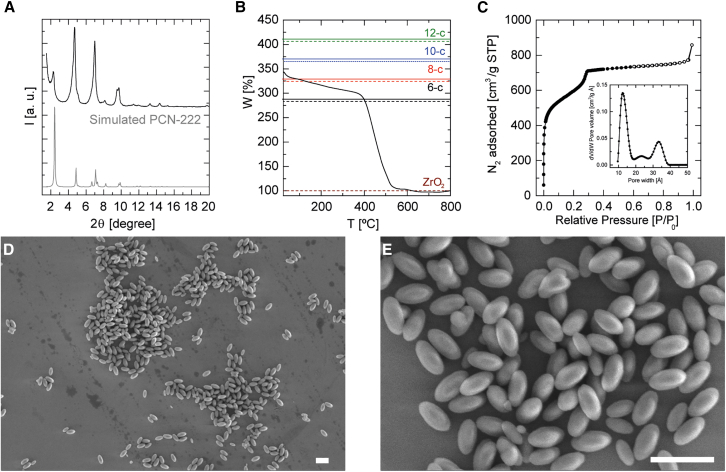
Characterization of porphyrinic MOF nanoparticles using Zr(OEt)_4_ as precursor and FA as modulator (L/M = 1.5 and Mod/M = 560) at 75°C for 1 h (A) PXRD diffractogram. (B) Normalized TGA with theoretical lines of defects for Zr_6_-oxo cluster (dashed lines are the dehydrated cluster). (C) N_2_ adsorption isotherm at 77 K (with pore size distribution plot inserted using NLDFT model). (D and E) FE-SEM images at (D) low and (E) high magnification of PCN-222 nanoMOFs. Scale bars represent 1 μm.

Encouraged by the promising outcomes, we undertook a comprehensive characterization of the system with L/M = 1.5 and Mod/M = 560, detailed in Figure 3. Further structural insights were obtained from PXRD analysis (Figure 4A), wherein our sample’s diffractogram was compared against simulated patterns for PCN-222. Pawley refinement (Figure S28) facilitated the determination of lattice parameters as a = b = 41.905 Å and c = 17.206 Å. These measurements align closely with the established cell parameters for PCN-222, a = b = 41.968 Å and c = 17.143 Å, within the P6/mmm space group, underscoring the precision of our synthetic approach. This was further confirmed by HR-TEM analysis, and after applying the FFT to the selected region (indicated by a dashed line), the d-spacings corresponding to the crystalline planes were determined (Figure S22A). Specifically, d-spacings were found to be 8.620 Å for the {002} family of planes and 11.600 Å for the {300} family of planes. These measurements are indicative of the hexagonal phase of PCN-222.

TGA (Figure 4B) indicated a coordination of ∼8 TCPP molecules per Zr_6_-oxo cluster, which agrees with the connectivity of the canonical PCN-222 phase. Moreover, the BET surface area measurement presented in Figure 4C, 2,142 m^2^/g, along with a pore size distribution featuring diameters of 1.2 and 3.3 nm (Table S7; Figure S7), closely mirrors the reported values for PCN-222.^44^ These figures closely match the reported values for canonical PCN-222, validating the successful synthesis and characterization of the material and its compliance with the anticipated structural and physicochemical properties of PCN-222.

The FE-SEM images (Figures 4D and 4E) consistently revealed the presence of elongated nanoparticles across the sample, with the particles displaying characteristic optical properties of nanoMOFs (Figure S22C) and a d_h_ of ∼150 nm (Figure S22D; Tables S20 and S21), highlighting the unique morphology achieved under these conditions.

### Interlaboratory reproducibility study

As previously discussed, achieving reproducibility in the synthesis of porphyrinic Zr-based MOFs has posed significant challenges.^21^ To validate the reproducibility of our synthesis method, the specific conditions from each experimental section were replicated by another research group using Zr(OEt)_4_ as the precursor. The porphyrinic Zr-MOF structure, as outlined in Table S2, was confirmed through PXRD analysis of PCN-224, which showed no supercell peaks (Figure S29A), consistent with the findings in Figure 2C. Similarly, the structure in Table S1, which used an L/M ratio of 0.25, exhibited distinct supercell peaks of PCN-224 (Figure S29B), in alignment with the data shown in Figures 1A and 1B. Moreover, the PCN-222 structure from Table S3 using an FA/Zr ratio of 560 was analyzed and confirmed (Figure S29C), validating the results in Figure 3A. These interlaboratory findings further strengthen the reproducibility of our synthesis method using Zr-alkoxide precursors.

### Continuous-flow reaction

Batch processes have traditionally been the mainstay for producing MOFs.^45^^,^^46^^,^^47^ These conventional methods are noted for their high costs, limited scalability, and prolonged durations, which can range from several hours to days.^48^^,^^49^^,^^50^^,^^51^ Despite progress in scaling up MOF synthesis, industrial production still faces significant challenges.^52^ Consequently, there is a pressing need for more efficient MOF preparation methods. Continuous processing has been recognized for significantly improving space-time yields (STYs) under analogous reaction parameters.^53^^,^^54^ Recent advancements in continuous synthesis include techniques such as spray drying,^55^^,^^56^^,^^57^ mechanochemical,^58^^,^^59^ flow methods,^60^^,^^61^^,^^62^^,^^63^^,^^64^^,^^65^^,^^66^ and microwave-^53^^,^^67^ and sonication-assisted methods,^68^ along with the use of supercritical fluids,^66^ among others.^69^^,^^70^ Notably, microfluidic and millifluidic technologies offer rapid mixing, enhanced heat transfer and energy efficiency, precise control over reaction parameters, and the capability to produce various configurations of MOF composites.^62^^,^^71^^,^^72^

A few studies have explored continuous-flow synthesis for Zr-MOFs, utilizing high temperatures that incur elevated operating costs.^73^^,^^74^ In contrast, as demonstrated in this work, using alkoxide precursors enables the ultrafast (second-scale) production of nanosized Zr-based porphyrinic MOFs at RT. Notably, traditional precursors like ZrCl_4_ and ZrOCl_2_ remained unreacted after 1 h at RT, as shown in Figure S30.

To showcase this method’s potential due to the high surfaces areas and catalytic activities of Zr-porphyrinic MOFs,^7^^,^^8^^,^^9^^,^^10^ continuous synthesis was carried out using a homemade flow reactor with two syringes, one with Zr(OEt)_4_ dissolved in a mixture of HOAc and DMF and the other with TCPP ligand dissolved in DMF. Both reagents were infused through hoses with an internal diameter of 1.8 mm until they mixed in a Y-shape tube with a resilience time of 25 s, as detailed in Figure S31.

**Figure 5:**
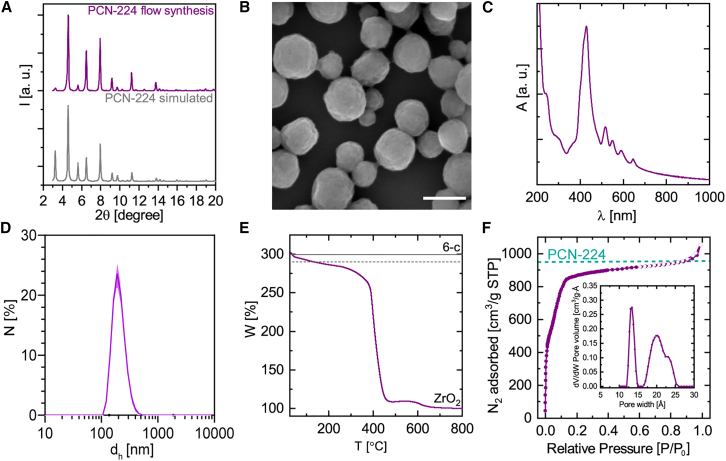
Continuous synthesis of nanosized PCN-224 using a homemade flow reactor (A) PXRD diffractogram with the simulated pattern of PCN-224. (B) Representative SEM image of PCN-224 synthesized by continuous-flow synthesis. (C) UV-vis extinction spectra. (D) d_h_ (by number). (E) Normalized TGA showing the theoretical lines for 6-c Zr_6_-oxo cluster (solid line hydrated and dashed line dehydrated cluster). (F) N_2_ adsorption isotherms at 77 K correspond to the expected values for ideal PCN-224 phases calculated with Zeo++.^37^ Inset: pore size using NLDFT model. Scale bar represents 200 nm.

Satisfyingly, PXRD analysis of the resulting sample confirmed that the RT, 20 s residence time reaction successfully produces a pure and highly crystalline phase of PCN-224, with a cell parameter a = 38.4084 Å following Pawley refinement (Figure 5A and S32). SEM images showed pseudospherical nanoparticles of 100–200 nm (Figure 5B). These findings align with the UV-vis spectra, which display the characteristic optical fingerprint of porphyrin MOFs, including a broad Soret band (Figure 5C). The d_h_ determined by DLS was ∼200 nm (Figure 5D). TGA, normalized to the inorganic residue (ZrO_2_) set at 100%, is presented in Figure 5E and confirms the 6-c expected for PCN-224. Notably, the microporosity of PCN-224 remained high under rapid flow synthesis, with a BET surface area of 2,387 m^2^/g and a pore size distribution (Figure 5F, inset graph) reminiscent of that of nanosized PCN-224 crystals.

This proof-of-concept experiment underscores the feasibility of synthesizing PCN-224 at RT within seconds, highlighting the process efficiency. To evaluate the synthesis efficiency in the continuous-flow regime, the STY and the surface area production rate (SAPR), based on the Zr ICP-OES yield (7.8% ± 0.4%; 9.2 mg), were calculated. The resulting values, 78,122 kg m^−3^ day^−1^ for the STY and 1.9 × 10^11^ m^2^ m^−3^ day^−1^ for the SAPR, demonstrate a high-quality and rate of production of PCN-224 by the continuous multifluidic method, showing substantial potential for industrial application.

This study introduces an innovative approach using Zr(IV) alkoxides to facilitate the synthesis of porphyrinic nanoMOFs with remarkably high efficiency and speed. By utilizing Zr-alkoxide precursors, we have demonstrated the capability to rapidly produce nanosized MOF-525, PCN-224, and PCN-222 within minutes, achieving high yields and controlled phase purity under mild conditions. This method significantly surpasses traditional Zr precursors, which typically require longer reaction times and higher temperatures and yield less favorable outcomes, thereby addressing a major bottleneck in the large-scale production of MOFs.

By tuning the L/M and Mod/M ratios, as well as the modulator, we have gained precise control over the crystal phases. This advancement is crucial for understanding the factors that facilitate the production of specific phases, which has recently been a subject of controversy.

Furthermore, the successful synthesis of nanosized PCN-224 using a continuous multifluidic method at RT represents a significant leap forward. Our approach not only simplifies the MOF production process but also paves the way for the integration of MOFs into various applications, offering a scalable and economically viable option. The high surface areas and biocompatibility of these materials underscore their immense potential across a broad spectrum of technological and medical applications, promising to overcome previous limitations in MOF production. This work sets a new benchmark for the synthesis of porphyrinic MOFs and opens the door to their widespread industrial and scientific use.

## Experimental procedures

Detailed information on the characterization techniques utilized in this study is provided in the supplemental information. Comprehensive descriptions of all synthetic protocols, including reagent specifications, are also included in the supplemental information. The variations in linker-to-metal (L/M) ratios for synthesizing MOF-525, dPCN-224, and PCN-224, as well as the influence of different modulators on the formation of PCN-222 and the continuous-flow reaction conditions for obtaining PCN-224, are extensively documented in the supplemental information. Additionally, the digestion protocol used for ^1^H-NMR quantification is fully outlined therein.

## Resource availability

### Lead contact

Further information and requests for resources should be directed to and will be fulfilled by the lead contact, Pablo del Pino (pablo.delpino@usc.es).

### Materials availability

This study did not generate new unique reagents or materials.

### Data and code availability

The data underlying this study are available in the article and supplemental information or from the lead contact upon request.

## Acknowledgments

The authors are thankful for the financial support of the 10.13039/501100000781European Research Council (starting grant #950421), the 10.13039/501100000780European Union (European Union NextGeneration EU/PRTR; H2020-MSCA-ITN #860942), the MICIU/10.13039/501100011033AEI/10.13039/501100011033 (PID2023-152844NB-I00, PID2022-142338OB-100, and PID2020-119206RB-I00), and the 10.13039/501100010801Xunta de Galicia (#ED431C 2022/18, #ED431B2023/19, and Centro de Investigación do Sistema Universitario de Galicia accreditation 2023-2027 #ED431G 2023/03). J.M.V.-F. acknowledges the 10.13039/501100004837Spanish Ministry of Science and Innovation for his postdoctoral grant (IJC2020-044369-I). The authors are grateful for the use of RIAIDT-USC analytical facilities, particularly to Bruno Da Cuña Mariño (UNIDADE DE DIFRACCIÓN DE RAIOS X).

## Author contributions

M.C., G.Z., O.S., S.F.-H., and S.M.-G. conducted the experiments. J.M.V.-F. performed the TEM characterization. M.C., S.T., C.M.-G., T.D., B.P., and P.d.P. designed the experiments and wrote the paper. B.P. and P.d.P. secured the funding necessary for this work. The manuscript was written with contributions from all authors, and all authors have approved the final version.

## Declaration of interests

The authors declare no competing interests.

## Supplemental information

Document S1. Figures S1–S51, Tables S1–S22, Equation S1, and supplemental experimental procedures

Document S2. Article plus supplemental information
