# Supplementary material for: Ultrafast synthesis of zirconium-porphyrin framework nanocrystals from alkoxide precursors
Source: Cell Rep Phys Sci. 2024 Dec 18;5(12):102318. doi: 10.1016/j.xcrp.2024.102318 (PMC11659387; doi:10.1016/j.xcrp.2024.102318)
Supplement: Document S1. Figures S1–S51, Tables S1–S22, Equation S1, and supplemental experimental procedures [file mmc1.pdf]

Cell Reports Physical Science, Volume *5*

Supplemental information

Ultrafast synthesis of zirconium-porphyrin framework nanocrystals from alkoxide precursors

Manuel Ceballos, Giulia Zampini, Oleg Semyonov, Samuel Funes-Hernando, José Manuel Vila-Fungueiriño, Sonia Martínez-Giménez, Sergio Tatay, Carlos Martí-Gastaldo, Thomas Devic, Beatriz Pelaz, and Pablo del Pino

Ultrafast Synthesis of Zirconium-Porphyrin Framework Nanocrystals from Alkoxide Precursors

Manuel Ceballos,[1] Giulia Zampini,[2] Oleg Semyonov,[2] Samuel Funes-Hernando,[1] José Manuel Vila-Fungueiriño,[3] Sonia Martínez-Giménez,[4] Sergio Tatay,[4] Carlos Martí-Gastaldo,[4] Thomas Devic,[5] Beatriz Pelaz,[6] and Pablo del Pino[1,*]

[1]Centro Singular de Investigación en Química Biolóxica e Materiais Moleculares (CiQUS), Departamento de Física de Partículas, Universidade de Santiago de Compostela, 15782 Santiago de Compostela, Spain.

[2]Centro Singular de Investigación en Química Biolóxica e Materiais Moleculares (CiQUS), Universidade de Santiago de Compostela, 15782 Santiago de Compostela, Spain.

[3]Centro Singular de Investigación en Química Biolóxica e Materiais Moleculares (CiQUS), Departamento de Química Física, Universidade de Santiago de Compostela, 15782 Santiago de Compostela, Spain.

[4]Instituto de Ciencia Molecular (ICMol), Universitat de València, Catedrático José Beltrán-2, Paterna, 46980 Spain.

[5]Nantes Université, CNRS, Institut des Matériaux de Nantes Jean Rouxel, IMN, F-44000, Nantes, France.

[6]Centro Singular de Investigación en Química Biolóxica e Materiais Moleculares (CiQUS), Departamento de Química Inorgánica, Universidade de Santiago de Compostela, 15782 Santiago de Compostela, Spain.

Table of Content

Characterization techniques 2
Materials and Methods 5
Chemicals 5
Synthesis of MOF-525, dPCN-224 and PCN-224 5
Synthesis of PCN-222 7
Digestion of Zr-porphyrinic MOFs for ^{1}H-NMR quantification 8
Continuous PCN-224 synthesis procedure 8
Alkoxy (acetic acid) L/M: 0.25 – 2.0 Mod/M = 560 9
EtO (acetic acid) role of the temperature in the reaction yield 18
Alkoxy (acetic acid) 75 °C (1 h): L/M = 0.25 Mod/M = 250 20
Alkoxy (acetic acid) 25 °C (1 h vs 24 h): L/M = 0.25 Mod/M = 250 23
Alkoxy (formic acid) 75 °C 27
Reproducibility test 34
Continuous flow reaction 35
APPENDIX BETSI N_2 adsorption analysis 37
References 56

Characterization techniques

UV-Vis spectroscopy (UV-Vis)

UV-visible extinction spectra ranging from 200 to 1000 nm were recorded using an Agilent Cary 3500 Multicell UV-Vis Spectrophotometer with the nanoparticles dispersed in MeOH. The measurements were performed using a 1 cm quartz cell, using pure MeOH as a blank.

Dynamic Light Scattering (DLS)

The hydrodynamic diameter (D_h) and polydispersity index (PDI) were determined using Dynamic Light Scattering (DLS) with a Malvern Zetasizer Ultra-Red instrument. The DLS measurements were conducted with a 10 mW He-Ne laser operating at a wavelength of 633 nm. Multiple scattering angles were utilized through the application of the Multi-Angle Dynamic Light Scattering (MADLS) technique.

Thermogravimetric analysis (TGA)

Thermogravimetric analysis (TGA) was performed using a TA Instruments Q5000 IR thermobalance. The TGA measurements followed a standard heating profile from 25 to 800 °C, with a heating rate of 5 °C min^{-1} under an air atmosphere and a gas flow rate of 25 mL min^{-1}. The samples were washed with MeOH three times to remove residual non-volatile DMF solvent and subsequently dried at 70 ºC for 24 hours to ensure complete solvent removal prior to measurement.

Differential Scanning calorimetry (DSC)

DSC measurements were performed using a TA Instruments Q200 calorimeter with a flow nitrogen flow rate of 50 mL min^{-1}. Around 0.5-1.0 mg of samples were place in aluminum crucibles hermetically closed and an empty aluminum crucible also hermetically closed were please as reference.

Inductively Coupled Plasma-Optical Emission Spectrometry (ICP-OES)

Elemental analysis was carried out using an Agilent 5800 Inductively Coupled Plasma Optical Emission Spectrometer (ICP-OES) due to its suitability for quantitative elemental analysis. Calibration curves were established with a concentration range of 0 to 10 ppm for Zr. The ICP-OES detector utilized the atomic emission lines of Zr at 339.198 nm and 343.823 nm.

Before analysis, sample preparation involved dilution and acid digestion. Specifically, 100 µL of the sample were diluted into 1.2 mL of nitric acid (67 wt%) and 3.5 mL of hydrochloric acid (37 wt%). Additionally, 100 µL of Mn solution (500 ppm) and 100 µL of Se solution (500 ppm) were added as internal standards. The entire mixture, totaling 5 mL, was transferred

into a Teflon (PTFE-TFM) tube, specifically an HVT50 tube from Anton Paar. The digestion step was carried out using an Anton Paar Multiwave GO Plus microwave heating system at 185 °C for 15 minutes.

Following digestion, 500 µL of the sample were further diluted into 4.5 mL of water, resulting in a final dilution factor of 500x. This diluted solution was then injected into the ICP-OES instrument, and the software provided the results in parts per million (ppm) for Zr concentration.

Scanning Electron Microscopy (SEM)

Microscopy images were acquired with a Zeiss Ultra Plus Field Emission Scanning Electron Microscope (FE-SEM) operating at acceleration voltages of 3 kV. Image acquisition was performed using the InLens detector.

Transmission Electron Microscopy (TEM)

TEM images were captured with a JEOL JEM F200 microscope, which was equipped with a Gatan OneView camera and a cold-field emission gun (FEG) operating at an accelerating voltage of 80 kV. For TEM specimen preparation, a drop of diluted samples was added to a 400-mesh Cu grid and allowed to dry.

Powder X-Ray Diffraction (PXRD)

Crystalline powder underwent Powder X-Ray diffraction (PXRD) analysis at room temperature using a Bragg-Brentano geometry on a "Bruker D8 Advance" X-ray diffractometer (40 kV, 40 mA, θ/θ configuration). The diffractometer was equipped with a sealed Cu X-ray tube ($\lambda_{CuK\alpha1}$ = 1.5406 Å) and a LYNXEYE detector. Diffractograms were generated within the angular range of $3<2\theta<40$, with a step size of 0.02° (2θ) at 2 seconds per step. Throughout the measurement, sample rotation optimized peak profiles for analysis and minimized the impact of preferred orientation. To prevent background noise from a glass support, samples were positioned on a Si(511) oriented crystal base.

Small-angle powder X-Ray Diffraction

Measurements of X-Ray diffraction at small angles were performed in a Malvern Panalytical-Empyrean with five-axis goniometer ("χ-φ-x-y-z stage"), with a sealed Cu tube (λ=0.154nm) and bicap W/Si parallel beam-generating optics with an acceptance angle of 0.8° and a length of 55.3 mm, equipped with an area detector type "PANalytical PIXcel-3D"

Fourier-transformed infrared spectroscopy (FTIR)

FTIR measurements were recorded using a PerkinElmer Spectrum Two spectrometer with Attenuated Total Reflection (ATR). The dried powders were placed on the ATR window for analysis within the wavenumber range of 700 to 4000 cm^{-1}.

N_2 adsorption–desorption analysis

Nitrogen (N_2) adsorption measurements were carried out using a Micromeritics 3Flex Adsorption Analyzer at 77 K. Before analysis, the samples were outgassed at 90ºC under high vacuum overnight. The amount of sample was around 20-30 mg powder. The specific surface area was determined by extrapolating within the relative pressure range of 0.05-0.3 (where P/P_0 represents the ratio of the measured pressure to the saturation pressure) using the Brunauer, Emmett & Teller (BET) equation. Pore size distribution was determined using a NLDFT (Non-local Density Functional Theory) model for Pillared Clay, considering cylindrical pores.

Data analysis was conducted using the 3Flex V5.03 software, developed by Micromeritics Instrument Corp. based in Norcross, GA, United States.

Analysis of BET surface area were performed with BESTI analysis following Rouquerol criteria.[1]

Raman spectroscopy

Raman measurements were conducted using a BWTEK i-RAMAN EX system on a glass substrate, employing a laser beam at 1064 nm and a 50× objective. The samples were exposed to a power of 160 mW with an acquisition time of 5 s and 10 accumulations.

NMR spectroscopy

^1H NMR spectra were obtained at room temperature employing a BRUKER AVIII 500 MHz spectrometer, with a frequency of 500 MHz. d1 = 20 s and 64 scans. The spectra were referenced to the residual solvent peak (D_2O, singlet, 4.80 ppm). Analysis of the spectra was performed using MestreNova© NMR data processing software. Chemical shifts (δ) are reported in ppm.

Photoluminescence spectroscopy (PL)

Photoluminescence (PL) emission spectra were performed using an Edinburgh-FS5-Spectrofluorometer from Edinburgh Instruments Ltd. Steady-state measurements involved the use of a Xenon lamp as the excitation source, and the recorded spectra were corrected for the instrument's response characteristics.

Materials and Methods

Chemicals

Zirconium ethoxide ($Zr(OEt)_4$, 97%), Zirconium(IV) isopropoxide isopropanol complex ($Zr(OiPr)_4$, 99.9% trace metals basis), Zirconium(IV) butoxide solution ($Zr(OBut)_4$, 80 wt. % in 1-butanol), formic acid (FA, ≥96%), Methylsulfonylmethane ($(CH_3)_2SO_2$, Pharmaceutical secondary standard) from Sigma-Aldrich. 5,10,15,20-(Tetra-4-carboxyphenyl)porphyrin (TCPP, 98%) from PorphyChem. N,N-dimethylformamide (DMF ≥99.8%), methanol (MeOH, LC/MS Grade), acetic acid glacial (AA, 99.7%), Sodium hydrogen carbonate ($NaHCO_3$) were purchased from Fischer Scientific. Deuterium oxide (D_2O, 99.8% D atoms, Acros Organics. All the chemical reagents were used without further purification.

Synthesis of MOF-525 and PCN-224

In 2 mL-vials, two solutions (A and B) are separately prepared and then mixed to start the reaction. In one solution it is dissolved the metal precursor (solution A – Zr alkoxide) together with the modulator (acetic acid), and in the other one the linker (solution B – TCPP or Tetrakis(4-carboxyphenyl) porphyrin). The two solutions are prepared in DMF.
The quantities are specified below, depending on the ratio L/M, the Zr precursor and Mod/M adopted.
In a vial, equipped with a magnetic stirrer, the solution B is added and put under magnetic stirring at 500 rpm; then, the solution A is quickly added, the vial is sealed, and the reaction is stirred at 75 °C (or room temperature) for 1 hour in dark condition.
The reaction is then centrifuged at 10000 g for 10 min; the recovered pellet is washed by centrifugation (10000 g, 10 min) 3-times with 1 mL of DMF and 2-times with 1 mL of MeOH. The final sample is resuspended in 1 mL of MeOH.

Used acronyms:

- L: linker (TCPP).
- M: metal precursor ($Zr(OEt)_4$ or $Zr(OiPr)_4$ or $Zr(OBut)_4$).
- Mod: modulator (acetic acid or formic acid).

All reagents were used under standard laboratory conditions without a glovebox, and the DMF was used without prior drying.

Table S1. Adopted quantities for the synthesis of PCN-224 and MOF-525 starting from $Zr(OEt)_4$ precursor.

	Entry	Ratio Mod/M 560				
**Sol. A**	$Zr(OEt)_4$	6.8 mg				
	AA	0.800 mL				
	DMF	0.200 mL				
	**Entry**	**Ratio L/M 0.25**	**Ratio L/M 0.5**	**Ratio L/M 1.0**	**Ratio L/M 1.5**	**Ratio L/M 2.0**
**Sol. B**	TCPP	5.0 mg	10.0 mg	20.0 mg	30.0 mg	40.0 mg
	DMF	1.000 mL	1.000 mL	1.000 mL	1.000 mL	1.000 mL

Table S2. Adopted quantities for the synthesis of dPCN-224 starting from different Zr precursor.

	Entry	Ratio Mod/M 250	Entry	Ratio Mod/M 250	Entry	Ratio Mod/M 250
**Sol. A**	$Zr(OEt)_4$	6.8 mg	$Zr(OiPr)_4$	9.7 mg	$Zr(OBut)_4$	11.5 µL
	AA	0.358 mL				
	DMF	0.642 mL				
	**Entry**	**Ratio L/M 0.25**				
**Sol. B**	TCPP	5.0 mg				
	DMF	1.000 mL				

Synthesis of PCN-222

In 2 mL-eppendorfs, the two solutions A (containing the modulator and the metal precursor) and B (with TCPP linker) are separately prepared and then mixed together to start the reaction.
In this case, the modulator is formic acid and the temperature is fixed at 75 °C.
The quantities specified below are relative to:

- ratio L/M = 0.33 and Mod/M = 100: 24h reaction.
- ratio L/M = 0.35-1.5 and Mod/M = 250 or 560: 1h reaction.

In a vial, equipped with a magnetic stirrer, the solution B is added and put at 75 °C under magnetic stirring at 500 rpm for 10 minutes; then, the solution A is quickly added, the vial is sealed and the reaction is stirred in dark condition for the proper time.
The reaction is then centrifuged at 10000 g for 10 min; the recovered pellet is washed by centrifugation (10000 g, 10 min) 3-times with 1 mL of DMF and 2-times with 1 mL of MeOH. The final sample is resuspended in 1 mL of MeOH.

Table S3. Adopted quantities for the synthesis of PCN-222.

	**Entry**	**Ratio Mod/M 100**	**Entry**	**Ratio Mod/M 100**	**Entry**	**Ratio Mod/M 100**
**Sol. A**	$Zr(OEt)_4$	6.8 mg	$Zr(OiPr)_4$	9.7 mg	$Zr(OBut)_4$	11.5 µL
	FA	0.095 mL				
	DMF	0.905 mL				
	**Entry**	**Ratio L/M 0.33**				
**Sol. B**	TCPP	6.5 mg				
	DMF	1.000 mL				

	**Entry**	**Ratio Mod/M 250**	**Ratio Mod/M 560**			
**Sol. A**	$Zr(OEt)_4$	6.8 mg	6.8 mg			
	FA	0.236 mL	0.529 mL			
	DMF	0.764 mL	0.471 mL			
	**Entry**	**Ratio L/M 0.35**	**Ratio L/M 0.5**	**Ratio L/M 0.75**	**Ratio L/M 1.0**	**Ratio L/M 1.5**
**Sol. B**	TCPP	7.0 mg	10.0 mg	15.0 mg	20.0 mg	30.0 mg
	DMF	1.000 mL	1.000 mL	1.000 mL	1.000 mL	1.000 mL

Digestion of Zr-porphyrinic MOFs for ^{1}H-NMR quantification

Quantification of TCPP/OAc^- was conducted by digesting approximately 2 mg of each sample (L/M ratios 0.25, 0.50, 1.00, 1.50, and 2.00) using a tip of a spatula in a 2 mL Eppendorf tube. Subsequently, 980 µL of a 1M $NaHCO_3$ solution prepared in D_2O were added, and the mixture was sonicated for 20 minutes to achieve complete digestion of Zr-porphyrinic nanoMOFs, as per the following reaction:[2]

$$\mathrm{Zr_6O_4(OH)_4(TCPP)_x(OAc)_y + 18HCO_3^- \rightarrow 3[Zr_2(OH)_2(CO_3)_4]^{2-} + 8H_2O + 6CO_2 + x\,TCPP + y\,OAc}$$

Following digestion, 20 µL 50 mM of methylsulfonylmethane ($(CH_3)_2SO_2$) were introduced into the solution as an internal standard, featuring a singlet peak for 6 protons at a chemical shift of 3.18 ppm.[3]

Continuous PCN-224 synthesis procedure

Stock solutions of ligand and metal precursor were prepared separately: TCPP (0.032mmol, 25mg) was dissolved in 5 mL of DMF and $Zr(OEt)_4$ (0.125 mmol, 34 mg) in a mixture of DMF (1 mL) and acetic acid (4 mL). Then both solutions $Zr(OEt)_4$ and TCPP were loaded in two discrete injection channels and were pumped using a syringe pump at room temperature with an individual flow rate 0.5 mL/min into PP Y-micromixer (I.D= 2.3mm) connected to 146 mm long silicone tube (1.8 I.D mm) giving a residence time of 25 s. The obtained purple powder was washed twice with DMF (1mL) and three times with MeOH (1 mL). The final sample is resuspended in 1 mL of MeOH.

Alkoxy (acetic acid) L/M: 0.25 – 2.0 Mod/M = 560

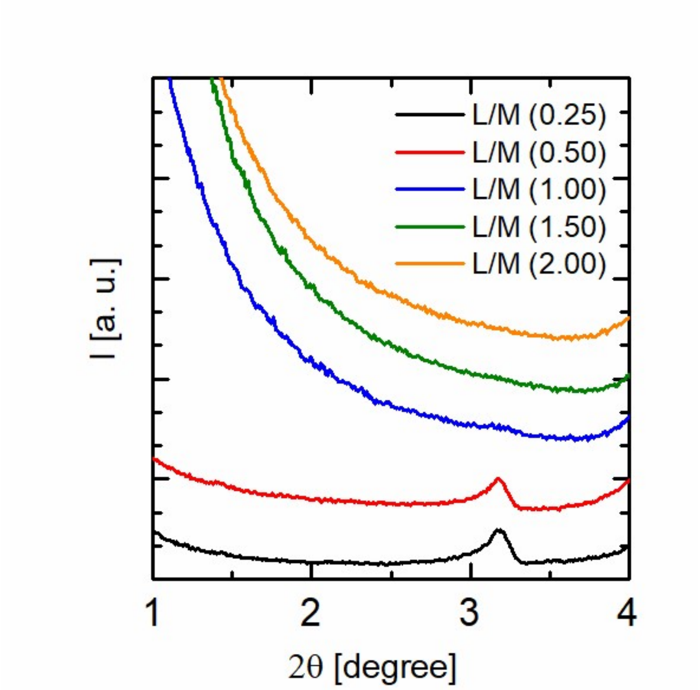

Figure S1. Small angle diffraction measurements of Zr-porphyrinic MOFs with different L/M ratios.

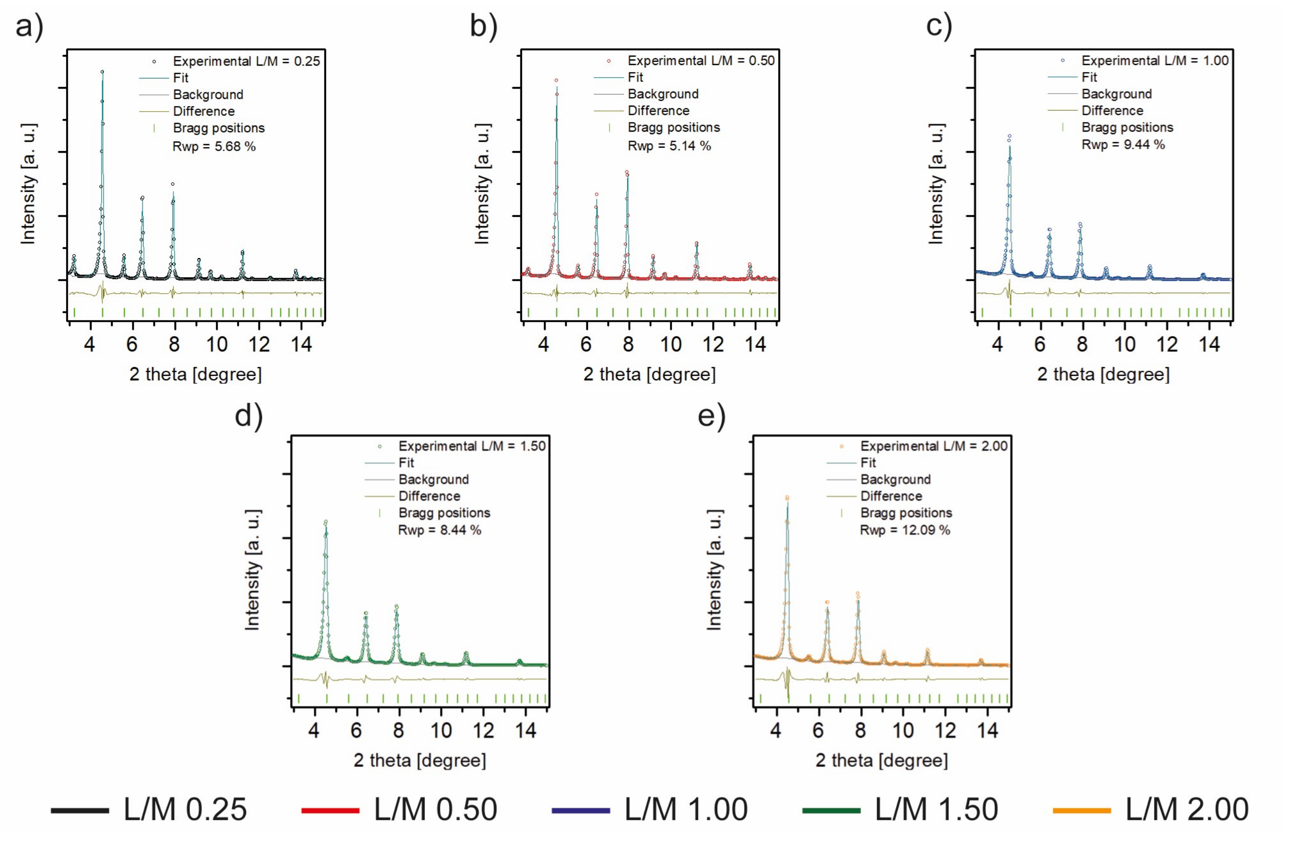

Figure S2. Pawley refinement for PCN-224 of Zr-porphyrinic MOF nanoparticles for a) 0.25, b) 0.50, c) 1.00, d) 1.50 and e) 2.00 L/M ratios.

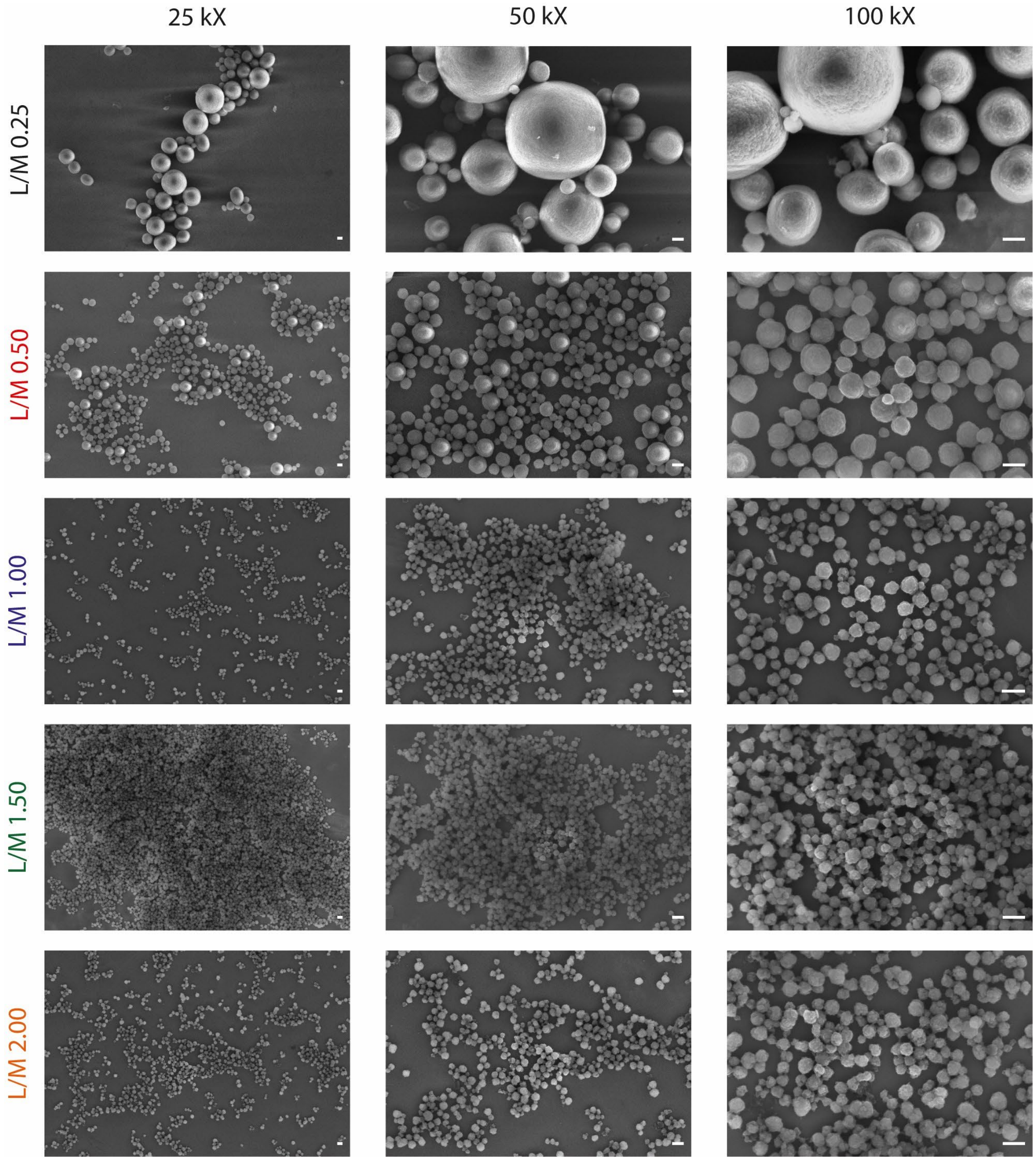

Figure S3. FE-SEM images of Zr-porphyrinic MOF nanoparticles at different magnifications (25 kX, 50 kX and 100 kX).

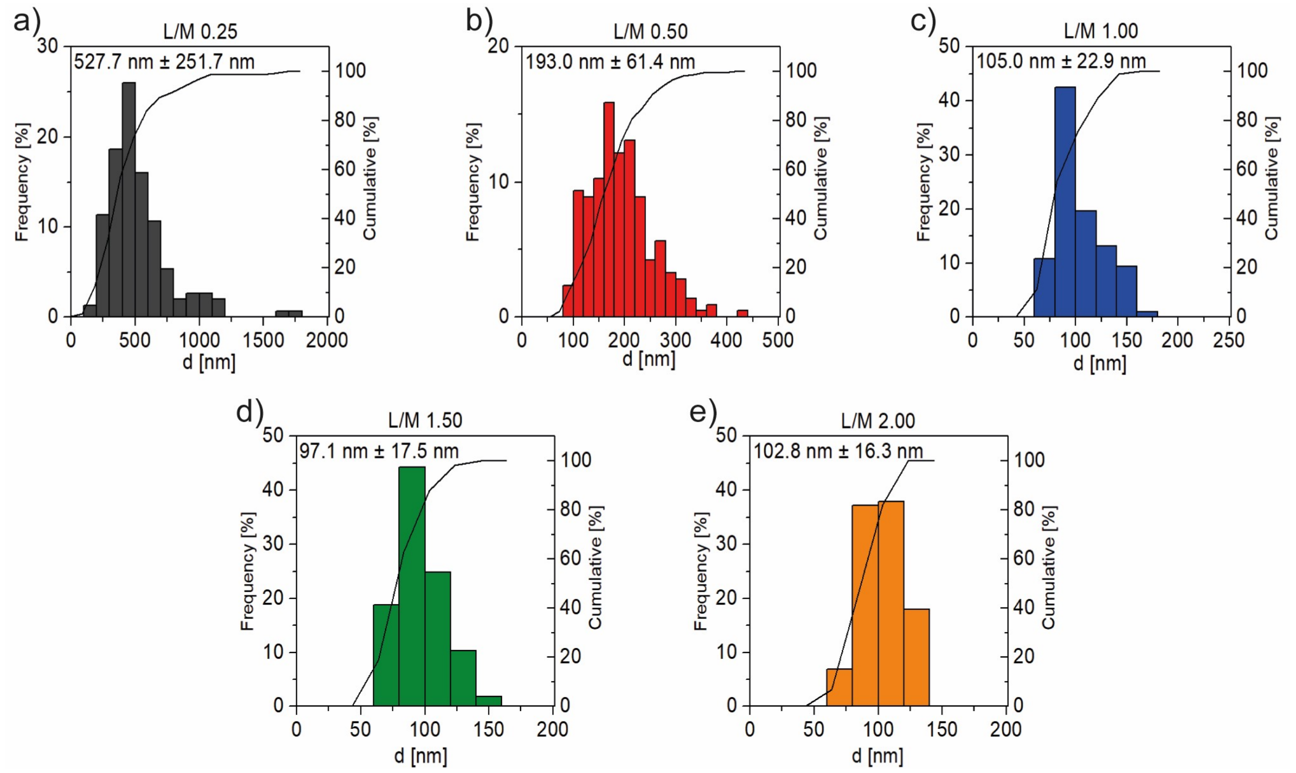

Figure S4. Particle size distribution histograms of Zr-porphyrinic MOF nanoparticles for a) 0.25, b) 0.50, c) 1.00, d) 1.50 and e) 2.00 L/M ratios.

$$D = \frac{K\lambda}{\beta \cos\theta} \qquad \text{Equation S1}$$

$$D = \text{Average crystallite size (nm)}$$

$$K = \text{Shape factor (0.94 for spherical crystallites with cubic symmetry)}$$

$$\lambda = \text{X} - \text{ray wavelength. Cu } K_{\alpha} \text{ average } = 1.54178 \text{ Å}$$

$$\beta = \text{FWHM (Full Width at Half Maximum)}$$

$$\theta = \text{XRD peak position, one half of } 2\theta$$

Table S4. Crystallite size of Zr-porphyrinic MOFs with different L/M ratios calculated by Scherrer equation.

L/M	Crystallite size (nm)
0.25	96.50
0.50	63.48
1.00	39.29
1.50	34.38
2.00	37.15

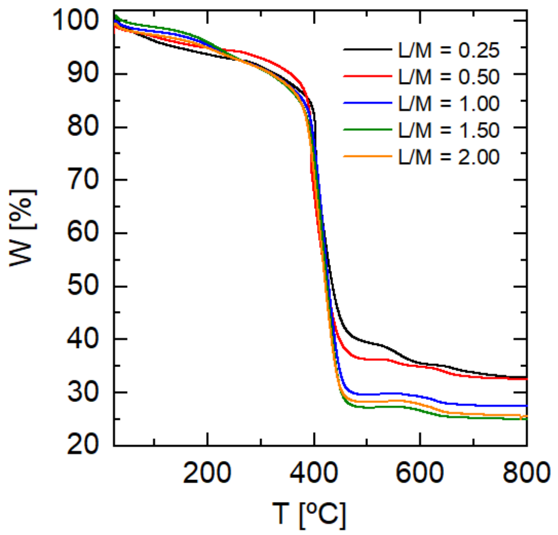

Figure S5. TGA without normalization of Zr-porphyrinic MOFs.

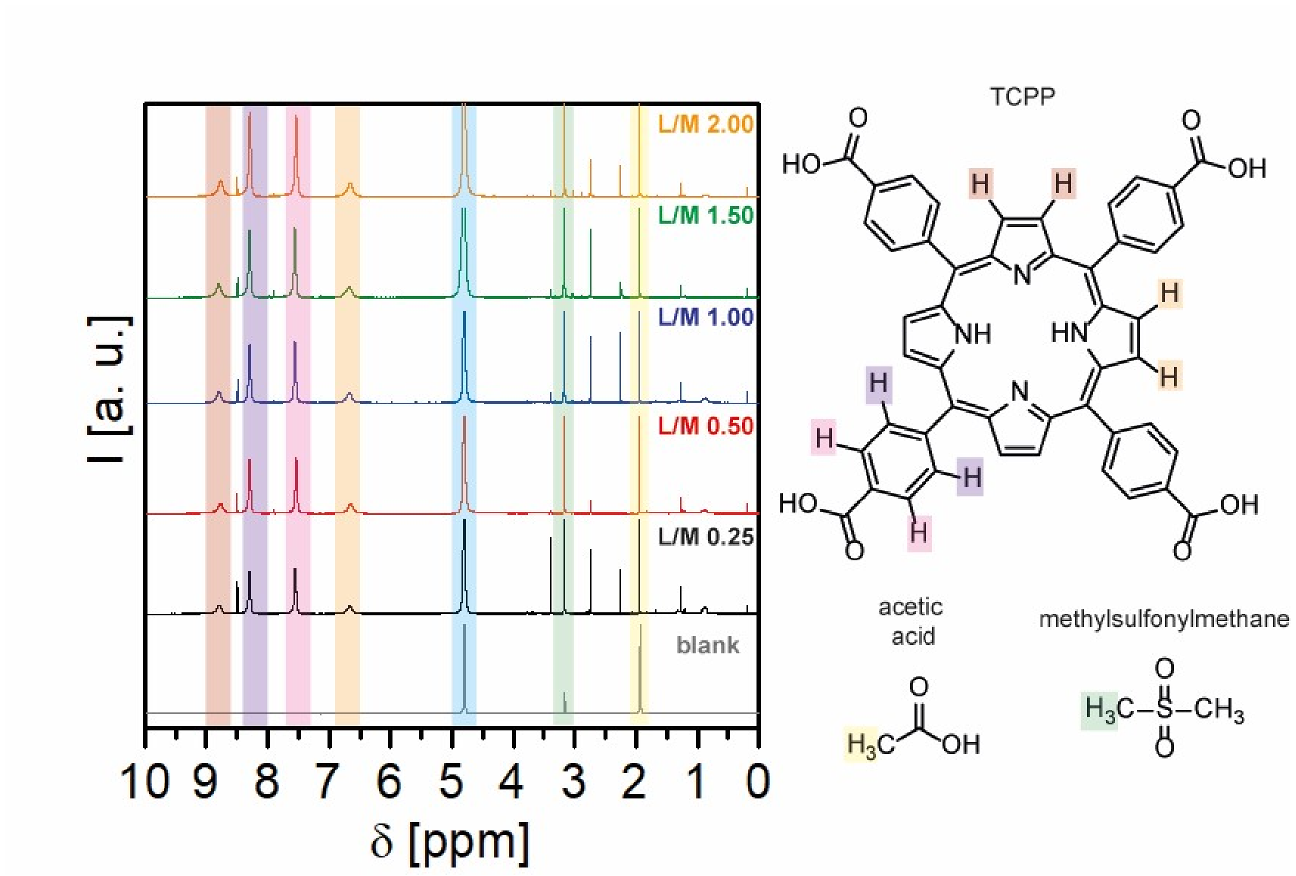

Figure S6. ^{1}H-NMR spectra of the blank (D_2O, $NaHCO_3$, CH_3COOH and $(CH_3)_2SO_2$) and Zr-porphyrinic MOFs. Signals not assigned correspond to remanent DMF and CH_3OH from the washing steps.[4]

Table S5. Assignation of ^{1}H-NMR spectra from Figure S10 and quantification using $(CH_3)_2SO_2$ as internal standard.

Sample	6H	3H	4H	4H	8H	8H	$(CH3)_2SO_2$ molecules	CH_3COO^- molecules	TCPP molecules	CH_3COO^- / TCPP
	$(CH_3)_2SO_2$	CH_3COO^-	pyrrol(1)	pyrrol (2)	benzene-o-porphyrin	benzene-m-porphyrin				
	(3.18ppm)	(1.95 ppm)	(8.79 ppm)	(6.66 ppm)	(8.30 ppm)	(7.55 ppm)				
L/M 0.25	1,95E+04	8,03E+04	-	-	1,37E+05	1,27E+05	6.0E+17	4,96E+18	3,06E+18	1,62
L/M 0.50	8,27E+04	7,89E+04			2,09E+05	2,08E+05		1,15E+18	1,14E+18	1,01
L/M 1.00	8,49E+04	2,46E+04			1,85E+05	1,82E+05		4,29E+17	9,76E+17	0,44
L/M 1.50	8,20E+04	2,33E+04			2,08E+05	1,98E+05		4,29E+17	4,46E+18	0,10
L/M 2.00	8,41E+04	3,01E+04			3,59E+05	3,63E+05		4,31E+17	2,00E+18	0,22

Table S6. Summary of N_2 uptake and BET surface area of Zr-porphyrinic MOFs with different L/M ratios at room temperature.

L/M	N_2 uptake (cm^3/g)	BET surface area (m^2/g)
0.25	991	2532
0.50	895	2428
1.00	670	2154
1.50	628	1987
2.00	631	2097

Table S7. Summary of different pore sizes of PCN-224, PCN-222 and MOF-525.

Zr-porphyrinic MOF	Space group	Cell parameter (Å)	Cluster connectivity	Pore width (Å) calculated with Zeo++[5]
**PCN-224**	$Im\bar{3}m$ (229)	a = 38.452	6-c	P_1 = 13.5<br>P_2 = 24.2
**PCN-222**	P6/mmm (191)	a = 41.968<br>c = 17.143	8-c	P_1 = 11.4<br>P_2 = 32.1
**MOF-525**	$Pm\bar{3}m$ (221)	a = 19.393	12-c	P_1 = 7.6<br>P_2 = 16.6

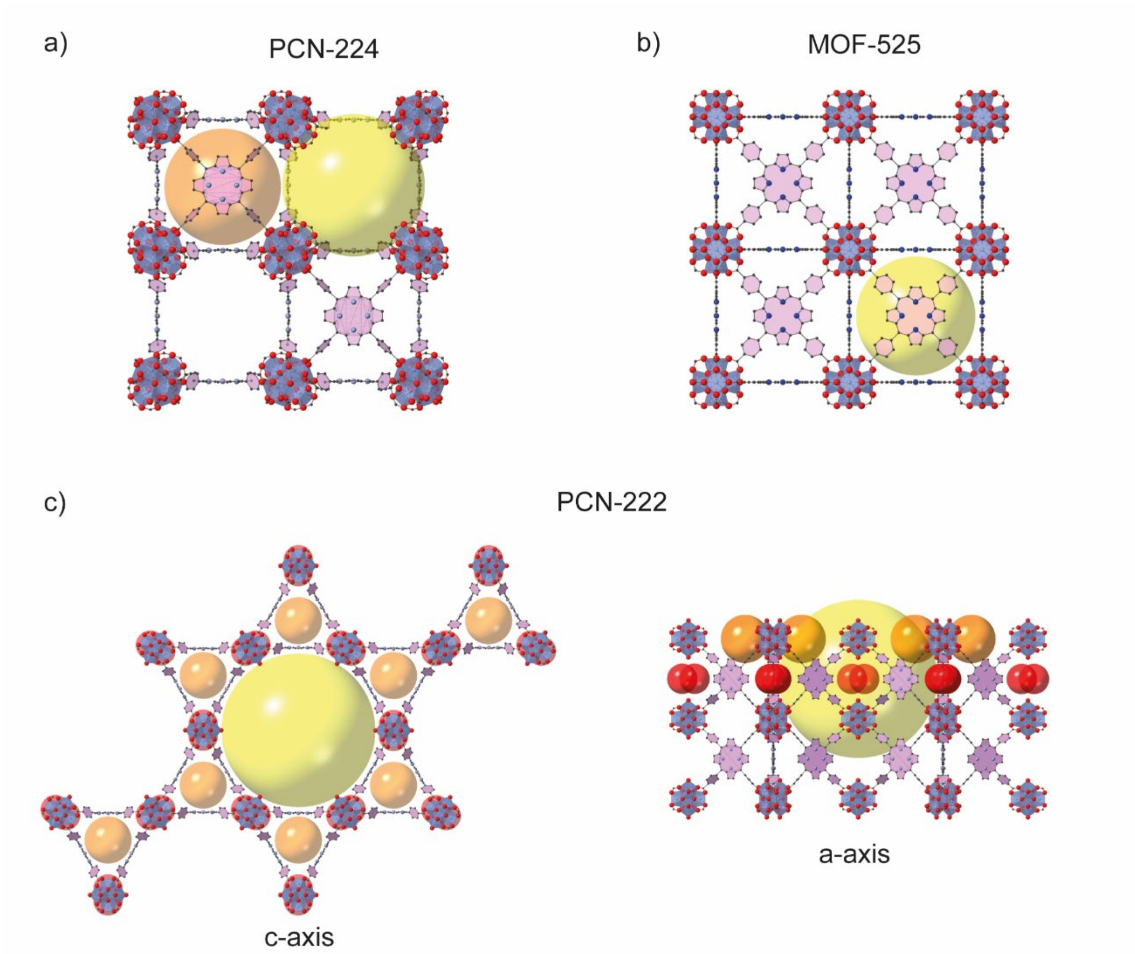

Figure S7. Pore size representation of a) PCN-224, b) MOF-525 and c) PCN-222.

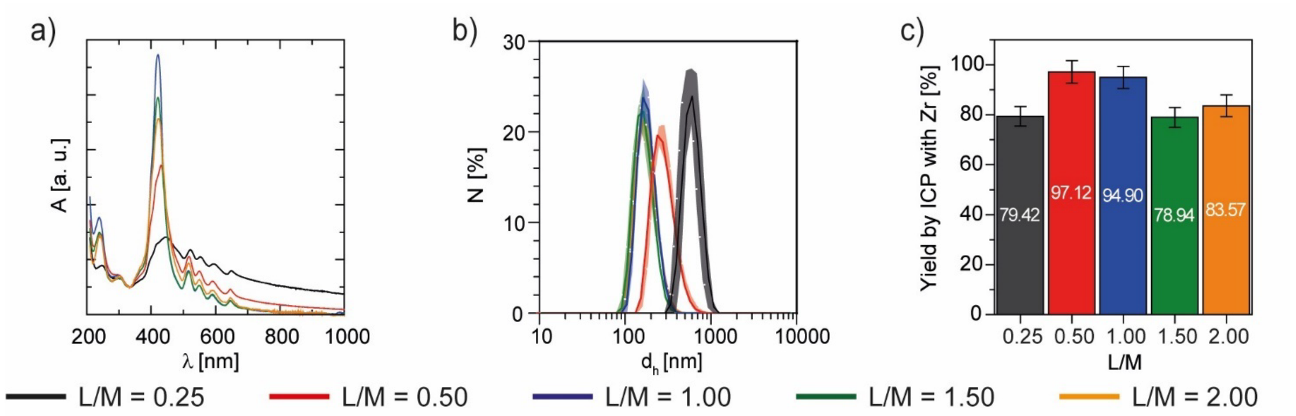

Figure S8. a) UV-Vis spectra, b) DLS analysis and c) yield of reaction obtained with the amount of Zr by ICP-OES of Zr-porphyrinic MOFs.

Table S8. Hydrodynamic size of Zr-porphyrinic MOFs with different L/M ratio measure by DLS in MeOH.

L/M	$D_{h,I}$ (nm)	$D_{h,V}$ (nm)	$D_{h,N}$ (nm)	PdI
0.25	636.0 ± 66.9	679.2 ± 78.7	618.1 ± 71.1	0.314 ± 0.050
0.50	386.8 ± 33.6	442.5 ± 93.2	307.7 ± 5.4	0.175 ± 0.044
1.00	216.2 ± 8.9	217.5 ± 9.2	186.1 ± 3.7	0.185 ± 0.023
1.50	200.7 ± 4.7	200.5 ± 4.3	168.7 ± 5.2	0.064 ± 0.043
2.00	222.5 ± 2.7	223.9 ± 2.9	180.9 ± 4.8	0.099 ± 0.038

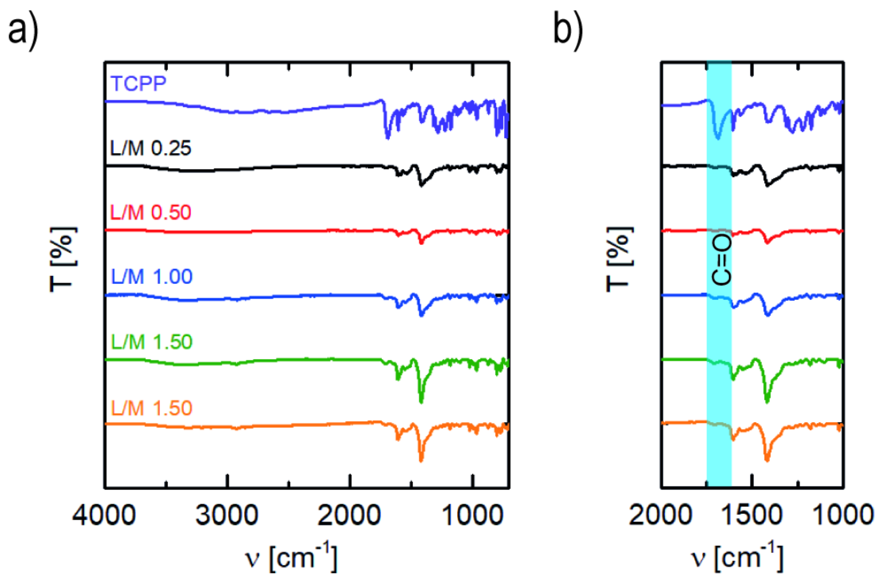

Figure S9. a) FT-IT spectra of Zr-porphyrinic MOFs and b) zoom in carbonyl region.

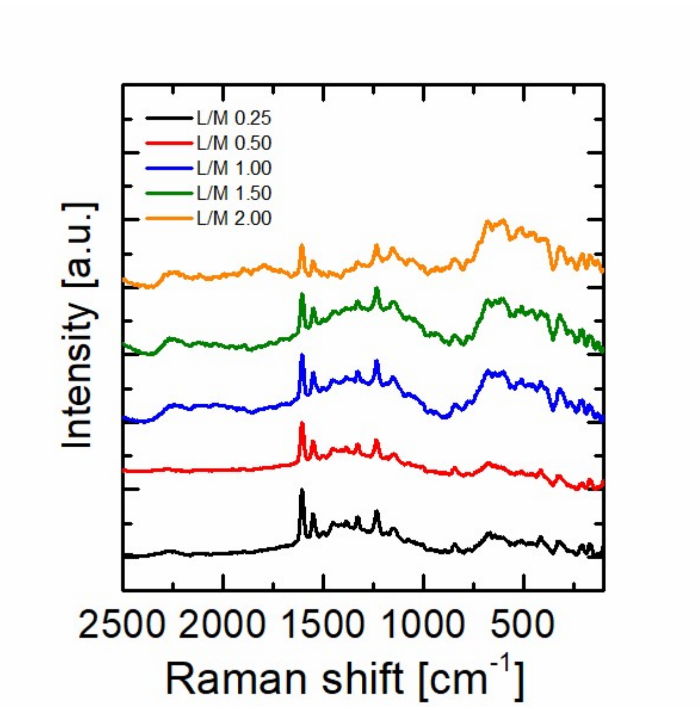

Figure S10. Raman spectra of Zr-porphyrinic MOFs.

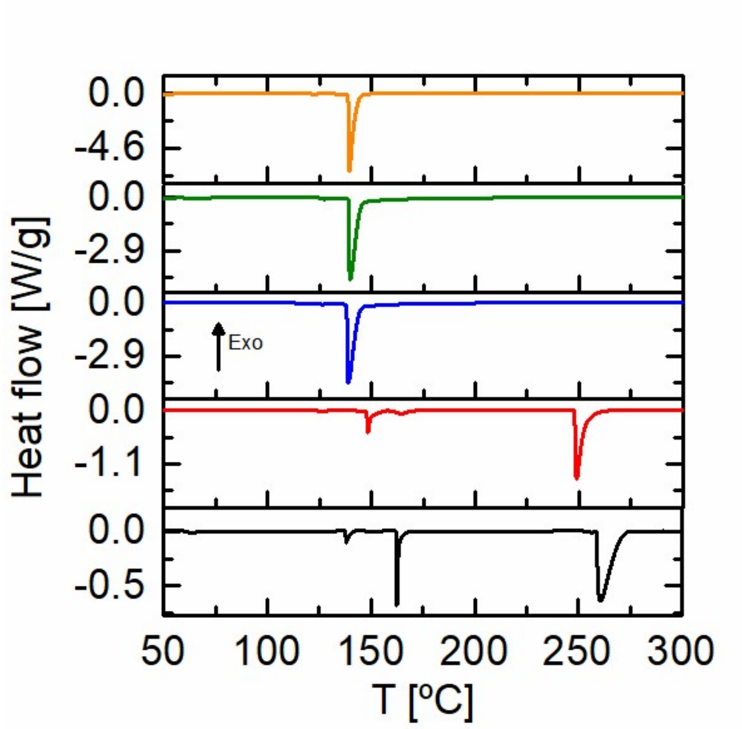

Figure S11. DSC analysis of Zr-porphyrinic MOFs for L/M = 0.25 (black), 0.50 (red), 1.00 (blue), 1.50 (gree) and 2.00 (orange).

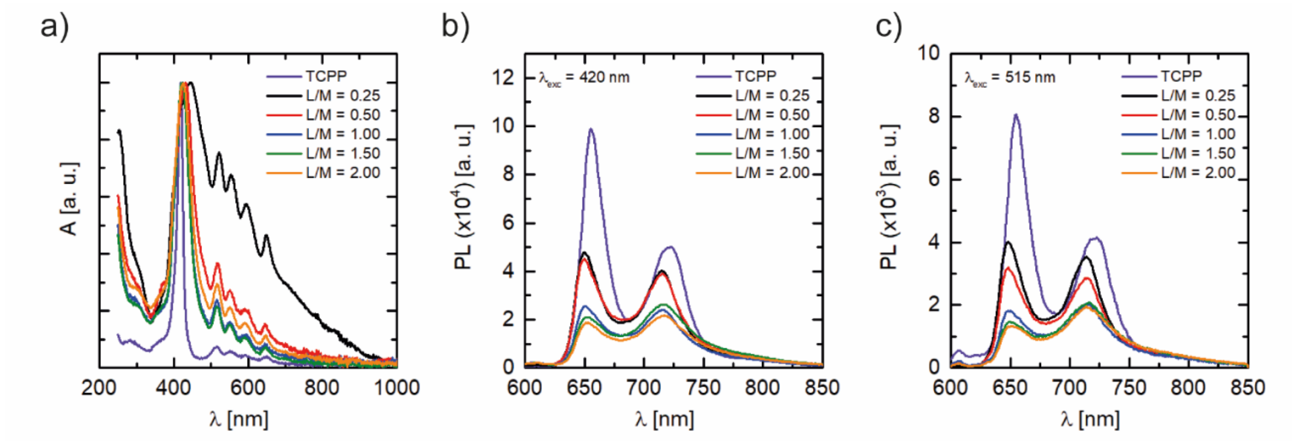

Figure S12. a) Normalized absorption spectra and photoluminescence spectra at b) 420nm and c) 515 nm excitation wavelength of Zr-porphyrinic MOFs.

EtO (acetic acid) role of the temperature in the reaction yield

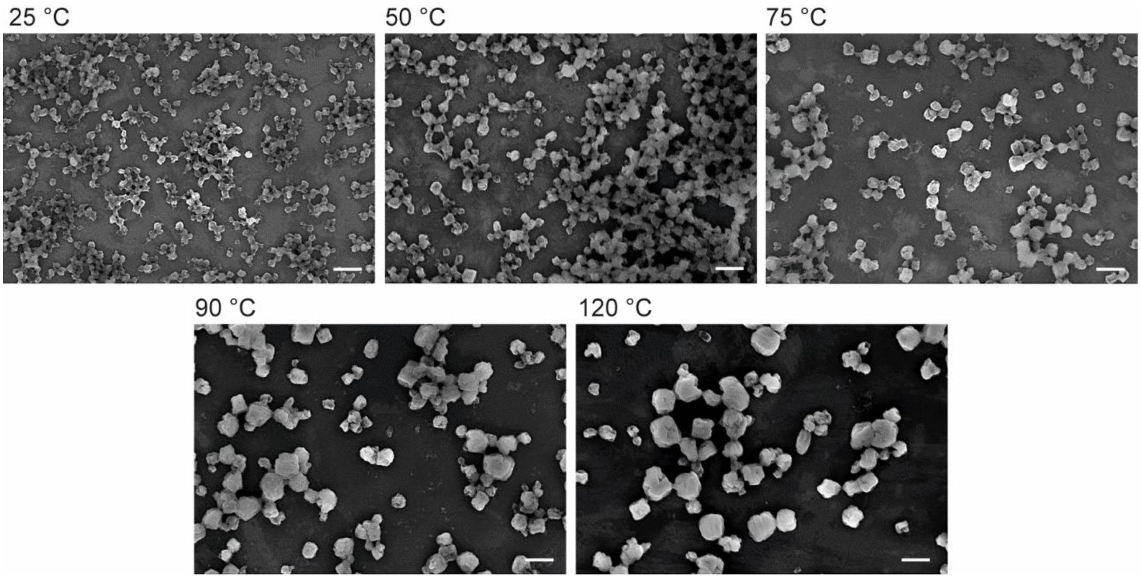

Figure S13. Representative FE-SEM images for 1 h reaction performed at different temperatures (25 °C, 50 °C, 75 °C, 90 °C and 120 °C). The ratios adopted for the synthesis of the particles are L/M = 0.25, Mod/M = 250; the Zr precursor used is $Zr(OEt)_4$ and the modulator is AA. Scale bars: 200 nm.

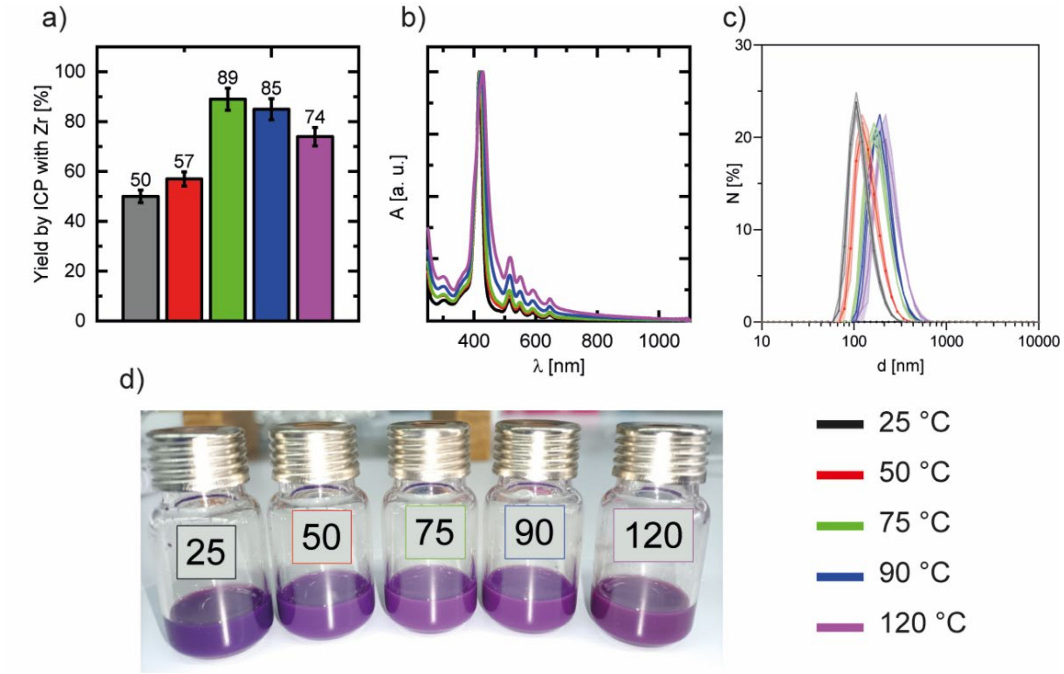

Figure S14. a) Graphical representation of the reaction yield evaluated through ICP-OES (see Table S1 for more details), b) UV-Vis extinction spectra, c) hydrodynamic diameters (by number) and d) digital images of 1h reaction conducted at different temperatures.

Table S9. Zr quantification through ICP-OES to determine the reaction yield at different temperatures (25 °C, 50 °C, 75 °C, 90 °C and 120 °C) of 1 h reaction. The reaction yield (Yield%) is calculated from the relation between the amount of Zr at the beginning of the reaction (Zr (precursor)) and the amount of Zr in the final particles after purification. Each measurement is performed in triplicate and the Zr value is the average value of two Zr emission lines (339.198 nm and 343.823 nm, respectively). The ratios adopted for the synthesis of the particles are L/M = 0.25, Mod/M = 250; the Zr precursor used is $Zr(OEt)_4$ and the modulator is AA.

Entry	Zr average	Yield%
Zr (precursor)	2.05 ± 0.10	/
25 °C	1.02 ± 0.05	49.9 ± 2.5
50 °C	1.17 ± 0,06	57.1 ± 2.8
75 °C	1.83 ± 0.09	88.9 ± 4.4
90 °C	1.74 ± 0.09	85.0 ± 4.2
120 °C	1.52 ± 0.08	74.2 ± 3.7

Table S10. Hydrodynamic diameter by intensity ($d_{h,I}$) and by number ($d_{h,n}$) measured through DLS of the sample synthesized at different temperatures (1 h reaction). The Polydispersity Index (PdI) is also reported.

Entry	$d_{h,I}$ (nm)	$d_{h,n}$ (nm)	PdI
25 °C	163.4 ± 3.6	121.3 ± 5.2	0.190 ± 0.028
50 °C	195.6 ± 3.7	144.9 ± 5.9	0.137 ± 0.013
75 °C	233.0 ± 5.4	184.4 ± 2.2	0.221 ± 0.009
90 °C	250.9 ± 6.5	200.8 ± 4.7	0.093 ± 0.022
120 °C	302.1 ± 12.8	237.5 ± 5.5	0.089 ± 0.027

Alkoxy (acetic acid) 75 °C (1 h): L/M = 0.25 Mod/M = 250

In this section, the results relative to the reaction performed at 75 °C (1 h) in presence of different Zr precursors ($Zr(OEt)_4$, $Zr(OiPr)_4$ and $Zr(OBut)_4$) and with the use of AA as modulator are reported. The ratios adopted for the synthesis of the particles are L/M = 0.25, Mod/M = 250.

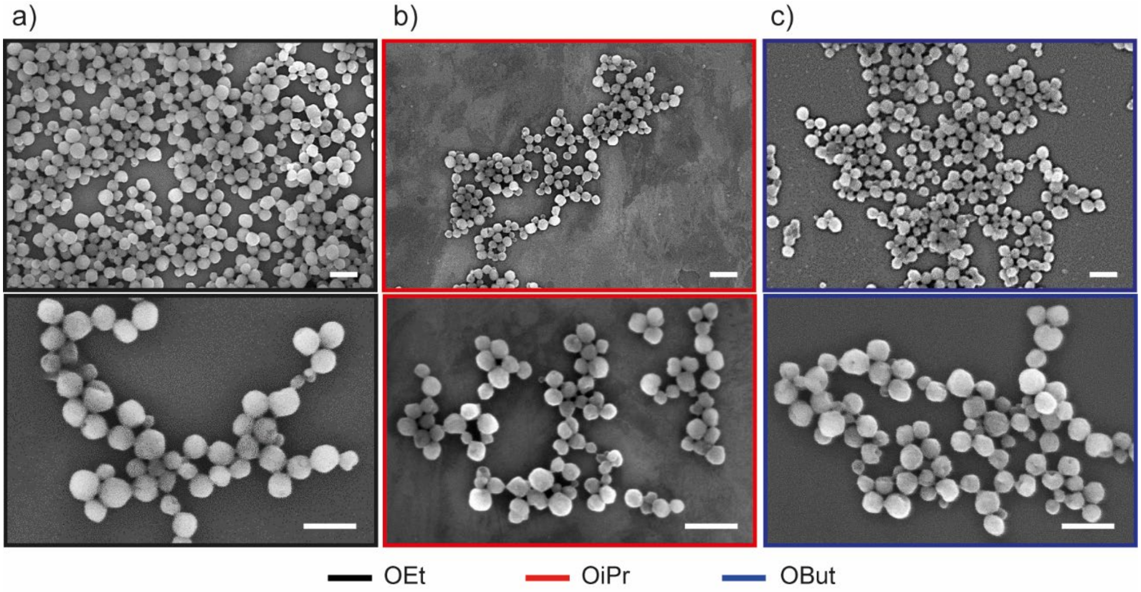

Figure S15. Representative FE-SEM images for 1 h reaction performed at 75 °C by using different Zr precursors and AA as modulator. Scale bars: 200 nm.

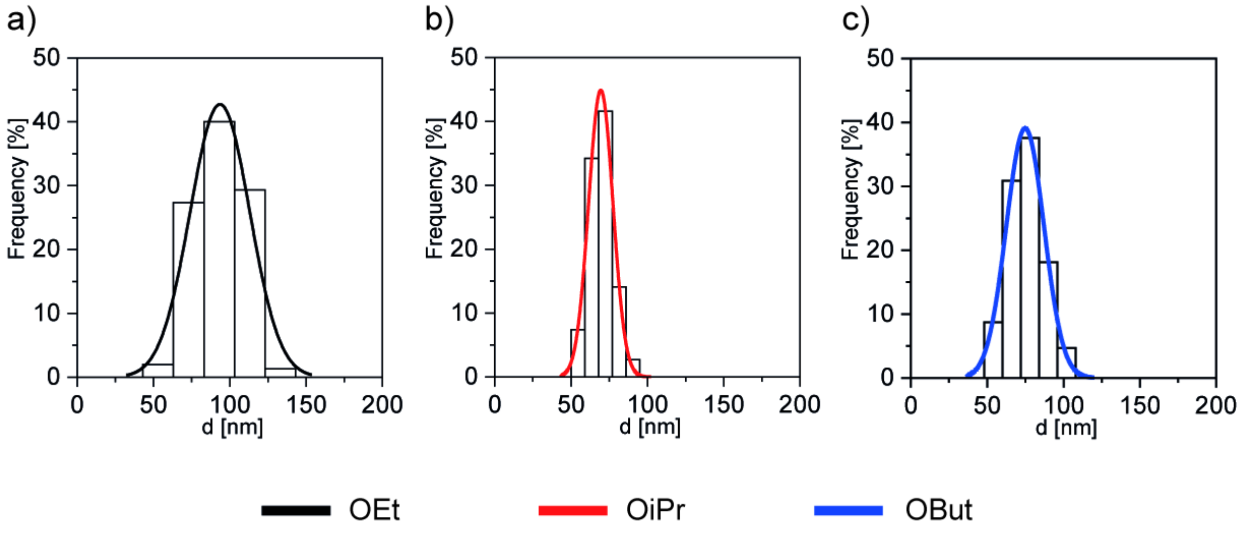

Figure S16. Particle size distribution histogram relative to SEM images of 1 h reaction performed at 75 °C by using different Zr precursors and AA as modulator.

Table S11. Particle size distribution measured through SEM analysis of the samples synthesized with different Zr precursors.

Precursor	Modulator	Time	Temperature	d_{SEM} (nm)
$Zr(OEt)_4$	AA	1 h	75 °C	92.7 ± 17.3
$Zr(OiPr)_4$				69.8 ± 7.6
$Zr(OBut)_4$				75.5 ± 11.5

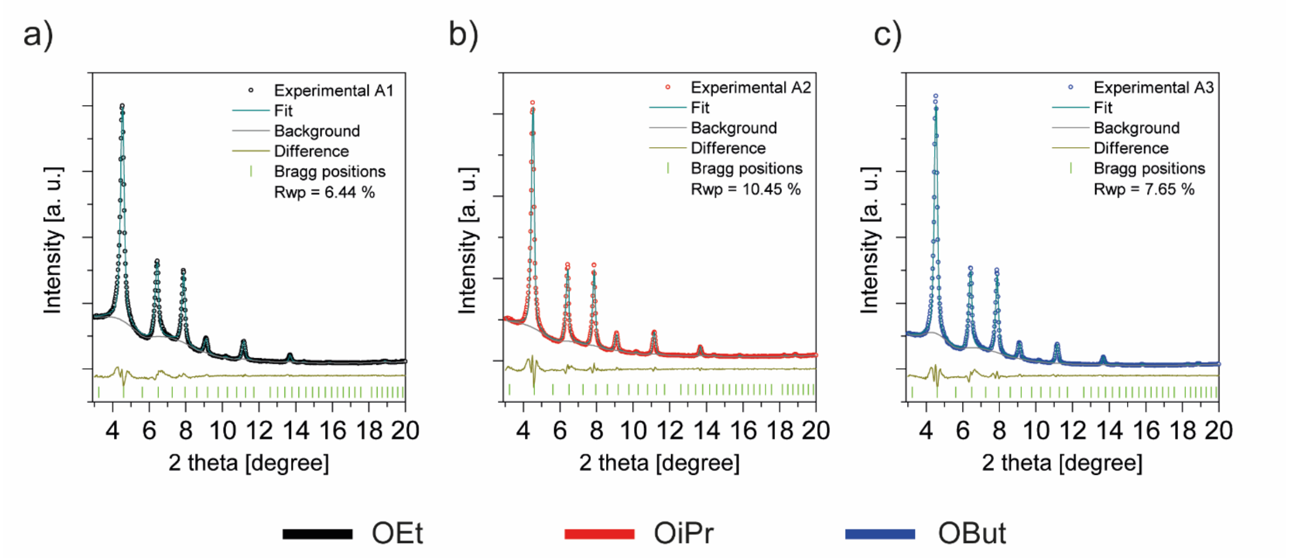

Figure S17. Pawley refinement of Zr-porphyrinic MOF nanoparticles, synthesized at 75 °C, 1 h reaction, by using different Zr precursors and AA as modulator.

Table S12. BET surface area and pore volume of the particles synthesized with different Zr precursors.

Precursor	BET surface area (m^2/g)	t-plot micropore volume (cm^3/g)
$Zr(OEt)_4$	2467	0.718
$Zr(OiPr)_4$	3083	0.904
$Zr(OBut)_4$	2629	0.845

Table S13. Zr quantification through ICP-OES to determine the reaction yield of the reaction performed with different Zr precursors.

Precursor	Modulator	Time	Temperature	Yield%
$Zr(OEt)_4$	AA	1 h	75 °C	88.9 ± 4.4
$Zr(OiPr)_4$				71.2 ± 3.6
$Zr(OBut)_4$				85.2 ± 4.2

Table S14. Hydrodynamic diameter by intensity ($d_{h,I}$) and by number ($d_{h,n}$) and the Polydispersity Index (PdI) measured through DLS of the samples synthesized with different Zr precursors at 75 ºC foy 1h. The Polydispersity Index is also reported (PdI).

Precursor	$d_{h,I}$ (nm)	$d_{h,n}$ (nm)	PdI
$Zr(OEt)_4$	176.6 ± 3.4	153.9 ± 1.8	0.026 ± 0.005
$Zr(OiPr)_4$	241.2 ± 17.0	158.1 ± 13.6	0.155 ± 0.041
$Zr(OBut)_4$	189.2 ± 2.2	158.2 ± 1.1	0.021 ± 0.018

Alkoxy (acetic acid) 25 °C (1 h vs 24 h): L/M = 0.25 Mod/M = 250

In this section, the results relative to the reaction performed at 25 °C in presence of different Zr precursors ($Zr(OEt)_4$, $Zr(OiPr)_4$ and $Zr(OBut)_4$) and with the use of AA as modulator are reported. The ratios adopted for the synthesis of the particles are L/M = 0.25, Mod/M = 250.

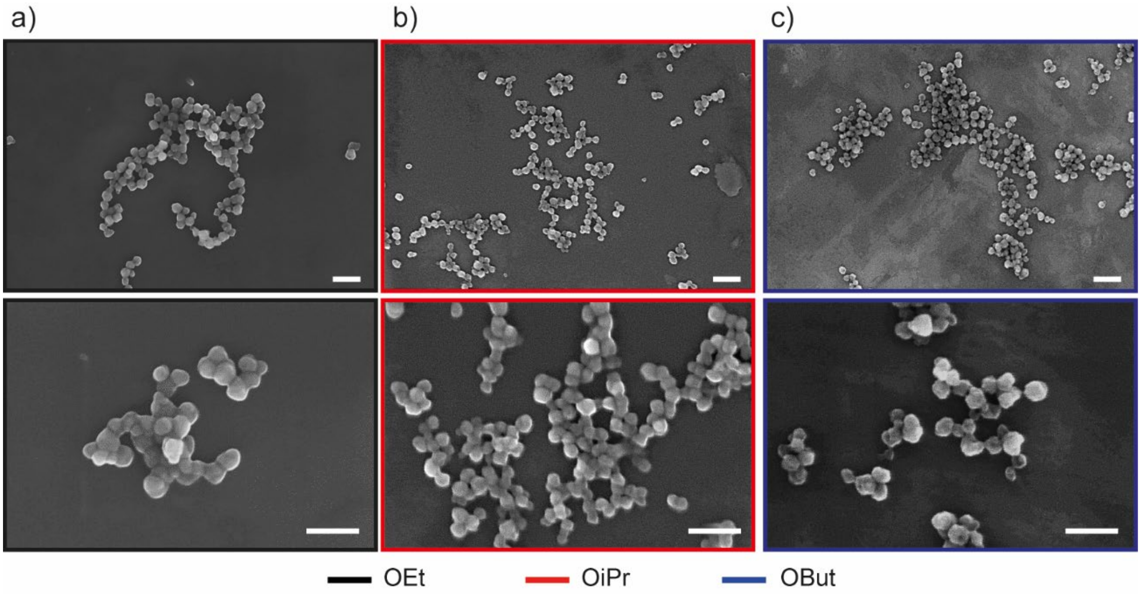

Figure S18. Representative FE-SEM images for 1 h reaction performed at RT by using different Zr precursors and AA as modulator. Scale bars: 200 nm.

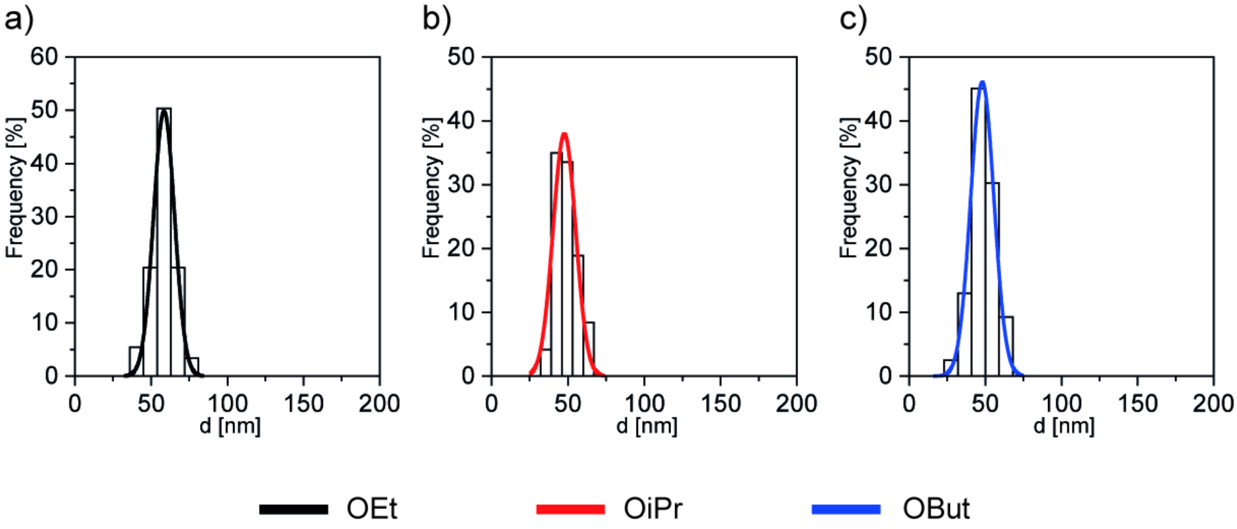

Figure S19. Particle size distribution histogram relative to SEM images of 1 h reaction performed at RT by using different Zr precursors and AA as modulator.

Table S15. Particle size distribution measured through SEM analysis of the samples synthesized with different Zr precursors.

Precursor	Modulator	Time	Temperature	d_{SEM} (nm)
$Zr(OEt)_4$	AA	1 h	25 °C	58.2 ± 7.7
$Zr(OiPr)_4$				49.1 ± 6.8
$Zr(OBut)_4$				48.2 ± 8.3

Table S16. Zr quantification through ICP-OES to determine the reaction yield of the reaction performed with different Zr precursors after 1h and 24h of reaction.

Precursor	Modulator	Temperature	Time	Yield%
$Zr(OEt)_4$	AA	25 °C	1 h	49.9 ± 2.5
$Zr(OiPr)_4$				43.0 ± 2.2
$Zr(OBut)_4$				55.3 ± 2.8
$Zr(OEt)_4$			24 h	68.5 ± 3.4
$Zr(OiPr)_4$				69.8 ± 3.5
$Zr(OBut)_4$				78.1 ± 3.9

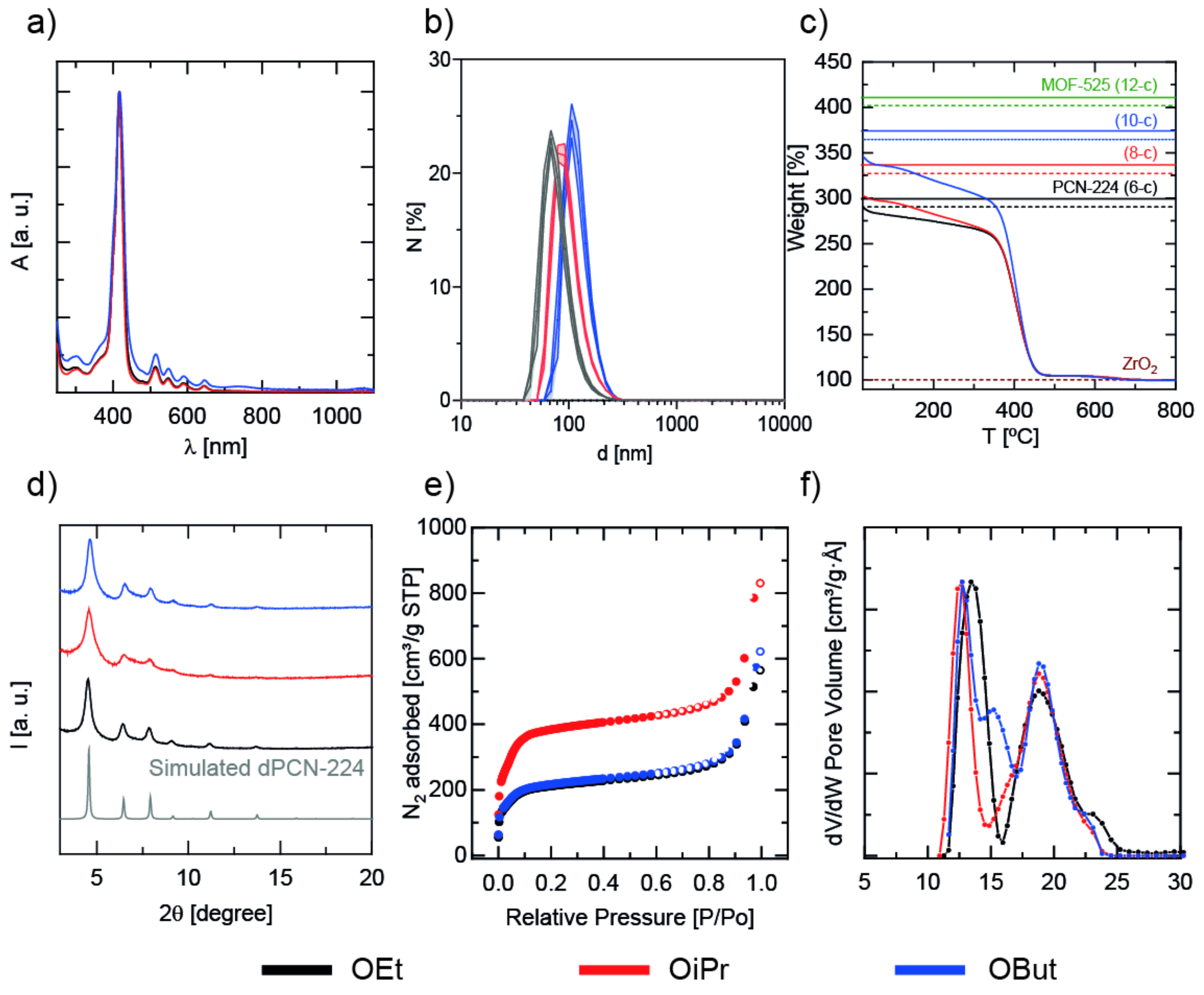

Figure S20. a) UV-Vis extinction spectra, b) hydrodynamic diameters (by number), c) thermogravimetric analysis (TGA), d) powder X-rays diffraction (PXRD) patterns, e) BET analysis and f) NLDFT pore size distribution of 1 h reaction at RT by using different Zr precursors.

Table S17. Hydrodynamic diameter by intensity ($D_{h,I}$) and by number ($D_{h,n}$) and the Polydispersity Index (PdI) measured through DLS of the samples synthesized with different Zr precursors for 1 h at RT. The Polydispersity Index is also reported (PdI).

Precursor	Time	$D_{h,I}$ (nm)	$D_{h,n}$ (nm)	PdI
$Zr(OEt)_4$	1 h	141.6 ± 1.8	79.5 ± 3.0	0.206 ± 0.003
$Zr(OiPr)_4$		156.6 ± 1.5	99.2 ± 1.8	0.102 ± 0.018
$Zr(OBut)_4$		153.5 ± 4.3	119.8 ± 39.3	0.060 ± 0.011

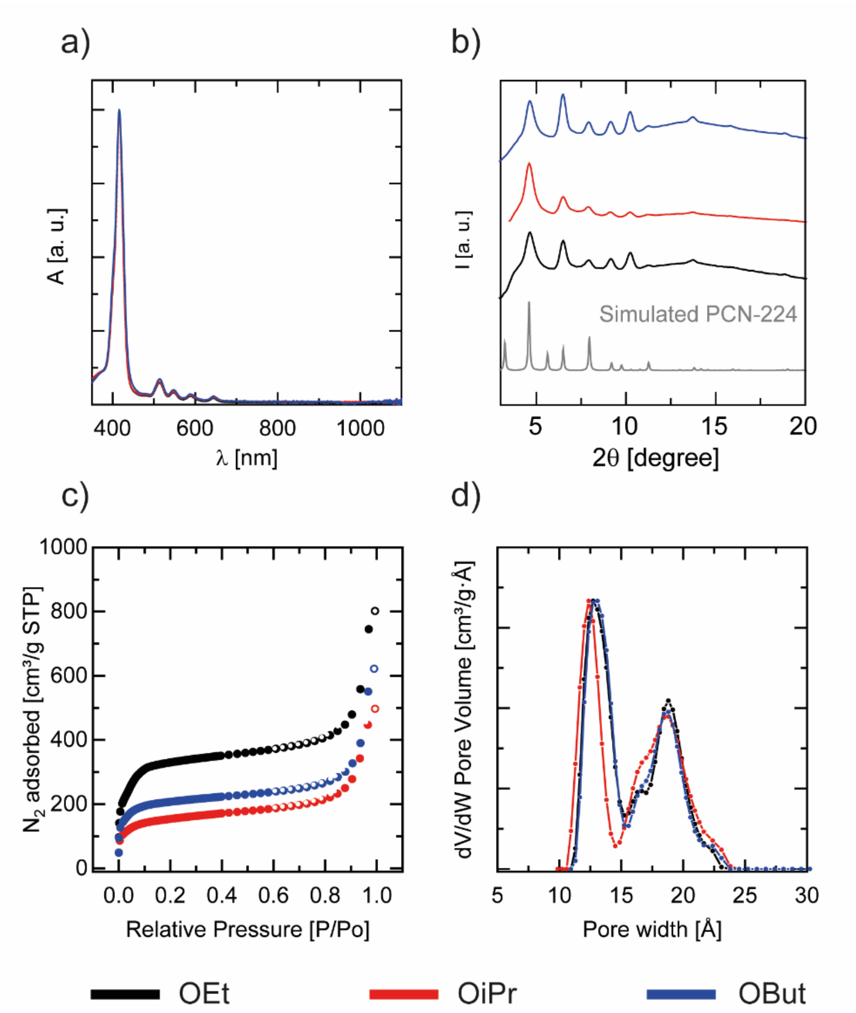

Figure S21. a) UV-Vis extinction spectra, b) PXRD patterns, c) BET analysis and d) NLDFT pore size distribution of 24 h reaction at RT by using different Zr precursors.

Table S18. BET surface area and pore volume of the particles synthesized with different Zr precursors.

**Precursor**	**Time**	**BET surface area (m^2/g)**	**t-plot micropore volume (cm^3/g)**
$Zr(OEt)_4$	1 h	816	0.218
$Zr(OiPr)_4$		1561	0.444
$Zr(OBut)_4$		833	0.206
$Zr(OEt)_4$	24 h	1347	0.363
$Zr(OiPr)_4$		580	0.132
$Zr(OBut)_4$		792	0.223

Alkoxy (formic acid) 75 °C

L/M = 0.35, Mod/M = 100; 24h; 75 °C

In this section, the results relative to the reaction performed at 75 °C (24 h) in presence of different Zr precursors ($Zr(OEt)_4$, $Zr(OiPr)_4$ and $Zr(OBut)_4$) and with the use of formic acid as modulator are reported. The ratios adopted for the synthesis of the particles are L/M = 0.35, Mod/M = 100.

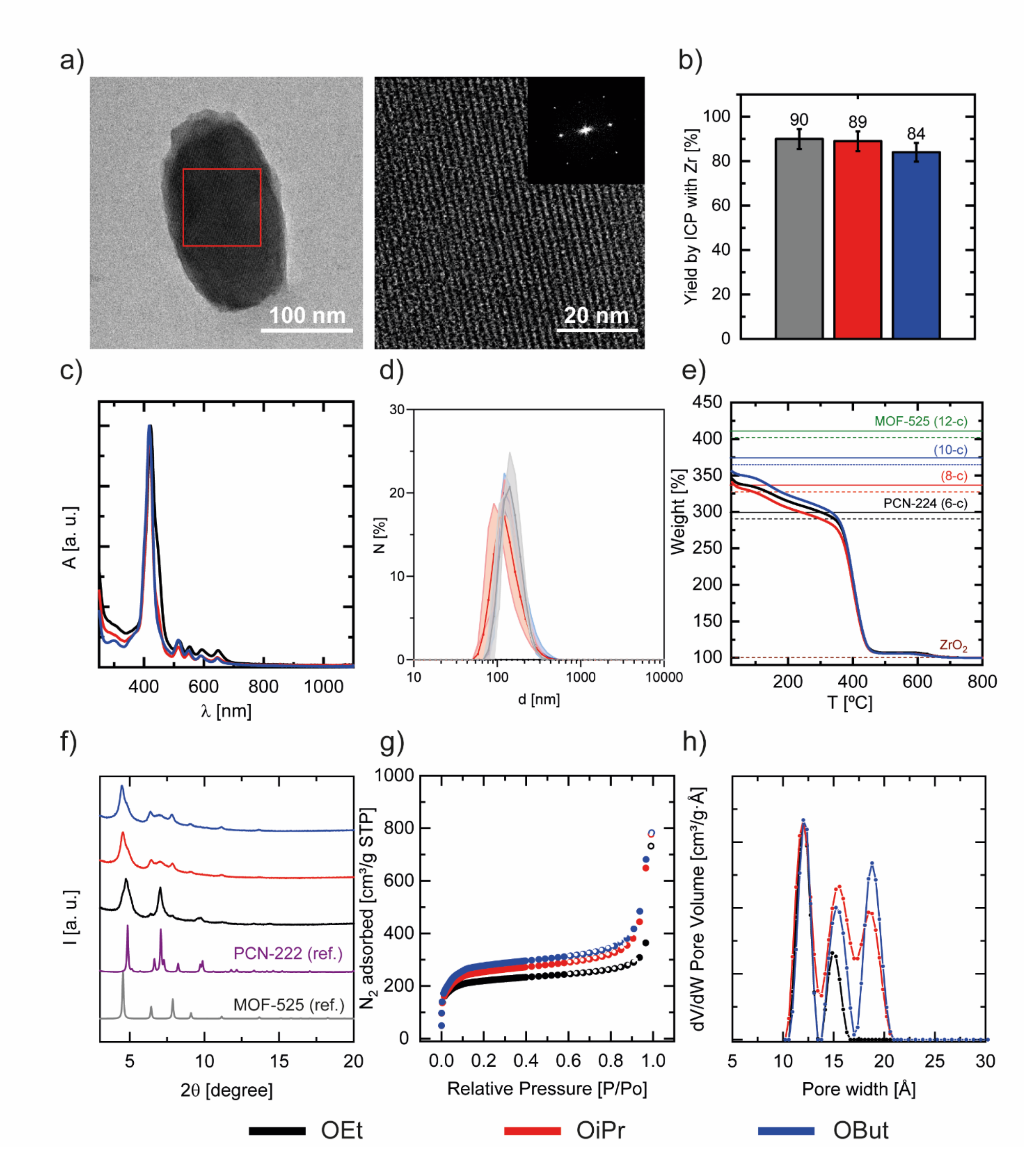

Figure S22. a) HRTEM images of nanoMOFs synthesized starting from $Zr(OEt)_4$, b) graphical representation of the reaction yields evaluated through ICP-OES of 1h-reaction

conducted at 75 °C for 24h c) UV-Vis extinction spectra, d) hydrodynamic diameters (by number), e) thermogravimetric analysis (TGA), f) PXRD patterns, g) N_2 adsorption isotherms at 77K and h) NLDFT pore size distribution.

Table S19. Zr quantification through ICP-OES to determine the reaction yield of the reaction performed with different Zr precursors.

Precursor	Modulator	Time	Temperature	Yield%
$Zr(OEt)_4$	Formic acid	24 h	75 °C	89.7 ± 4.5
$Zr(OiPr)_4$				88.6 ± 4.4
$Zr(OBut)_4$				84.5 ± 4.2

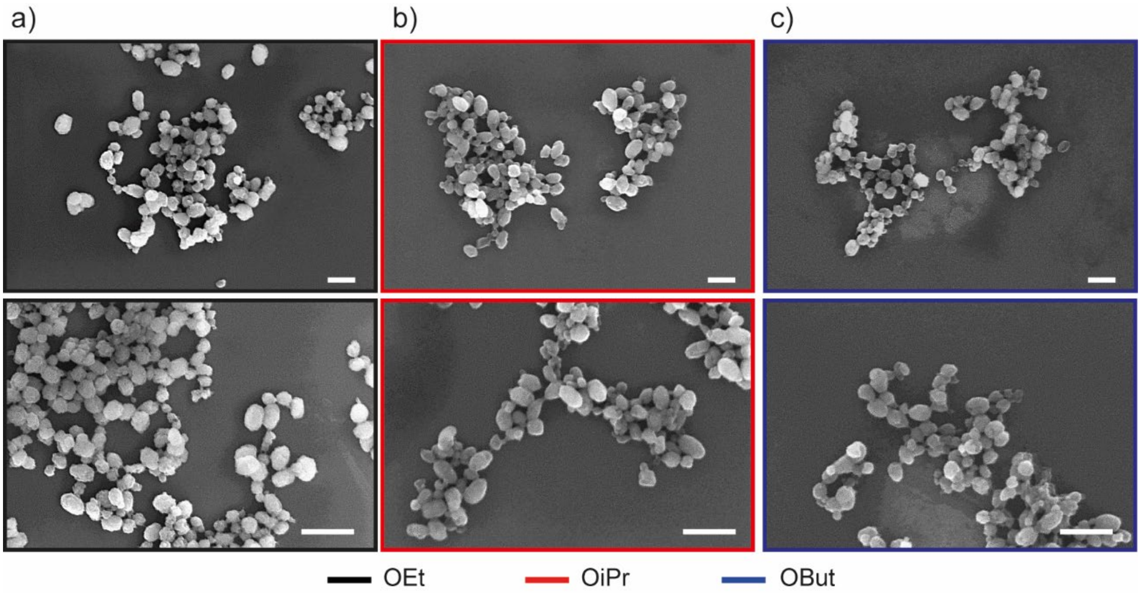

Figure S23. Representative FE-SEM images for 24 h reaction performed at 75 °C by using different Zr precursors and formic acid as modulator. Scale bars: 200 nm.

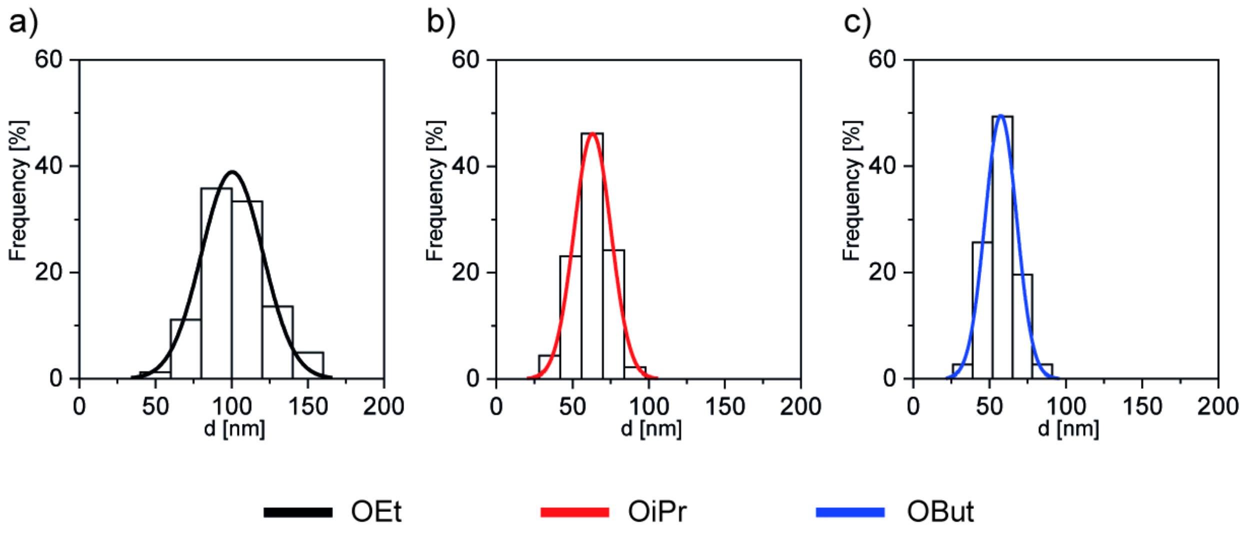

Figure S24. Particle size distribution histogram relative to SEM images of 24 h reaction performed at 75 °C by using different Zr precursors and formic acid as modulator.

Table S20. Particle size distribution measured through SEM analysis of the samples synthesized with different Zr precursors.

Precursor	Modulator	Time	Temperature	d_{SEM} (nm)
$Zr(OEt)_4$	Formic acid	24 h	75 °C	102.8 ± 20.1
$Zr(OiPr)_4$				62.8 ± 12.1
$Zr(OBut)_4$				57.2 ± 10.0

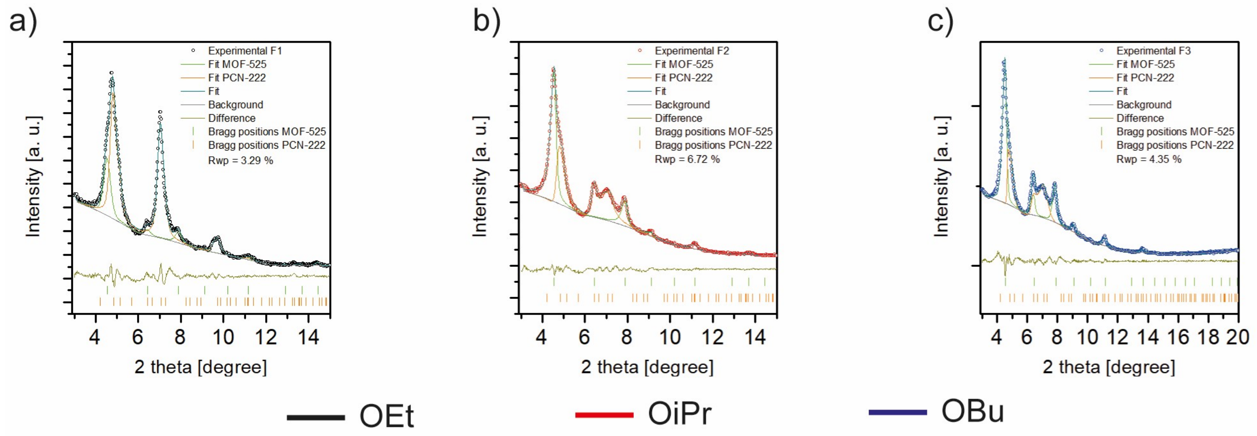

Figure S25. Pawley refinement of Zr-porphyrinic MOF nanoparticles, synthesized at 75 °C, 24 h reaction, by using different Zr precursors and formic acid as modulator.

Table S21. Hydrodynamic diameter by intensity ($D_{h,I}$) and by number ($D_{h,n}$) and the Polydispersity Index (PdI) measured through DLS of the samples synthesized with different Zr precursors. The Polydispersity Index is also reported (PdI).

Precursor	Time	$D_{h,I}$ (nm)	$D_{h,n}$ (nm)	PdI
$Zr(OEt)_4$	24 h	191.5 ± 4.4	151.7 ± 11.3	0.038 ± 0.032
$Zr(OiPr)_4$		214.2 ± 5.1	136.1 ± 19.1	0.174 ± 0.053
$Zr(OBut)_4$		215.6 ± 34.6	154.0 ± 11.9	0.135 ± 0.065

Table S22. BET surface area and pore volume of the particles synthesized with different Zr precursors.

Precursor	BET surface area (m^2/g)	t-plot micropore volume (cm^3/g)
$Zr(OEt)_4$	840	0.250
$Zr(OiPr)_4$	981	0.275
$Zr(OBut)_4$	1068	0.315

L/M = 0.35-1.5, Mod/M = 250 or 560; 1h; 75 °C

In this section, the results shown are relative to the reaction performed at 75 °C (1 h) with $Zr(OEt)_4$ as a precursor and formic acid as modulator. The ratios adopted for the synthesis were L/M = 0.35-1.5 and Mod/M = 250 or 560.

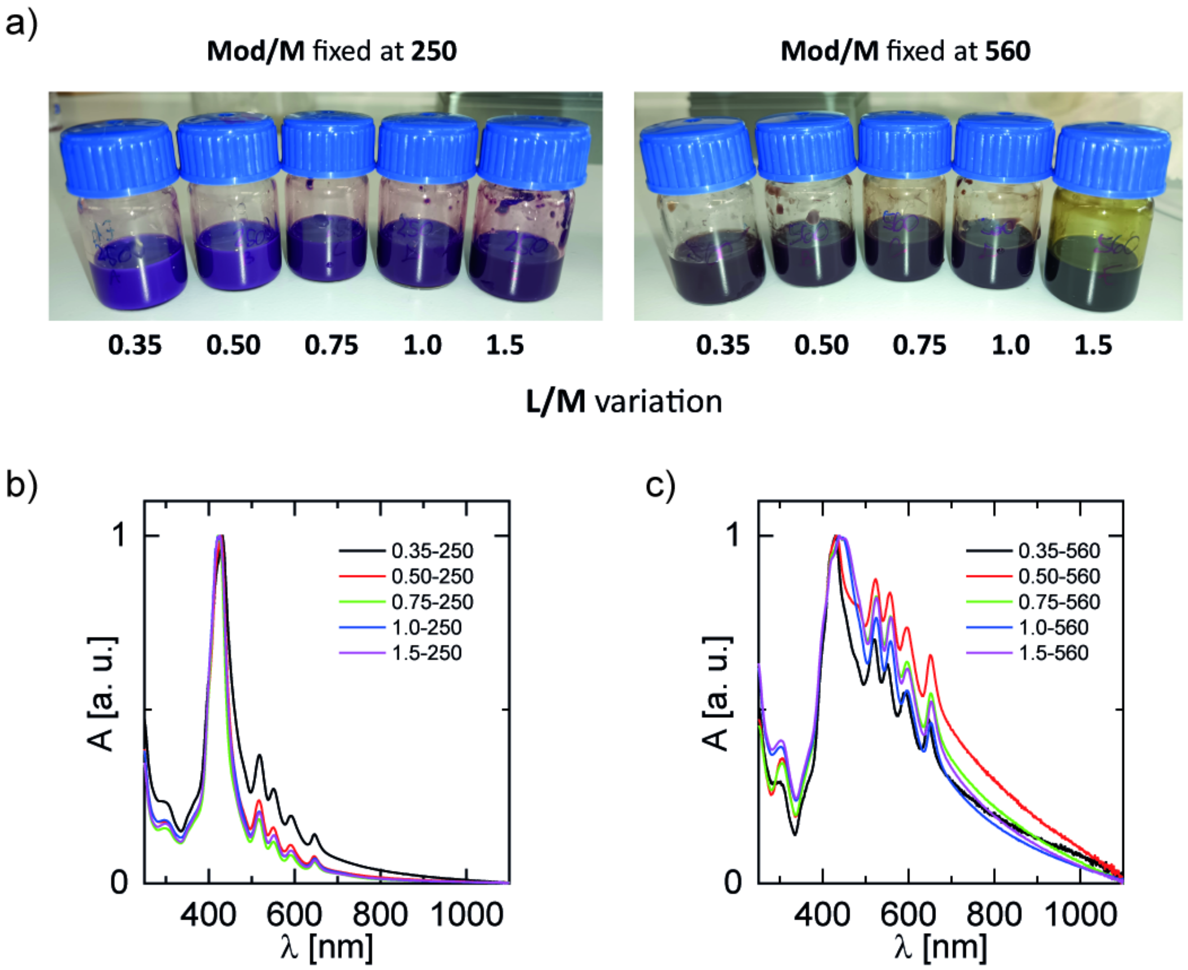

Figure S26. a) Digital images of 1h reaction conducted at 75 °C with FA and $Zr(OEt)_4$, by varying the L/M and Mod/M ratios. UV-Vis extinction spectra of b) Mod/M = 250 and c) Mod/M = 560.

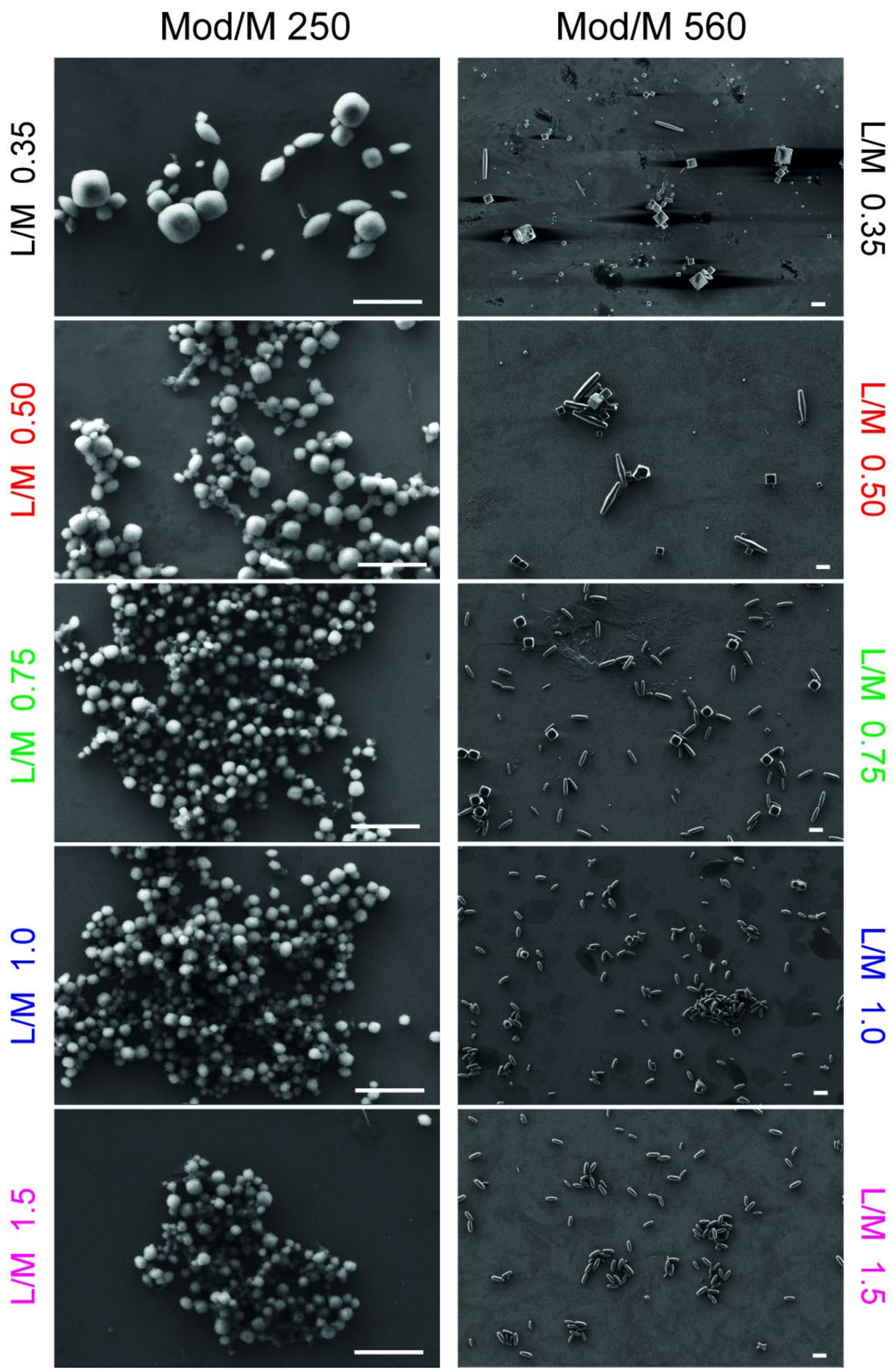

Figure S27. Representative FE-SEM images of the L/M and Mod/M modulations for Zr-porphyrinic MOF nanoparticles synthesis performed at 75 °C, 1h-reaction, with the use of FA as modulator and $Zr(OEt)_4$ as precursor. Mod/M 250 scale bars: 1 μm. Mod/M 560 scale bars: 2 μm.

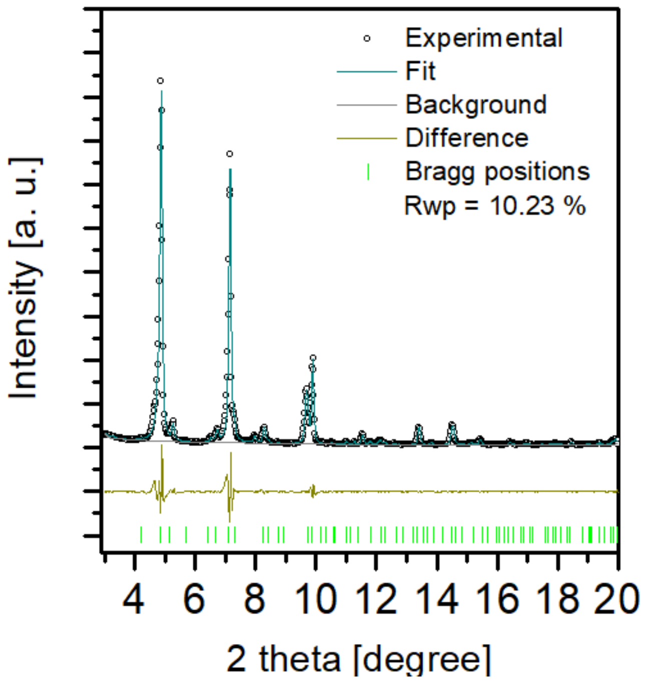

Figure S28. Pawley refinement of Zr-porphyrinic MOF nanoparticles, synthesized at 75 °C, 1 h reaction, by using $Zr(OEt)_4$ as precursor and FA as modulator. The ratios adopted were L/M = 1.5 and Mod/M = 560.

Reproducibility test

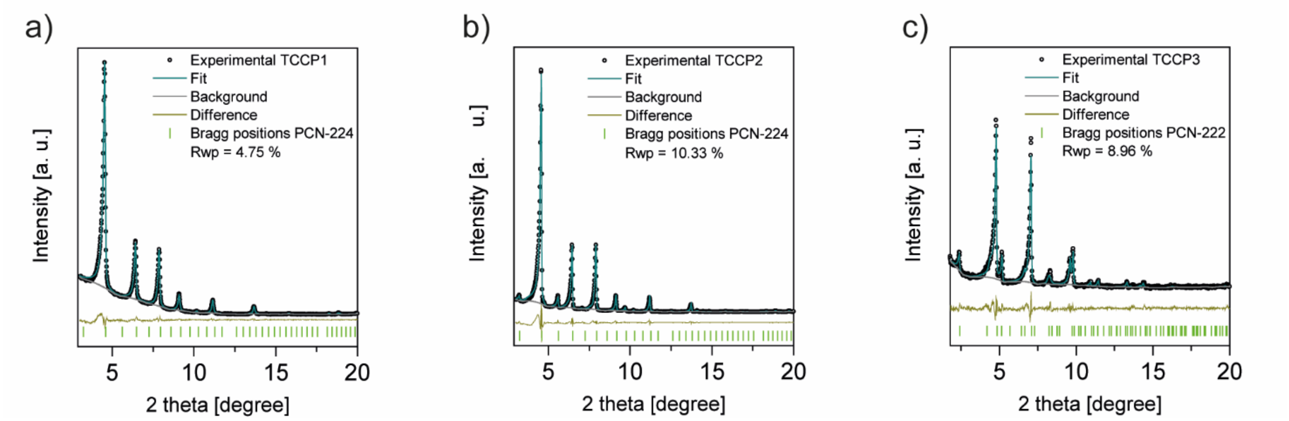

Figure S29. Reproducibility test for the synthesis of PCN-224 following synthetic conditions of a) Table S1 and b) Table S2 and for the synthesis of PCN-222 following the synthetic conditions of Table S3, using $Zr(OEt)_4$ as precursor.

Continuous flow reaction

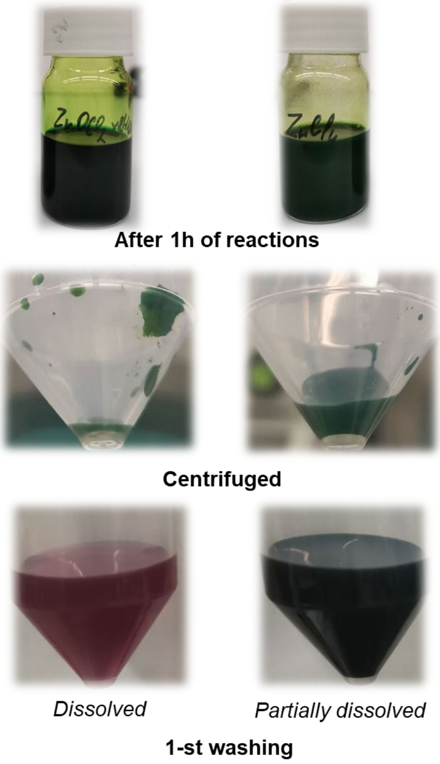

Figure S30. Photo of the reactions using $ZrCl_4$ and $ZrOCl_2$ as metal precursors at room temperature, 1h.

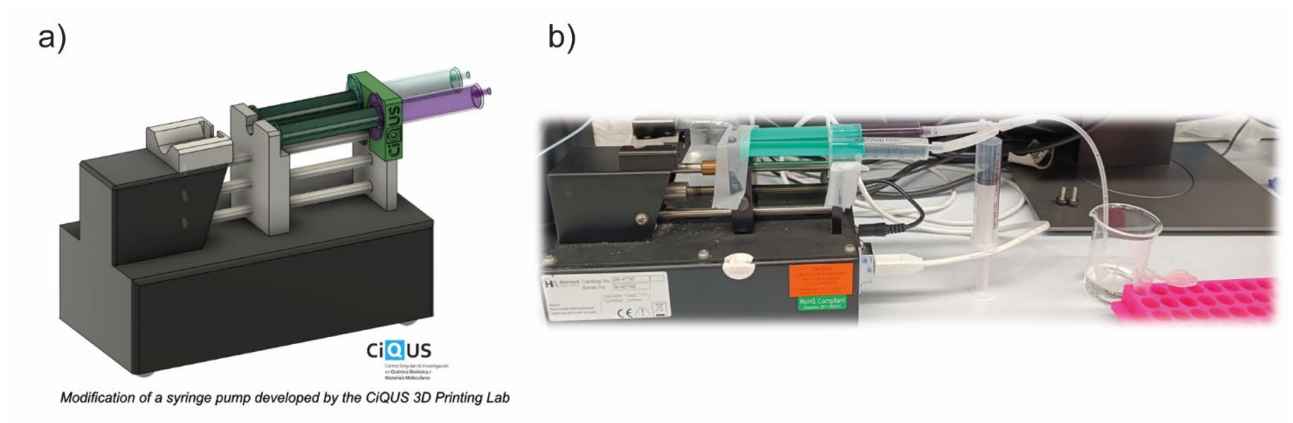

Figure S31. a) Pump infusion prototype and b) photo of continuous flow setup using an infusion pump with two syringes.

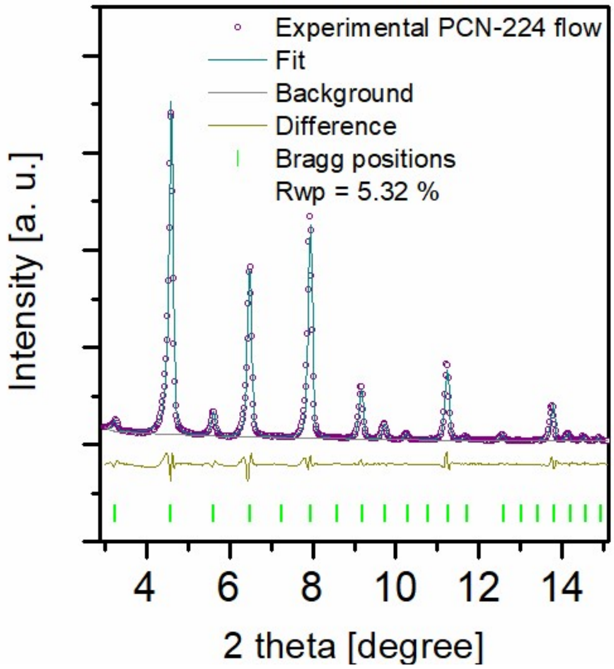

Figure S32. Pawley refinement of PCN-224 synthesized by continuous flow reaction.

APPENDIX BETSI N_2 adsorption analysis

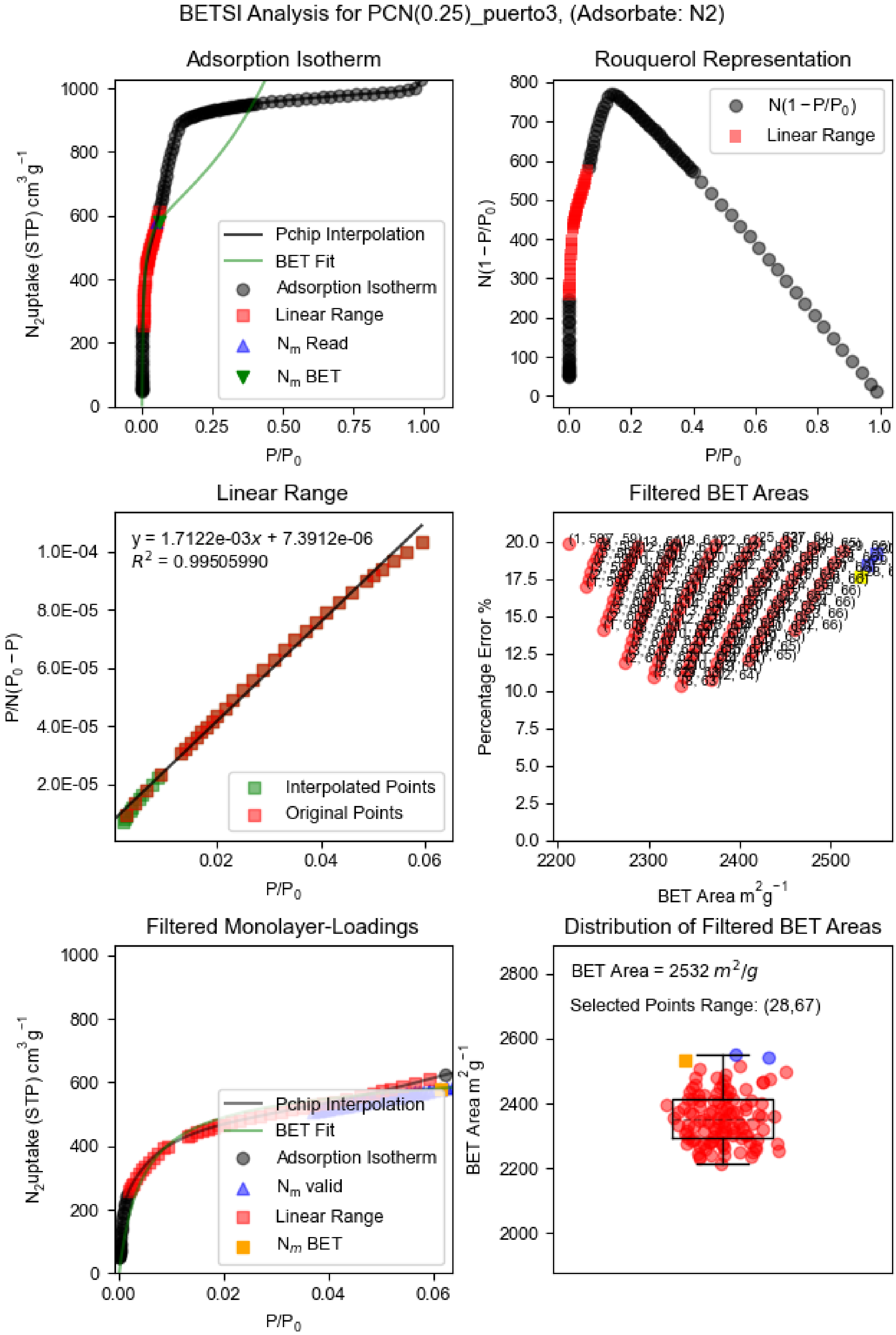

Figure S33. BETSI analysis of Zr-porphyrinic MOF synthesized with L/M 0.25, Mod(AA)/M 560, RT, 1 h of reaction and $Zr(OEt)_4$ as precursor.

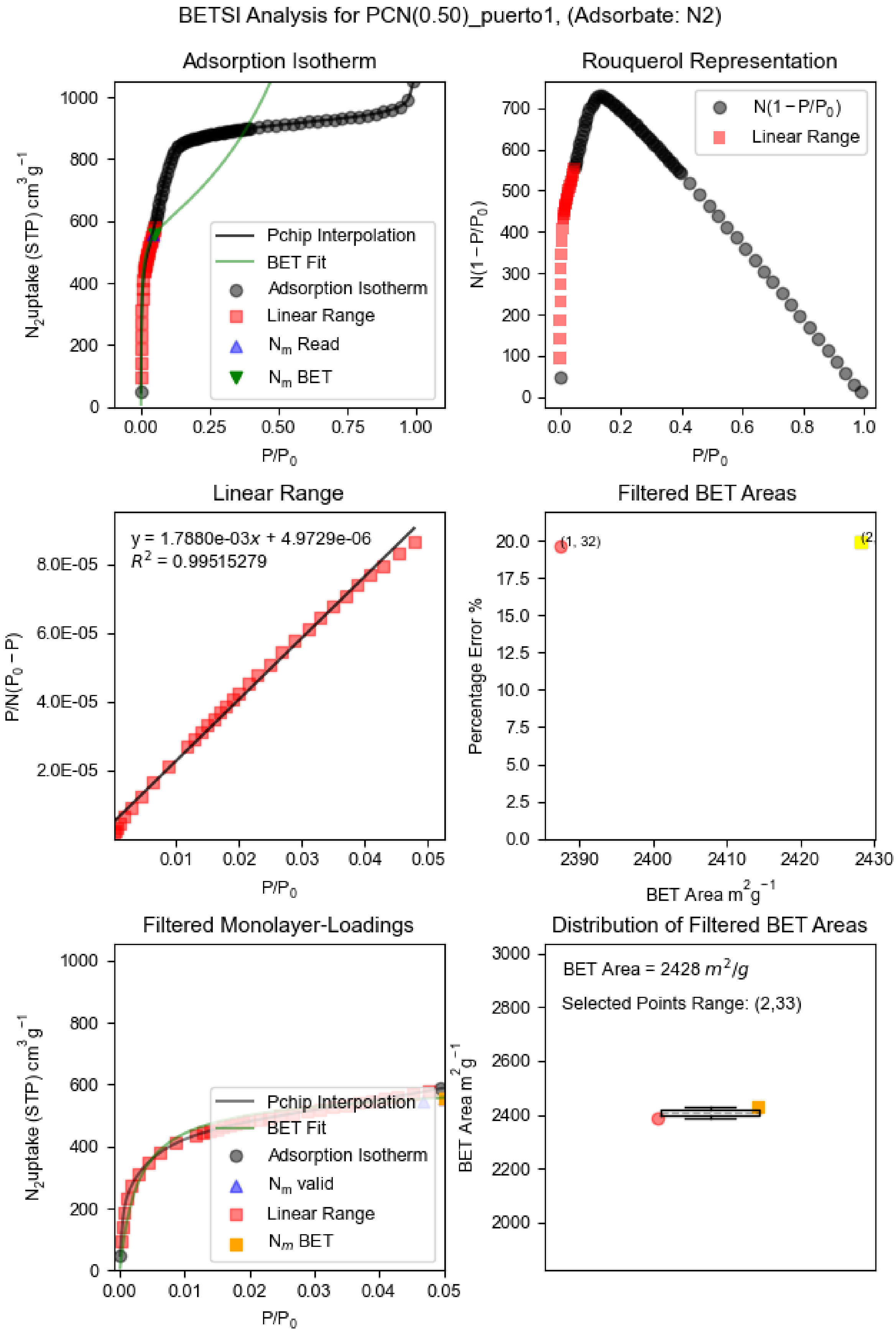

Figure S34. BETSI analysis of Zr-porphyrinic MOF synthesized with L/M 0.50, Mod(AA)/M 560, RT, 1h of reaction and $Zr(OEt)_4$ as precursor.

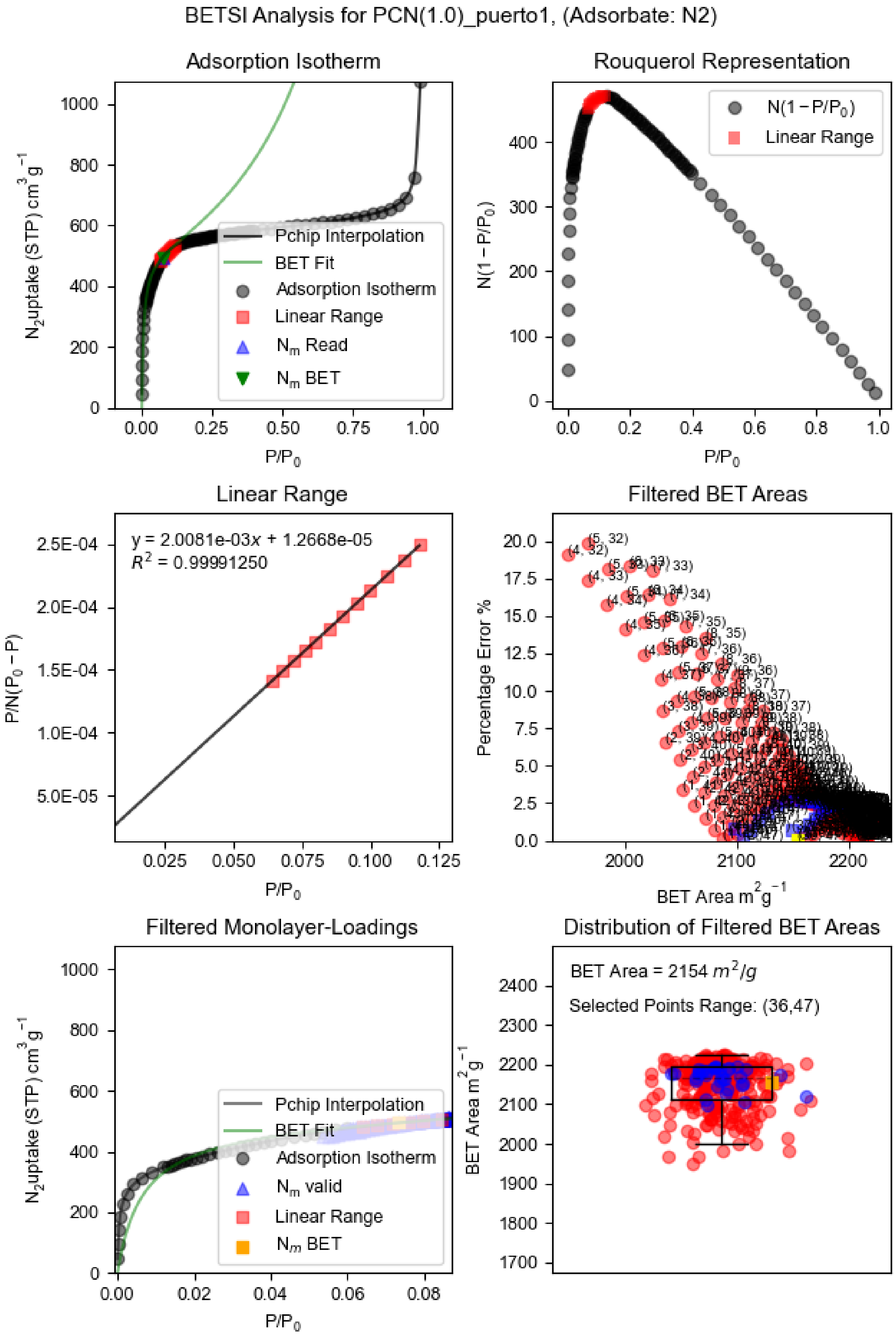

Figure S35. BETSI analysis of Zr-porphyrinic MOF synthesized with L/M 1.00, Mod(AA)/M 560, RT, 1h of reaction and $Zr(OEt)_4$ as precursor.

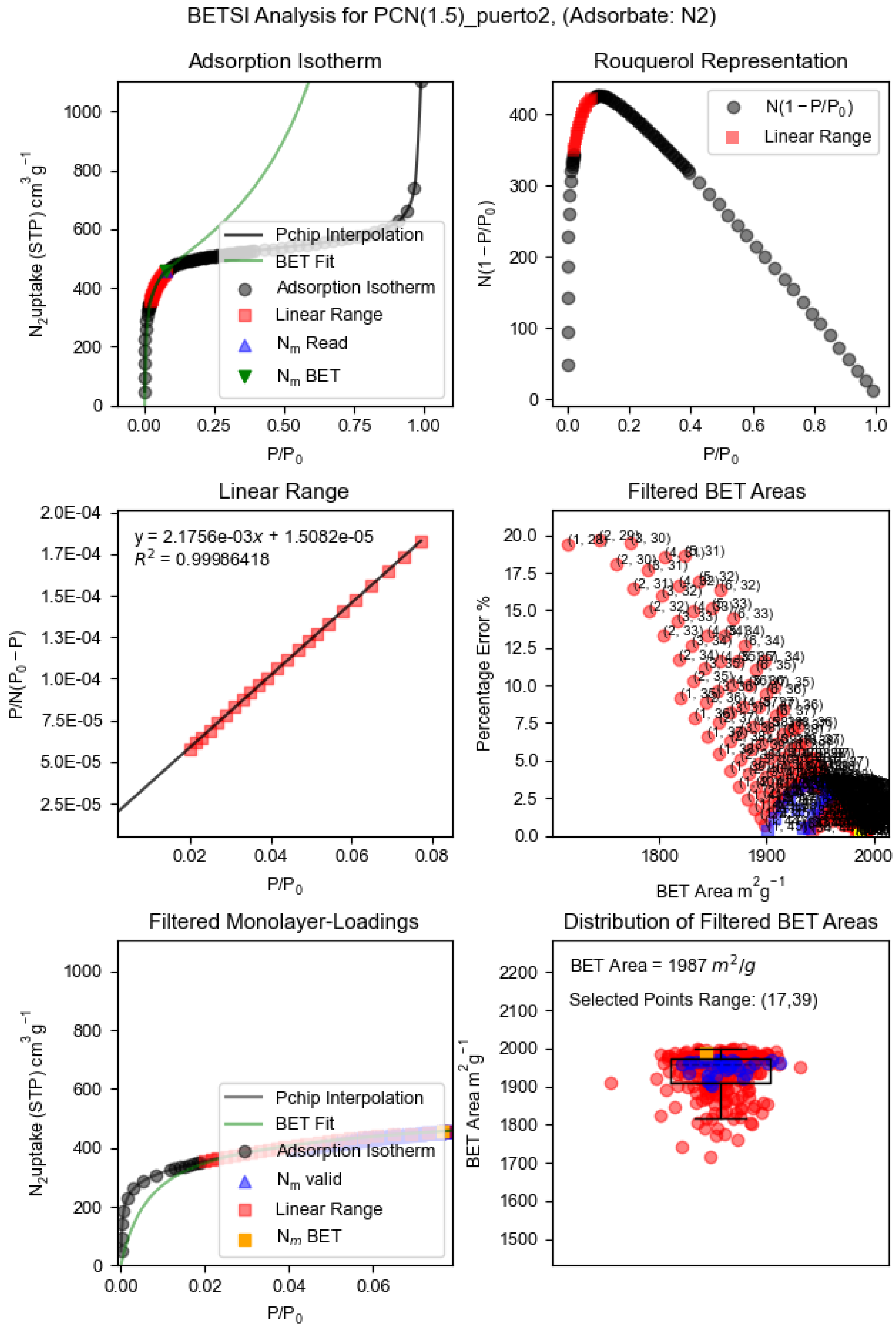

Figure S36. BETSI analysis of Zr-porphyrinic MOF synthesized with L/M 1.50, Mod(AA)/M 560, RT, 1h of reaction and Zr(OEt)4 as precursor.

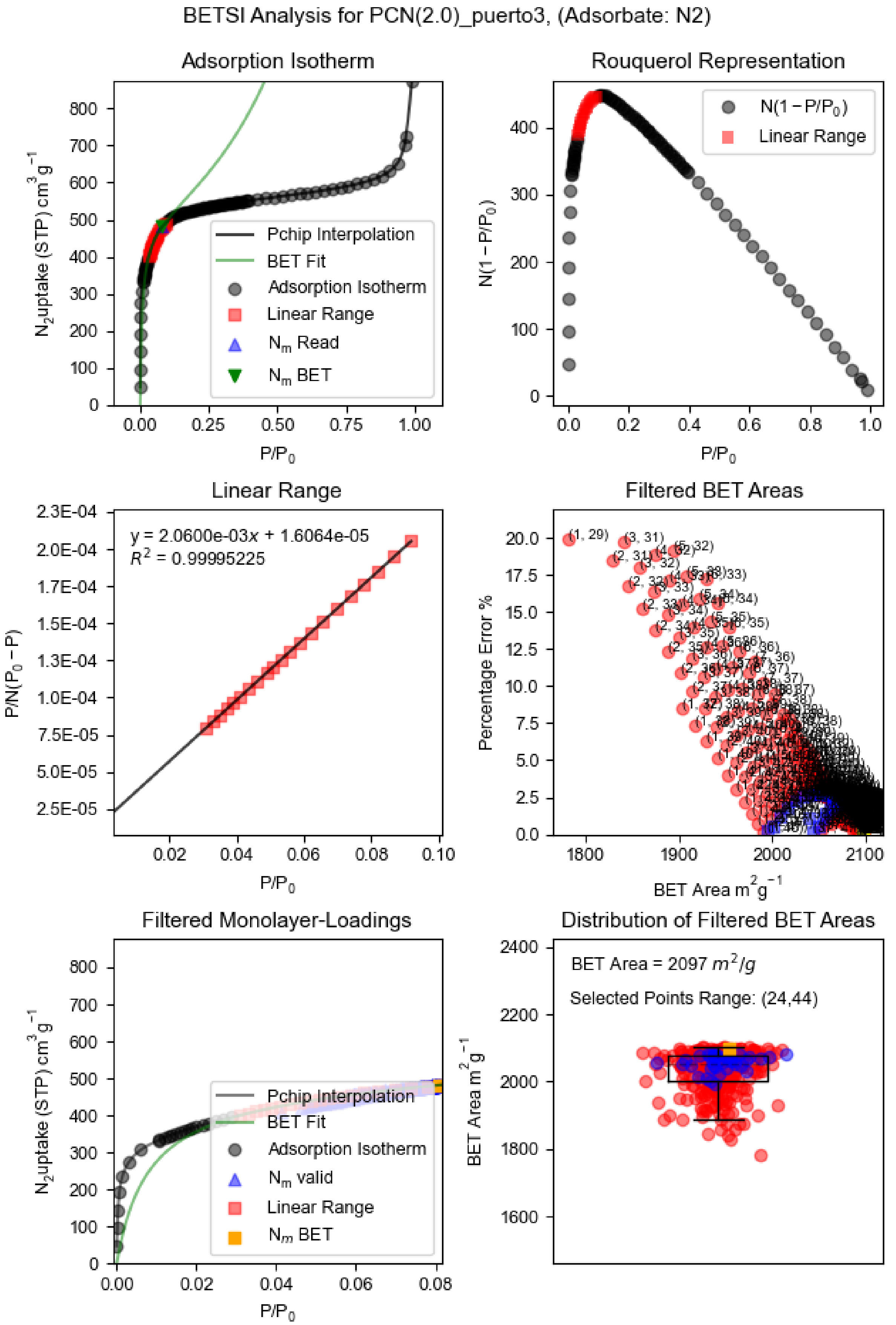

Figure S37. BETSI analysis of Zr-porphyrinic MOF synthesized with L/M 2.00, Mod(AA)/M 560, RT, 1h of reaction and $Zr(OEt)_4$ as precursor.

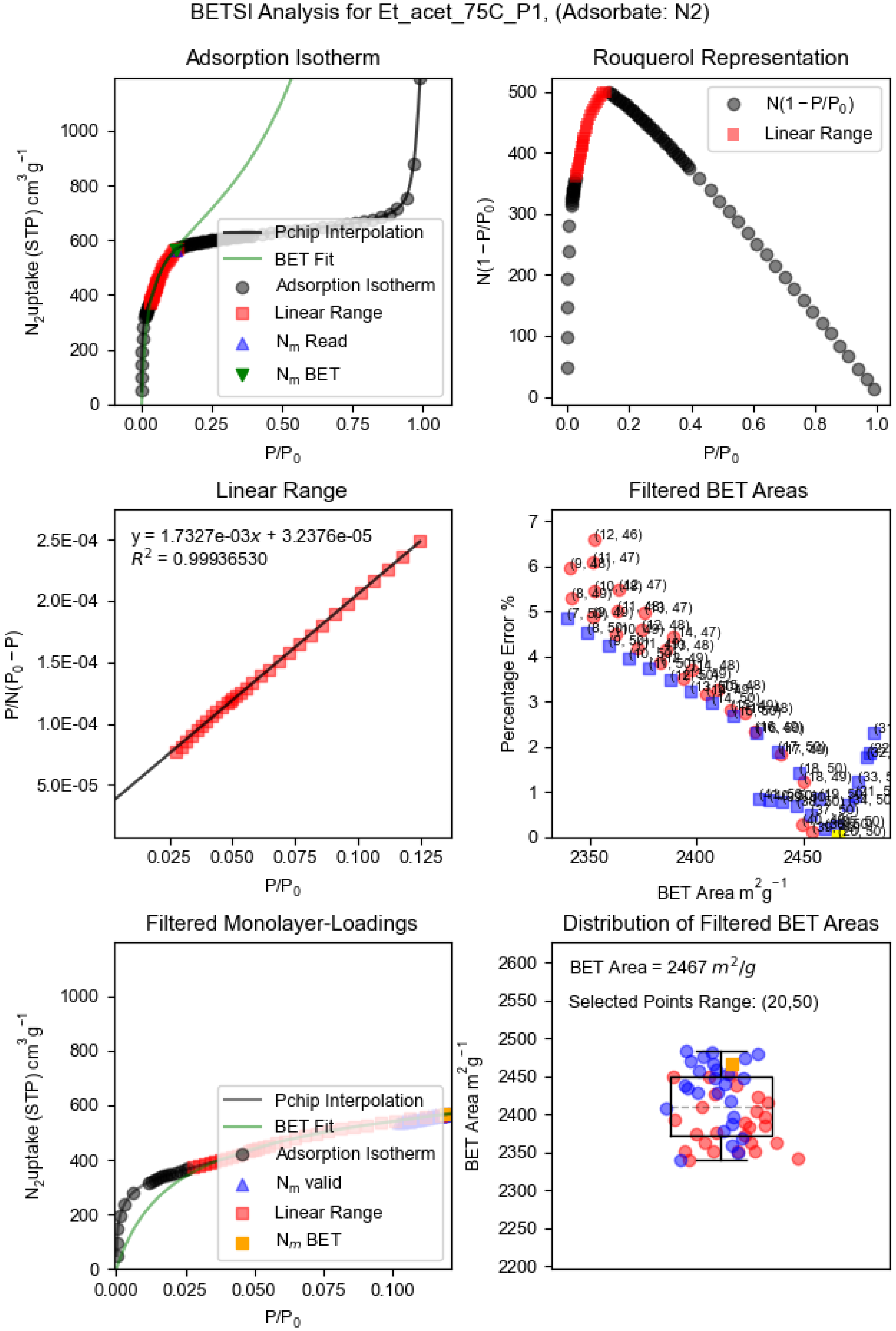

Figure S38. BETSI analysis of Zr-porphyrinic MOF synthesized with L/M 0.25, Mod(AA)/M 250, 75 ºC, 1h of reaction and $Zr(OEt)_4$ as precursor.

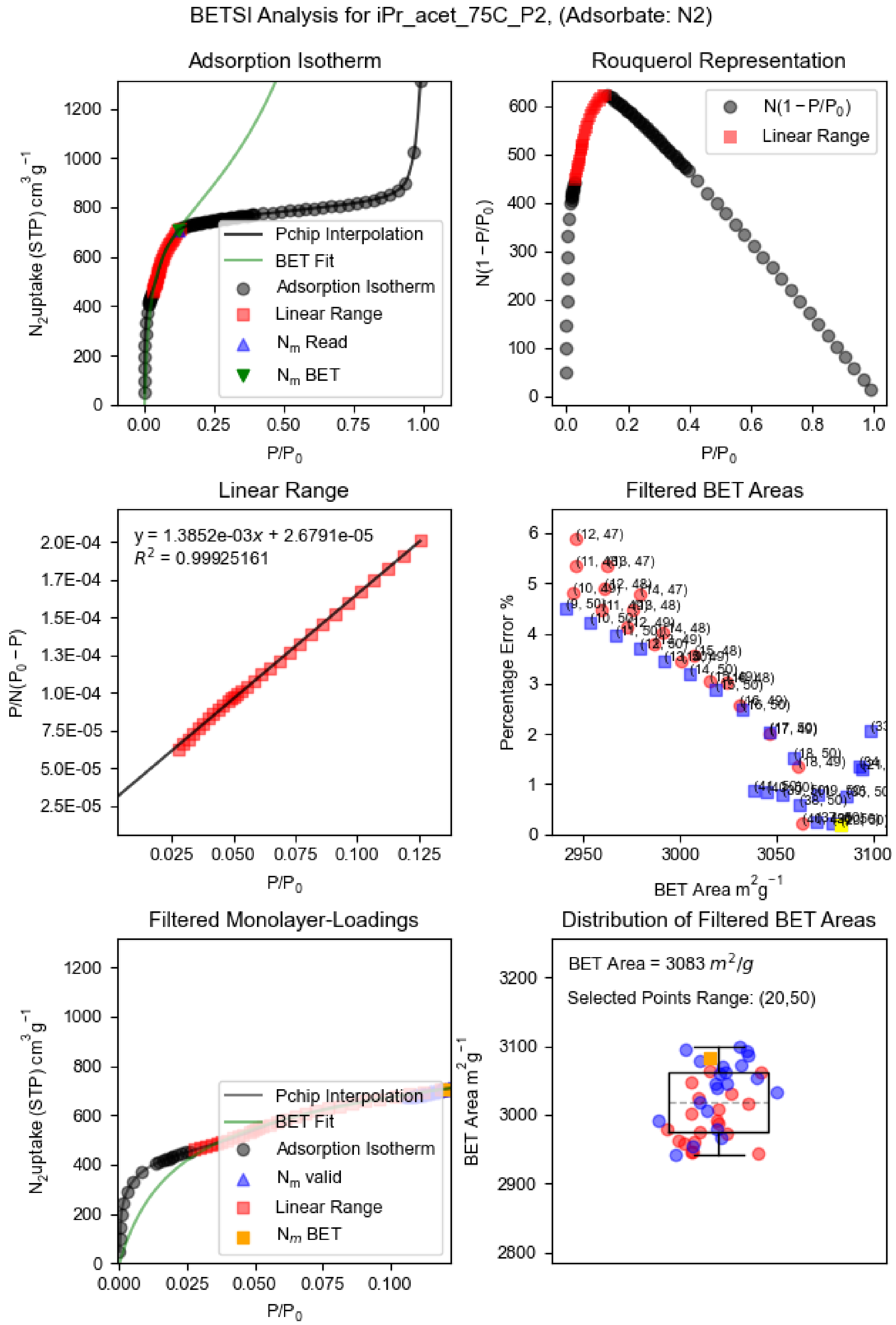

Figure S39. BETSI analysis of Zr-porphyrinic MOF synthesized with L/M 0.25, Mod(AA)/M 250, 75 ºC, 1h of reaction and Zr(OiPr)$_4$ as precursor.

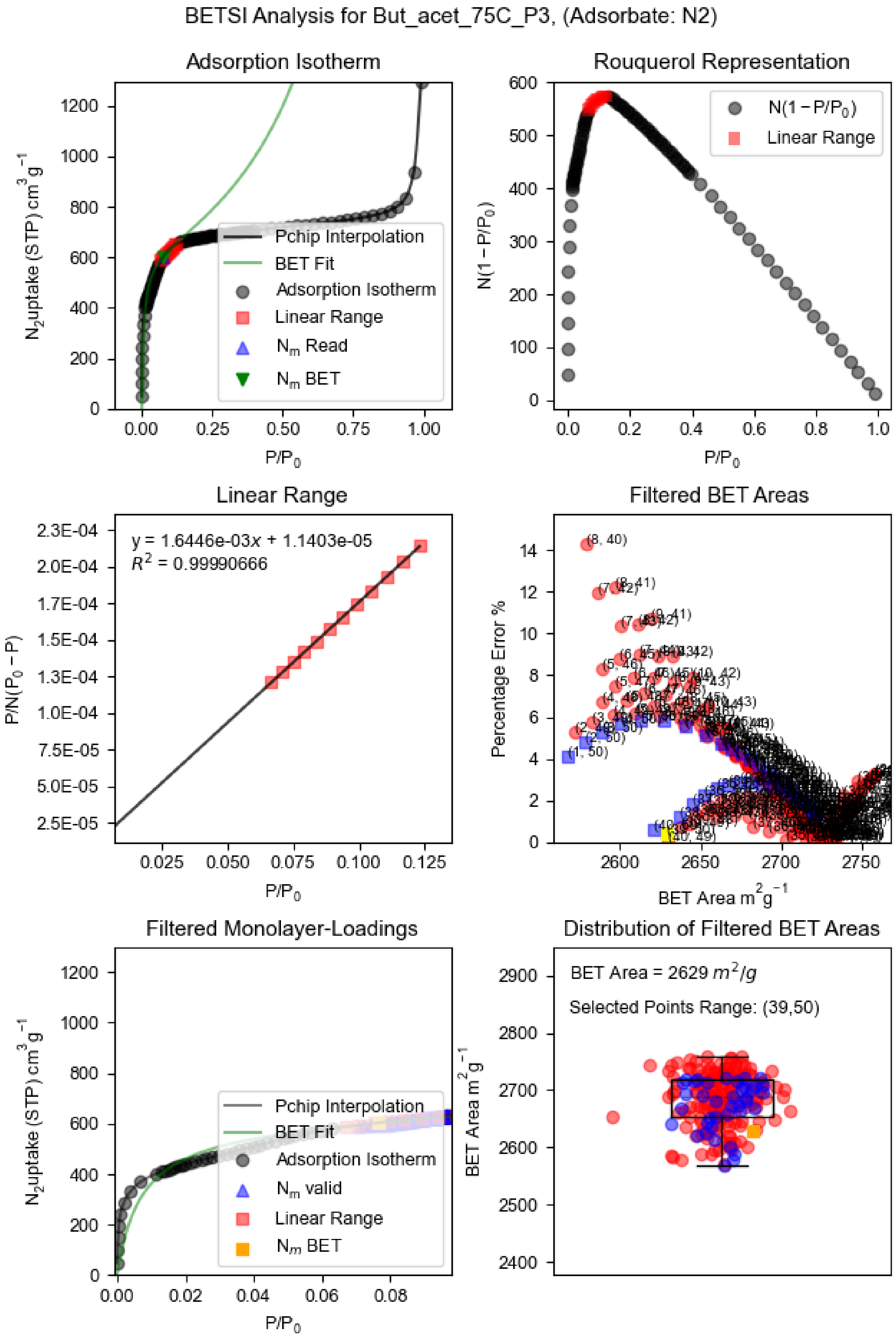

Figure S40. BETSI analysis of Zr-porphyrinic MOF synthesized with L/M 0.25, Mod(AA)/M 250, 75 ºC, 1h of reaction and Zr(OiBu)$_4$ as precursor.

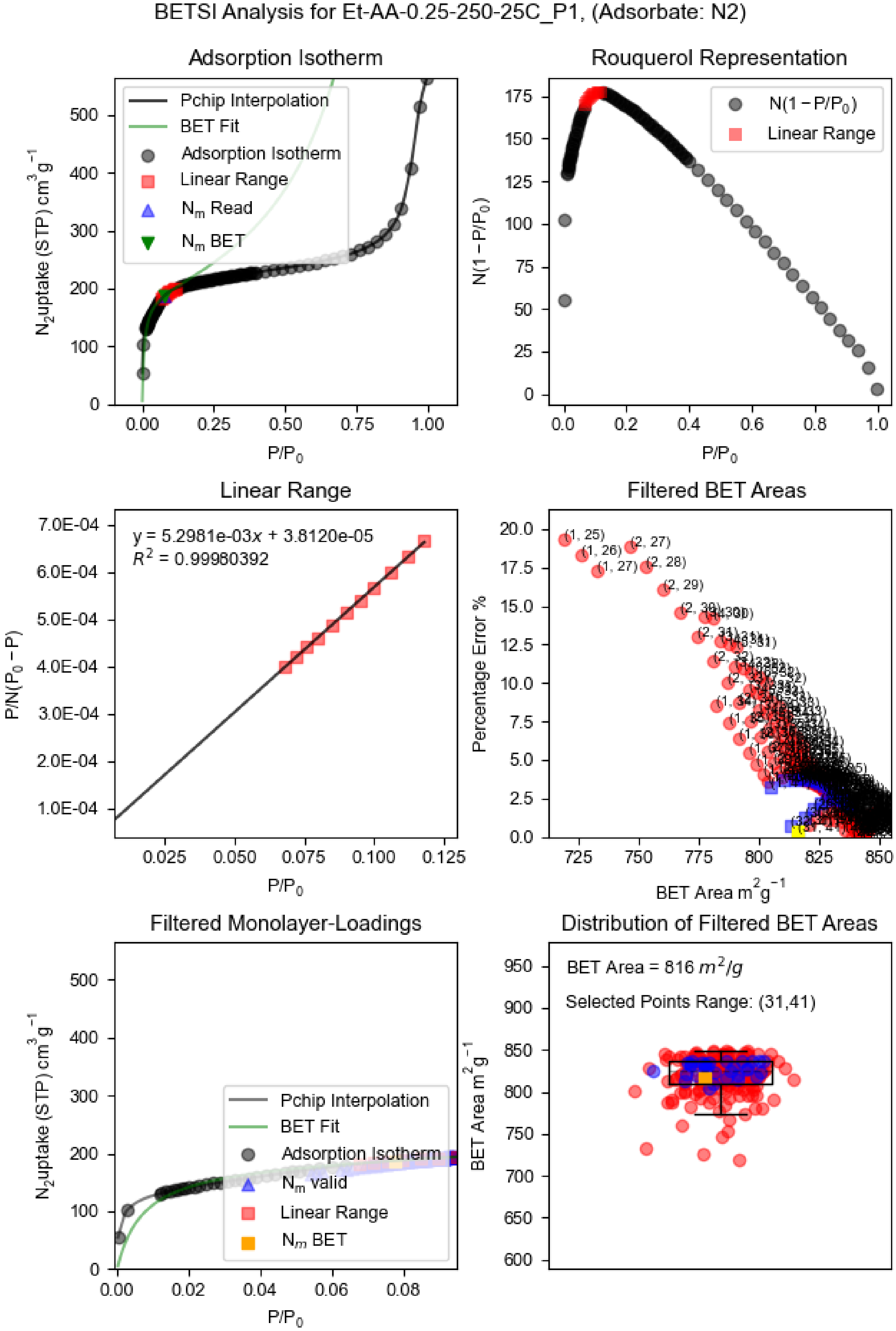

Figure S41. BETSI analysis of Zr-porphyrinic MOF synthesized with L/M 0.25, Mod(AA)/M 250, RT, 1h of reaction and $Zr(OEt)_4$ as precursor.

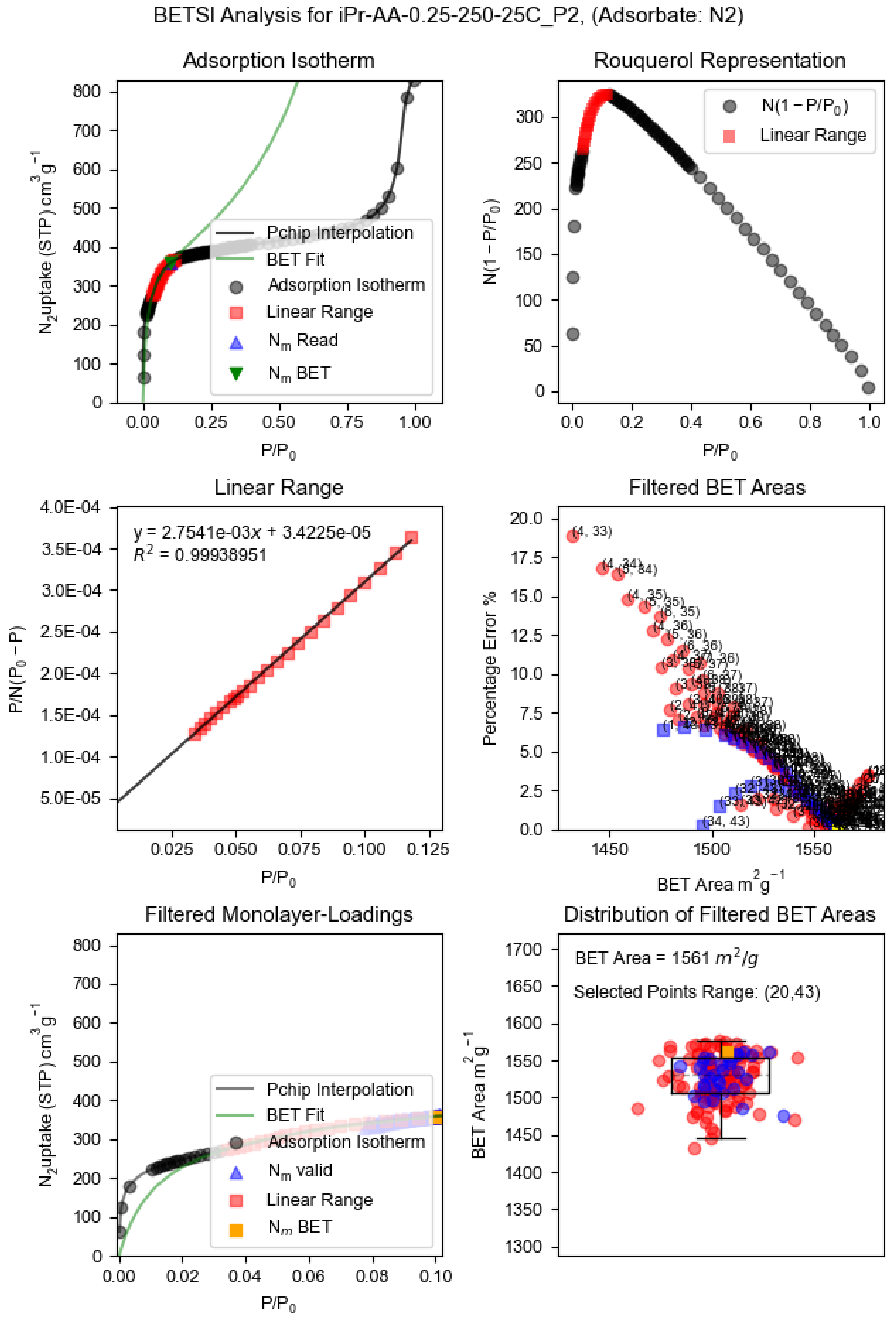

Figure S42. BETSI analysis of Zr-porphyrinic MOF synthesized with L/M 0.25, Mod(AA)/M 250, RT, 1h of reaction and $Zr(OiPr)_4$ as precursor.

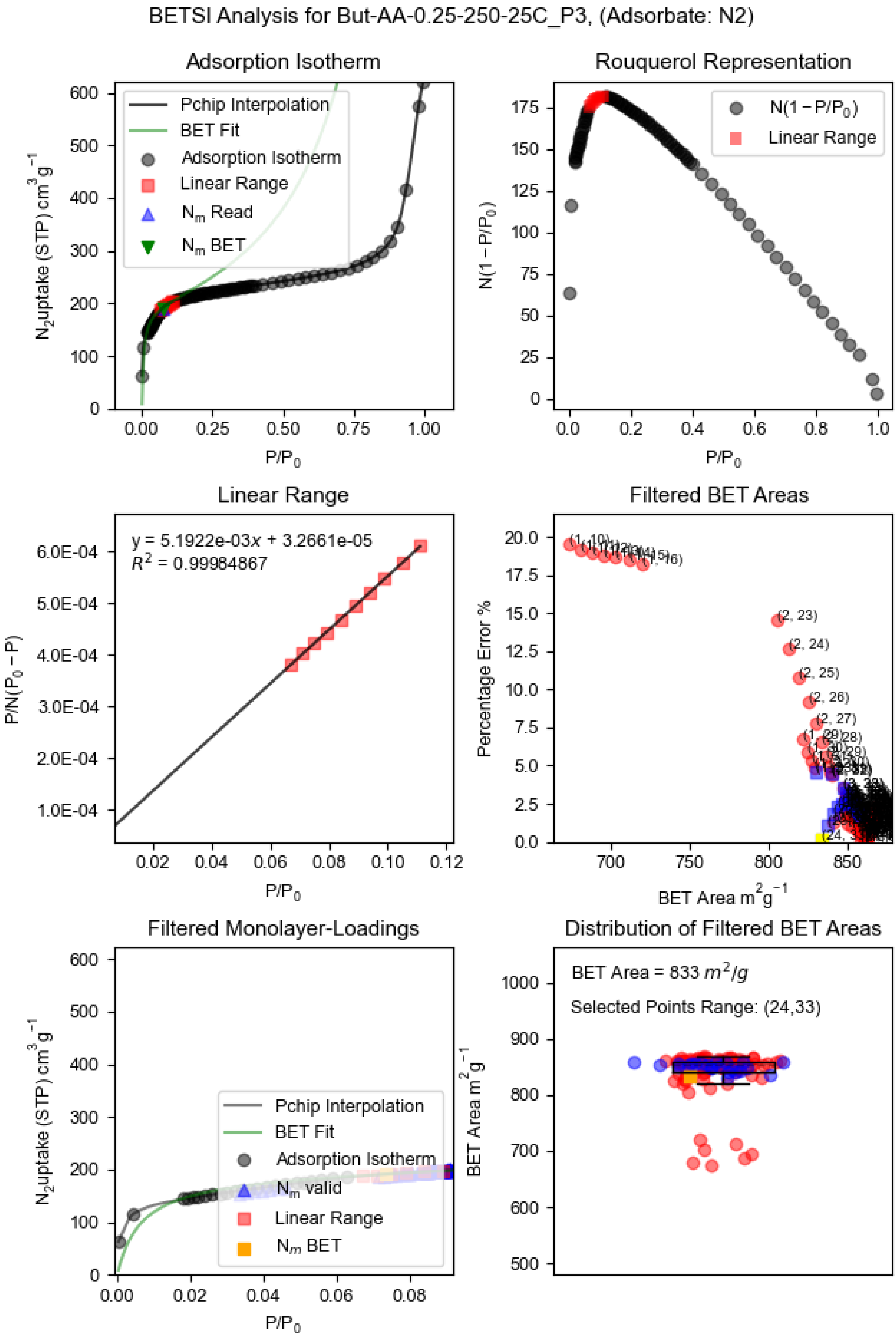

Figure S43. BETSI analysis of Zr-porphyrinic MOF synthesized with L/M 0.25, Mod(AA)/M 250, RT, 1h of reaction and Zr(OiBu)$_4$ as precursor.

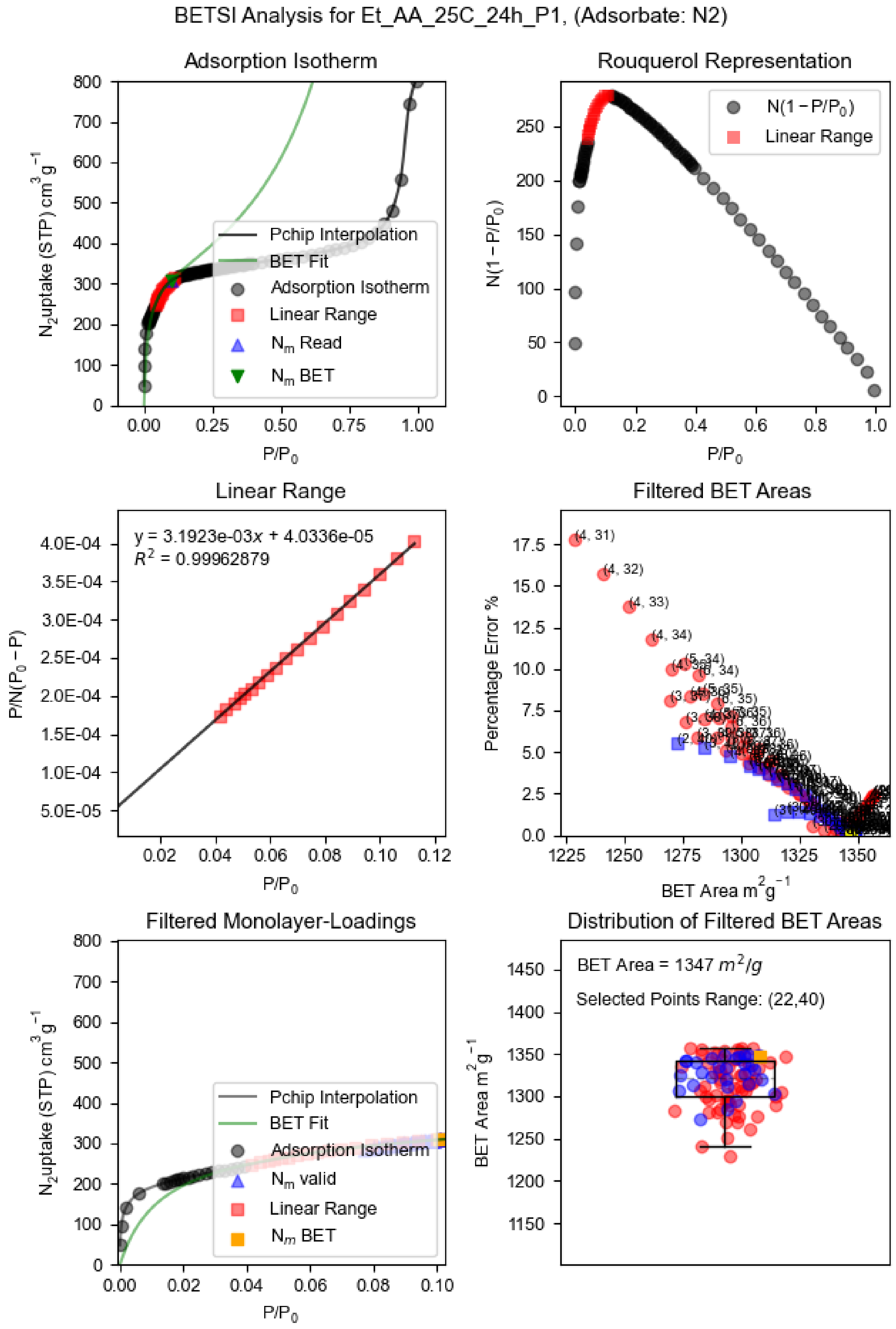

Figure S44. BETSI analysis of Zr-porphyrinic MOF synthesized with L/M 0.25, Mod(AA)/M 250, RT, 24h of reaction and Zr(OEt)4 as precursor.

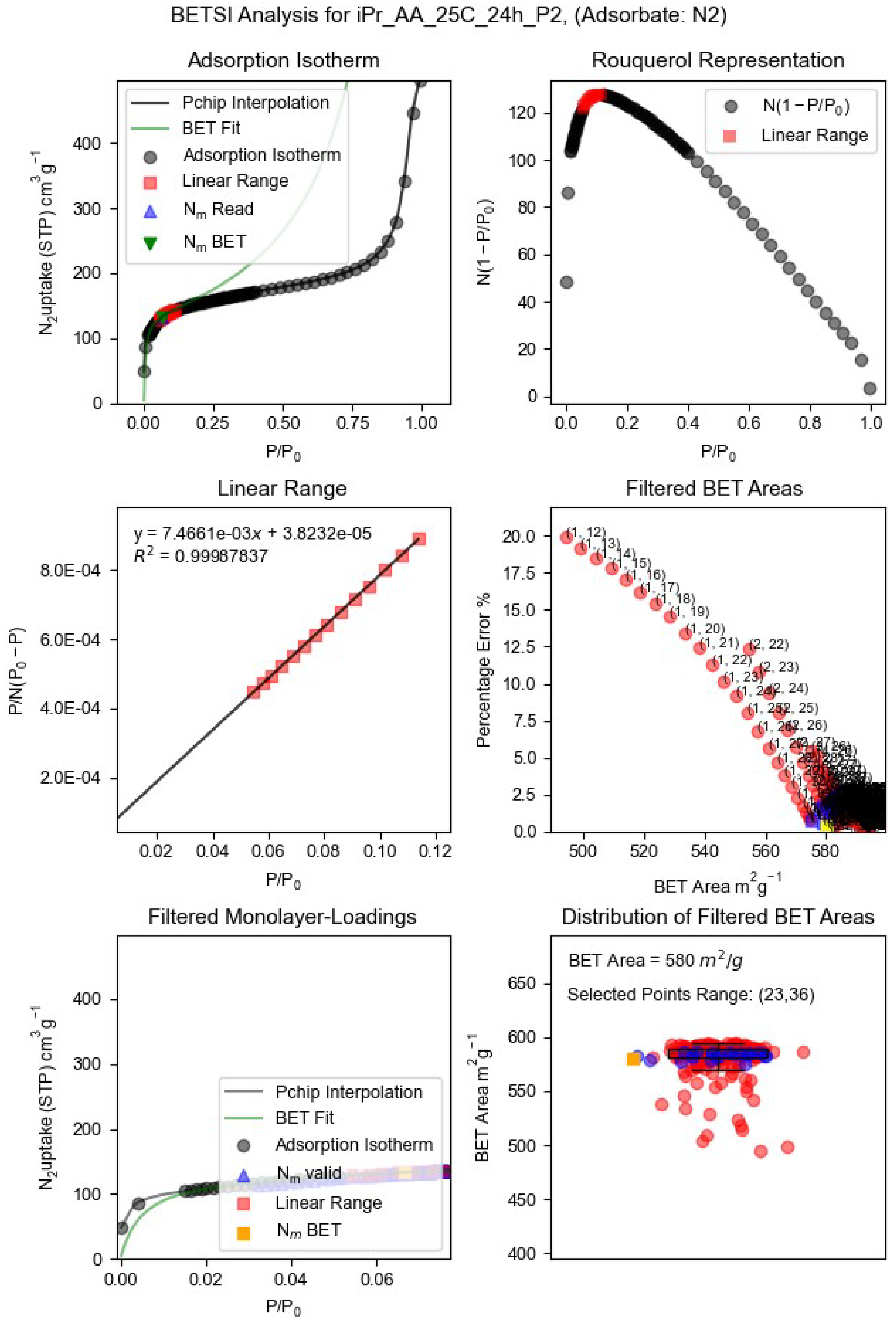

Figure S45. BETSI analysis of Zr-porphyrinic MOF synthesized with L/M 0.25, Mod(AA)/M 250, RT, 24h of reaction and $Zr(OiPr)_4$ as precursor.

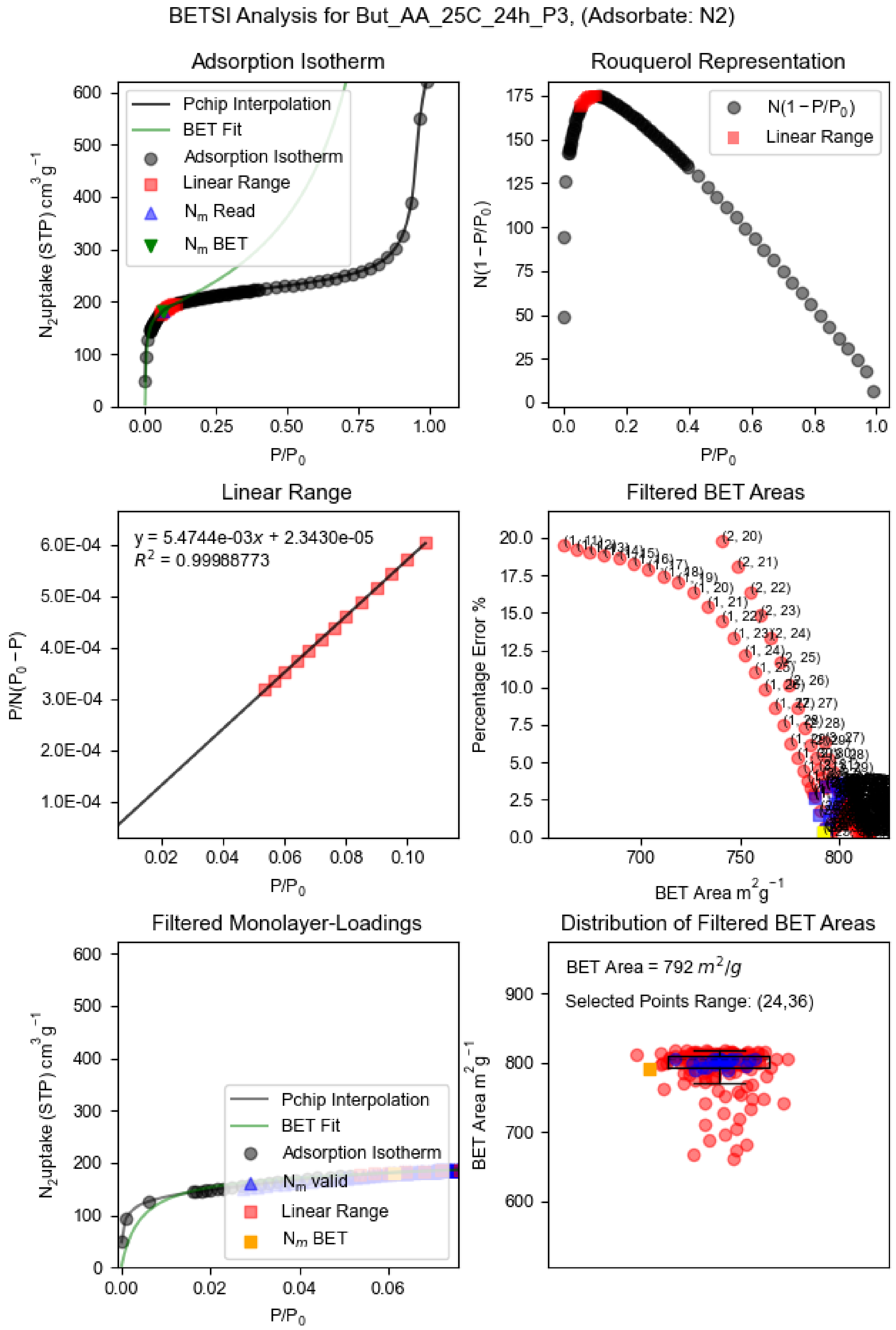

Figure S46. BETSI analysis of Zr-porphyrinic MOF synthesized with L/M 0.25, Mod(AA)/M 250, RT, 24h of reaction and $Zr(OBu)_4$ as precursor.

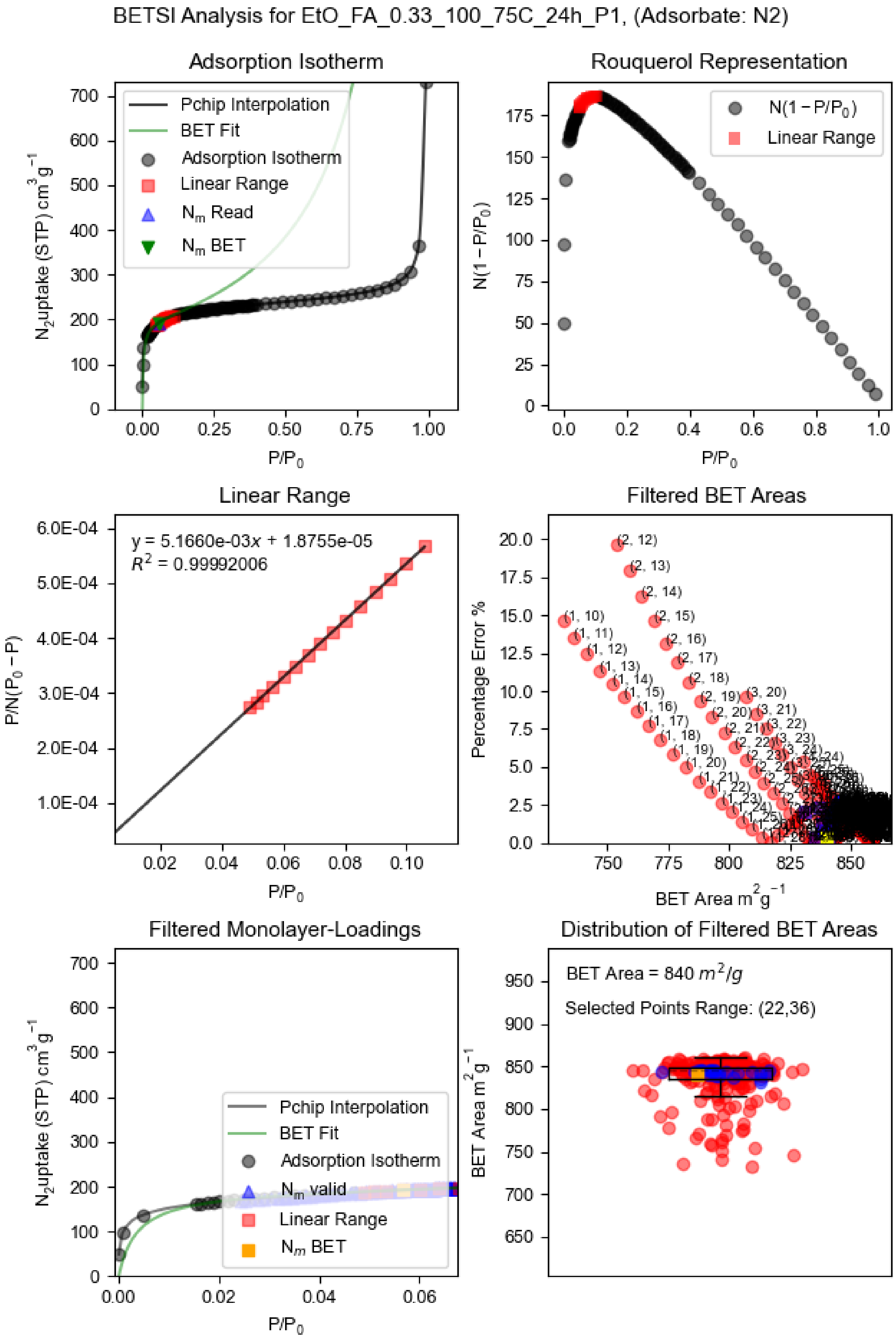

Figure S47. BETSI analysis of Zr-porphyrinic MOF synthesized with L/M 0.33, Mod(FA)/M 100, 75 ºC, 24h of reaction and Zr(OEt)4 as precursor.

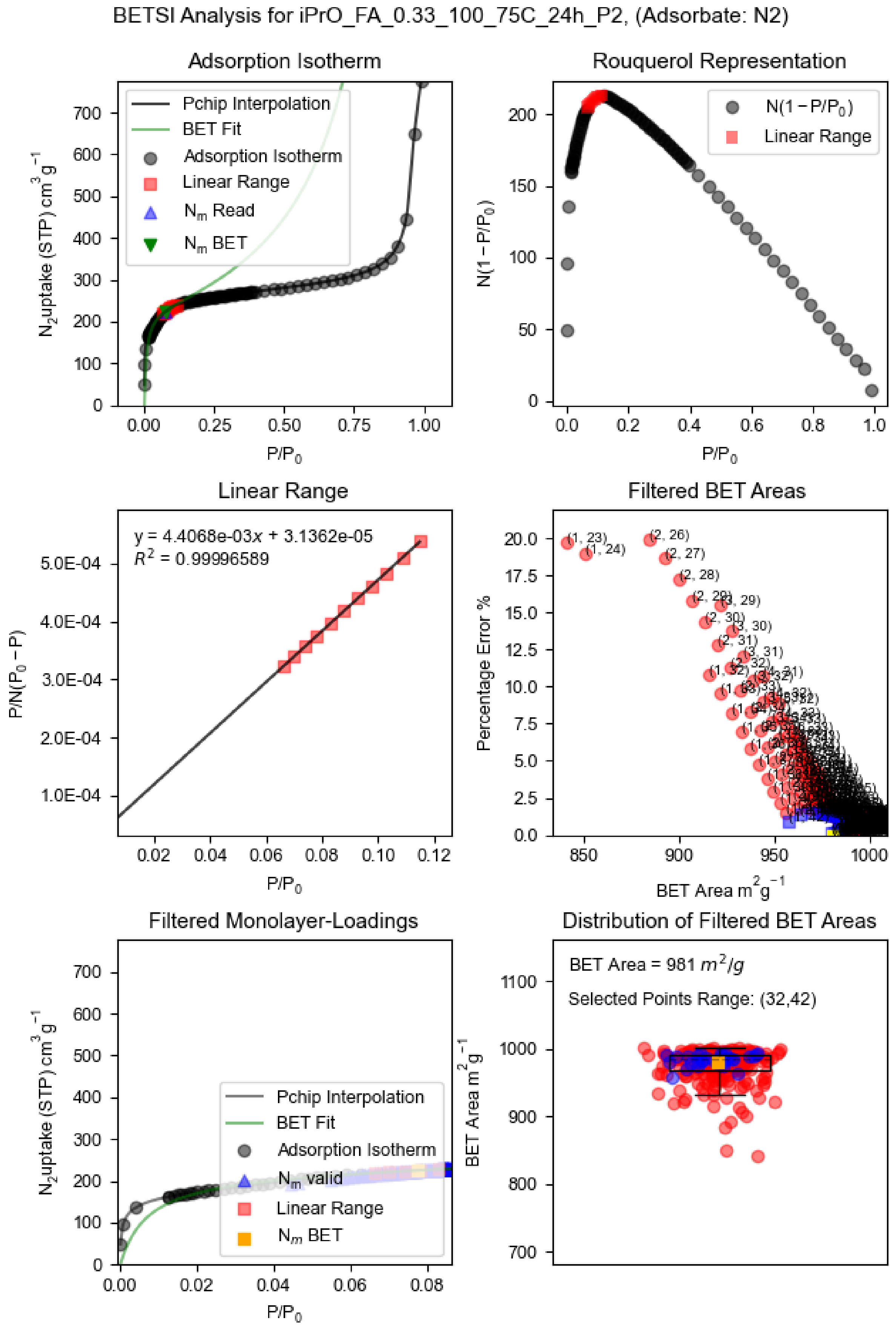

Figure S48. BETSI analysis of Zr-porphyrinic MOF synthesized with L/M 0.33, Mod(FA)/M 100, 75 ºC, 24h of reaction and $Zr(OiPr)_4$ as precursor.

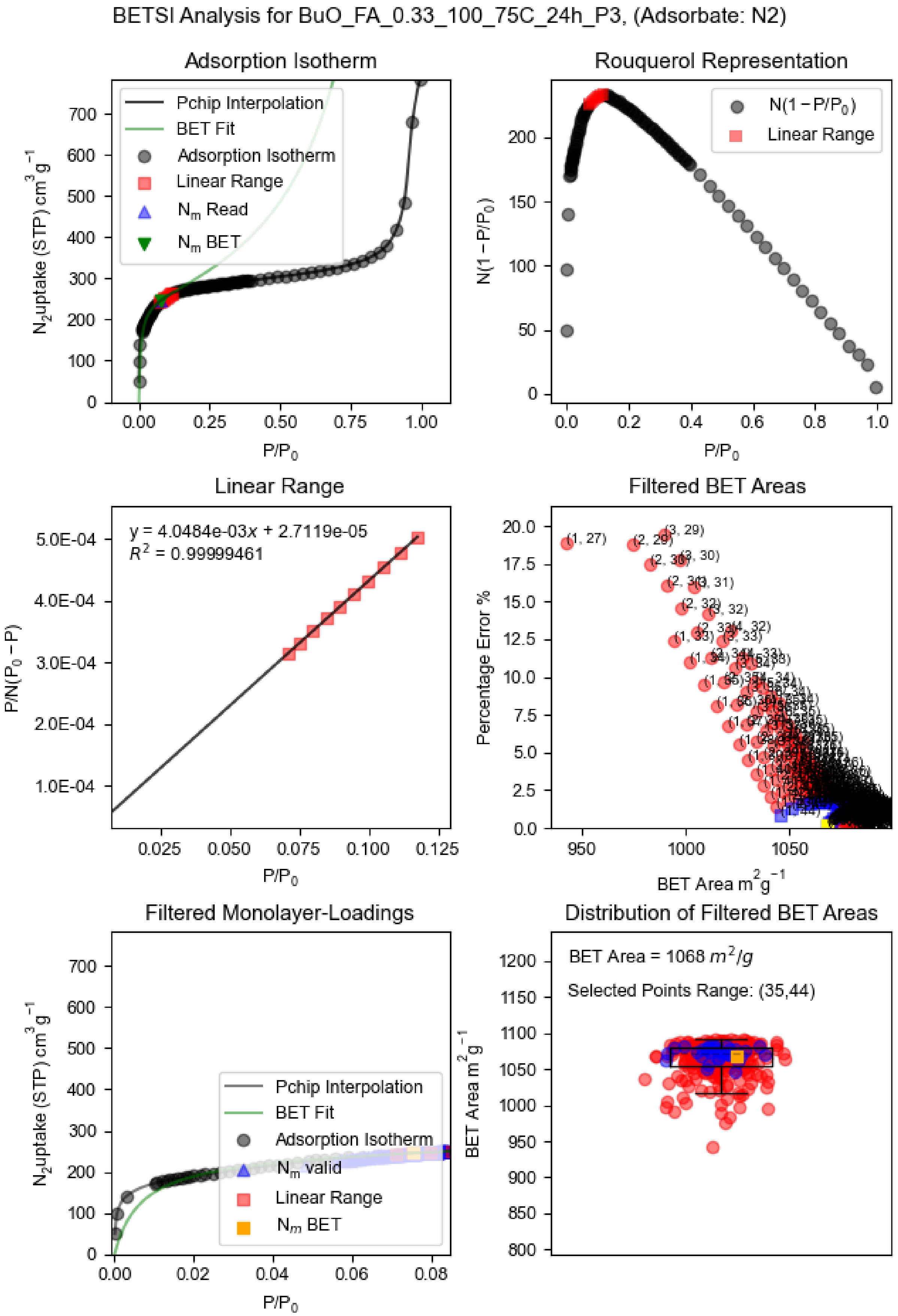

Figure S49. BETSI analysis of Zr-porphyrinic MOF synthesized with L/M 0.33, Mod(FA)/M 100, 75 ºC, 24h of reaction and $Zr(OBu)_4$ as precursor.

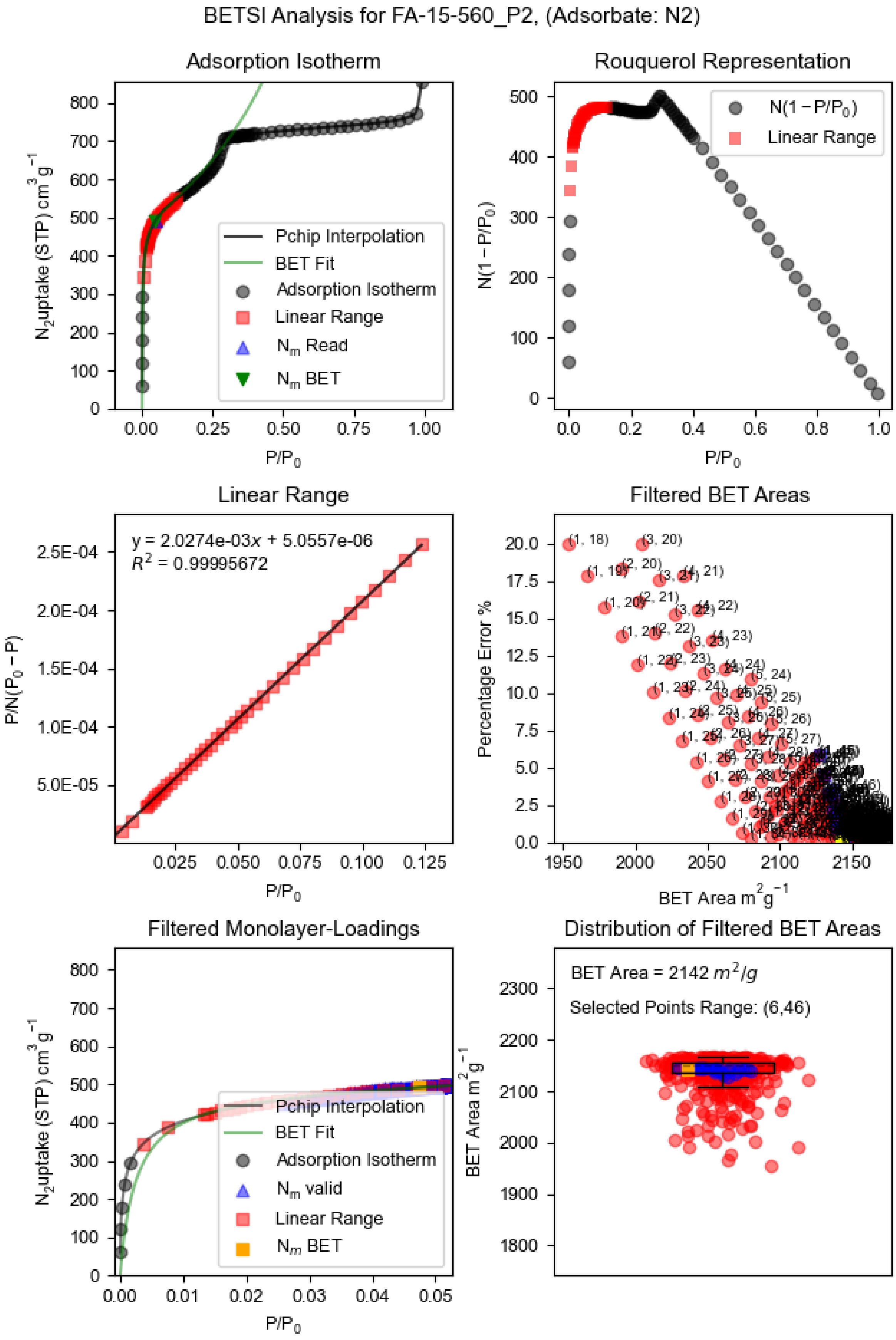

Figure S50. BETSI analysis of Zr-porphyrinic MOF synthesized with L/M 1.50, Mod(FA)/M 560, 75 ºC, 1h of reaction and $Zr(OEt)_4$ as precursor.

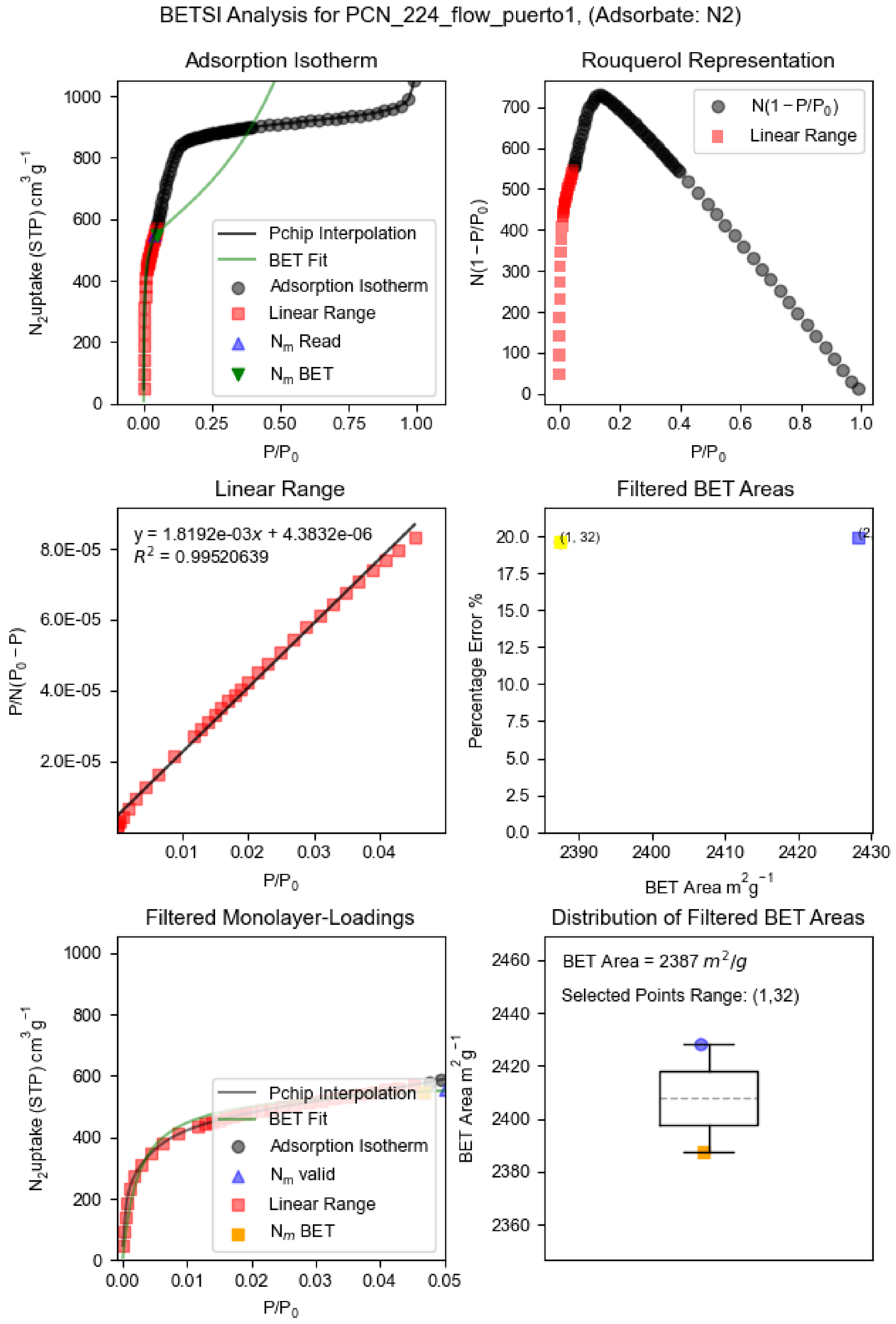

Figure S51. BETSI analysis of Zr-porphyrinic MOF synthesized in flow with L/M 0.25, Mod(AA)/M 560, RT, 25 s of reaction and $Zr(OEt)_4$ as precursor.

References

1. Osterrieth, J.W.M., Rampersad, J., Madden, D., Rampal, N., Skoric, L., Connolly, B., Allendorf, M.D., Stavila, V., Snider, J.L., Ameloot, R., et al. (2022). How Reproducible are Surface Areas Calculated from the BET Equation? Adv. Mater. *34*, 2201502.
2. Chu, J., Ke, F.S., Wang, Y., Feng, X., Chen, W., Ai, X., Yang, H., and Cao, Y. (2020). Facile and reversible digestion and regeneration of zirconium-based metal-organic frameworks. Commun. Chem. *3*, 5.
3. Li, Y., Lo, W.S., Zhang, F., Si, X., Chou, L.Y., Liu, X.Y., Williams, B.P., Li, Y.H., Jung, S.H., Hsu, Y.S., et al. (2021). Creating an Aligned Interface between Nanoparticles and MOFs by Concurrent Replacement of Capping Agents. J. Am. Chem. Soc. *143*, 5182–5190.
4. G, E., K, V., and Nudelman, A. (1997). NRM Chemicals Shifts of common laboratory solvents as traces imputities. J. org. Chem *3263*, 7512–7515.
5. Willems, T.F., Rycroft, C.H., Kazi, M., Meza, J.C., and Haranczyk, M. (2012). Algorithms and tools for high-throughput geometry-based analysis of crystalline porous materials. Microporous Mesoporous Mater. *149*, 134–141.
