# Supplementary material for: Ultrafast synthesis of zirconium-porphyrin framework nanocrystals from alkoxide precursors
Source: Cell Rep Phys Sci. 2024 Dec 18;5(12):102318. doi: 10.1016/j.xcrp.2024.102318 (PMC11659387; doi:10.1016/j.xcrp.2024.102318)
Supplement: Document S2. Article plus supplemental information [file mmc2.pdf]

Cell Reports
Physical Science

Article

Ultrafast synthesis of zirconium-porphyrin framework nanocrystals from alkoxide precursors

Graphical abstract

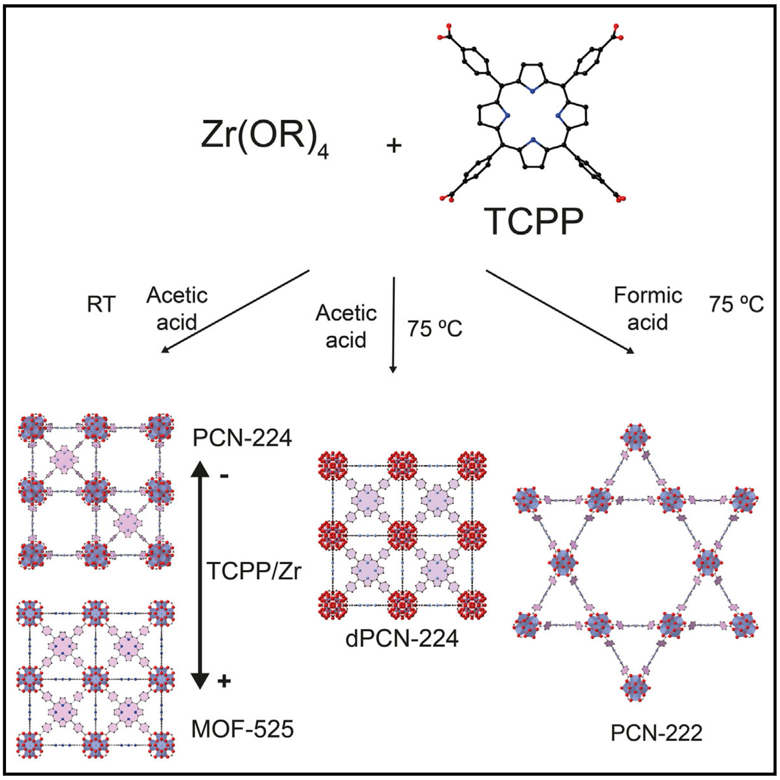

Highlights

- Zr(IV) alkoxides enable the fast, efficient synthesis of porphyrinic MOF nanocrystals
- MOF-525, PCN-224, and PCN-222 are synthesized in minutes with high yields and purity
- Tuning linker and modulator ratios allows precise control of crystal phases
- Room temperature continuous-flow synthesis supports scalable MOF production

Authors

Manuel Ceballos, Giulia Zampini, Oleg Semyonov, ..., Thomas Devic, Beatriz Pelaz, Pablo del Pino

Correspondence

pablo.delpino@usc.es

In brief

Ceballos et al. present a rapid and efficient method using Zr(IV) alkoxides to synthesize porphyrinic MOF nanocrystals, including MOF-525, PCN-224, and PCN-222, under mild conditions. This approach enables precise control over crystal phases and continuous synthesis at room temperature, supporting scalable MOF production with potential applications in technology and medicine.

Ceballos et al., 2024, Cell Reports Physical Science 5, 102318
December 18, 2024 © 2024 The Author(s). Published by Elsevier Inc.
https://doi.org/10.1016/j.xcrp.2024.102318

Cell Reports
Physical Science

CellPress
OPEN ACCESS

Article

Ultrafast synthesis of zirconium-porphyrin framework nanocrystals from alkoxide precursors

Manuel Ceballos,[1] Giulia Zampini,[2] Oleg Semyonov,[2] Samuel Funes-Hernando,[1] José Manuel Vila-Fungueiriño,[3] Sonia Martínez-Giménez,[4] Sergio Tatay,[4] Carlos Martí-Gastaldo,[4] Thomas Devic,[5] Beatriz Pelaz,[6] and Pablo del Pino[1,7,*]

[1]Centro Singular de Investigación en Química Biolóxica e Materiais Moleculares (CiQUS), Departamento de Física de Partículas, Universidade de Santiago de Compostela, 15782 Santiago de Compostela, Spain

[2]Centro Singular de Investigación en Química Biolóxica e Materiais Moleculares (CiQUS), Universidade de Santiago de Compostela, 15782 Santiago de Compostela, Spain

[3]Centro Singular de Investigación en Química Biolóxica e Materiais Moleculares (CiQUS), Departamento de Química Física, Universidade de Santiago de Compostela, 15782 Santiago de Compostela, Spain

[4]Instituto de Ciencia Molecular (ICMol), Universitat de València, Catedrático José Beltrán-2, 46980 Paterna, Spain

[5]Nantes Université, CNRS, Institut des Matériaux de Nantes Jean Rouxel, IMN, 44000 Nantes, France

[6]Centro Singular de Investigación en Química Biolóxica e Materiais Moleculares (CiQUS), Departamento de Química Inorgánica, Universidade de Santiago de Compostela, 15782 Santiago de Compostela, Spain

[7]Lead contact

*Correspondence: pablo.delpino@usc.es

https://doi.org/10.1016/j.xcrp.2024.102318

SUMMARY

Porphyrinic metal-organic frameworks (MOFs) offer high surface areas and tunable catalytic and optoelectronic properties, making them versatile candidates for applications in phototherapy, drug delivery, photocatalysis, electronics, and energy storage. However, a key challenge for industrial integration is the rapid, cost-effective production of suitable sizes. This study introduces Zr(IV) alkoxides as metal precursors, achieving ultrafast (~minutes) and high-yield (>90%) synthesis of three well-known Zr-based porphyrinic MOF nanocrystals: MOF-525, PCN-224, and PCN-222, each with distinct topologies. By adjusting linker-to-metal and modulator-to-metal ratios, we attain precise control over single-phase formation. Demonstrating alkoxides' potential, we synthesized nanosized PCN-224 at room temperature within seconds using a continuous multifluidic method. This advancement greatly simplifies porphyrinic MOF production, enabling broader industrial and scientific applications.

INTRODUCTION

Zirconium-based metal-organic frameworks (MOFs) are among the most promising microporous materials for a wide range of applications, from energy production and environmental remediation to biomedical uses in creating theragnostic nanocarriers.[1–3] Most Zr-carboxylate MOFs rely on the robust secondary building unit (SBU) formed by the Zr_6-oxo cluster, which can connect up to 12 carboxylate ligands, resulting generally in highly stable nets,[4] where the SBU can be tuned to create missing linker defects or missing cluster defects, which directly impact the physicochemical properties.[5] Over 7,600 Zr-based networks have been reported in the literature.[6] One of the most prolific building blocks in the literature, tetrakis(4-carboxyphenyl) porphyrin (TCPP), serves as a tetratopic carboxylate ligand that, when combined with the Zr_6-oxo cluster, has led to the renowned MOF networks MOF-525,[7] PCN-222 (also known as MOF-545 or MMPF-6),[7–9] and PCN-224,[10] with connectivities of 12, 8, and 6, respectively. Other notable examples include the 8-connected (8-c) polymorphs PCN-225 and NU-902,[11,12] as well as the 12-c PCN-223.[13] The recent development of dPCN-224, a disordered variant of PCN-224, which incorporates the Zr_6-oxo cluster in four spatial orientations,[14] has elucidated the previously puzzling short and unrealistic Zr-Zr distance of 2.69 Å found in the Zr_8O_6 cluster in PCN-221.[15] These porphyrinic frameworks exhibit exceptionally high surface areas (~2,500 m^2/g) and are remarkably versatile, presenting significant potential as nanozymes for myriad applications,[16] including photocatalysis,[17] energy production,[18] photodynamic therapy,[19] and drug delivery.[20]

A recent interlaboratory study, led by Lotsch's group, highlighted challenges in achieving reproducible syntheses of pure PCN-222 and PCN-224.[21] This study identified that the synthesis process is sensitive to factors that are not yet fully understood, with the major challenge being the identification and control of these variables. Interestingly, another recent study by the same group suggested that the amount of water used during synthesis directly affects the speciation and phase formation of Zr-porphyrinic MOFs.[22]

Although metal chlorides/oxochlorides have been the preferred metal sources for many years, the mechanism underlying the formation of the Zr_6-oxo cluster remains not yet fully elucidated. Some reports indicate that dimethylformamide (DMF) and water are essential for forming an intermediate

 © 2024 The Author(s). Published by Elsevier Inc.
This is an open access article under the CC BY license (http://creativecommons.org/licenses/by/4.0/).

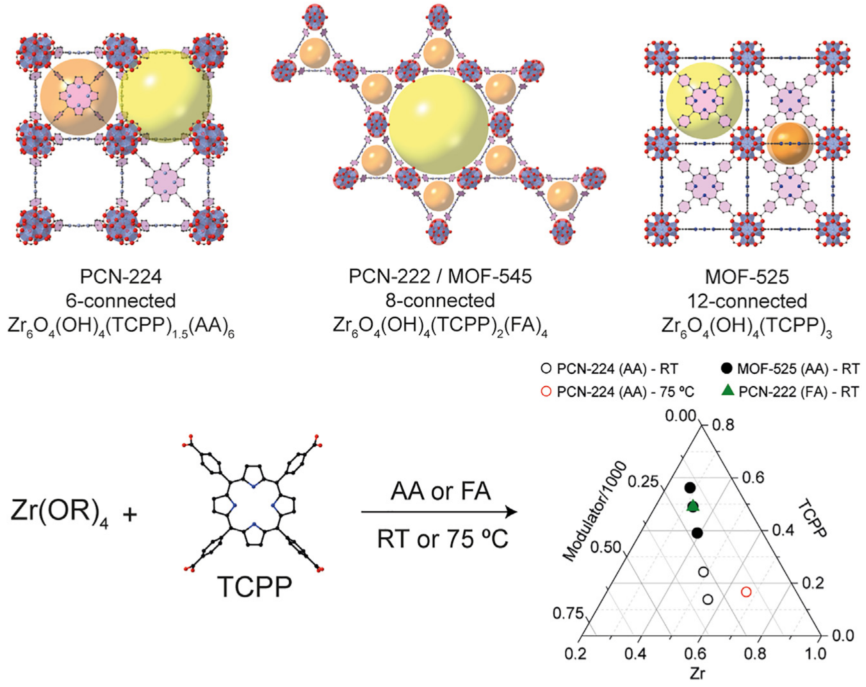

Scheme 1. Variations in connectivity and organic linker arrangements in porphyrinic MOFs

Top row: comparative structures of three different MOFs: PCN-224, PCN-222/MOF-545, and MOF-525, showing varying connectivity and organic linker arrangements. Bottom row: synthesis approach utilizing Zr-alkoxide precursors and TCPP. By adjusting the linker-to-metal (L/M) and modulator-to-metal (Mod/M) ratios, as well as the choice of modulator, we facilitate the formation of single phases of the depicted MOFs. These phases are further delineated in the tertiary diagram (bottom right).

$[ZrCl(OH)_2(DMF)_2]Cl$. This intermediate then reacts with a carbonylated ligand to form the corresponding MOF.[23] In more recent research, high-resolution mass spectrometry revealed that DMF undergoes partial hydrolysis, producing dimethylammonium species that subsequently exchange with zirconium atoms.[24] Additionally, in this work, chloride ions from $ZrCl_4$ were found to play a crucial role in the formation of zirconium chloroterephthalates, as demonstrated in the synthesis of UiO-66. This finding has sparked controversy, particularly regarding the use of other zirconium precursors, such as chloride-free alkoxides, which may impact the reaction pathway and the structure of the resulting MOFs.

While metal alkoxides have been extensively utilized in sol-gel synthesis due to their rapid hydrolysis, enabling the synthesis of catalytically active metal oxides such as Al_2O_3, Y_2O_3, ZrO_2, or TiO_2,[25] their use as metal sources in Zr-MOF synthesis is more limited. They have been employed in the early synthesis of molecular oxo clusters like $[Zr_6(\mu_3\text{-}O)_4(\mu_3\text{-}OH)_4]^{12+}$ stabilized with various carboxylic acids.[26–28] These clusters were next used as precursors to produce Zr-based MOFs that, because of cluster facile hydrolysis, could be prepared in mild conditions[29] and with short reaction times while avoiding the formation of ordered defects and impurities[30] and preventing the formation of polymorphs.[31] The benefits of using alkoxides as metal sources also include high reaction yields, and the production of alcohols as by-products rather than hydrochloric acid or chlorides may impact the reaction pathway and structure of the resulting MOFs.[24] Moreover, their use helps to control the amount of water used during synthesis that has been shown to directly affect the speciation and phase formation of Zr-porphyrinic MOFs.[22] Nonetheless, Zr alkoxides have scarcely been used for the direct synthesis of Zr-based MOFs.[32–35] On top of that, alkoxide rapid hydrolysis makes them well suited for the synthesis of MOF nanocrystals (nanoMOFs) because it favors seeding during the crystallization process. These nanoMOFs often possess a larger outer surface area, which imparts distinct properties such as higher surface energy and a greater number of defects. These properties can significantly affect the flexibility, processability, and catalytic performance of the MOFs.[36]

In this work, we demonstrate that Zr alkoxide precursors facilitate the rapid and straightforward synthesis of nanosized MOF-525, PCN-224, and PCN-222 under mild synthetic conditions, crucially avoiding the mixing of crystalline phases. Most importantly, we show that our synthetic protocol can be adapted to the production of PCN-224 in continuous microflow reaction conditions.

As depicted in Scheme 1, the targeted synthesis of MOF-525, PCN-224, or PCN-222 can be accomplished by Zr alkoxide precursors by adjusting the linker-to-metal (L/M) and modulator-to-metal (Mod/M) ratios and changing the modulator from acetic acid (AA) to formic acid (FA) (Tables S1–S3). The ternary diagram shown in Scheme 1 provides a comprehensive overview of the synthetic conditions used to achieve pure phases of MOF-525, PCN-224, and PCN-222.

RESULTS AND DISCUSSION

Influence of linker-to-metal ratio at RT on MOF-525 and PCN-224 synthesis

This initial section explores the influence of the L/M ratio on the crystal phase of the attempted solids while keeping other potential reaction variables constant. We maintained a constant reaction time of 1 h at room temperature (RT) and used $Zr(OEt)_4$ as the metal precursor and AA as the modulator. The Mod/M ratio was set to 560, inspired by previous work.[31] To synthetize the porphyrinic framework with the lowest coordination connectivity, specifically the 6-c PCN-224, we initially set the L/M ratio to 0.25. This value aligns with the ideal formula of this MOF, $Zr_6O_4(OH)_4(TCPP)_{1.5}(AA)_6$, with the anticipation that higher values would favor the formation of phases of higher

CellPress
OPEN ACCESS

connectivity, such as the 8-c PCN-222 or the 12-c MOF-525. We then gradually increased the L/M ratio up to 2.00, four times the amount necessary for maximal connectivity within the Zr_6-oxo cluster of the canonical 12-c MOF-525. A comprehensive physicochemical characterization was conducted on the five purified solids, which were examined both as dried solids, to assess the coordination degree of the Zr_6-oxo cluster and the potential presence of defects, and as colloidal dispersions.

We confirmed the crystalline nature of the purified solids through powder X-ray diffraction (PXRD) analysis. Figures 1A and 1B display the PXRD diffractogram and its detailed magnification for the five solids studied, along with the simulated patterns for PCN-224 and MOF-525. Figure 1B presents close-up views of the initial five diffraction reflections. Notably, the absence of the (110) diffraction reflection and the broadening of the (211) diffraction reflection, with very low intensity visible only after normalization (Figure 2A), at two-theta angles of 3.2° and 5.6°, characteristic of PCN-224, are evident for L/M ratios of 1.00, 1.50, and 2.00, indicating the presence of MOF-525. To corroborate the absence of this reflection (110) for the higher L/M ratios, small-angle diffraction was performed (Figure S1). Overall, summarizing the PXRD data in Figures 1A and 1B, the L/M ratios in the stoichiometric condition for PCN-224 (L/M = 0.25) and the double (L/M = 0.50) suggest the formation of PCN-224 solids, while overpassing this L/M ratio, i.e., 1.00, 1.50, and 2.00, suggests the formation of MOF-525, compatible with Pawley refinement (Figure S2).

Figures 1C–1G display transmission electron microscopy (TEM) images of the five solids at L/M ratios ranging from 0.25 to 2.00. Average particle sizes for these ratios were measured as 527.7 ± 251.7, 193.0 ± 61.4, 105.0 ± 22.9, 97.1 ± 17.5, and 102.8 ± 16.3 nm, respectively (Figures S3 and S4) A trend is observed where an increase in L/M ratio leads to a decrease in both particle size and polydispersity, as well as a decrease in crystallite size calculated using the Scherrer equation (Equation S1; Table S4). Furthermore, high-resolution TEM (HR-TEM) images (Figures 1C–1G) confirm the stability of MOF structures under an 80 kV electron beam, with fast Fourier transform (FFT) analyses on designated regions of interest (dashed ROIs) providing critical d-spacing measurements that illustrate a transition to the MOF-525 phase at higher L/M ratios in agreement with the PXRD analysis results depicted in Figures 1A and 1B.

The thermogravimetric analysis (TGA) of the dried crystalline solids clearly demonstrated the high thermal stability of the samples, enduring temperatures up to 400°C (Figure 2A), as expected for such Zr frameworks (Figure S5).[13] For lower L/M ratios (0.25 and 0.50), the analysis revealed an inorganic residue constituting ~33% of the overall weight, attributable to residual ZrO_2, which strongly agrees with the theoretical residue of the 6-c $Zr_6O_4(OH)_4(TCPP)_{1.5}(OAc)_6$ of 33.4%, following thermal decomposition. For intermediate (1.00) and higher (1.50 and 2.00) L/M ratios, inorganic residues of around 27% and 25% were obtained, respectively. The latter being in full agreement with the theoretical value of the 12-c MOF-525 ($Zr_6O_4(OH)_4(TCPP)_3$), i.e., 24.3%.

The normalized TGA results illustrate that the two solids with the lower L/M ratio reflect the stoichiometric ratio expected for 6-c PCN-224, with the theoretical formula $Zr_6O_4(OH)_4(TCPP)_{1.5}(OAc)_6$ indicating full coordination with modulator AA. At a moderate L/M ratio (1.00), the degree of coordination involved fewer than 10 TCPP molecules. This finding suggests a slightly defective variant of the canonical 12-c MOF-525, characterized by a few missing linker defects (12.8%). Finally, for the two higher L/M ratios (1.50 and 2.00), the 12-c expected for MOF-525 is attained. As far as we are aware, there appears to be limited literature providing TGA evidence for a nearly saturated 12-c porphyrinic MOF.[38]

To further validate the TCPP coordination extent of the Zr porphyrinic MOFs, quantification was carried out using ^{1}H-nuclear magnetic resonance (^{1}H-NMR). This process involved dissolving the solids in a deuterated aqueous solution of $NaHCO_3$[39] and incorporating an internal standard (methylsulfonylmethane)[40] to establish a correlation between the proton signal areas and the quantities of TCPP molecules and acetate ions (OAc). This correlation facilitated the determination of the OAc/TCPP ratio (Figure 2B).

In cases of lower L/M ratios with a coordination index of 6, the OAc/TCPP ratios were lower than 4.00 (1.62 and 1.01 for L/M ratios 0.25 and 0.50, respectively). This is compatible with an excess of TCPP molecules, a coordination degree slightly over 8, or the loss of OAc ions in the cluster. At an L/M ratio of 1, the ^{1}H-NMR quantification yielded a ratio of 0.44, closely approximating a coordination of 10. This implies the presence of 10 TCPP molecules for every 2 OAc molecules within a cluster. Furthermore, the two higher L/M ratios (1.50 and 2.00) yielded ratios of 0.10 and 0.22, respectively, being lower than the ratio of 0.36 within the range of 11–12 TCPP molecules per cluster. These results imply that the L/M ratio of 1.50 accommodates more than 11 TCPP molecules per cluster. Similarly, the L/M ratio of 2.00 exhibits an alignment with nearly 12 TCPP molecules per Zr_6-oxo cluster. NMR spectra and quantification data are available in the supplemental information (Figure S6; Table S5).

N_2 adsorption isotherms, measured at 77 K as shown in Figure 2C and detailed in Table S6, reveal the expected differences in N_2 uptake among samples with varying L/M ratios, impacting directly in Brunauer-Emmett-Teller (BET) surface areas, which were calculated with BETSI analysis.[41] Lower L/M ratios (0.50 and 0.25) result in adsorption isotherms and pore width distributions that align with those expected for PCN-224,[10] while higher L/M ratios (1.00, 1.50, and 2.00) lead to a decreased N_2 uptake, a reduction in BET surface areas, and a narrowing of pore size distribution that align with what is expected for MOF-525,[42] as depicted in Figure 2D and elaborated on in Table S7 and Figure S7. Higher ratios feature a primary pore size centered around 12 Å (Figure S7). The N_2 adsorption data thus support and complement the structural characterization previously discussed, underlining the impact of L/M ratio variations on the physicochemical properties of the MOFs.

The UV-visible (UV-vis) spectra of colloidal dispersions of the purified solids in MeOH reveal that at an L/M ratio of 0.25 (black line), the porphyrinic framework characteristic Soret band undergoes a red shift and notable broadening, with significant scattering observed in the four Q bands beyond 515 nm due to large particle sizes (Figure S8A). Increasing the L/M ratio to 0.50 (red

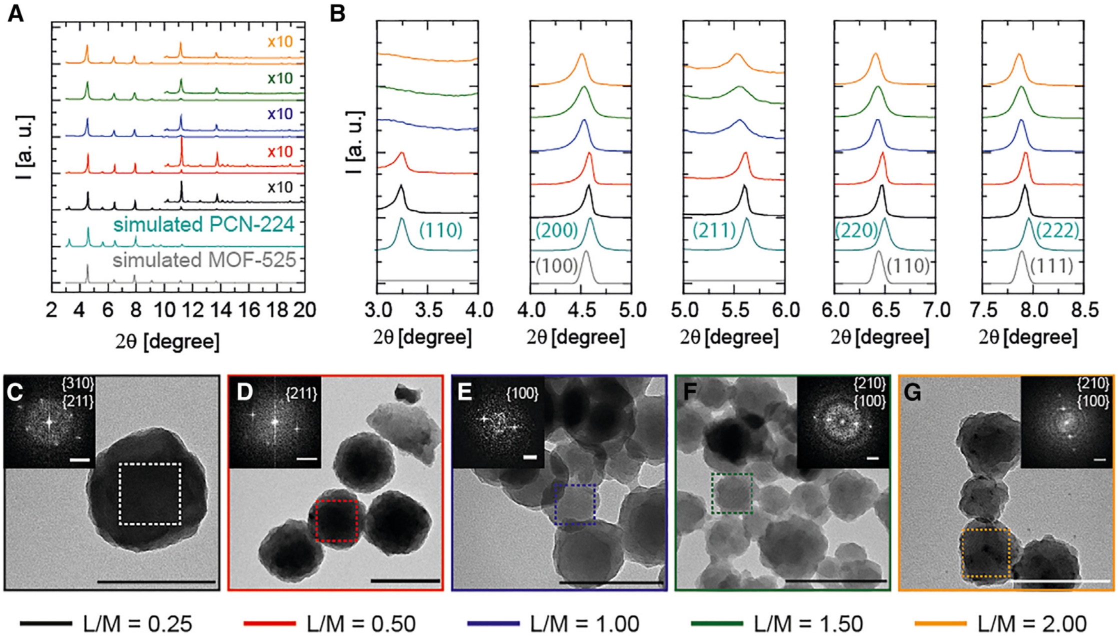

Figure 1. Structural characterization of nanoMOFs obtained by varying the L/M ratio at room temperature

(A and B) PXRD diffractogram with the simulated patterns of PCN-224 and MOF-525 (A) and normalized first 5 diffraction peaks (B).

(C–G) HR-TEM images of porphyrinic MOF nanoparticles changing the linker-to-metal (L/M) ratios (C) 0.25, (D) 0.50, (E) 1.00, (F) 1.50, and (G) 2.00 and their corresponding FFT, indexing some different crystallographic planes (PCN-224 for L/M ratios 0.25 and 0.50 and MOF-525 for L/M ratios 1.00, 1.50, and 2.00). Scale bars represent 200 nm for HR-TEM images and 0.5 nm^{-1} for FFT images.

line) narrows the Soret band and decreases Q-band scattering. Higher L/M ratios (1.00, 1.50, and 2.00) further narrow the Soret band and eliminate scattering in the 700–1,000 nm range, indicating reduced polydispersity and smaller nanoMOF sizes. Similarly, dynamic light scattering (DLS) data of colloidal dispersions of the purified solids presented in Figure S8B show a decrease in particle size and polydispersity with higher L/M ratios. Initial ratios (0.25 and 0.50) resulted in relatively large, polydisperse nanoMOFs, while higher ratios yielded smaller, highly monodisperse nanoMOFs. Details on hydrodynamic sizes and the corresponding polydispersity index (PdI) are provided in Table S8. Most notably, the chosen synthesis conditions achieved remarkably high reaction yields in terms of Zr consumed, ranging from ~80% to nearly 100% for L/M ratios of 0.50 and 1.00, as determined by inductively coupled plasma optical emission spectroscopy (ICP-OES) measurements of Zr content post-purification of the solids (Figure S8C).

Vibrational spectroscopy techniques, including Fourier transform infrared spectroscopy (FTIR spectroscopy) and Raman, revealed no significant differences among the samples (Figures S9 and S10). However, differential scanning calorimetry (DSC) analysis produced two distinct sets of thermograms (Figure S11). Specifically, the samples with larger L/M ratios (1.00, 1.50, and 2.00) exhibited a single endothermic peak around 140°C. In contrast, the samples with lower L/M ratios (0.25 and 0.50) demonstrated three endothermic peaks, with the most intense peaks occurring at 260°C and 250°C, respectively. These findings align with results from previous characterization techniques, indicating that the samples with lower L/M ratios (0.25 and 0.50) differ from those with higher L/M ratios (1.00, 1.50, and 2.00).

The emission spectra of all prepared samples exhibit two bands when excited at both their Soret band (excitation at 420 nm) and Q band (excitation at 515 nm) regions: a first band located at ~650 nm and a second band at around 720 nm, assigned to Q_{00} and Q_{01} transitions, respectively. The normalized emission spectra of the porphyrinic MOFs (Figure S12) demonstrate consistent emission behavior, regardless of excitation at the Soret or Q band. As displayed by TCPP emission, Q_{01} is typically less intense than the Q_{00} band because it involves vibrational excitation in the ground state, making it an overtone transition. However, as the ratio of the components (referring to the L/M ratio) increases, the intensity of the Q_{01} emission band (~720 nm) over the Q_{00} emission band (~650 nm) coherently increases. This suggests that higher L/M ratios enhance the electronic environment, favoring changes in oscillator strength due to an increased incorporation of coordinated TCPP molecules. This change potentially leads to enhanced electronic coupling, altered vibrational relaxation pathways, and variations in structural rigidity and symmetry, affecting the radiative transitions of the porphyrinic system. Moreover, the increase in TCPP coordination number from 6-c (L/M = 0.25) to 12-c (L/M = 2.00) results in a different spatial arrangement and orientation of the porphyrin units within the MOF framework. Such changes in dihedral angles and increased structural rigidity modify the electronic environment, leading to variations in the emission spectra akin to those observed in simpler porphyrin aggregates.

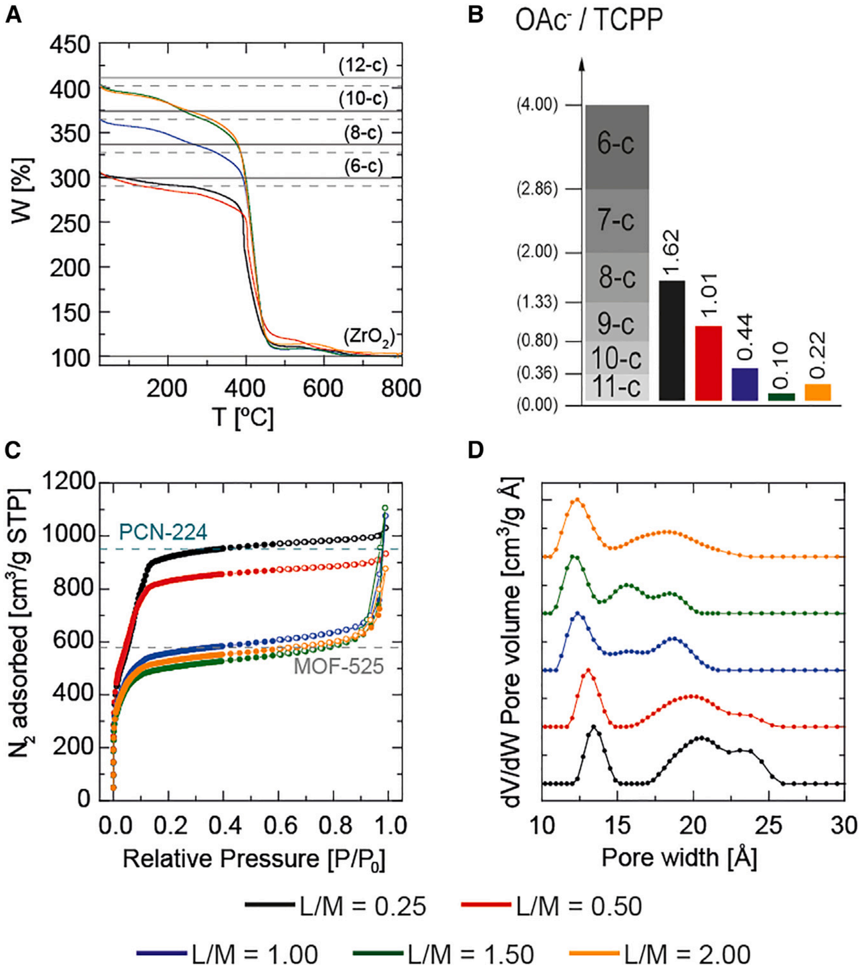

Figure 2. Characterization of nanoMOFs obtained by varying the L/M ratio at room temperature

(A) Normalized TGA with the theoretical mass molar of Zr_6-oxo cluster with different coordination degrees with TCPP and acetate molecules (dashed lines are the dehydrated cluster).

(B) Quantification of TCPP molecules by ^{1}H-NMR.

(C) N_2 adsorption isotherms at 77 K correspond to the expected values for ideal PCN-224 and MOF-525 phases calculated with Zeo++.[37]

(D) Pore size distribution using NLDFT model.

Effect of temperature and alkoxide precursor variation on PCN-224 synthesis

Once confirmed that using the $Zr(OEt)_4$ at RT enables the production of single-phase polydisperse (PdI > 0.3) PCN-224 and monodisperse (PdI < 0.1) MOF-525 nanocrystals, we decided to explore new synthetic conditions to reduce the polydispersity of nanosized PCN-224. Aiming to produce PCN-224 nanocrystals with sizes around 100 nm and low polydispersity, as in the previous section, we set the L/M ratio to 0.25, fixed the reaction time at 1 h, and used AA as the modulator. However, we substantially decreased the Mod/M ratio to 250, aiming to reduce the nanocrystal size, and varied the temperature (Figures S13 and S14). To this end, we explored five temperature settings ranging from RT to 120°C, the latter being widely used to produce MOFs based on the Zr_6-oxo cluster. As anticipated from Figure S13, relatively monodisperse nanoparticles are obtained across the entire temperature range, with their size increasing as the temperature rises, with particle size ranging from 50 to 200 nm.

By examining the reaction yields in terms of Zr consumed (Figure S14A), we observed that when moving from RT to 75°C, the reaction yield moved from 50% to 85% of added Zr, as quantified by ICP-OES (Table S9). Additionally, we noted that increasing the temperature led to subtle changes in the optical properties. Specifically, there was a progressive broadening of the Soret band and an enhancement of the scattering baseline observed (Figure S14B). These changes are attributed to an increase in particle size and polydispersity, as qualitatively confirmed through scanning electron microscopy (SEM) (Figure S13) and DLS in Figure S14C and Table S10.

To go one step further and analyze the impact of different alkoxide precursors in the reaction that allow us to produce nanoMOFs (Figures 3A and 3B), we decided to fix the temperature at 75°C, keep the rest of the parameters unchanged, and employ other Zr precursors besides the ethoxy, such as $Zr(OiPr)_4$ and $Zr(OBut)_4$ (Figures S15 and S16; Table S11). We confirmed the crystalline structure of these solids through PXRD analysis, which revealed that the use of different Zr precursors led to the formation of virtually the same crystalline phase (Figure 3C). Moreover, HR-TEM confirmed the crystallinity of all nanoMOFs (Figure 3B) obtained, as can be seen with FFT analysis on designated ROIs (dashed ROI), providing a critical d-spacing of 16.528 Å compatible with the {211} family of planes for PCN-224, which are not present in MOF-525 or dPCN-224 due to the inherent symmetry in MOF-525 or forbidden reflections resulting from the disorder in the orientations of Zr_6-oxo clusters in dPCN-224; these features were not observed in the PXRD pattern (Figure 3C). Although visual inspection of PXRD analysis does not unequivocally differentiate between PCN-224, MOF-525, and dPCN-224,[14] our results align with the initial expectations of using an L/M ratio of 0.25 (Figure S17). Pawley refinement supports the presence of the PCN-224 phase, reinforcing the interpretation of the normalized TGA thermograms (Figure 3D). This is further evidenced by the exclusive presence of the {211} family of planes in PCN-224. Additionally, the absence of supercell peaks at 3.2° and 5.6° two-theta can be attributed to disorder in defects and/or variations in cluster orientation compared to the ordered structure of PCN-224, which gives the disordered phase dPCN-224 as a result.[14]

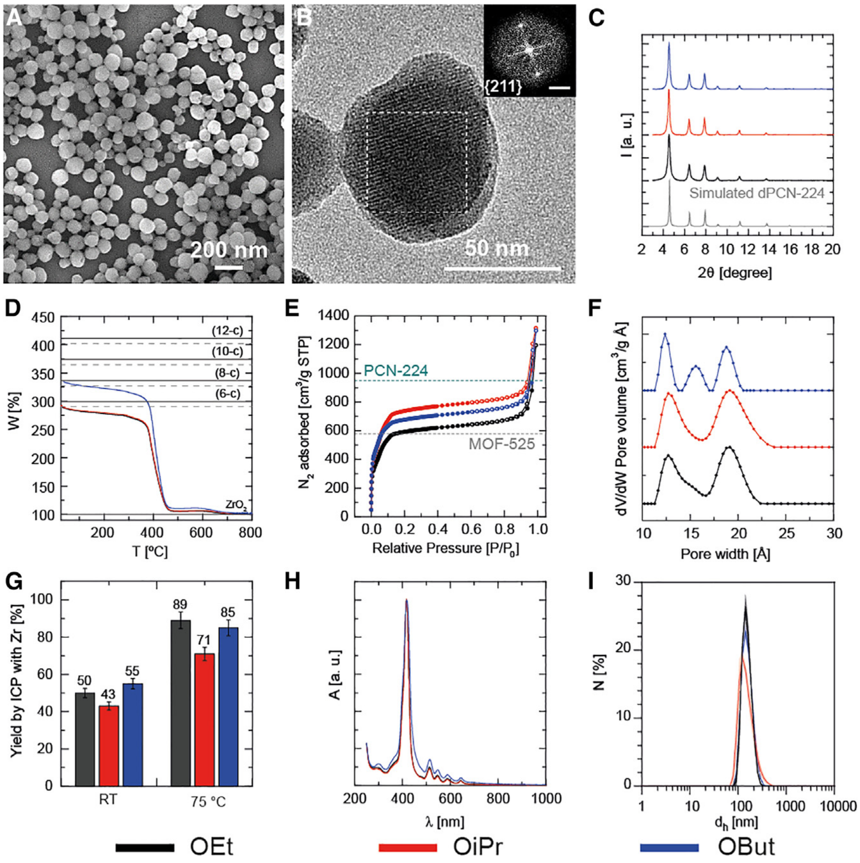

Figure 3. Characterization of nanosized PCN-224: Effect of temperature and alkoxide in the synthesis

(A and B) Representative SEM (A) and HR-TEM (B) with FTT images of nanoMOFs synthesized starting from $Zr(OEt)_4$ precursor at 75°C for 1 h in the presence of AA.

(C) PXRD patterns.

(D) Normalized TGA with the theoretical molar mass of Zr_6-oxo cluster with different coordination degrees with TCPP and acetate molecules (dashed lines are the dehydrated cluster).

(E) N_2 adsorption isotherms at 77 K with the expected uptake values for ideal PCN-224 and MOF-525 phases calculated with Zeo++.[37]

(F) NLDFT pore size distribution by using different Zr precursors.

(G) Graphical representation of the reaction yields evaluated through ICP-OES of 1 h reaction conducted at RT and 75°C.

(H and I) UV-vis extinction spectra (H) and hydrodynamic diameters d_h (by number) (I). Scale in FFT represents 0.5 nm^{-1}.

N_2 adsorption isotherms of the materials revealed the microporosity of the nanoMOFs, with BET surface areas reaching values between 2,600 and 3,000 m^2/g, closely aligning with expectations for PCN-224 (Figures 3E; Table S12).[10] The pore size distribution analysis (Figure 3F), regardless of the alkoxy precursor used, revealed the presence of two primary pore sizes, centered at ~13 and ~19 Å (Figure S7; Table S7) with an extra pore size around 15 Å. This distribution is more closely aligned with the characteristics of PCN-224 rather than those of MOF-525. The presence of some degree of defectivity in our samples was confirmed by TGA, which nevertheless showed cluster connectivity indexes compatible with PCN-224 (6-c) but far from that of MOF-525 (12-c) (Figure 3D).

The 1 h reaction conducted at 75°C displayed high reaction yields (>70%) with all the alkoxide precursors (Figure 3G; Table S13).

In all cases, the characteristic optical signature of porphyrin nanoMOFs could be clearly observed (Figure 3H), with a dominating, very narrow Soret band (at ~420 nm) that is significantly more intense compared to the weaker Q bands (500–630 nm), imparting to the colloidal solution a purple/violet color (Figure S14D) easily distinguishable with respect to the starting red TCPP solution. All the produced nanoMOFs presented superimposable extinction spectra, suggesting similar outcomes despite the different Zr precursors. The average d_h (hydrodynamic diameter by number distribution) was about 150 nm, with a relatively low PdI (<0.15), demonstrating the uniformity in particle d_h (Figure 3I; Table S14).

For completeness, we assessed the impact of temperature on the crystallinity and particle size of the nanoMOFs and conducted the reactions with the three different alkoxy precursors at RT (Figures S18 and S19; Table S15). Under these conditions, the 1 h reaction reached a yield of 50%, regardless of the alkoxide used (Figure 3G; Table S16). While the optical properties were comparable to those of particles synthesized at 75°C (Figure S20A), a notable reduction in d_h to ~100 nm was observed (Figure S20B; Table S17). Most notably, the TGA thermograms closely resemble those obtained at 75°C, even when using $Zr(OBut)_4$ (Figure S20C), which we tentatively associate with the presence of missing cluster defects. Moreover, the crystalline phase of the systems synthesized at RT was similar to that of the solids obtained at 75°C (Figure 3C), consistent with dPCN-224 (Figure S20D), but with considerably lower crystallinity as expected due to the low temperature. However, a pronounced decrease in BET surface areas was observed (Figures S20E and S20F).

Aiming to improve the reaction yield, the RT reaction was extended to 24 h. As anticipated, this adjustment led to an increase in reaction yields up to 70% (Table S16) without compromising the optical properties and crystalline phase of the synthesis conducted at 75°C (Figures S21A and S21B). Despite these adjustments, there was no significant enhancement in the BET surface areas, which remained indicative of a semi-condensed phase (Figures S21C and S21D; Table S18), echoing the outcomes observed with the 1 h of reaction.

Effect of modulator on PCN-222 synthesis

Despite PCN-222 sometimes being found during zirconium halide synthesis of Zr-oxo MOFs, this phase was systematically absent during all our previous experiments. It has been

previously reported that subtle changes, not only in reaction conditions but also in the modulator used, can lead to substantially different phase proportions in Zr-oxo MOFs.[43] To demonstrate the capability of introducing an additional phase through minor modifications in the experimental setup, we substituted the AA modulator with FA and systematically varied the L/M (0.35–1.5) and Mod/L (100, 250, and 560) ratios, the temperature (25°C–75°C), and the Zr precursor (OEt, OiPr, or OBut).

Conducting the reaction at RT for 24 h with any of the Zr precursors at L/M = 0.35, the ideal L/M ratio for PCN-222, and Mod/M = 100 did not yield a solid product. However, at 75°C, solid particles were formed after 24 h (Figure S22A), with almost quantitative yields observed thereafter (Figure S22B; Table S19). The particles exhibited an elongated morphology (Figures S23 and S24; Table S20), suggesting a crystalline phase distinct from those observed in previous sections. Figure S22F presents PXRD diffractograms of Zr-porphyrinic nanocrystals synthesized with different Zr alkoxides and shows that the three samples comprise a phase mixture of MOF-525 and a partially formed phase PCN-222, as corroborated by Pawley refinement (Figure S25), which agrees with TGA showing a plateau slightly above the 8-c line (Figure S22E). Although this refinement does not allow for the quantification of each phase, it is clear that $Zr(OEt)_4$ predominantly favors the formation of PCN-222, evident from the intensity of its reflections (Figure S25A) and the predominantly elongated particle morphology (Figure S23A). This contrasts with $Zr(OiPr)_4$ and $Zr(OBu)_4$, where PXRD patterns indicate a greater presence of the MOF-525 phase (Figures S25B and S25C), aligning with FE-SEM images that show more pseudospherical particles (Figures S23B and S23C), all of them with sizes around 150 nm (Table S21) and BET surface areas below the canonical PCN-222 phase (Table S22). In the case of $Zr(OEt)_4$, with an increase in the L/M ratio, there was a gradual transition toward predominantly pseudospherical particles, indicating a loss of the elongated shape characteristic of PCN-222. Conversely, with a Mod/M ratio of 560 (Figure S26), an increase in the L/M ratio noticeably favored the formation of rod-like shaped particles (Figure S27). Particularly at an L/M ratio of 1.5, there was a marked shift toward the exclusive presence of elongated nanocrystals, highlighting a clear correlation between the Mod/M ratio, the L/M ratio, and the resulting particle morphology (Figure S27).

Encouraged by the promising outcomes, we undertook a comprehensive characterization of the system with L/M = 1.5 and Mod/M = 560, detailed in Figure 3. Further structural insights were obtained from PXRD analysis (Figure 4A), wherein our sample's diffractogram was compared against simulated patterns for PCN-222. Pawley refinement (Figure S28) facilitated the determination of lattice parameters as a = b = 41.905 Å and c = 17.206 Å. These measurements align closely with the established cell parameters for PCN-222, a = b = 41.968 Å and c = 17.143 Å, within the P6/mmm space group, underscoring the precision of our synthetic approach. This was further confirmed by HR-TEM analysis, and after applying the FFT to the selected region (indicated by a dashed line), the d-spacings corresponding to the crystalline planes were determined (Figure S22A). Specifically, d-spacings were found to be 8.620 Å for the {002} family of planes and 11.600 Å for the {300} family of planes. These measurements are indicative of the hexagonal phase of PCN-222.

TGA (Figure 4B) indicated a coordination of ~8 TCPP molecules per Zr_6-oxo cluster, which agrees with the connectivity of the canonical PCN-222 phase. Moreover, the BET surface area measurement presented in Figure 4C, 2,142 m^2/g, along with a pore size distribution featuring diameters of 1.2 and 3.3 nm (Table S7; Figure S7), closely mirrors the reported values for PCN-222.[44] These figures closely match the reported values for canonical PCN-222, validating the successful synthesis and characterization of the material and its compliance with the anticipated structural and physicochemical properties of PCN-222.

The FE-SEM images (Figures 4D and 4E) consistently revealed the presence of elongated nanoparticles across the sample, with the particles displaying characteristic optical properties of nanoMOFs (Figure S22C) and a d_h of ~150 nm (Figure S22D; Tables S20 and S21), highlighting the unique morphology achieved under these conditions.

Interlaboratory reproducibility study

As previously discussed, achieving reproducibility in the synthesis of porphyrinic Zr-based MOFs has posed significant challenges.[21] To validate the reproducibility of our synthesis method, the specific conditions from each experimental section were replicated by another research group using $Zr(OEt)_4$ as the precursor. The porphyrinic Zr-MOF structure, as outlined in Table S2, was confirmed through PXRD analysis of PCN-224, which showed no supercell peaks (Figure S29A), consistent with the findings in Figure 2C. Similarly, the structure in Table S1, which used an L/M ratio of 0.25, exhibited distinct supercell peaks of PCN-224 (Figure S29B), in alignment with the data shown in Figures 1A and 1B. Moreover, the PCN-222 structure from Table S3 using an FA/Zr ratio of 560 was analyzed and confirmed (Figure S29C), validating the results in Figure 3A. These interlaboratory findings further strengthen the reproducibility of our synthesis method using Zr-alkoxide precursors.

Continuous-flow reaction

Batch processes have traditionally been the mainstay for producing MOFs.[45–47] These conventional methods are noted for their high costs, limited scalability, and prolonged durations, which can range from several hours to days.[48–51] Despite progress in scaling up MOF synthesis, industrial production still faces significant challenges.[52] Consequently, there is a pressing need for more efficient MOF preparation methods. Continuous processing has been recognized for significantly improving space-time yields (STYs) under analogous reaction parameters.[53,54] Recent advancements in continuous synthesis include techniques such as spray drying,[55–57] mechanochemical,[58,59] flow methods,[60–66] and microwave-[53,67] and sonication-assisted methods,[68] along with the use of supercritical fluids,[66] among others.[69,70] Notably, microfluidic and millifluidic technologies offer rapid mixing, enhanced heat transfer and energy efficiency, precise control over reaction parameters, and the capability to produce various configurations of MOF composites.[62,71,72]

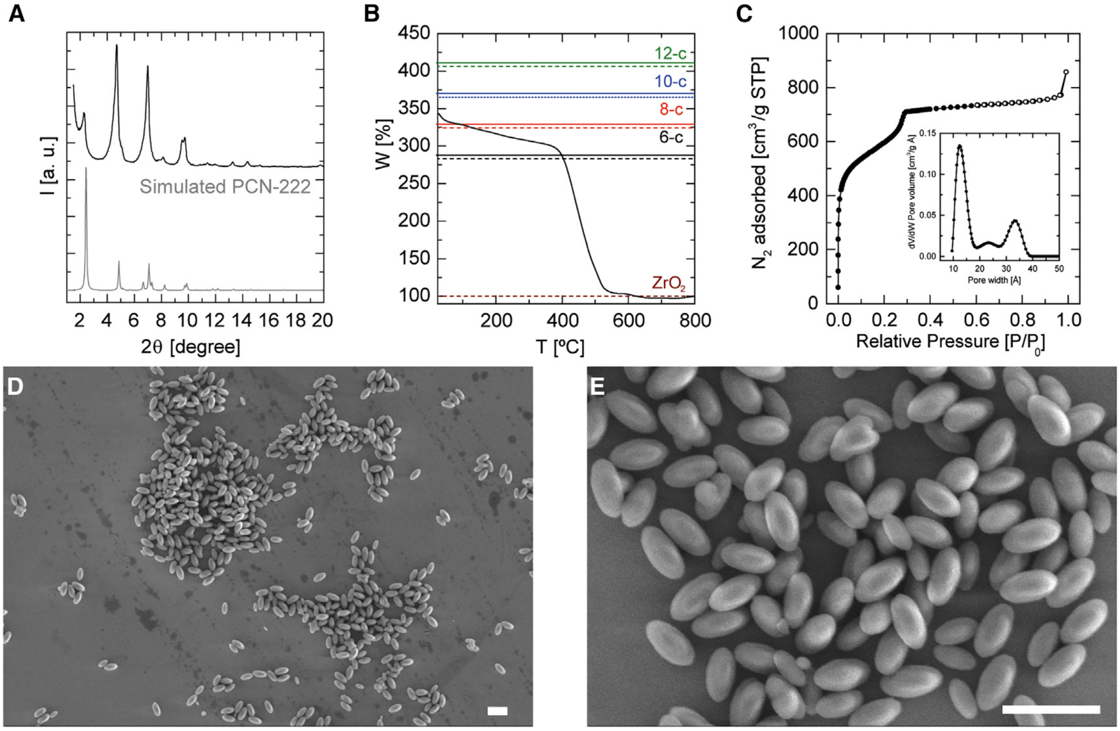

Figure 4. Characterization of porphyrinic MOF nanoparticles using $Zr(OEt)_4$ as precursor and FA as modulator (L/M = 1.5 and Mod/M = 560) at 75°C for 1 h

(A) PXRD diffractogram.

(B) Normalized TGA with theoretical lines of defects for Zr_6-oxo cluster (dashed lines are the dehydrated cluster).

(C) N_2 adsorption isotherm at 77 K (with pore size distribution plot inserted using NLDFT model).

(D and E) FE-SEM images at (D) low and (E) high magnification of PCN-222 nanoMOFs. Scale bars represent 1 μm.

A few studies have explored continuous-flow synthesis for Zr-MOFs, utilizing high temperatures that incur elevated operating costs.[73,74] In contrast, as demonstrated in this work, using alkoxide precursors enables the ultrafast (second-scale) production of nanosized Zr-based porphyrinic MOFs at RT. Notably, traditional precursors like $ZrCl_4$ and $ZrOCl_2$ remained unreacted after 1 h at RT, as shown in Figure S30.

To showcase this method's potential due to the high surfaces areas and catalytic activities of Zr-porphyrinic MOFs,[7–10] continuous synthesis was carried out using a homemade flow reactor with two syringes, one with $Zr(OEt)_4$ dissolved in a mixture of HOAc and DMF and the other with TCPP ligand dissolved in DMF. Both reagents were infused through hoses with an internal diameter of 1.8 mm until they mixed in a Y-shape tube with a resilience time of 25 s, as detailed in Figure S31.

Satisfyingly, PXRD analysis of the resulting sample confirmed that the RT, 20 s residence time reaction successfully produces a pure and highly crystalline phase of PCN-224, with a cell parameter a = 38.4084 Å following Pawley refinement (Figure 5A and S32). SEM images showed pseudospherical nanoparticles of 100–200 nm (Figure 5B). These findings align with the UV-vis spectra, which display the characteristic optical fingerprint of porphyrin MOFs, including a broad Soret band (Figure 5C). The d_h determined by DLS was ~200 nm (Figure 5D). TGA, normalized to the inorganic residue (ZrO_2) set at 100%, is presented in Figure 5E and confirms the 6-c expected for PCN-224. Notably, the microporosity of PCN-224 remained high under rapid flow synthesis, with a BET surface area of 2,387 m^2/g and a pore size distribution (Figure 5F, inset graph) reminiscent of that of nanosized PCN-224 crystals.

This proof-of-concept experiment underscores the feasibility of synthesizing PCN-224 at RT within seconds, highlighting the process efficiency. To evaluate the synthesis efficiency in the continuous-flow regime, the STY and the surface area production rate (SAPR), based on the Zr ICP-OES yield (7.8% ± 0.4%; 9.2 mg), were calculated. The resulting values, 78,122 kg m^{-3} day^{-1} for the STY and 1.9 × 10^{11} m^2 m^{-3} day^{-1} for the SAPR, demonstrate a high-quality and rate of production of PCN-224 by the continuous multifluidic method, showing substantial potential for industrial application.

This study introduces an innovative approach using Zr(IV) alkoxides to facilitate the synthesis of porphyrinic nanoMOFs with remarkably high efficiency and speed. By utilizing Zr-alkoxide precursors, we have demonstrated the capability to rapidly produce nanosized MOF-525, PCN-224, and PCN-222 within minutes, achieving high yields and controlled phase purity under mild conditions. This method significantly surpasses traditional Zr precursors, which typically require longer reaction times and higher temperatures and yield less favorable outcomes, thereby addressing a major bottleneck in the large-scale production of MOFs.

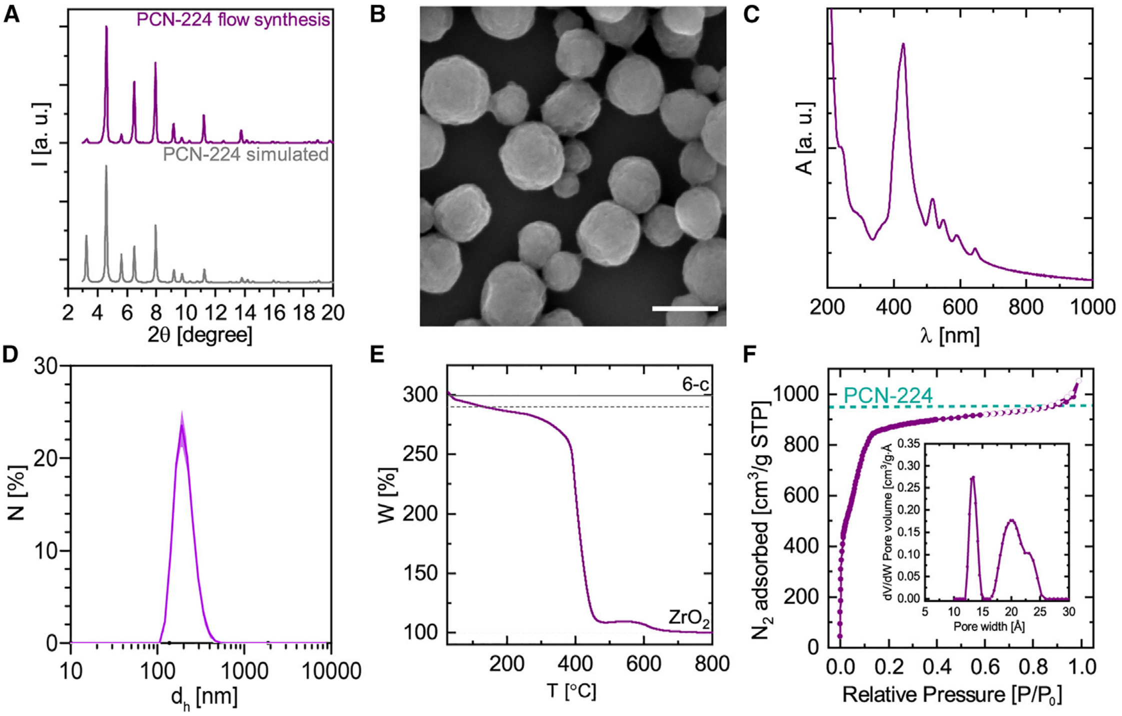

Figure 5. Continuous synthesis of nanosized PCN-224 using a homemade flow reactor

(A) PXRD diffractogram with the simulated pattern of PCN-224.

(B) Representative SEM image of PCN-224 synthesized by continuous-flow synthesis.

(C) UV-vis extinction spectra.

(D) d_h (by number).

(E) Normalized TGA showing the theoretical lines for 6-c Zr_6-oxo cluster (solid line hydrated and dashed line dehydrated cluster).

(F) N_2 adsorption isotherms at 77 K correspond to the expected values for ideal PCN-224 phases calculated with Zeo++.[37] Inset: pore size using NLDFT model. Scale bar represents 200 nm.

By tuning the L/M and Mod/M ratios, as well as the modulator, we have gained precise control over the crystal phases. This advancement is crucial for understanding the factors that facilitate the production of specific phases, which has recently been a subject of controversy.

Furthermore, the successful synthesis of nanosized PCN-224 using a continuous multifluidic method at RT represents a significant leap forward. Our approach not only simplifies the MOF production process but also paves the way for the integration of MOFs into various applications, offering a scalable and economically viable option. The high surface areas and biocompatibility of these materials underscore their immense potential across a broad spectrum of technological and medical applications, promising to overcome previous limitations in MOF production. This work sets a new benchmark for the synthesis of porphyrinic MOFs and opens the door to their widespread industrial and scientific use.

EXPERIMENTAL PROCEDURES

Detailed information on the characterization techniques utilized in this study is provided in the supplemental information. Comprehensive descriptions of all synthetic protocols, including reagent specifications, are also included in the supplemental information. The variations in linker-to-metal (L/M) ratios for synthesizing MOF-525, dPCN-224, and PCN-224, as well as the influence of different modulators on the formation of PCN-222 and the continuous-flow reaction conditions for obtaining PCN-224, are extensively documented in the supplemental information. Additionally, the digestion protocol used for ^{1}H-NMR quantification is fully outlined therein.

RESOURCE AVAILABILITY

Lead contact

Further information and requests for resources should be directed to and will be fulfilled by the lead contact, Pablo del Pino (pablo.delpino@usc.es).

Materials availability

This study did not generate new unique reagents or materials.

Data and code availability

The data underlying this study are available in the article and supplemental information or from the lead contact upon request.

ACKNOWLEDGMENTS

The authors are thankful for the financial support of the European Research Council (starting grant #950421), the European Union (European Union NextGeneration EU/PRTR; H2020-MSCA-ITN #860942), the MICIU/AEI/10.13039/501100011033 (PID2023-152844NB-I00, PID2022-142338OB-100, and PID2020-119206RB-I00), and the Xunta de Galicia (#ED431C 2022/18, #ED431B2023/19, and Centro de Investigación do Sistema Universitario de Galicia accreditation 2023-2027 #ED431G 2023/03). J.M.V.-F. acknowledges

the Spanish Ministry of Science and Innovation for his postdoctoral grant (IJC2020-044369-I). The authors are grateful for the use of RIAIDT-USC analytical facilities, particularly to Bruno Da Cuña Mariño (UNIDADE DE DIFRACCIÓN DE RAIOS X).

AUTHOR CONTRIBUTIONS

M.C., G.Z., O.S., S.F.-H., and S.M.-G. conducted the experiments. J.M.V.-F. performed the TEM characterization. M.C., S.T., C.M.-G., T.D., B.P., and P.d.P. designed the experiments and wrote the paper. B.P. and P.d.P. secured the funding necessary for this work. The manuscript was written with contributions from all authors, and all authors have approved the final version.

DECLARATION OF INTERESTS

The authors declare no competing interests.

SUPPLEMENTAL INFORMATION

Supplemental information can be found online at https://doi.org/10.1016/j.xcrp.2024.102318.

Received: September 10, 2024
Revised: November 3, 2024
Accepted: November 13, 2024
Published: December 11, 2024

REFERENCES

1. Zhang, X., Wasson, M.C., Shayan, M., Berdichevsky, E.K., Ricardo-Noordberg, J., Singh, Z., Papazyan, E.K., Castro, A.J., Marino, P., Ajoyan, Z., et al. (2021). A historical perspective on porphyrin-based metal–organic frameworks and their applications. Coord. Chem. Rev. *429*, 213615.
2. Zhang, X., Tong, S., Huang, D., Liu, Z., Shao, B., Liang, Q., Wu, T., Pan, Y., Huang, J., Liu, Y., et al. (2021). Recent advances of Zr based metal organic frameworks photocatalysis: Energy production and environmental remediation. Coord. Chem. Rev. *448*, 214177.
3. Ceballos, M., Cedrún-Morales, M., Rodríguez-Pérez, M., Funes-Hernando, S., Vila-Fungueiriño, J.M., Zampini, G., Navarro Poupard, M.F., Polo, E., del Pino, P., and Pelaz, B. (2022). High-yield halide-assisted synthesis of metal-organic framework UiO-based nanocarriers. Nanoscale *14*, 6789–6801.
4. Nam, D., Kim, J., and Choe, W. (2023). Evolution of Zr nodes in metal–organic frameworks. Trends Chem. *5*, 339–352.
5. Tatay, S., Martínez-Giménez, S., Rubio-Gaspar, A., Gómez-Oliveira, E., Castells-Gil, J., Dong, Z., Mayoral, Á., Almora-Barrios, N., M. Padial, N., and Martí-Gastaldo, C. (2023). Synthetic control of correlated disorder in UiO-66 frameworks. Nat. Commun. *14*, 6962.
6. Bobbitt, N.S., Shi, K., Bucior, B.J., Chen, H., Tracy-Amoroso, N., Li, Z., Sun, Y., Merlin, J.H., Siepmann, J.I., Siderius, D.W., and Snurr, R.Q. (2023). MOFX-DB: An Online Database of Computational Adsorption Data for Nanoporous Materials. J. Chem. Eng. Data *68*, 483–498.
7. Morris, W., Volosskiy, B., Demir, S., Gándara, F., McGrier, P.L., Furukawa, H., Cascio, D., Stoddart, J.F., and Yaghi, O.M. (2012). Synthesis, structure, and metalation of two new highly porous zirconium metal-organic frameworks. Inorg. Chem. *51*, 6443–6445.
8. Feng, D., Gu, Z.Y., Li, J.R., Jiang, H.L., Wei, Z., and Zhou, H.C. (2012). Zirconium-metalloporphyrin PCN-222: Mesoporous metal-organic frameworks with ultrahigh stability as biomimetic catalysts. Angew. Chem., Int. Ed. Engl. *51*, 10307–10310.
9. Chen, Y., Hoang, T., and Ma, S. (2012). Biomimetic catalysis of a porous iron-based metal-metalloporphyrin framework. Inorg. Chem. *51*, 12600–12602.
10. Feng, D., Chung, W.C., Wei, Z., Gu, Z.Y., Jiang, H.L., Chen, Y.P., Darensbourg, D.J., and Zhou, H.C. (2013). Construction of ultrastable porphyrin Zr metal-organic frameworks through linker elimination. J. Am. Chem. Soc. *135*, 17105–17110.
11. Jiang, H.L., Feng, D., Wang, K., Gu, Z.Y., Wei, Z., Chen, Y.P., and Zhou, H.C. (2013). An exceptionally stable, porphyrinic Zr metal-organic framework exhibiting pH-dependent fluorescence. J. Am. Chem. Soc. *135*, 13934–13938.
12. Deria, P., Gómez-Gualdrón, D.A., Hod, I., Snurr, R.Q., Hupp, J.T., and Farha, O.K. (2016). Framework-Topology-Dependent Catalytic Activity of Zirconium-Based (Porphinato)zinc(II) MOFs. J. Am. Chem. Soc. *138*, 14449–14457.
13. Feng, D., Gu, Z.Y., Chen, Y.P., Park, J., Wei, Z., Sun, Y., Bosch, M., Yuan, S., and Zhou, H.C. (2014). A highly stable porphyrinic zirconium metal-organic framework with shp-a topology. J. Am. Chem. Soc. *136*, 17714–17717.
14. Koschnick, C., Stäglich, R., Scholz, T., Terban, M.W., von Mankowski, A., Savasci, G., Binder, F., Schökel, A., Etter, M., Nuss, J., et al. (2021). Understanding disorder and linker deficiency in porphyrinic zirconium-based metal–organic frameworks by resolving the Zr_8O_6 cluster conundrum in PCN-221. Nat. Commun. *12*, 3099.
15. Feng, D., Jiang, H.L., Chen, Y.P., Gu, Z.Y., Wei, Z., and Zhou, H.C. (2013). Metal-organic frameworks based on previously unknown Zr8/Hf 8 cubic clusters. Inorg. Chem. *52*, 12661–12667.
16. Wei, Y.J., Li, J., Hu, Z.E., Xing, X., Zhou, Z.W., Yu, Y., Yu, X.Q., Zhang, J., Liu, Y.H., and Wang, N. (2023). A porphyrin-MOF-based integrated nanozyme system for catalytic cascades and light-enhanced synergistic amplification of cellular oxidative stress. J. Mater. Chem. B *11*, 6581–6594.
17. Mo, Q., Zhang, L., Li, S., Song, H., Fan, Y., and Su, C.Y. (2022). Engineering Single-Atom Sites into Pore-Confined Nanospaces of Porphyrinic Metal-Organic Frameworks for the Highly Efficient Photocatalytic Hydrogen Evolution Reaction. J. Am. Chem. Soc. *144*, 22747–22758.
18. Wang, H., Zhu, Q.L., Zou, R., and Xu, Q. (2017). Metal-Organic Frameworks for Energy Applications. Chem *2*, 52–80.
19. Chen, Z., Sun, Y., Wang, J., Zhou, X., Kong, X., Meng, J., and Zhang, X. (2023). Dual-Responsive Triple-Synergistic Fe-MOF for Tumor Theranostics. ACS Nano *17*, 9003–9013.
20. Oh, J.Y., Choi, E., Jana, B., Go, E.M., Jin, E., Jin, S., Lee, J., Bae, J.H., Yang, G., Kwak, S.K., et al. (2023). Protein-Precoated Surface of Metal-Organic Framework Nanoparticles for Targeted Delivery. Small *19*, 2300218.
21. Boström, H.L.B., Emmerling, S., Heck, F., Koschnick, C., Jones, A.J., Cliffe, M.J., Al Natour, R., Bonneau, M., Guillerm, V., Shekhah, O., et al. (2024). How Reproducible is the Synthesis of Zr–Porphyrin Metal–Organic Frameworks? An Interlaboratory Study. Adv. Mater. *36*, 102304832.
22. Koschnick, C., Terban, M.W., Canossa, S., Etter, M., Dinnebier, R.E., and Lotsch, B.V. (2024). Influence of Water Content on Speciation and Phase Formation in Zr–Porphyrin-Based MOFs. Adv. Mater. *36*, 2210613.
23. Taddei, M., Van Bokhoven, J.A., and Ranocchiari, M. (2020). Influence of Water in the Synthesis of the Zirconium-Based Metal-Organic Framework UiO-66: Isolation and Reactivity of $[ZrCl(OH)_2(DMF)_2]Cl$. Inorg. Chem. *59*, 7860–7868.
24. Semivrazhskaya, O.O., Salionov, D., Clark, A.H., Casati, N.P.M., Nachtegaal, M., Ranocchiari, M., Bjelić, S., Verel, R., van Bokhoven, J.A., and Sushkevich, V.L. (2023). Deciphering the Mechanism of Crystallization of UiO-66 Metal-Organic Framework. Small *19*, 2305771.
25. Schubert, U. (2023). En route from metal alkoxides to metal oxides: metal oxo/alkoxo clusters. J. Sol. Gel Sci. Technol. *105*, 587–595.
26. Piszczek, P., Radtke, A., Grodzicki, A., Wojtczak, A., and Chojnacki, J. (2007). The new type of [Zr6(μ3-O)4(μ3-OH)4] cluster core: Crystal structure and spectral characterization of [Zr6O4(OH)4(OOCR)12] (R = But, C(CH3)2Et). Polyhedron *26*, 679–685.

27. Kickelbick, G., Wiede, P., and Schubert, U. (1999). Variations in capping the $Zr_6O_4(OH)_4$ cluster core: X-ray structure analyses of [Zr_6(O-H)$_4O_4$(OOC-CH=CH$_2$) $_{10}$]$_2$(μ-OOC-CH=CH$_2$)$_4$ and $Zr_6(OH)_4O_4(OOCR)_{12}$ (PrOH) (R = Ph, CMe = CH$_2$). Inorg. Chim. Acta. *284*, 1–7.

28. Kickelbick, G., and Schubert, U. (1997). Oxozirconium Methacrylate Clusters: $Zr_6(OH)_40_4(OMc)_{12}$ and $Zr_40_2(OMc)_{12}$ (OMc = Methacrylate). Chem. Berichte-Recueil *130*, 473–477.

29. Guillerm, V., Gross, S., Serre, C., Devic, T., Bauer, M., and Férey, G. (2010). A zirconium methacrylate oxocluster as precursor for the low-temperature synthesis of porous zirconium(iv) dicarboxylates. Chem. Commun. *46*, 767–769.

30. Jerozal, R.T., Pitt, T.A., MacMillan, S.N., and Milner, P.J. (2023). High-Concentration Self-Assembly of Zirconium- and Hafnium-Based Metal-Organic Materials. J. Am. Chem. Soc. *145*, 13273–13283.

31. Ceballos, M., Funes-Hernando, S., Zampini, G., Cedrún-Morales, M., Vila-Fungueiriño, J.M., Pelaz, B., and del Pino, P. (2024). Seeded-Growth of PCN-224 onto Plasmonic Nanoparticles: Photoactive Microporous Nano-carriers. Small Struct. *5*, 2300464.

32. Kubo, M., Miyoshi, Y., Uchitomi, Y., and Shimada, M. (2024). Insights into the Spray Synthesis of UiO-66 and UiO-66-NH2 Metal–Organic Frameworks: Effect of Zirconium Precursors and Process Parameters. Crystals *14*, 116.

33. DeStefano, M.R., Islamoglu, T., Garibay, S.J., Hupp, J.T., and Farha, O.K. (2017). Room-Temperature Synthesis of UiO-66 and Thermal Modulation of Densities of Defect Sites. Chem. Mater. *29*, 1357–1361.

34. Dai, S., Nouar, F., Zhang, S., Tissot, A., and Serre, C. (2021). One-Step Room-Temperature Synthesis of Metal(IV) Carboxylate Metal—Organic Frameworks. Angew. Chem., Int. Ed. Engl. *60*, 4282–4288.

35. Tulig, K., and Walton, K.S. (2014). An alternative UiO-66 synthesis for HCl-sensitive nanoparticle encapsulation. RSC Adv. *4*, 51080–51083.

36. Wang, J., Imaz, I., and Maspoch, D. (2022). Metal−Organic Frameworks: Why Make Them Small? Small Struct. *3*, 2100126.

37. Willems, T.F., Rycroft, C.H., Kazi, M., Meza, J.C., and Haranczyk, M. (2012). Algorithms and tools for high-throughput geometry-based analysis of crystalline porous materials. Microporous Mesoporous Mater. *149*, 134–141.

38. Verma, P.K., Koellner, C.A., Hall, H., Phister, M.R., Stone, K.H., Nichols, A.W., Dhakal, A., Ashcraft, E., Machan, C.W., and Giri, G. (2023). Solution Shearing of Zirconium (Zr)-Based Metal-Organic Frameworks NU-901 and MOF-525 Thin Films for Electrocatalytic Reduction Applications. ACS Appl. Mater. Interfaces *15*, 53913–53923.

39. Chu, J., Ke, F.S., Wang, Y., Feng, X., Chen, W., Ai, X., Yang, H., and Cao, Y. (2020). Facile and reversible digestion and regeneration of zirconium-based metal-organic frameworks. Commun. Chem. *3*, 5.

40. Li, Y., Lo, W.S., Zhang, F., Si, X., Chou, L.Y., Liu, X.Y., Williams, B.P., Li, Y.H., Jung, S.H., Hsu, Y.S., et al. (2021). Creating an Aligned Interface between Nanoparticles and MOFs by Concurrent Replacement of Capping Agents. J. Am. Chem. Soc. *143*, 5182–5190.

41. Osterrieth, J.W.M., Rampersad, J., Madden, D., Rampal, N., Skoric, L., Connolly, B., Allendorf, M.D., Stavila, V., Snider, J.L., Ameloot, R., et al. (2022). How Reproducible are Surface Areas Calculated from the BET Equation? Adv. Mater. *34*, 2201502.

42. Gong, X., Noh, H., Gianneschi, N.C., and Farha, O.K. (2019). Interrogating Kinetic versus Thermodynamic Topologies of Metal-Organic Frameworks via Combined Transmission Electron Microscopy and X-ray Diffraction Analysis. J. Am. Chem. Soc. *141*, 6146–6151.

43. Ma, C., Zheng, L., Wang, G., Guo, J., Li, L., He, Q., Chen, Y., and Zhang, H. (2022). Phase engineering of metal-organic frameworks. Aggregate *3*, e145.

44. Ma, C., Wolterbeek, H.T., Denkova, A.G., and Serra Crespo, P. (2023). Porphyrinic metal-organic frameworks as molybdenum adsorbents for the 99Mo/99mTc generator. Inorg. Chem. Front. *10*, 2239–2249.

45. Polyzoidis, A., Etter, M., Herrmann, M., Loebbecke, S., and Dinnebier, R.E. (2017). Revealing the Initial Reaction Behavior in the Continuous Synthesis of Metal-Organic Frameworks Using Real-Time Synchrotron X-ray Analysis. Inorg. Chem. *56*, 5489–5492.

46. Shukre, R., Ericson, T.E., Unruh, D.K., Harbin, H.J., Cozzolino, A.F., Chen, C.C., and Vanapalli, S.A. (2022). Batch-screening guided continuous flow synthesis of the metal-organic framework HKUST-1 in a millifluidic droplet reactor. Microporous Mesoporous Mater. *339*, 112005.

47. Rasmussen, E.G., Kramlich, J., and Novosselov, I.V. (2022). Synthesis of metal-organic framework HKUST-1 via tunable continuous flow supercritical carbon dioxide reactor. Chem. Eng. J. *450*, 138053.

48. Ma, D., Huang, X., Zhang, Y., Wang, L., and Wang, B. (2023). Metal-organic frameworks: Synthetic methods for industrial production. Nano Res. *16*, 7906–7925.

49. Chen, Y., and Wu, T. (2023). Sustainable and scalable continuous synthesis of metal-organic frameworks for CO_2 capture. Greenhouse Gases. *13*, 409–420.

50. Bailey, T., Pinto, M., Hondow, N., and Wu, K.J. (2021). Continuous microfluidic synthesis of zirconium-based UiO-67 using a coiled flow invertor reactor. MethodsX *8*, 101246.

51. Ren, J., Dyosiba, X., Musyoka, N.M., Langmi, H.W., Mathe, M., and Liao, S. (2017). Review on the current practices and efforts towards pilot-scale production of metal-organic frameworks (MOFs). Coord. Chem. Rev. *352*, 187–219.

52. Dunne, P.W., Lester, E., and Walton, R.I. (2016). Towards scalable and controlled synthesis of metal-organic framework materials using continuous flow reactors. React. Chem. Eng. *1*, 352–360.

53. McKinstry, C., Cussen, E.J., Fletcher, A.J., Patwardhan, S.V., and Sefcik, J. (2017). Scalable continuous production of high quality HKUST-1 via conventional and microwave heating. Chem. Eng. J. *326*, 570–577.

54. Chang, M., Ren, J., Wei, Y., Yan, T., Wang, J.X., Liu, D., and Chen, J.F. (2023). Discovery of a Scalable Metal-Organic Framework with a Switchable Structure for Efficient CH_4/N_2 Separation. Chem. Mater. *35*, 4286–4296.

55. Carné-Sánchez, A., Imaz, I., Cano-Sarabia, M., and Maspoch, D. (2013). A spray-drying strategy for synthesis of nanoscale metal-organic frameworks and their assembly into hollow superstructures. Nat. Chem. *5*, 203–211.

56. Carné-Sánchez, A., Stylianou, K.C., Carbonell, C., Naderi, M., Imaz, I., and Maspoch, D. (2015). Protecting metal-organic framework crystals from hydrolytic degradation by spray-dry encapsulating them into polystyrene microspheres. Adv. Mater. *27*, 869–873.

57. Garzón-Tovar, L., Cano-Sarabia, M., Carné-Sánchez, A., Carbonell, C., Imaz, I., and Maspoch, D. (2016). A spray-drying continuous-flow method for simultaneous synthesis and shaping of microspherical high nuclearity MOF beads. React. Chem. Eng. *1*, 533–539.

58. Crawford, D., Casaban, J., Haydon, R., Giri, N., McNally, T., and James, S.L. (2015). Synthesis by extrusion: Continuous, large-scale preparation of MOFs using little or no solvent. Chem. Sci. *6*, 1645–1649.

59. Zhao, J., and Peng, R. (2020). Gas-Solid Two-Phase Flow Synthesis Equipment: A New Method for Continuous, Large-Scale Preparation of Metal-Organic Frameworks with No Solvent. Ind. Eng. Chem. Res. *59*, 15791–15795.

60. Zhang, M., Yu, Z., Sun, Z., Wang, A., Zhang, J., Liu, Y.Y., and Wang, Y. (2021). Continuous synthesis of ZIF-67 by a microchannel mixer: A recyclable approach. Microporous Mesoporous Mater. *327*, 111423.

61. Wang, Y., Li, L., Yan, L., Cao, L., Dai, P., Gu, X., and Zhao, X. (2018). Continuous synthesis for zirconium metal-organic frameworks with high quality and productivity *via* microdroplet flow reaction. Chin. Chem. Lett. *29*, 849–853.

62. Sun, C., Barton, M., Pask, C.M., Edokali, M., Yang, L., Britton, A.J., Micklethwaite, S., Iacoviello, F., Hassanpour, A., Besenhard, M., et al.

(2023). Droplet-based millifluidic synthesis of a proton-conducting sulfonate metal–organic framework. Chem. Eng. J. *474*, 145892.

63. Fu, Q., Niu, W., Yan, L., Xie, W., Jiang, H., Zhang, S., Yang, L., Wang, Y., Xing, Y., and Zhao, X. (2023). A versatile microfluidic strategy using air–liquid segmented flow for continuous and efficient synthesis of metal–organic frameworks. Mater. Lett. *343*, 134344.
64. Wu, H.Y., Wu, C.L., Liao, W., Matsagar, B.M., Chang, K.Y., Huang, J.H., and Wu, K.C.W. (2023). Continuous and ultrafast MOF synthesis using droplet microfluidic nanoarchitectonics. J. Mater. Chem. A *11*, 9427–9435.
65. Kevat, S., Sutariya, B., and Lad, V.N. (2023). Microfluidics-assisted, time-effective and continuous synthesis of bimetallic ZIF-8/67 under different synthesis conditions. J. Mater. Sci. *58*, 5219–5233.
66. Rasmussen, E.G., Kramlich, J., and Novosselov, I.V. (2020). Scalable Continuous Flow Metal-Organic Framework (MOF) Synthesis Using Supercritical CO_2. ACS Sustain. Chem. Eng. *8*, 9680–9689.
67. Taddei, M., Steitz, D.A., Van Bokhoven, J.A., and Ranocchiari, M. (2016). Continuous-Flow Microwave Synthesis of Metal-Organic Frameworks: A Highly Efficient Method for Large-Scale Production. Chem. Eur J. *22*, 3245–3249.
68. Waitschat, S., Wharmby, M.T., and Stock, N. (2015). Flow-synthesis of carboxylate and phosphonate based metal-organic frameworks under non-solvothermal reaction conditions. Dalton Trans. *44*, 11235–11240.
69. Chang, M., Wei, Y., Liu, D., Wang, J.X., and Chen, J.F. (2021). A General Strategy for Instantaneous and Continuous Synthesis of Ultrasmall Metal–Organic Framework Nanoparticles. Angew. Chem., Int. Ed. Engl. *60*, 26390–26396.
70. Rubio-Martinez, M., Avci-Camur, C., Thornton, A.W., Imaz, I., Maspoch, D., and Hill, M.R. (2017). New synthetic routes towards MOF production at scale. Chem. Soc. Rev. *46*, 3453–3480.
71. Carraro, F., Williams, J.D., Linares-Moreau, M., Parise, C., Liang, W., Amenitsch, H., Doonan, C., Kappe, C.O., and Falcaro, P. (2020). Continuous-Flow Synthesis of ZIF-8 Biocomposites with Tunable Particle Size. Angew. Chem., Int. Ed. Engl. *59*, 8123–8127.
72. Liu, Z., Zhu, J., Peng, C., Wakihara, T., and Okubo, T. (2019). Continuous flow synthesis of ordered porous materials: from zeolites to metal–organic frameworks and mesoporous silica. React. Chem. Eng. *4*, 1699–1720.
73. Zuliani, A., Carmen Castillejos, M., and Khiar, N. (2023). Continuous flow synthesis of PCN-222 (MOF-545) with controlled size and morphology: a sustainable approach for efficient production. Green Chem. *25*, 10596–10610.
74. Polyzoidis, A., Reichle, S., Schwarzer, M., Piscopo, C.G., Löbbecke, S., and Boskovic, D. (2021). Improved continuous synthesis of UiO-66 enabling outstanding production rates. React. Chem. Eng. *6*, 679–684.

Cell Reports Physical Science, Volume *5*

Supplemental information

Ultrafast synthesis of zirconium-porphyrin framework nanocrystals from alkoxide precursors

Manuel Ceballos, Giulia Zampini, Oleg Semyonov, Samuel Funes-Hernando, José Manuel Vila-Fungueiriño, Sonia Martínez-Giménez, Sergio Tatay, Carlos Martí-Gastaldo, Thomas Devic, Beatriz Pelaz, and Pablo del Pino

Ultrafast Synthesis of Zirconium-Porphyrin Framework Nanocrystals from Alkoxide Precursors

Manuel Ceballos,[1] Giulia Zampini,[2] Oleg Semyonov,[2] Samuel Funes-Hernando,[1] José Manuel Vila-Fungueiriño,[3] Sonia Martínez-Giménez,[4] Sergio Tatay,[4] Carlos Martí-Gastaldo,[4] Thomas Devic,[5] Beatriz Pelaz,[6] and Pablo del Pino[1,*]

[1]Centro Singular de Investigación en Química Biolóxica e Materiais Moleculares (CiQUS), Departamento de Física de Partículas, Universidade de Santiago de Compostela, 15782 Santiago de Compostela, Spain.

[2]Centro Singular de Investigación en Química Biolóxica e Materiais Moleculares (CiQUS), Universidade de Santiago de Compostela, 15782 Santiago de Compostela, Spain.

[3]Centro Singular de Investigación en Química Biolóxica e Materiais Moleculares (CiQUS), Departamento de Química Física, Universidade de Santiago de Compostela, 15782 Santiago de Compostela, Spain.

[4]Instituto de Ciencia Molecular (ICMol), Universitat de València, Catedrático José Beltrán-2, Paterna, 46980 Spain.

[5]Nantes Université, CNRS, Institut des Matériaux de Nantes Jean Rouxel, IMN, F-44000, Nantes, France.

[6]Centro Singular de Investigación en Química Biolóxica e Materiais Moleculares (CiQUS), Departamento de Química Inorgánica, Universidade de Santiago de Compostela, 15782 Santiago de Compostela, Spain.

Table of Content

Characterization techniques 2
Materials and Methods 5
- Chemicals 5
- Synthesis of MOF-525, dPCN-224 and PCN-224 5
- Synthesis of PCN-222 7
- Digestion of Zr-porphyrinic MOFs for ^{1}H-NMR quantification 8
- Continuous PCN-224 synthesis procedure 8

Alkoxy (acetic acid) L/M: 0.25 – 2.0 Mod/M = 560 9
EtO (acetic acid) role of the temperature in the reaction yield 18
Alkoxy (acetic acid) 75 °C (1 h): L/M = 0.25 Mod/M = 250 20
Alkoxy (acetic acid) 25 °C (1 h vs 24 h): L/M = 0.25 Mod/M = 250 23
Alkoxy (formic acid) 75 °C 27
Reproducibility test 34
Continuous flow reaction 35
APPENDIX BETSI N_2 adsorption analysis 37
References 56

Characterization techniques

UV-Vis spectroscopy (UV-Vis)

UV-visible extinction spectra ranging from 200 to 1000 nm were recorded using an Agilent Cary 3500 Multicell UV-Vis Spectrophotometer with the nanoparticles dispersed in MeOH. The measurements were performed using a 1 cm quartz cell, using pure MeOH as a blank.

Dynamic Light Scattering (DLS)

The hydrodynamic diameter (D_h) and polydispersity index (PDI) were determined using Dynamic Light Scattering (DLS) with a Malvern Zetasizer Ultra-Red instrument. The DLS measurements were conducted with a 10 mW He-Ne laser operating at a wavelength of 633 nm. Multiple scattering angles were utilized through the application of the Multi-Angle Dynamic Light Scattering (MADLS) technique.

Thermogravimetric analysis (TGA)

Thermogravimetric analysis (TGA) was performed using a TA Instruments Q5000 IR thermobalance. The TGA measurements followed a standard heating profile from 25 to 800 °C, with a heating rate of 5 °C min^{-1} under an air atmosphere and a gas flow rate of 25 mL min^{-1}. The samples were washed with MeOH three times to remove residual non-volatile DMF solvent and subsequently dried at 70 ºC for 24 hours to ensure complete solvent removal prior to measurement.

Differential Scanning calorimetry (DSC)

DSC measurements were performed using a TA Instruments Q200 calorimeter with a flow nitrogen flow rate of 50 mL min^{-1}. Around 0.5-1.0 mg of samples were place in aluminum crucibles hermetically closed and an empty aluminum crucible also hermetically closed were please as reference.

Inductively Coupled Plasma-Optical Emission Spectrometry (ICP-OES)

Elemental analysis was carried out using an Agilent 5800 Inductively Coupled Plasma Optical Emission Spectrometer (ICP-OES) due to its suitability for quantitative elemental analysis. Calibration curves were established with a concentration range of 0 to 10 ppm for Zr. The ICP-OES detector utilized the atomic emission lines of Zr at 339.198 nm and 343.823 nm.

Before analysis, sample preparation involved dilution and acid digestion. Specifically, 100 µL of the sample were diluted into 1.2 mL of nitric acid (67 wt%) and 3.5 mL of hydrochloric acid (37 wt%). Additionally, 100 µL of Mn solution (500 ppm) and 100 µL of Se solution (500 ppm) were added as internal standards. The entire mixture, totaling 5 mL, was transferred

into a Teflon (PTFE-TFM) tube, specifically an HVT50 tube from Anton Paar. The digestion step was carried out using an Anton Paar Multiwave GO Plus microwave heating system at 185 °C for 15 minutes.

Following digestion, 500 µL of the sample were further diluted into 4.5 mL of water, resulting in a final dilution factor of 500x. This diluted solution was then injected into the ICP-OES instrument, and the software provided the results in parts per million (ppm) for Zr concentration.

Scanning Electron Microscopy (SEM)

Microscopy images were acquired with a Zeiss Ultra Plus Field Emission Scanning Electron Microscope (FE-SEM) operating at acceleration voltages of 3 kV. Image acquisition was performed using the InLens detector.

Transmission Electron Microscopy (TEM)

TEM images were captured with a JEOL JEM F200 microscope, which was equipped with a Gatan OneView camera and a cold-field emission gun (FEG) operating at an accelerating voltage of 80 kV. For TEM specimen preparation, a drop of diluted samples was added to a 400-mesh Cu grid and allowed to dry.

Powder X-Ray Diffraction (PXRD)

Crystalline powder underwent Powder X-Ray diffraction (PXRD) analysis at room temperature using a Bragg-Brentano geometry on a "Bruker D8 Advance" X-ray diffractometer (40 kV, 40 mA, θ/θ configuration). The diffractometer was equipped with a sealed Cu X-ray tube ($\lambda_{CuK\alpha1}$ = 1.5406 Å) and a LYNXEYE detector. Diffractograms were generated within the angular range of $3<2\theta<40$, with a step size of 0.02° (2θ) at 2 seconds per step. Throughout the measurement, sample rotation optimized peak profiles for analysis and minimized the impact of preferred orientation. To prevent background noise from a glass support, samples were positioned on a Si(511) oriented crystal base.

Small-angle powder X-Ray Diffraction

Measurements of X-Ray diffraction at small angles were performed in a Malvern Panalytical-Empyrean with five-axis goniometer ("χ-φ-x-y-z stage"), with a sealed Cu tube (λ=0.154nm) and bicap W/Si parallel beam-generating optics with an acceptance angle of 0.8° and a length of 55.3 mm, equipped with an area detector type "PANalytical PIXcel-3D"

Fourier-transformed infrared spectroscopy (FTIR)

FTIR measurements were recorded using a PerkinElmer Spectrum Two spectrometer with Attenuated Total Reflection (ATR). The dried powders were placed on the ATR window for analysis within the wavenumber range of 700 to 4000 cm^{-1}.

N_2 adsorption–desorption analysis

Nitrogen (N_2) adsorption measurements were carried out using a Micromeritics 3Flex Adsorption Analyzer at 77 K. Before analysis, the samples were outgassed at 90ºC under high vacuum overnight. The amount of sample was around 20-30 mg powder. The specific surface area was determined by extrapolating within the relative pressure range of 0.05-0.3 (where P/P_0 represents the ratio of the measured pressure to the saturation pressure) using the Brunauer, Emmett & Teller (BET) equation. Pore size distribution was determined using a NLDFT (Non-local Density Functional Theory) model for Pillared Clay, considering cylindrical pores.

Data analysis was conducted using the 3Flex V5.03 software, developed by Micromeritics Instrument Corp. based in Norcross, GA, United States.

Analysis of BET surface area were performed with BESTI analysis following Rouquerol criteria.[1]

Raman spectroscopy

Raman measurements were conducted using a BWTEK i-RAMAN EX system on a glass substrate, employing a laser beam at 1064 nm and a 50× objective. The samples were exposed to a power of 160 mW with an acquisition time of 5 s and 10 accumulations.

NMR spectroscopy

^1H NMR spectra were obtained at room temperature employing a BRUKER AVIII 500 MHz spectrometer, with a frequency of 500 MHz. d1 = 20 s and 64 scans. The spectra were referenced to the residual solvent peak (D_2O, singlet, 4.80 ppm). Analysis of the spectra was performed using MestreNova© NMR data processing software. Chemical shifts (δ) are reported in ppm.

Photoluminescence spectroscopy (PL)

Photoluminescence (PL) emission spectra were performed using an Edinburgh-FS5-Spectrofluorometer from Edinburgh Instruments Ltd. Steady-state measurements involved the use of a Xenon lamp as the excitation source, and the recorded spectra were corrected for the instrument's response characteristics.

Materials and Methods

Chemicals

Zirconium ethoxide ($Zr(OEt)_4$, 97%), Zirconium(IV) isopropoxide isopropanol complex ($Zr(OiPr)_4$, 99.9% trace metals basis), Zirconium(IV) butoxide solution ($Zr(OBut)_4$, 80 wt. % in 1-butanol), formic acid (FA, ≥96%), Methylsulfonylmethane ($(CH_3)_2SO_2$, Pharmaceutical secondary standard) from Sigma-Aldrich. 5,10,15,20-(Tetra-4-carboxyphenyl)porphyrin (TCPP, 98%) from PorphyChem. N,N-dimethylformamide (DMF ≥99.8%), methanol (MeOH, LC/MS Grade), acetic acid glacial (AA, 99.7%), Sodium hydrogen carbonate ($NaHCO_3$) were purchased from Fischer Scientific. Deuterium oxide (D_2O, 99.8% D atoms, Acros Organics. All the chemical reagents were used without further purification.

Synthesis of MOF-525 and PCN-224

In 2 mL-vials, two solutions (A and B) are separately prepared and then mixed to start the reaction. In one solution it is dissolved the metal precursor (solution A – Zr alkoxide) together with the modulator (acetic acid), and in the other one the linker (solution B – TCPP or Tetrakis(4-carboxyphenyl) porphyrin). The two solutions are prepared in DMF.
The quantities are specified below, depending on the ratio L/M, the Zr precursor and Mod/M adopted.
In a vial, equipped with a magnetic stirrer, the solution B is added and put under magnetic stirring at 500 rpm; then, the solution A is quickly added, the vial is sealed, and the reaction is stirred at 75 °C (or room temperature) for 1 hour in dark condition.
The reaction is then centrifuged at 10000 g for 10 min; the recovered pellet is washed by centrifugation (10000 g, 10 min) 3-times with 1 mL of DMF and 2-times with 1 mL of MeOH. The final sample is resuspended in 1 mL of MeOH.

Used acronyms:

- L: linker (TCPP).
- M: metal precursor ($Zr(OEt)_4$ or $Zr(OiPr)_4$ or $Zr(OBut)_4$).
- Mod: modulator (acetic acid or formic acid).

All reagents were used under standard laboratory conditions without a glovebox, and the DMF was used without prior drying.

Table S1. Adopted quantities for the synthesis of PCN-224 and MOF-525 starting from $Zr(OEt)_4$ precursor.

	Entry	Ratio Mod/M 560				
**Sol. A**	$Zr(OEt)_4$	6.8 mg				
	AA	0.800 mL				
	DMF	0.200 mL				
	**Entry**	**Ratio L/M 0.25**	**Ratio L/M 0.5**	**Ratio L/M 1.0**	**Ratio L/M 1.5**	**Ratio L/M 2.0**
**Sol. B**	TCPP	5.0 mg	10.0 mg	20.0 mg	30.0 mg	40.0 mg
	DMF	1.000 mL	1.000 mL	1.000 mL	1.000 mL	1.000 mL

Table S2. Adopted quantities for the synthesis of dPCN-224 starting from different Zr precursor.

	Entry	Ratio Mod/M 250	Entry	Ratio Mod/M 250	Entry	Ratio Mod/M 250
**Sol. A**	$Zr(OEt)_4$	6.8 mg	$Zr(OiPr)_4$	9.7 mg	$Zr(OBut)_4$	11.5 µL
	AA	0.358 mL				
	DMF	0.642 mL				
	**Entry**	**Ratio L/M 0.25**				
**Sol. B**	TCPP	5.0 mg				
	DMF	1.000 mL				

Synthesis of PCN-222

In 2 mL-eppendorfs, the two solutions A (containing the modulator and the metal precursor) and B (with TCPP linker) are separately prepared and then mixed together to start the reaction.
In this case, the modulator is formic acid and the temperature is fixed at 75 °C.
The quantities specified below are relative to:

- ratio L/M = 0.33 and Mod/M = 100: 24h reaction.
- ratio L/M = 0.35-1.5 and Mod/M = 250 or 560: 1h reaction.

In a vial, equipped with a magnetic stirrer, the solution B is added and put at 75 °C under magnetic stirring at 500 rpm for 10 minutes; then, the solution A is quickly added, the vial is sealed and the reaction is stirred in dark condition for the proper time.
The reaction is then centrifuged at 10000 g for 10 min; the recovered pellet is washed by centrifugation (10000 g, 10 min) 3-times with 1 mL of DMF and 2-times with 1 mL of MeOH. The final sample is resuspended in 1 mL of MeOH.

Table S3. Adopted quantities for the synthesis of PCN-222.

	**Entry**	**Ratio Mod/M 100**	**Entry**	**Ratio Mod/M 100**	**Entry**	**Ratio Mod/M 100**
**Sol. A**	$Zr(OEt)_4$	6.8 mg	$Zr(OiPr)_4$	9.7 mg	$Zr(OBut)_4$	11.5 µL
	FA	0.095 mL				
	DMF	0.905 mL				
	**Entry**	**Ratio L/M 0.33**				
**Sol. B**	TCPP	6.5 mg				
	DMF	1.000 mL				

	**Entry**	**Ratio Mod/M 250**	**Ratio Mod/M 560**			
**Sol. A**	$Zr(OEt)_4$	6.8 mg	6.8 mg			
	FA	0.236 mL	0.529 mL			
	DMF	0.764 mL	0.471 mL			
	**Entry**	**Ratio L/M 0.35**	**Ratio L/M 0.5**	**Ratio L/M 0.75**	**Ratio L/M 1.0**	**Ratio L/M 1.5**
**Sol. B**	TCPP	7.0 mg	10.0 mg	15.0 mg	20.0 mg	30.0 mg
	DMF	1.000 mL	1.000 mL	1.000 mL	1.000 mL	1.000 mL

Digestion of Zr-porphyrinic MOFs for ^{1}H-NMR quantification

Quantification of TCPP/OAc^- was conducted by digesting approximately 2 mg of each sample (L/M ratios 0.25, 0.50, 1.00, 1.50, and 2.00) using a tip of a spatula in a 2 mL Eppendorf tube. Subsequently, 980 µL of a 1M $NaHCO_3$ solution prepared in D_2O were added, and the mixture was sonicated for 20 minutes to achieve complete digestion of Zr-porphyrinic nanoMOFs, as per the following reaction:[2]

$$\mathrm{Zr_6O_4(OH)_4(TCPP)_x(OAc)_y + 18HCO_3^- \rightarrow 3[Zr_2(OH)_2(CO_3)_4]^{2-} + 8H_2O + 6CO_2 + x\,TCPP + y\,OAc}$$

Following digestion, 20 µL 50 mM of methylsulfonylmethane ($(CH_3)_2SO_2$) were introduced into the solution as an internal standard, featuring a singlet peak for 6 protons at a chemical shift of 3.18 ppm.[3]

Continuous PCN-224 synthesis procedure

Stock solutions of ligand and metal precursor were prepared separately: TCPP (0.032mmol, 25mg) was dissolved in 5 mL of DMF and $Zr(OEt)_4$ (0.125 mmol, 34 mg) in a mixture of DMF (1 mL) and acetic acid (4 mL). Then both solutions $Zr(OEt)_4$ and TCPP were loaded in two discrete injection channels and were pumped using a syringe pump at room temperature with an individual flow rate 0.5 mL/min into PP Y-micromixer (I.D= 2.3mm) connected to 146 mm long silicone tube (1.8 I.D mm) giving a residence time of 25 s. The obtained purple powder was washed twice with DMF (1mL) and three times with MeOH (1 mL). The final sample is resuspended in 1 mL of MeOH.

Alkoxy (acetic acid) L/M: 0.25 – 2.0 Mod/M = 560

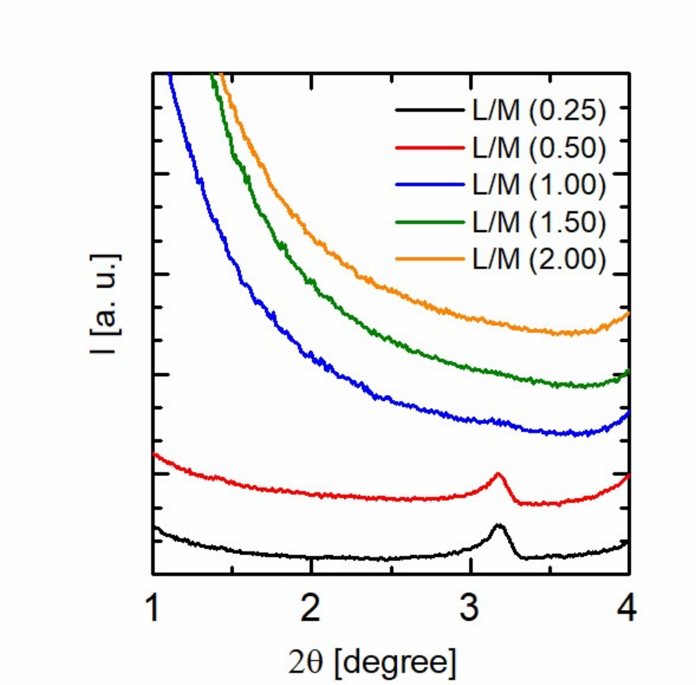

Figure S1. Small angle diffraction measurements of Zr-porphyrinic MOFs with different L/M ratios.

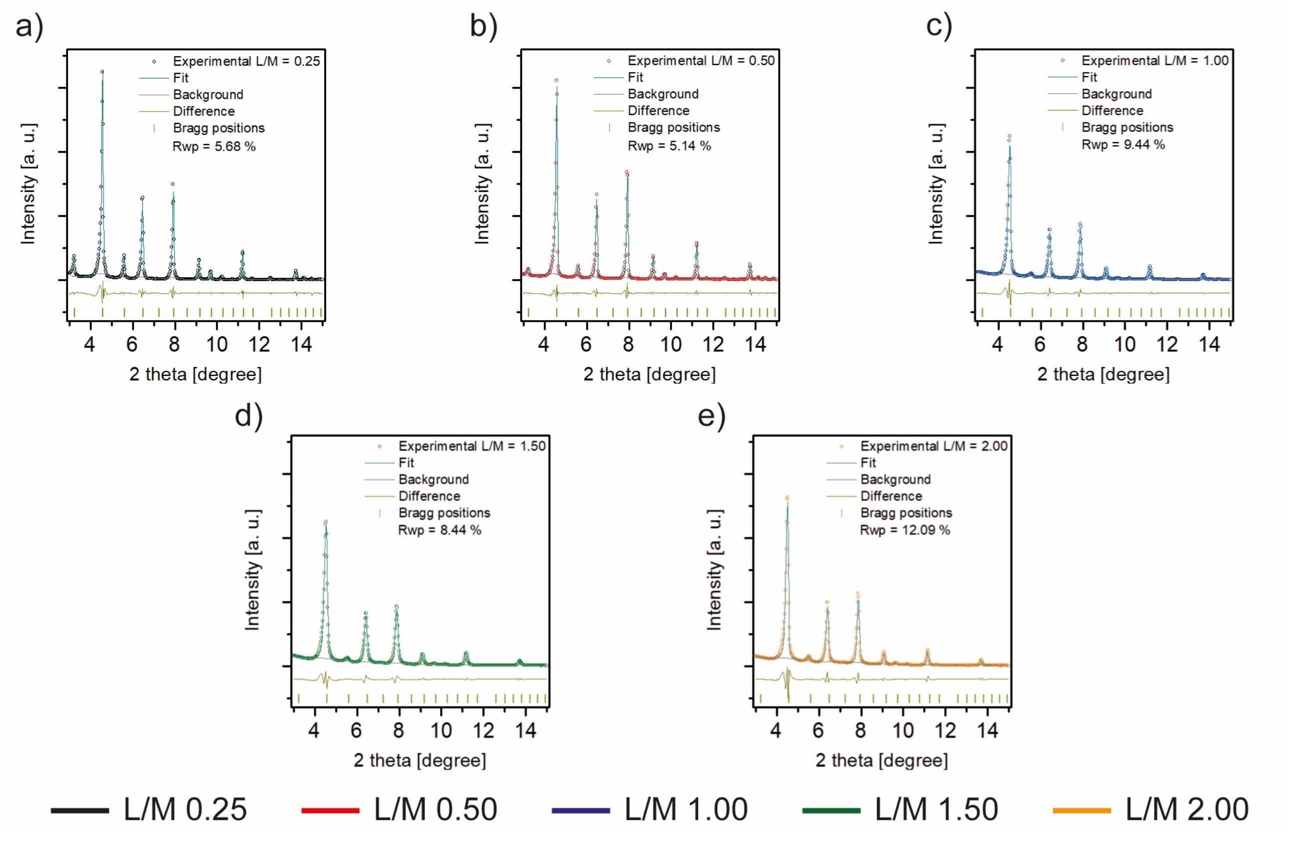

Figure S2. Pawley refinement for PCN-224 of Zr-porphyrinic MOF nanoparticles for a) 0.25, b) 0.50, c) 1.00, d) 1.50 and e) 2.00 L/M ratios.

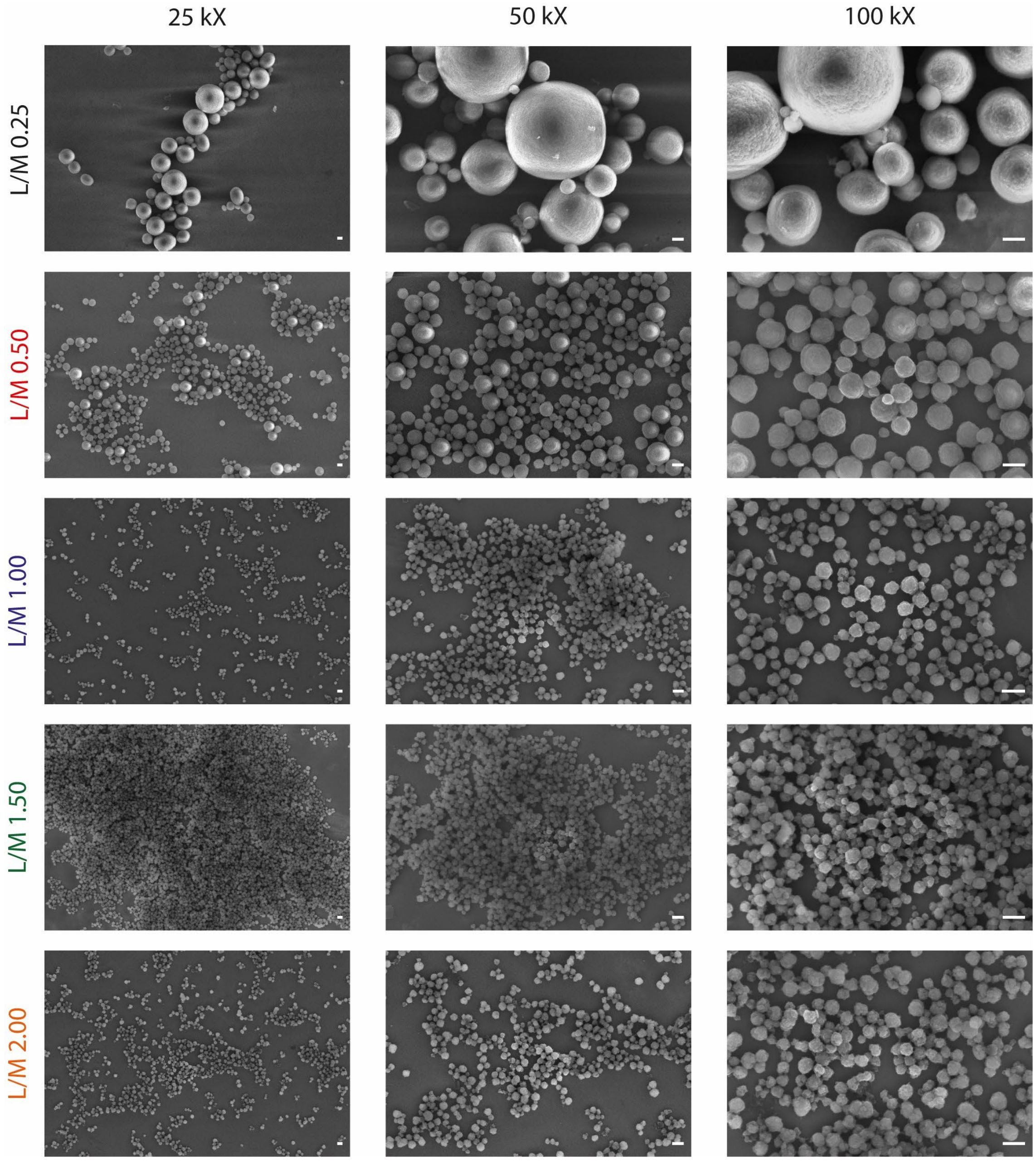

Figure S3. FE-SEM images of Zr-porphyrinic MOF nanoparticles at different magnifications (25 kX, 50 kX and 100 kX).

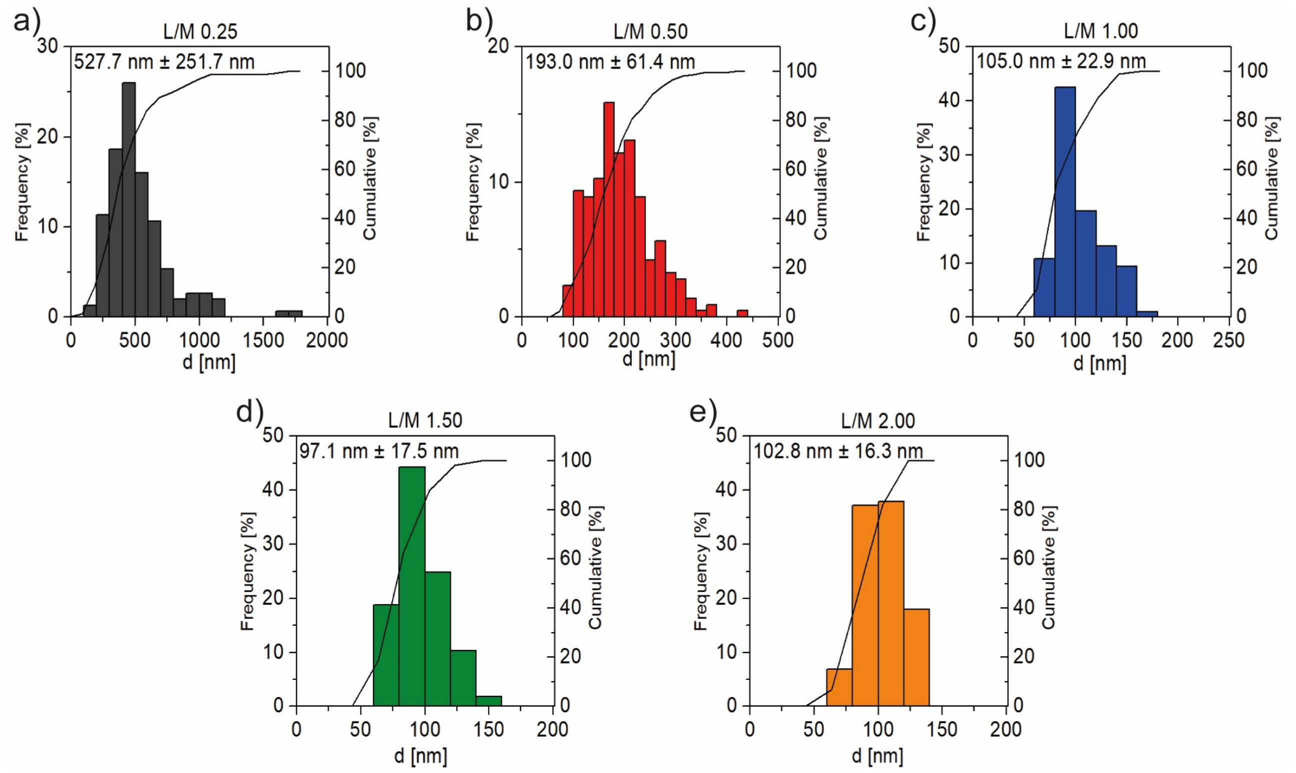

Figure S4. Particle size distribution histograms of Zr-porphyrinic MOF nanoparticles for a) 0.25, b) 0.50, c) 1.00, d) 1.50 and e) 2.00 L/M ratios.

$$D = \frac{K\lambda}{\beta\cos\theta} \qquad \text{Equation S1}$$

$$D = \text{Average crystallite size (nm)}$$

$$K = \text{Shape factor (0.94 for spherical crystallites with cubic symmetry)}$$

$$\lambda = \text{X} - \text{ray wavelength. Cu } K_{\alpha} \text{ average } = 1.54178 \text{ Å}$$

$$\beta = \text{FWHM (Full Width at Half Maximum)}$$

$$\theta = \text{XRD peak position, one half of } 2\theta$$

Table S4. Crystallite size of Zr-porphyrinic MOFs with different L/M ratios calculated by Scherrer equation.

L/M	Crystallite size (nm)
0.25	96.50
0.50	63.48
1.00	39.29
1.50	34.38
2.00	37.15

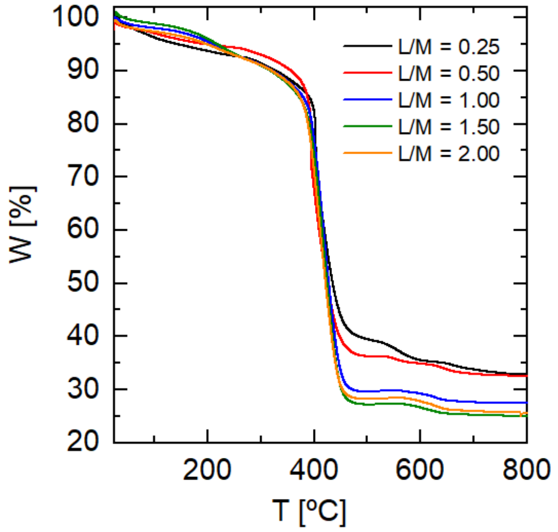

Figure S5. TGA without normalization of Zr-porphyrinic MOFs.

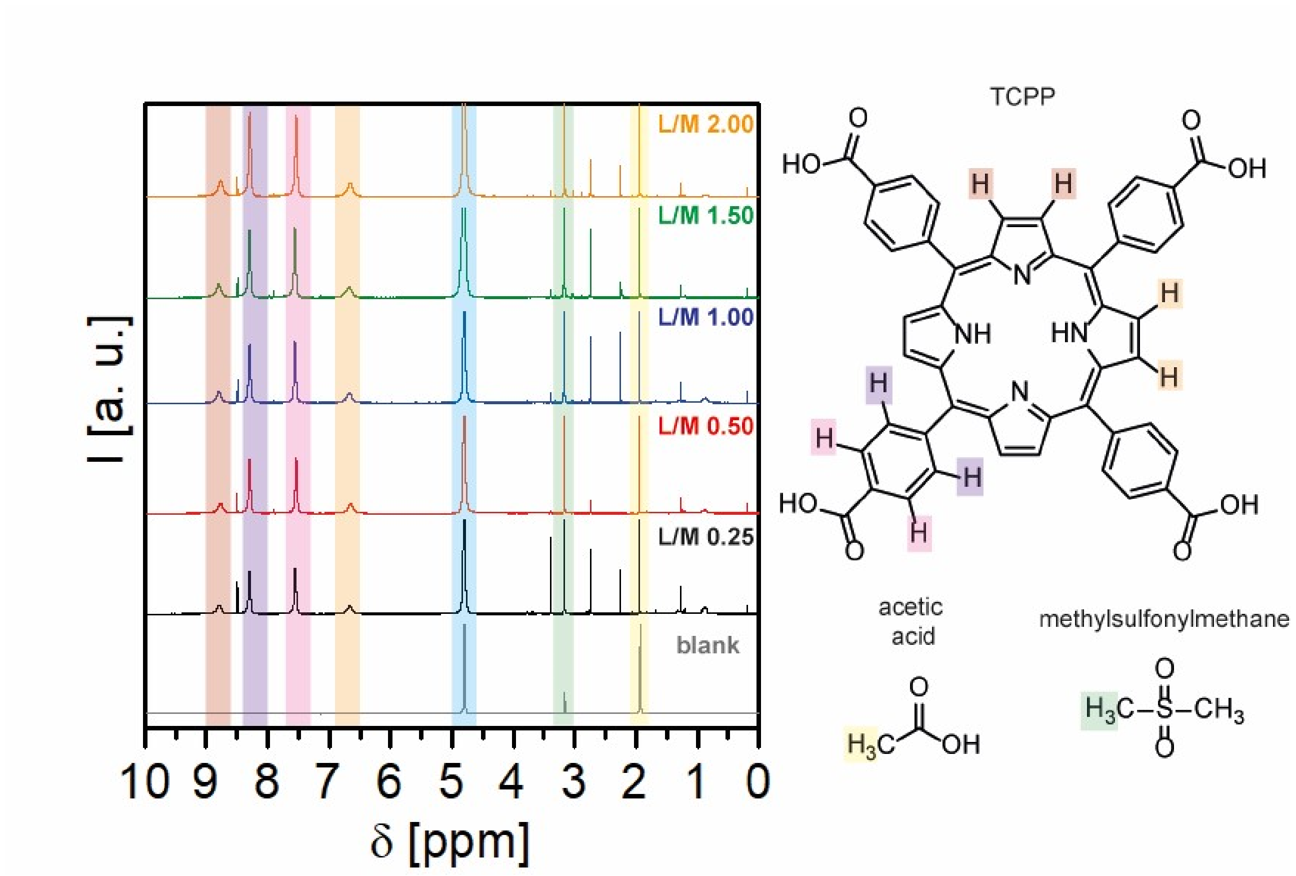

Figure S6. ^{1}H-NMR spectra of the blank (D_2O, $NaHCO_3$, CH_3COOH and $(CH_3)_2SO_2$) and Zr-porphyrinic MOFs. Signals not assigned correspond to remanent DMF and CH_3OH from the washing steps.[4]

Table S5. Assignation of ^{1}H-NMR spectra from Figure S10 and quantification using $(CH_3)_2SO_2$ as internal standard.

Sample	6H	3H	4H	4H	8H	8H	$(CH3)_2SO_2$ molecules	CH_3COO^- molecules	TCPP molecules	CH_3COO^- / TCPP
	$(CH_3)_2SO_2$	CH_3COO^-	pyrrol(1)	pyrrol (2)	benzene-o-porphyrin	benzene-m-porphyrin				
	(3.18ppm)	(1.95 ppm)	(8.79 ppm)	(6.66 ppm)	(8.30 ppm)	(7.55 ppm)				
L/M 0.25	1,95E+04	8,03E+04	-	-	1,37E+05	1,27E+05	6.0E+17	4,96E+18	3,06E+18	1,62
L/M 0.50	8,27E+04	7,89E+04			2,09E+05	2,08E+05		1,15E+18	1,14E+18	1,01
L/M 1.00	8,49E+04	2,46E+04			1,85E+05	1,82E+05		4,29E+17	9,76E+17	0,44
L/M 1.50	8,20E+04	2,33E+04			2,08E+05	1,98E+05		4,29E+17	4,46E+18	0,10
L/M 2.00	8,41E+04	3,01E+04			3,59E+05	3,63E+05		4,31E+17	2,00E+18	0,22

Table S6. Summary of N_2 uptake and BET surface area of Zr-porphyrinic MOFs with different L/M ratios at room temperature.

**L/M**	**N_2 uptake (cm^3/g)**	**BET surface area (m^2/g)**
0.25	991	2532
0.50	895	2428
1.00	670	2154
1.50	628	1987
2.00	631	2097

Table S7. Summary of different pore sizes of PCN-224, PCN-222 and MOF-525.

**Zr-porphyrinic MOF**	**Space group**	**Cell parameter (Å)**	**Cluster connectivity**	**Pore width (Å) calculated with Zeo++[5]**
**PCN-224**	$Im\bar{3}m$ (229)	a = 38.452	6-c	P_1 = 13.5<br>P_2 = 24.2
**PCN-222**	P6/mmm (191)	a = 41.968<br>c = 17.143	8-c	P_1 = 11.4<br>P_2 = 32.1
**MOF-525**	$Pm\bar{3}m$ (221)	a = 19.393	12-c	P_1 = 7.6<br>P_2 = 16.6

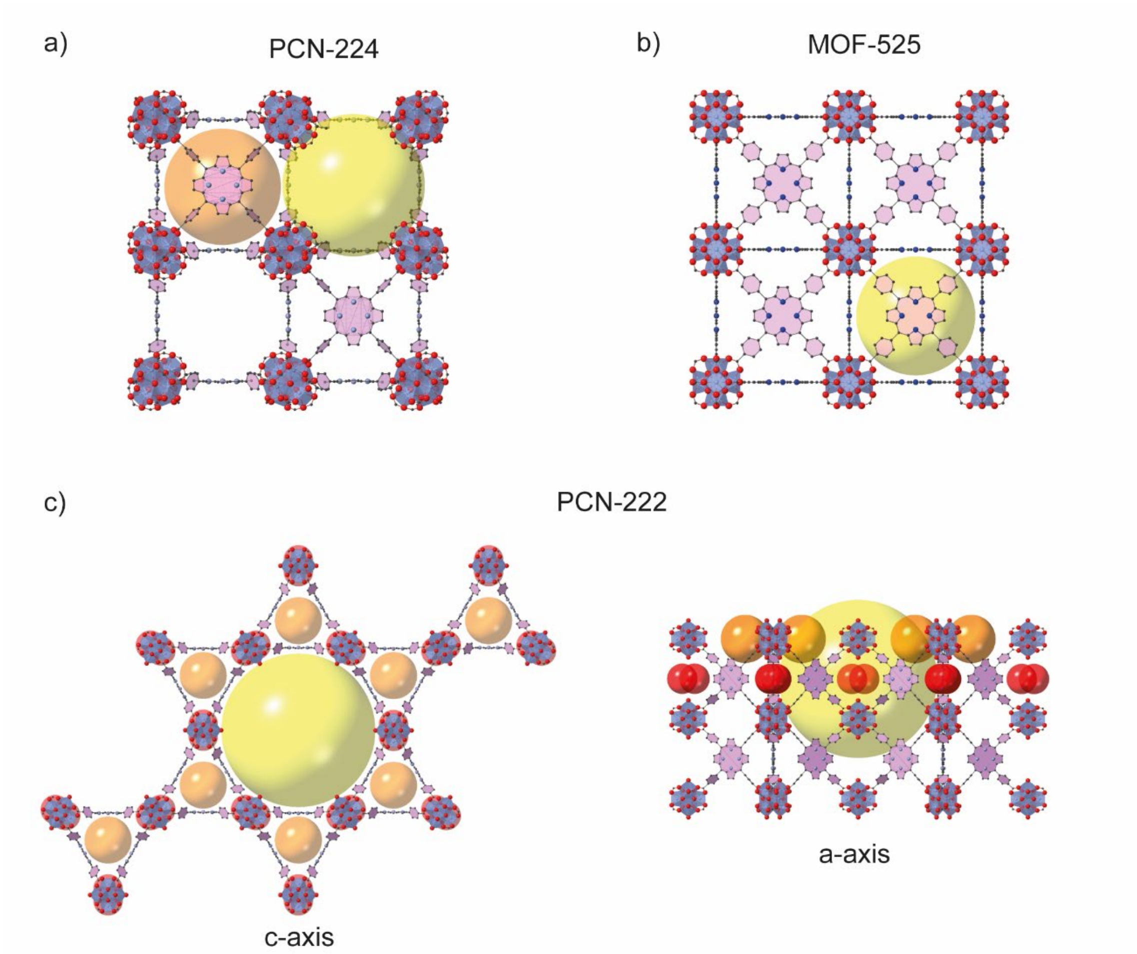

Figure S7. Pore size representation of a) PCN-224, b) MOF-525 and c) PCN-222.

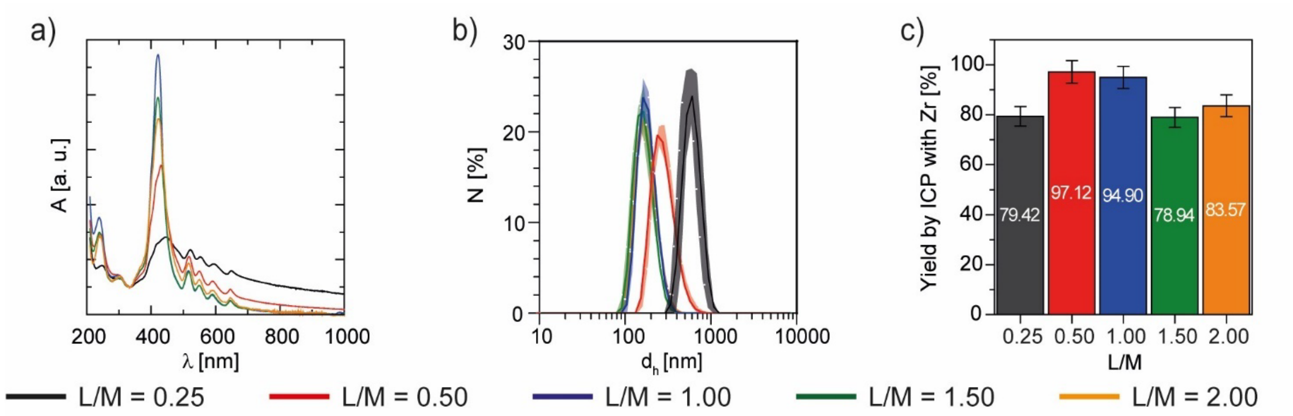

Figure S8. a) UV-Vis spectra, b) DLS analysis and c) yield of reaction obtained with the amount of Zr by ICP-OES of Zr-porphyrinic MOFs.

Table S8. Hydrodynamic size of Zr-porphyrinic MOFs with different L/M ratio measure by DLS in MeOH.

L/M	$D_{h,I}$ (nm)	$D_{h,V}$ (nm)	$D_{h,N}$ (nm)	PdI
0.25	636.0 ± 66.9	679.2 ± 78.7	618.1 ± 71.1	0.314 ± 0.050
0.50	386.8 ± 33.6	442.5 ± 93.2	307.7 ± 5.4	0.175 ± 0.044
1.00	216.2 ± 8.9	217.5 ± 9.2	186.1 ± 3.7	0.185 ± 0.023
1.50	200.7 ± 4.7	200.5 ± 4.3	168.7 ± 5.2	0.064 ± 0.043
2.00	222.5 ± 2.7	223.9 ± 2.9	180.9 ± 4.8	0.099 ± 0.038

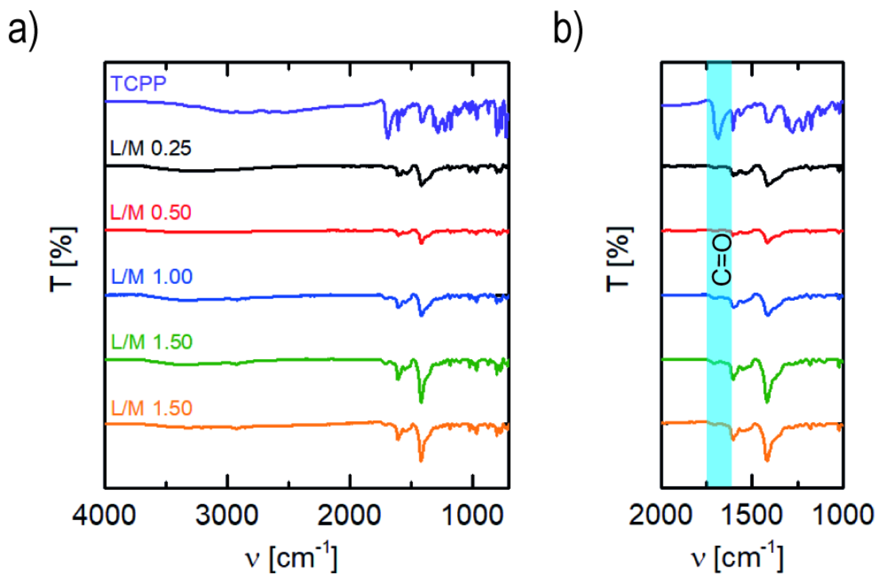

Figure S9. a) FT-IT spectra of Zr-porphyrinic MOFs and b) zoom in carbonyl region.

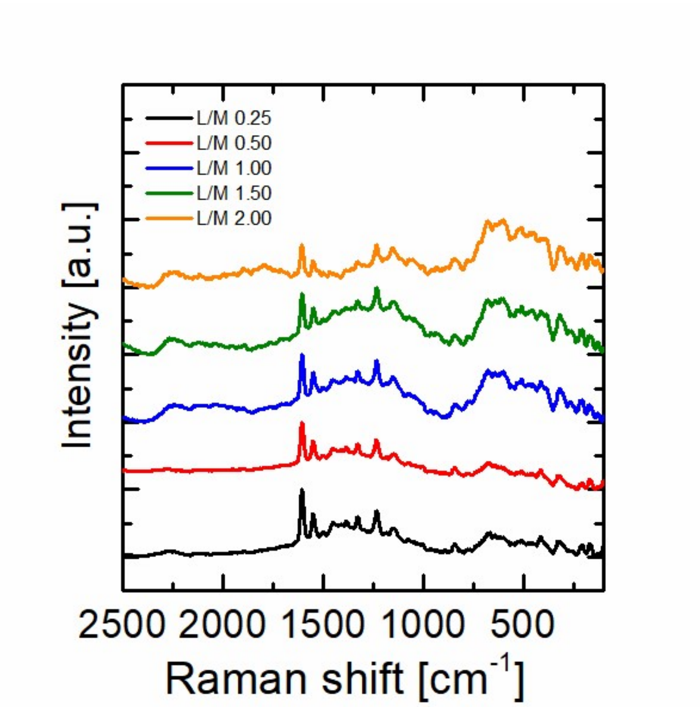

Figure S10. Raman spectra of Zr-porphyrinic MOFs.

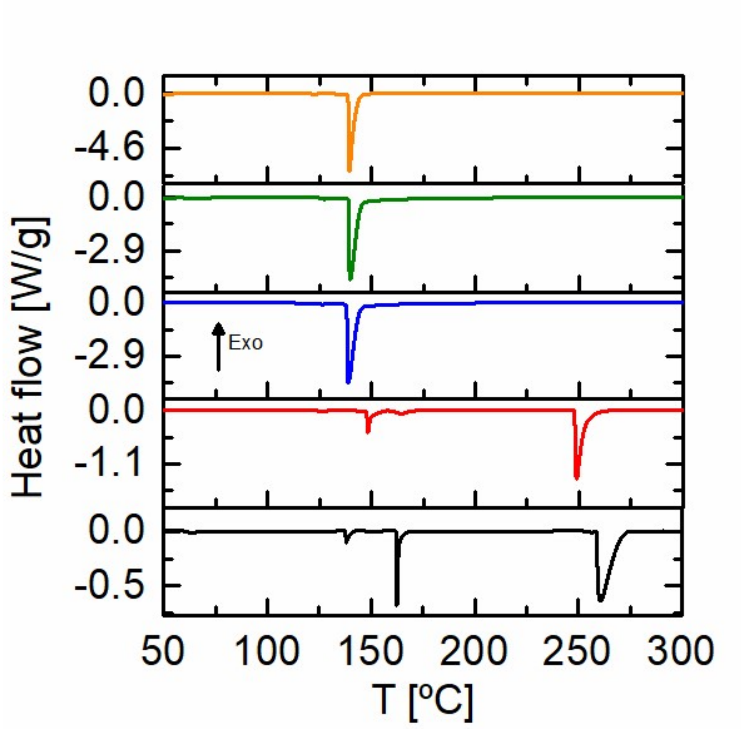

Figure S11. DSC analysis of Zr-porphyrinic MOFs for L/M = 0.25 (black), 0.50 (red), 1.00 (blue), 1.50 (gree) and 2.00 (orange).

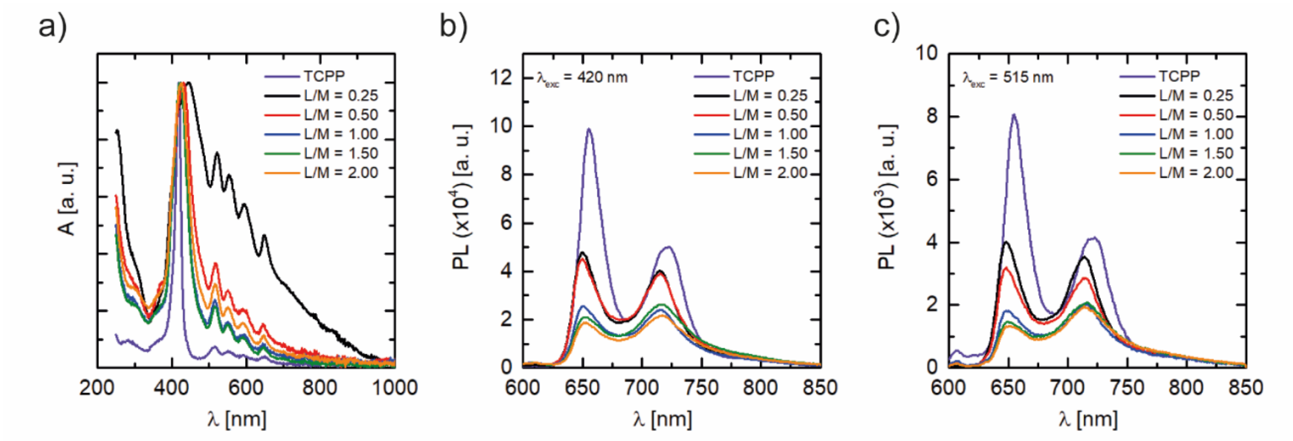

Figure S12. a) Normalized absorption spectra and photoluminescence spectra at b) 420nm and c) 515 nm excitation wavelength of Zr-porphyrinic MOFs.

EtO (acetic acid) role of the temperature in the reaction yield

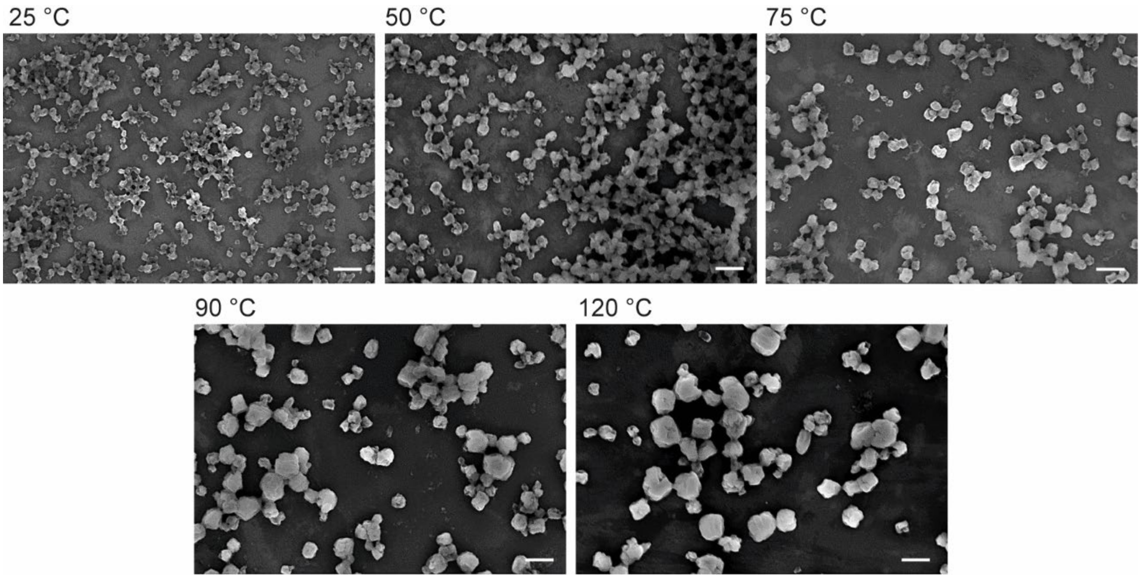

Figure S13. Representative FE-SEM images for 1 h reaction performed at different temperatures (25 °C, 50 °C, 75 °C, 90 °C and 120 °C). The ratios adopted for the synthesis of the particles are L/M = 0.25, Mod/M = 250; the Zr precursor used is $Zr(OEt)_4$ and the modulator is AA. Scale bars: 200 nm.

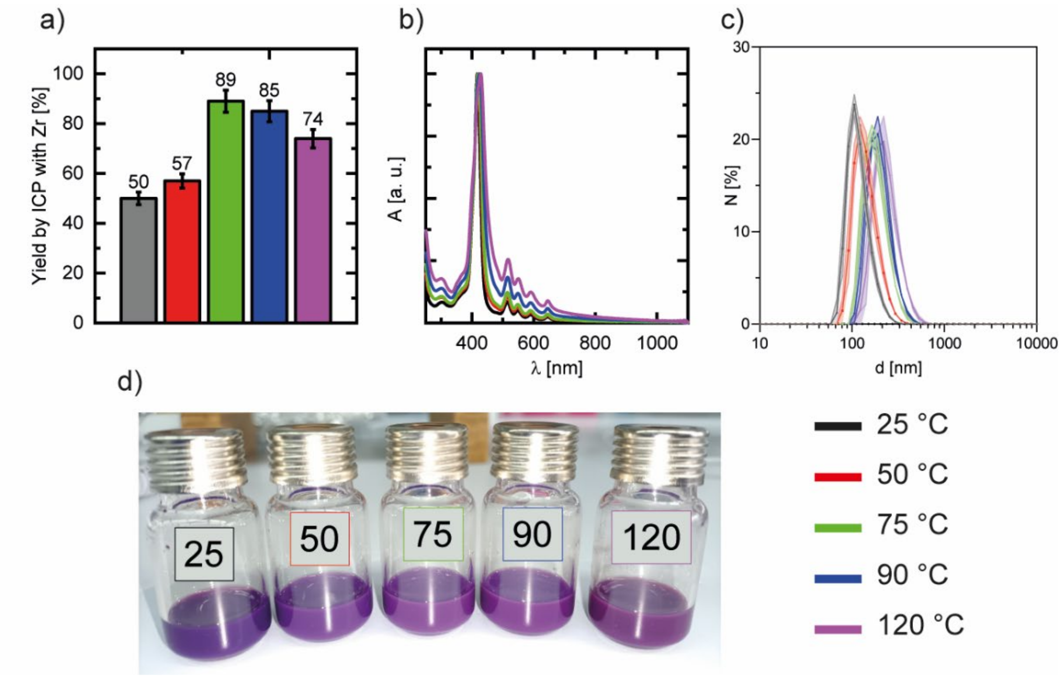

Figure S14. a) Graphical representation of the reaction yield evaluated through ICP-OES (see Table S1 for more details), b) UV-Vis extinction spectra, c) hydrodynamic diameters (by number) and d) digital images of 1h reaction conducted at different temperatures.

Table S9. Zr quantification through ICP-OES to determine the reaction yield at different temperatures (25 °C, 50 °C, 75 °C, 90 °C and 120 °C) of 1 h reaction. The reaction yield (Yield%) is calculated from the relation between the amount of Zr at the beginning of the reaction (Zr (precursor)) and the amount of Zr in the final particles after purification. Each measurement is performed in triplicate and the Zr value is the average value of two Zr emission lines (339.198 nm and 343.823 nm, respectively). The ratios adopted for the synthesis of the particles are L/M = 0.25, Mod/M = 250; the Zr precursor used is $Zr(OEt)_4$ and the modulator is AA.

Entry	Zr average	Yield%
Zr (precursor)	2.05 ± 0.10	/
25 °C	1.02 ± 0.05	49.9 ± 2.5
50 °C	1.17 ± 0,06	57.1 ± 2.8
75 °C	1.83 ± 0.09	88.9 ± 4.4
90 °C	1.74 ± 0.09	85.0 ± 4.2
120 °C	1.52 ± 0.08	74.2 ± 3.7

Table S10. Hydrodynamic diameter by intensity ($d_{h,I}$) and by number ($d_{h,n}$) measured through DLS of the sample synthesized at different temperatures (1 h reaction). The Polydispersity Index (PdI) is also reported.

Entry	$d_{h,I}$ (nm)	$d_{h,n}$ (nm)	PdI
25 °C	163.4 ± 3.6	121.3 ± 5.2	0.190 ± 0.028
50 °C	195.6 ± 3.7	144.9 ± 5.9	0.137 ± 0.013
75 °C	233.0 ± 5.4	184.4 ± 2.2	0.221 ± 0.009
90 °C	250.9 ± 6.5	200.8 ± 4.7	0.093 ± 0.022
120 °C	302.1 ± 12.8	237.5 ± 5.5	0.089 ± 0.027

Alkoxy (acetic acid) 75 °C (1 h): L/M = 0.25 Mod/M = 250

In this section, the results relative to the reaction performed at 75 °C (1 h) in presence of different Zr precursors ($Zr(OEt)_4$, $Zr(OiPr)_4$ and $Zr(OBut)_4$) and with the use of AA as modulator are reported. The ratios adopted for the synthesis of the particles are L/M = 0.25, Mod/M = 250.

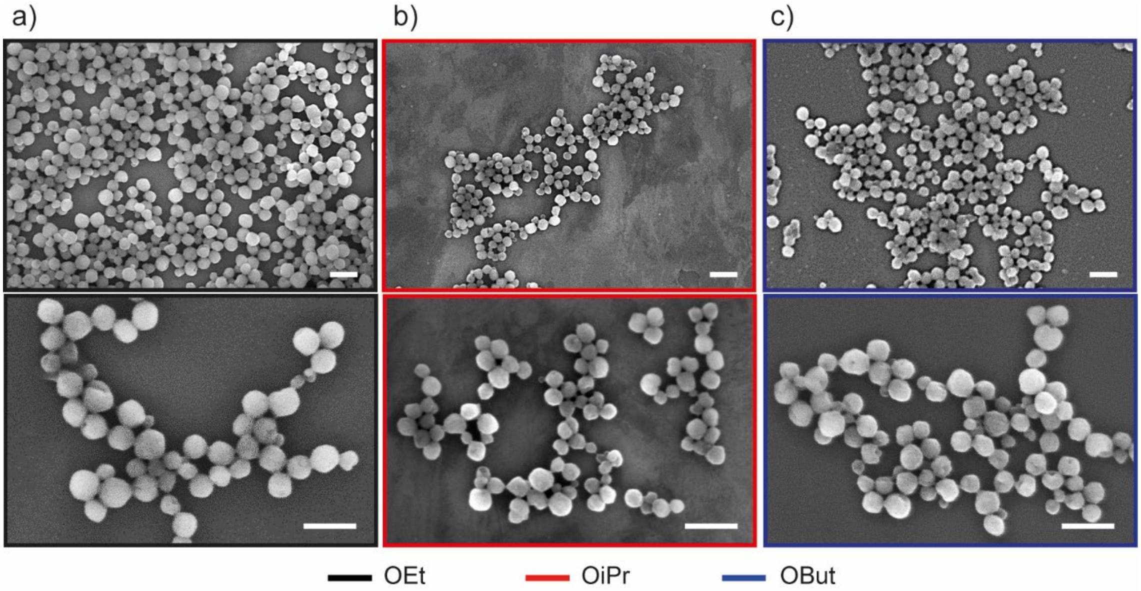

Figure S15. Representative FE-SEM images for 1 h reaction performed at 75 °C by using different Zr precursors and AA as modulator. Scale bars: 200 nm.

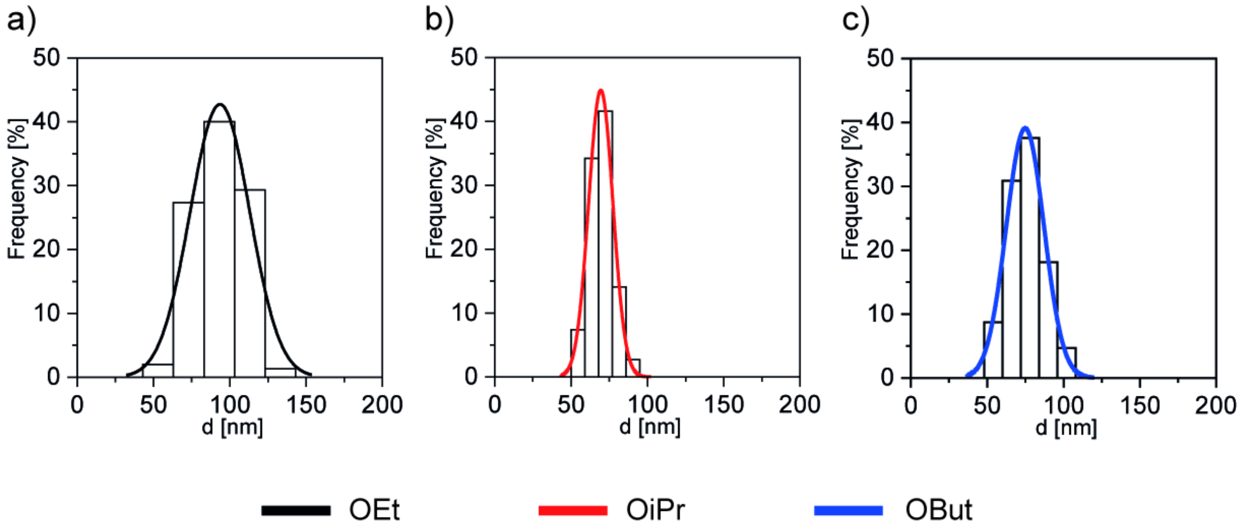

Figure S16. Particle size distribution histogram relative to SEM images of 1 h reaction performed at 75 °C by using different Zr precursors and AA as modulator.

Table S11. Particle size distribution measured through SEM analysis of the samples synthesized with different Zr precursors.

Precursor	Modulator	Time	Temperature	d_{SEM} (nm)
$Zr(OEt)_4$	AA	1 h	75 °C	92.7 ± 17.3
$Zr(OiPr)_4$				69.8 ± 7.6
$Zr(OBut)_4$				75.5 ± 11.5

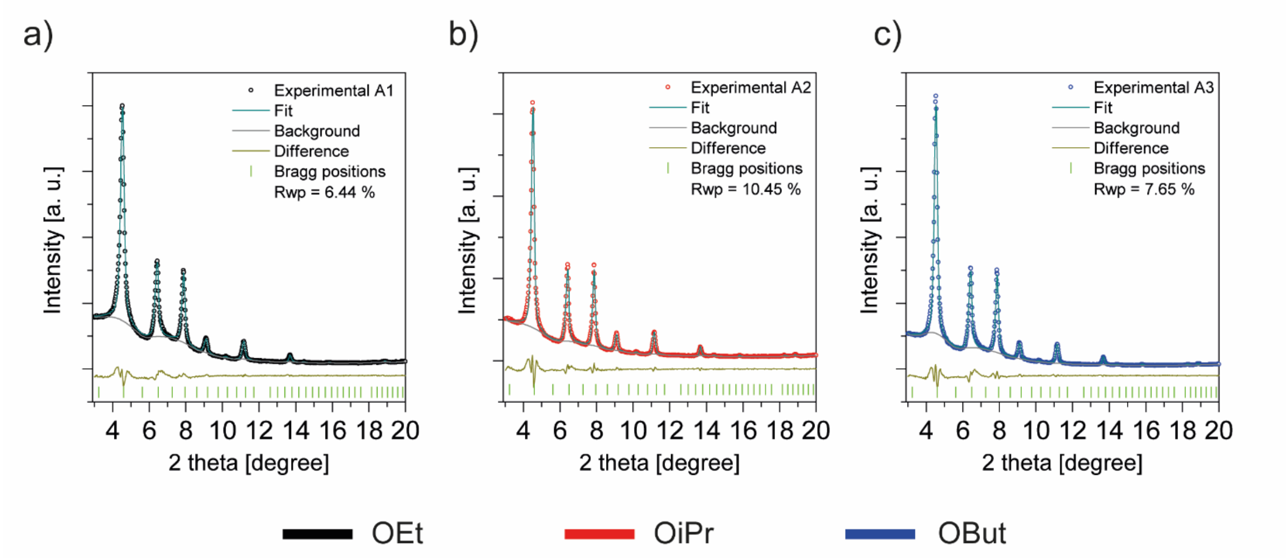

Figure S17. Pawley refinement of Zr-porphyrinic MOF nanoparticles, synthesized at 75 °C, 1 h reaction, by using different Zr precursors and AA as modulator.

Table S12. BET surface area and pore volume of the particles synthesized with different Zr precursors.

Precursor	BET surface area (m^2/g)	t-plot micropore volume (cm^3/g)
$Zr(OEt)_4$	2467	0.718
$Zr(OiPr)_4$	3083	0.904
$Zr(OBut)_4$	2629	0.845

Table S13. Zr quantification through ICP-OES to determine the reaction yield of the reaction performed with different Zr precursors.

Precursor	Modulator	Time	Temperature	Yield%
$Zr(OEt)_4$	AA	1 h	75 °C	88.9 ± 4.4
$Zr(OiPr)_4$				71.2 ± 3.6
$Zr(OBut)_4$				85.2 ± 4.2

Table S14. Hydrodynamic diameter by intensity ($d_{h,I}$) and by number ($d_{h,n}$) and the Polydispersity Index (PdI) measured through DLS of the samples synthesized with different Zr precursors at 75 ºC foy 1h. The Polydispersity Index is also reported (PdI).

Precursor	$d_{h,I}$ (nm)	$d_{h,n}$ (nm)	PdI
$Zr(OEt)_4$	176.6 ± 3.4	153.9 ± 1.8	0.026 ± 0.005
$Zr(OiPr)_4$	241.2 ± 17.0	158.1 ± 13.6	0.155 ± 0.041
$Zr(OBut)_4$	189.2 ± 2.2	158.2 ± 1.1	0.021 ± 0.018

Alkoxy (acetic acid) 25 °C (1 h vs 24 h): L/M = 0.25 Mod/M = 250

In this section, the results relative to the reaction performed at 25 °C in presence of different Zr precursors ($Zr(OEt)_4$, $Zr(OiPr)_4$ and $Zr(OBut)_4$) and with the use of AA as modulator are reported. The ratios adopted for the synthesis of the particles are L/M = 0.25, Mod/M = 250.

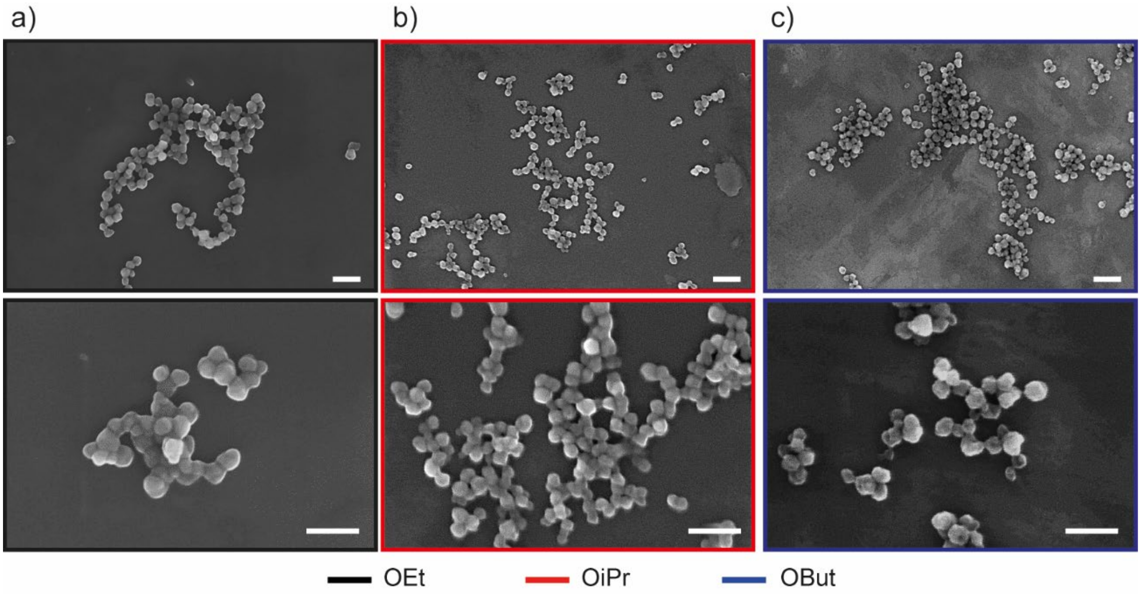

Figure S18. Representative FE-SEM images for 1 h reaction performed at RT by using different Zr precursors and AA as modulator. Scale bars: 200 nm.

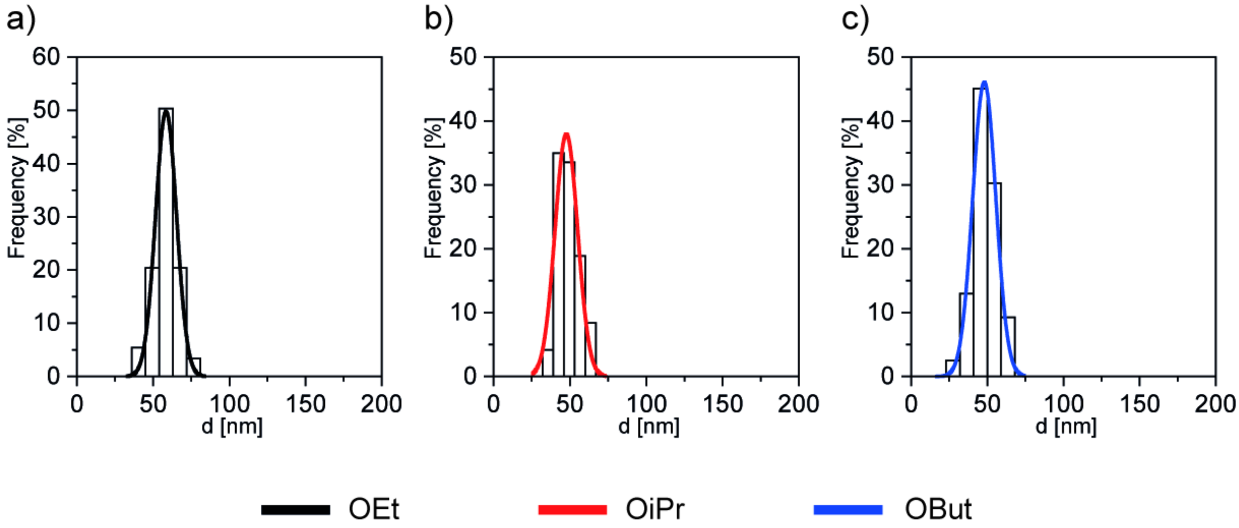

Figure S19. Particle size distribution histogram relative to SEM images of 1 h reaction performed at RT by using different Zr precursors and AA as modulator.

Table S15. Particle size distribution measured through SEM analysis of the samples synthesized with different Zr precursors.

Precursor	Modulator	Time	Temperature	d_{SEM} (nm)
$Zr(OEt)_4$	AA	1 h	25 °C	58.2 ± 7.7
$Zr(OiPr)_4$				49.1 ± 6.8
$Zr(OBut)_4$				48.2 ± 8.3

Table S16. Zr quantification through ICP-OES to determine the reaction yield of the reaction performed with different Zr precursors after 1h and 24h of reaction.

Precursor	Modulator	Temperature	Time	Yield%
$Zr(OEt)_4$	AA	25 °C	1 h	49.9 ± 2.5
$Zr(OiPr)_4$				43.0 ± 2.2
$Zr(OBut)_4$				55.3 ± 2.8
$Zr(OEt)_4$			24 h	68.5 ± 3.4
$Zr(OiPr)_4$				69.8 ± 3.5
$Zr(OBut)_4$				78.1 ± 3.9

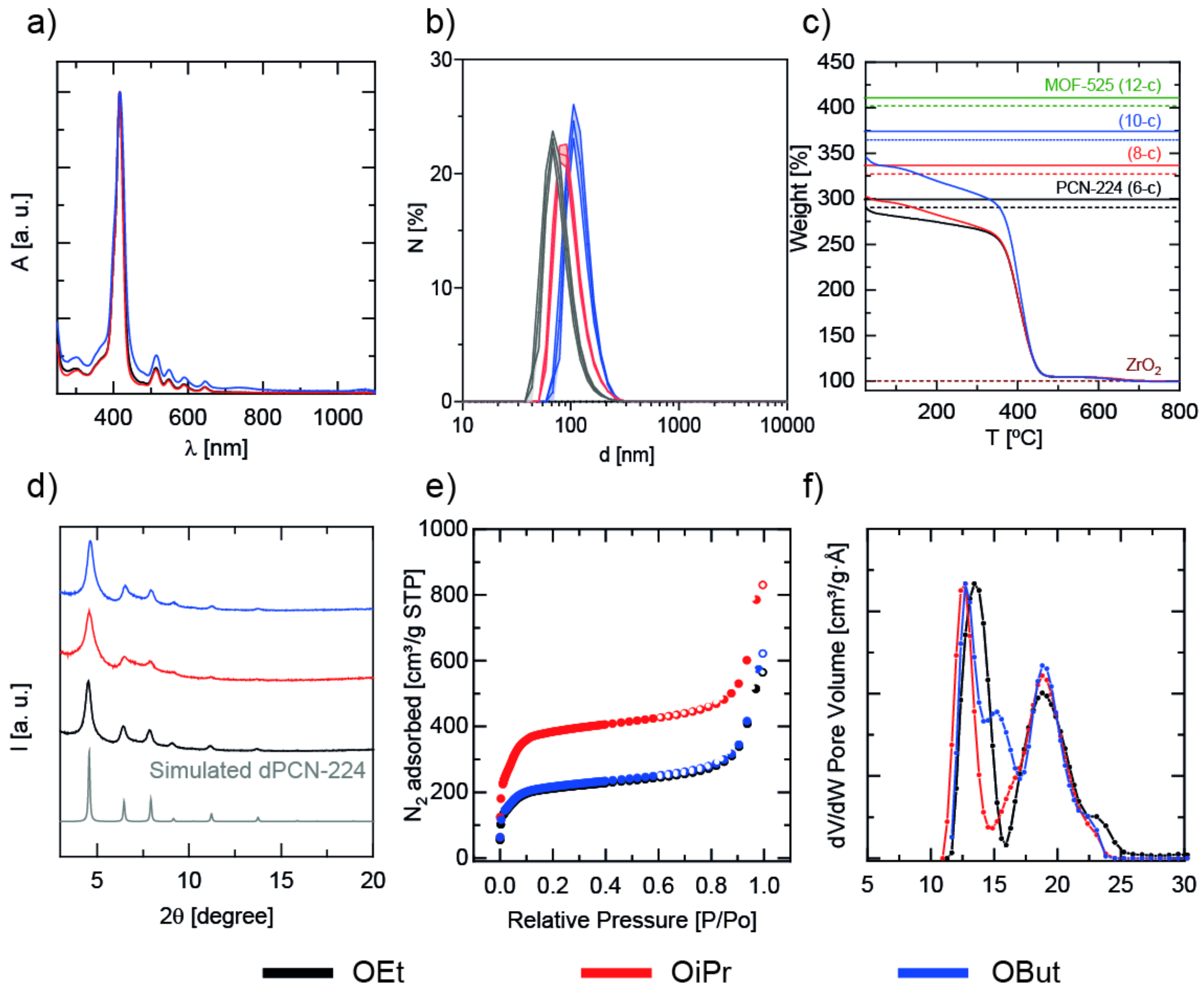

Figure S20. a) UV-Vis extinction spectra, b) hydrodynamic diameters (by number), c) thermogravimetric analysis (TGA), d) powder X-rays diffraction (PXRD) patterns, e) BET analysis and f) NLDFT pore size distribution of 1 h reaction at RT by using different Zr precursors.

Table S17. Hydrodynamic diameter by intensity ($D_{h,I}$) and by number ($D_{h,n}$) and the Polydispersity Index (PdI) measured through DLS of the samples synthesized with different Zr precursors for 1 h at RT. The Polydispersity Index is also reported (PdI).

Precursor	Time	$D_{h,I}$ (nm)	$D_{h,n}$ (nm)	PdI
$Zr(OEt)_4$	1 h	141.6 ± 1.8	79.5 ± 3.0	0.206 ± 0.003
$Zr(OiPr)_4$		156.6 ± 1.5	99.2 ± 1.8	0.102 ± 0.018
$Zr(OBut)_4$		153.5 ± 4.3	119.8 ± 39.3	0.060 ± 0.011

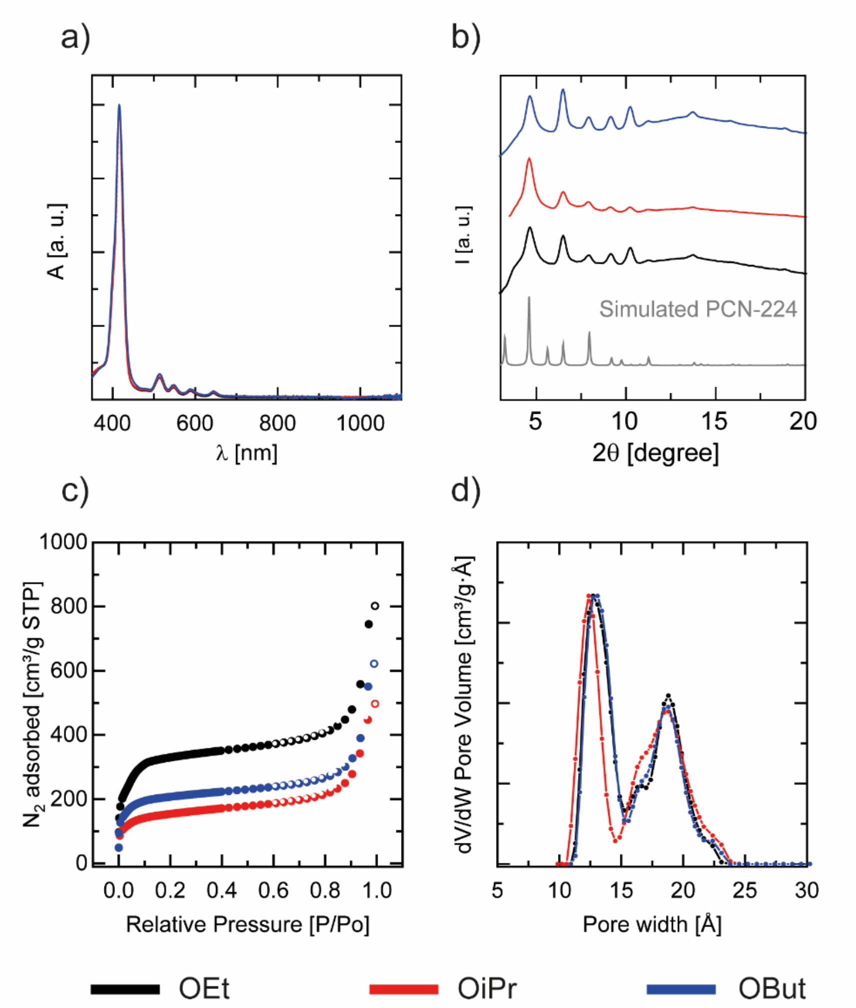

Figure S21. a) UV-Vis extinction spectra, b) PXRD patterns, c) BET analysis and d) NLDFT pore size distribution of 24 h reaction at RT by using different Zr precursors.

Table S18. BET surface area and pore volume of the particles synthesized with different Zr precursors.

Precursor	Time	BET surface area (m^2/g)	t-plot micropore volume (cm^3/g)
$Zr(OEt)_4$	1 h	816	0.218
$Zr(OiPr)_4$		1561	0.444
$Zr(OBut)_4$		833	0.206
$Zr(OEt)_4$	24 h	1347	0.363
$Zr(OiPr)_4$		580	0.132
$Zr(OBut)_4$		792	0.223

Alkoxy (formic acid) 75 °C

L/M = 0.35, Mod/M = 100; 24h; 75 °C

In this section, the results relative to the reaction performed at 75 °C (24 h) in presence of different Zr precursors ($Zr(OEt)_4$, $Zr(OiPr)_4$ and $Zr(OBut)_4$) and with the use of formic acid as modulator are reported. The ratios adopted for the synthesis of the particles are L/M = 0.35, Mod/M = 100.

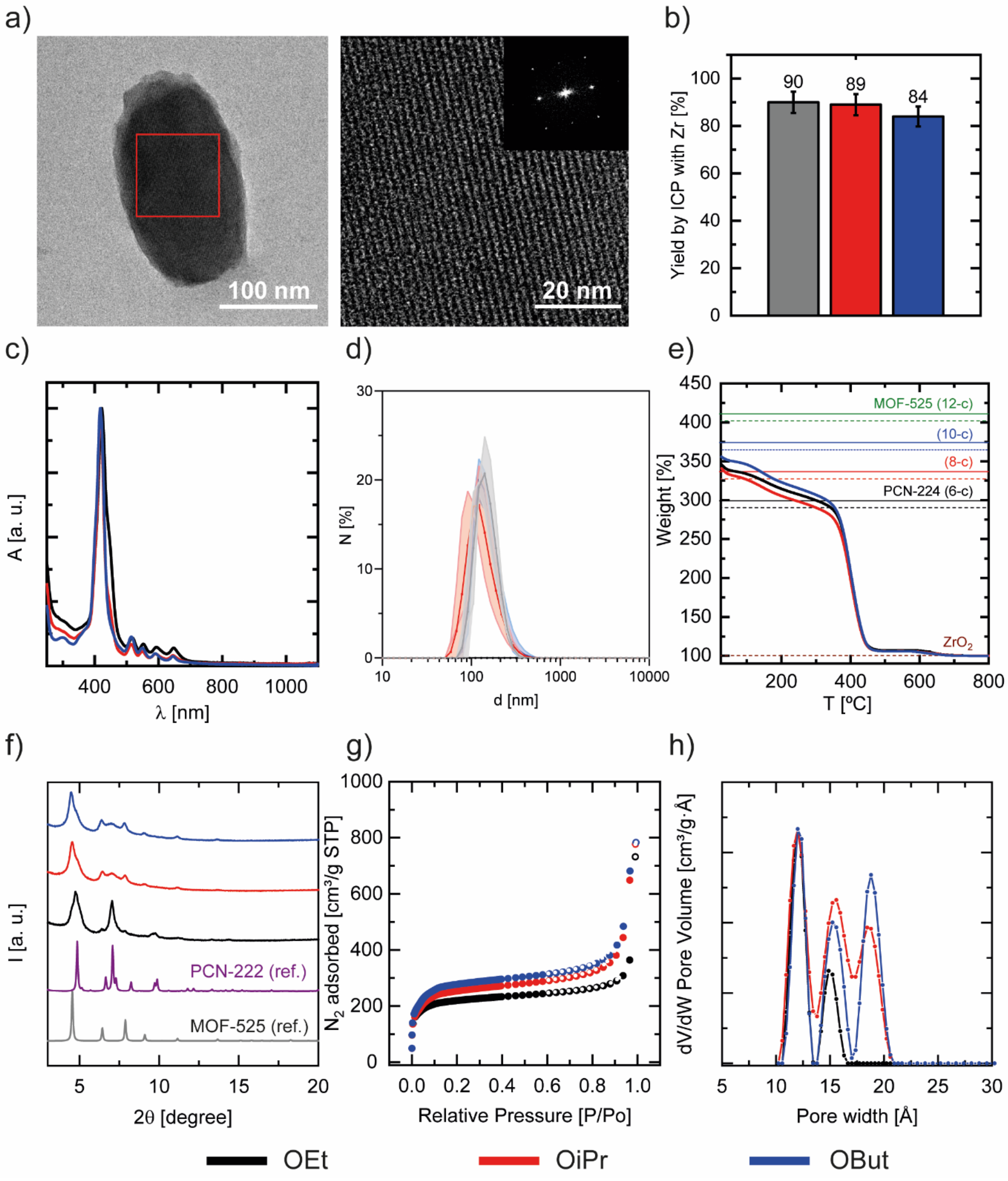

Figure S22. a) HRTEM images of nanoMOFs synthesized starting from $Zr(OEt)_4$, b) graphical representation of the reaction yields evaluated through ICP-OES of 1h-reaction

conducted at 75 °C for 24h c) UV-Vis extinction spectra, d) hydrodynamic diameters (by number), e) thermogravimetric analysis (TGA), f) PXRD patterns, g) N_2 adsorption isotherms at 77K and h) NLDFT pore size distribution.

Table S19. Zr quantification through ICP-OES to determine the reaction yield of the reaction performed with different Zr precursors.

Precursor	Modulator	Time	Temperature	Yield%
$Zr(OEt)_4$	Formic acid	24 h	75 °C	89.7 ± 4.5
$Zr(OiPr)_4$				88.6 ± 4.4
$Zr(OBut)_4$				84.5 ± 4.2

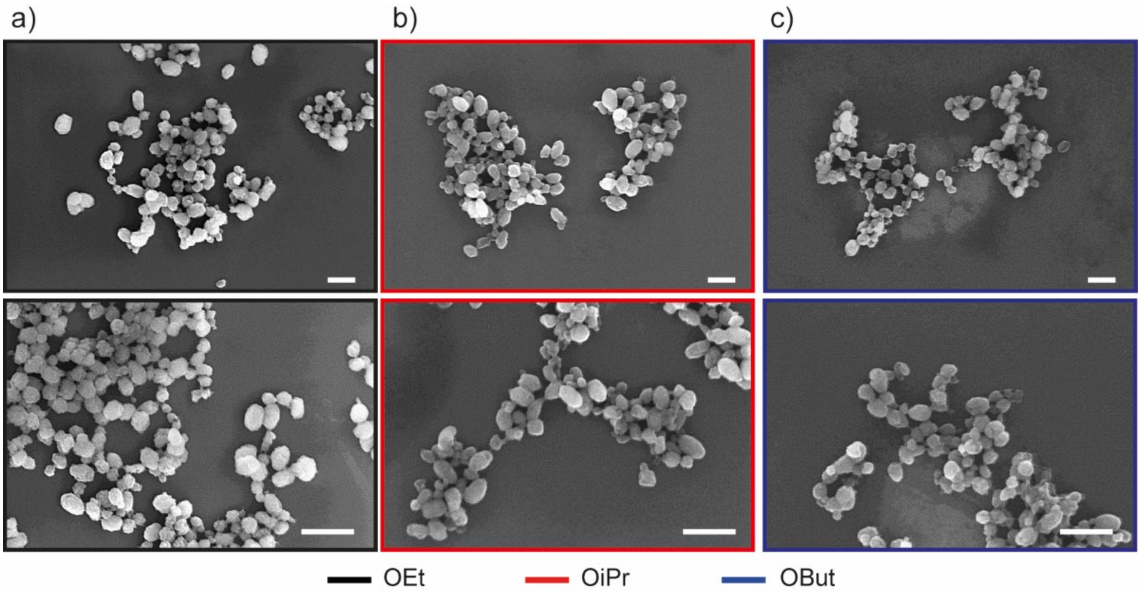

Figure S23. Representative FE-SEM images for 24 h reaction performed at 75 °C by using different Zr precursors and formic acid as modulator. Scale bars: 200 nm.

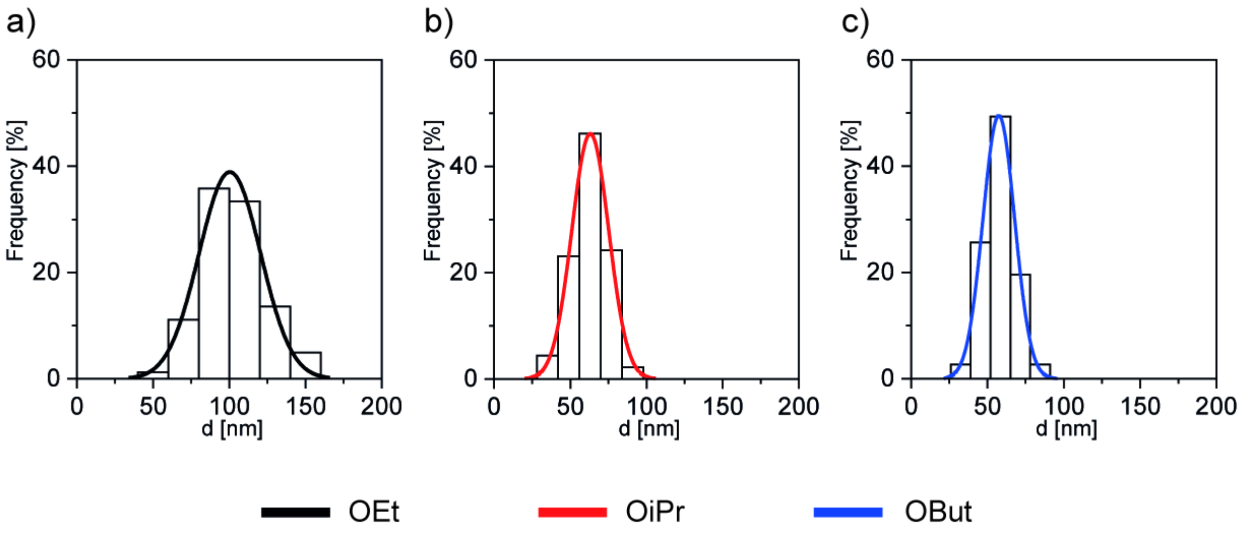

Figure S24. Particle size distribution histogram relative to SEM images of 24 h reaction performed at 75 °C by using different Zr precursors and formic acid as modulator.

Table S20. Particle size distribution measured through SEM analysis of the samples synthesized with different Zr precursors.

Precursor	Modulator	Time	Temperature	d_{SEM} (nm)
$Zr(OEt)_4$	Formic acid	24 h	75 °C	102.8 ± 20.1
$Zr(OiPr)_4$				62.8 ± 12.1
$Zr(OBut)_4$				57.2 ± 10.0

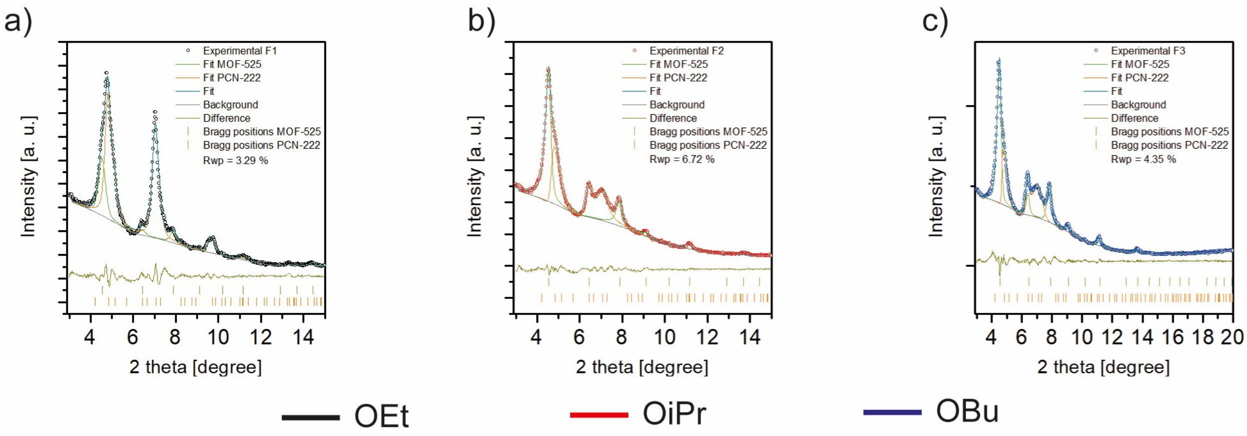

Figure S25. Pawley refinement of Zr-porphyrinic MOF nanoparticles, synthesized at 75 °C, 24 h reaction, by using different Zr precursors and formic acid as modulator.

Table S21. Hydrodynamic diameter by intensity ($D_{h,I}$) and by number ($D_{h,n}$) and the Polydispersity Index (PdI) measured through DLS of the samples synthesized with different Zr precursors. The Polydispersity Index is also reported (PdI).

Precursor	Time	$D_{h,I}$ (nm)	$D_{h,n}$ (nm)	PdI
$Zr(OEt)_4$	24 h	191.5 ± 4.4	151.7 ± 11.3	0.038 ± 0.032
$Zr(OiPr)_4$		214.2 ± 5.1	136.1 ± 19.1	0.174 ± 0.053
$Zr(OBut)_4$		215.6 ± 34.6	154.0 ± 11.9	0.135 ± 0.065

Table S22. BET surface area and pore volume of the particles synthesized with different Zr precursors.

Precursor	BET surface area (m^2/g)	t-plot micropore volume (cm^3/g)
$Zr(OEt)_4$	840	0.250
$Zr(OiPr)_4$	981	0.275
$Zr(OBut)_4$	1068	0.315

L/M = 0.35-1.5, Mod/M = 250 or 560; 1h; 75 °C

In this section, the results shown are relative to the reaction performed at 75 °C (1 h) with $Zr(OEt)_4$ as a precursor and formic acid as modulator. The ratios adopted for the synthesis were L/M = 0.35-1.5 and Mod/M = 250 or 560.

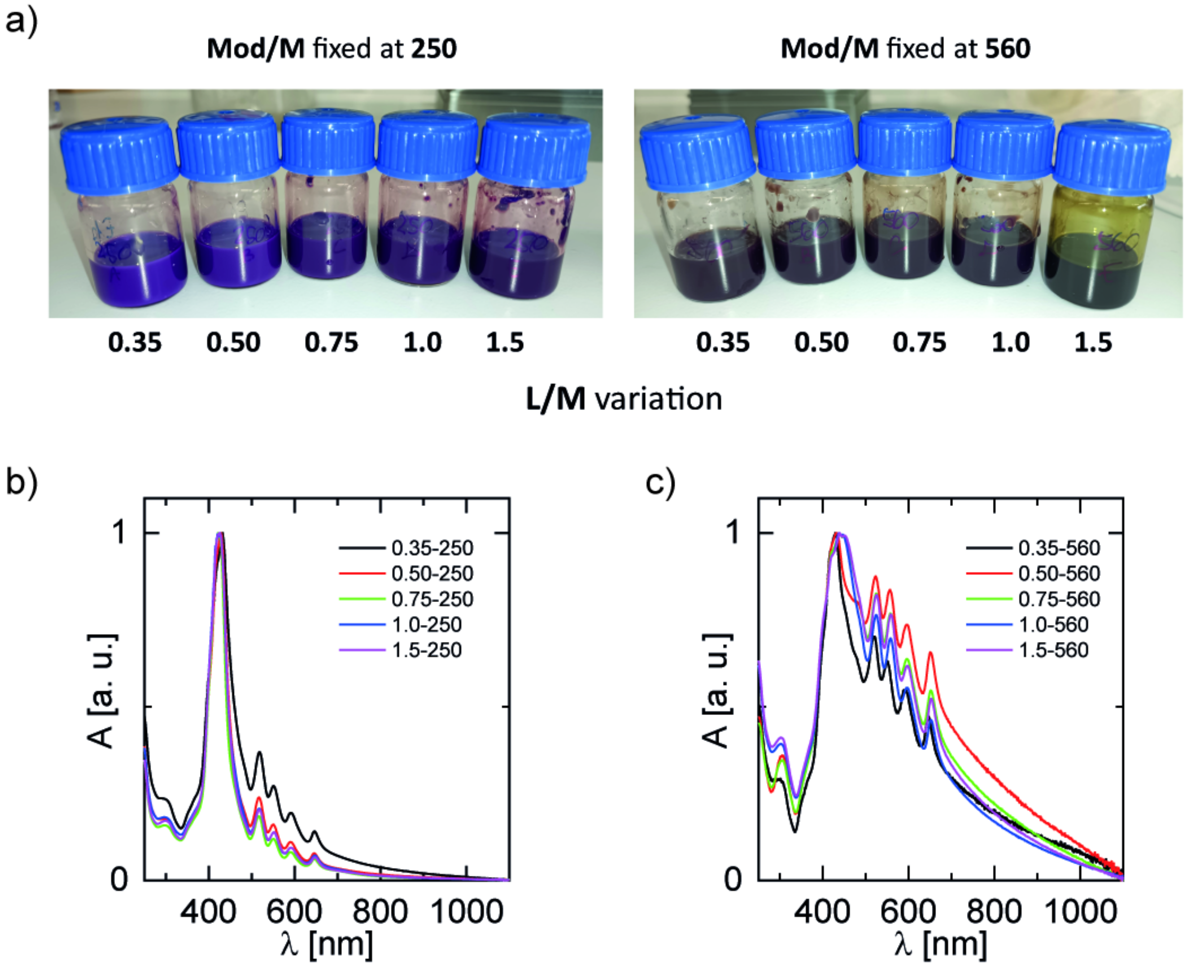

Figure S26. a) Digital images of 1h reaction conducted at 75 °C with FA and $Zr(OEt)_4$, by varying the L/M and Mod/M ratios. UV-Vis extinction spectra of b) Mod/M = 250 and c) Mod/M = 560.

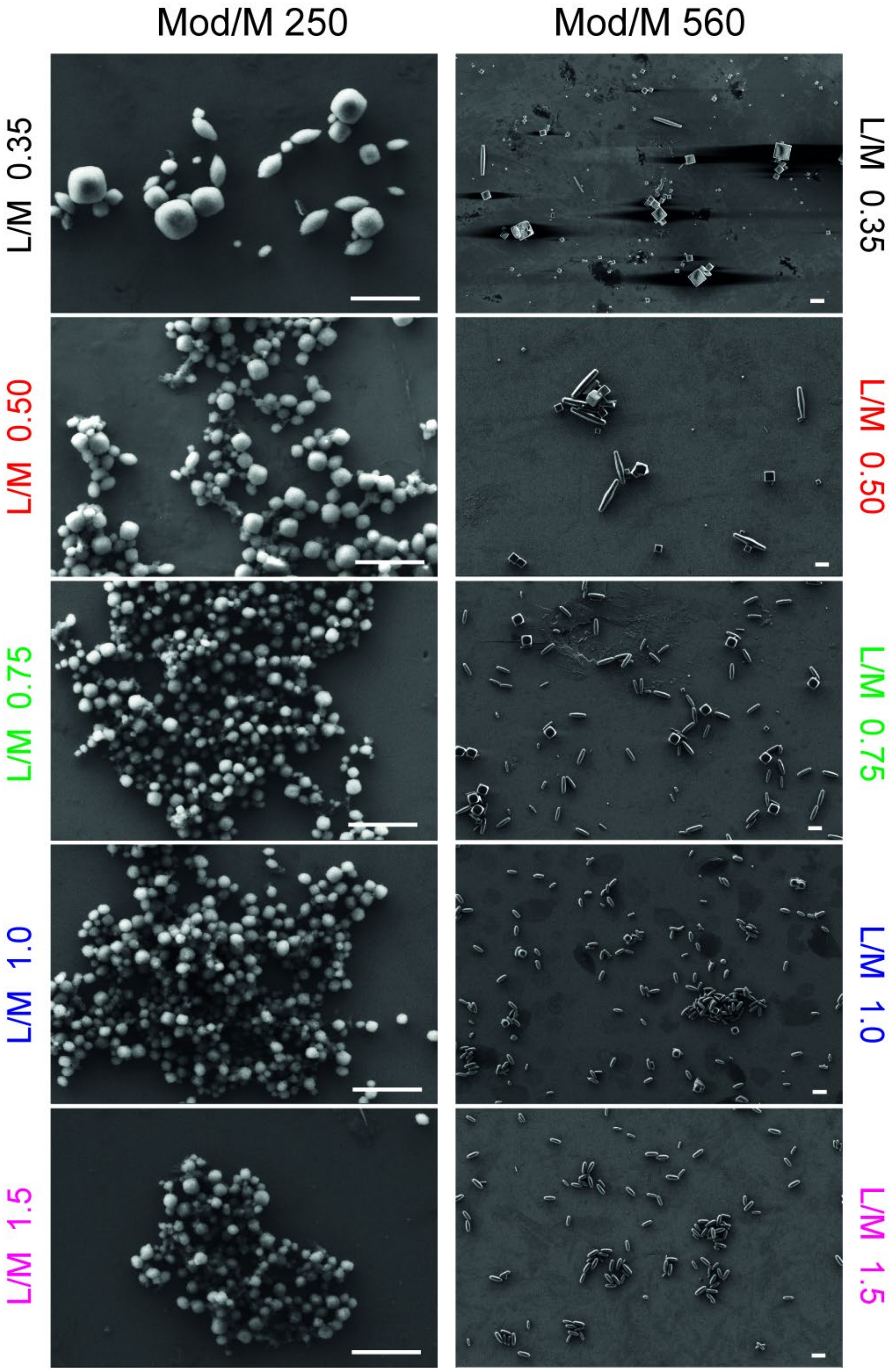

Figure S27. Representative FE-SEM images of the L/M and Mod/M modulations for Zr-porphyrinic MOF nanoparticles synthesis performed at 75 °C, 1h-reaction, with the use of FA as modulator and $Zr(OEt)_4$ as precursor. Mod/M 250 scale bars: 1 μm. Mod/M 560 scale bars: 2 μm.

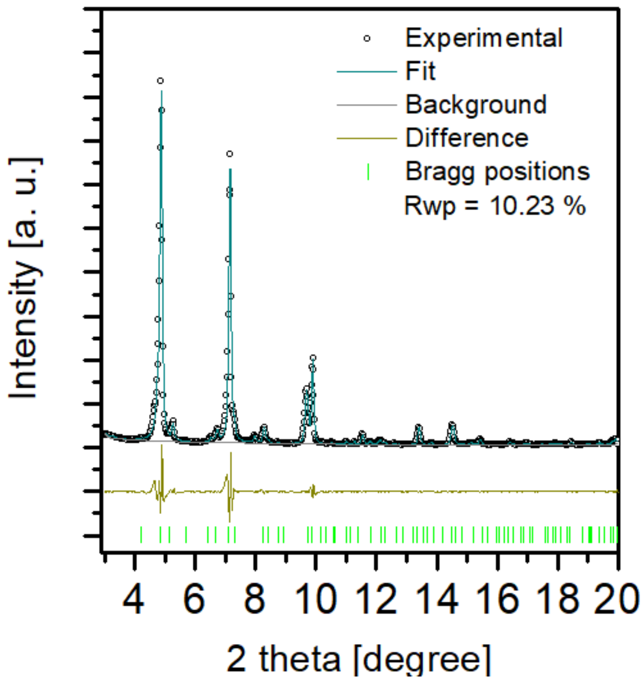

Figure S28. Pawley refinement of Zr-porphyrinic MOF nanoparticles, synthesized at 75 °C, 1 h reaction, by using $Zr(OEt)_4$ as precursor and FA as modulator. The ratios adopted were L/M = 1.5 and Mod/M = 560.

Reproducibility test

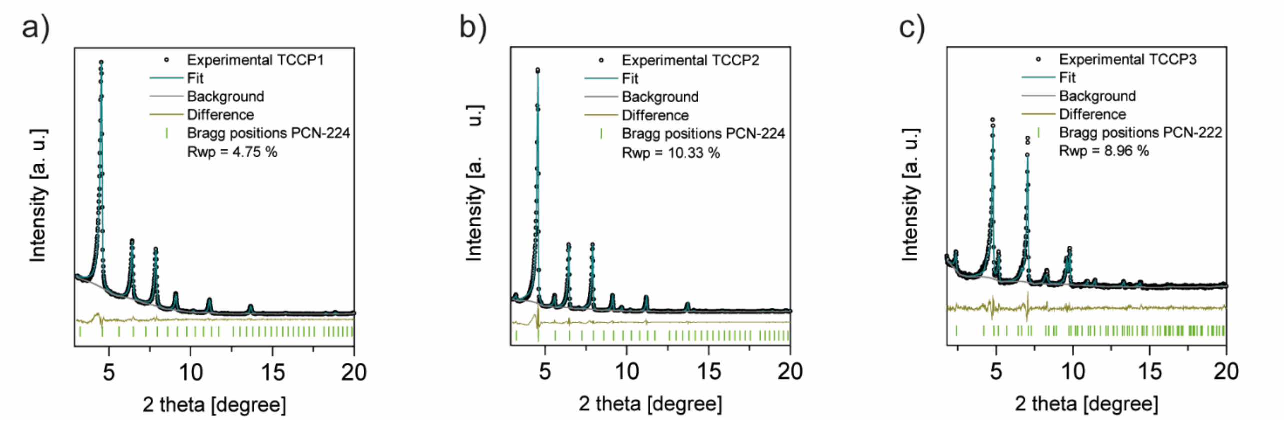

Figure S29. Reproducibility test for the synthesis of PCN-224 following synthetic conditions of a) Table S1 and b) Table S2 and for the synthesis of PCN-222 following the synthetic conditions of Table S3, using $Zr(OEt)_4$ as precursor.

Continuous flow reaction

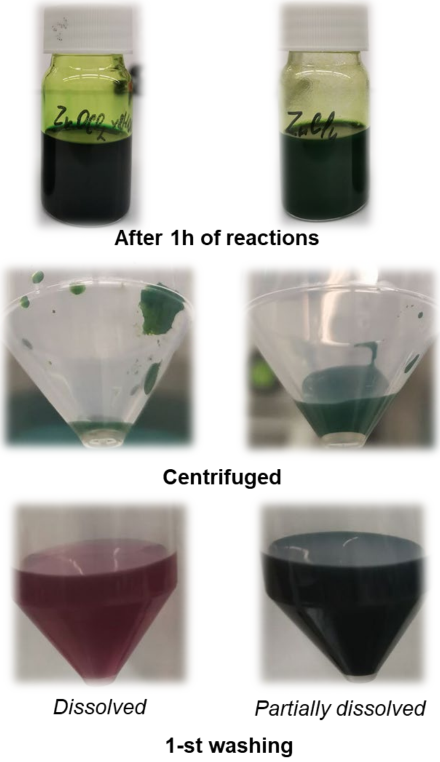

Figure S30. Photo of the reactions using $ZrCl_4$ and $ZrOCl_2$ as metal precursors at room temperature, 1h.

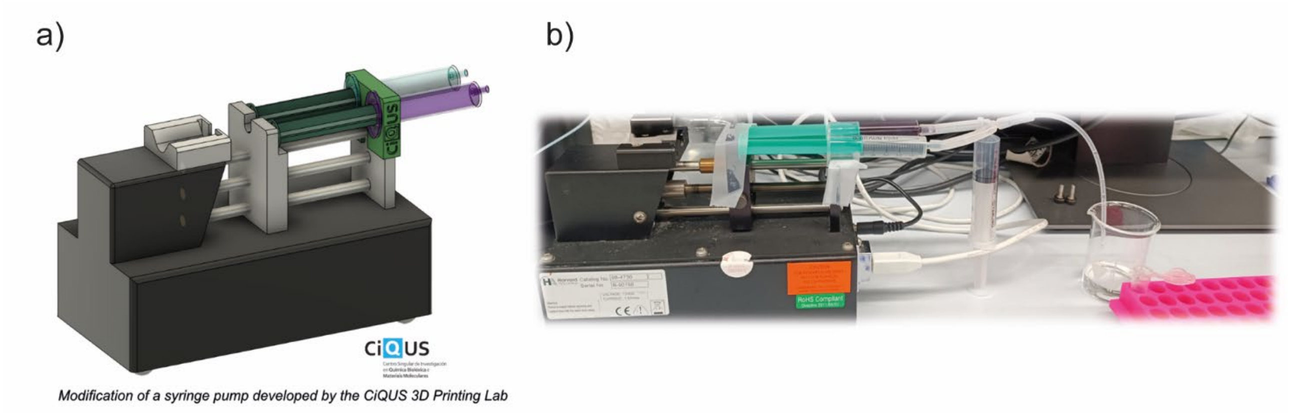

Figure S31. a) Pump infusion prototype and b) photo of continuous flow setup using an infusion pump with two syringes.

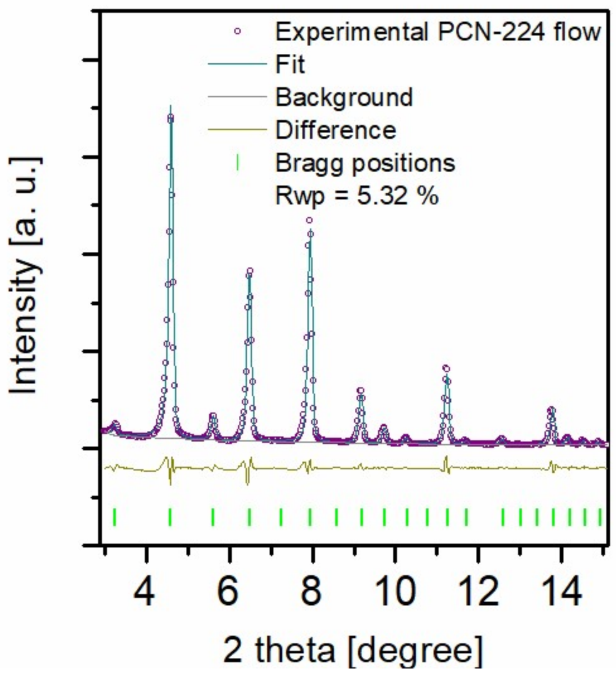

Figure S32. Pawley refinement of PCN-224 synthesized by continuous flow reaction.

APPENDIX BETSI N_2 adsorption analysis

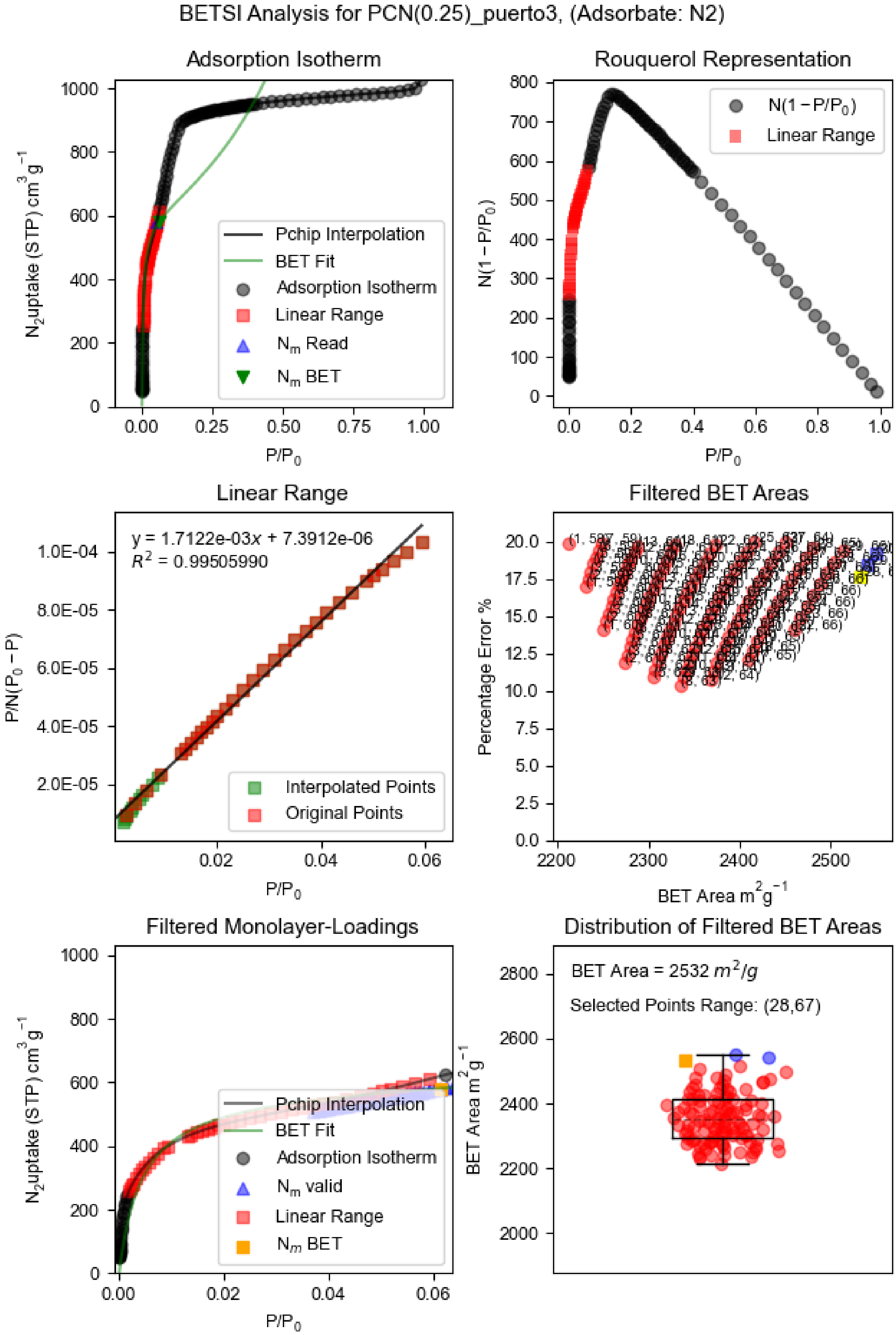

Figure S33. BETSI analysis of Zr-porphyrinic MOF synthesized with L/M 0.25, Mod(AA)/M 560, RT, 1 h of reaction and $Zr(OEt)_4$ as precursor.

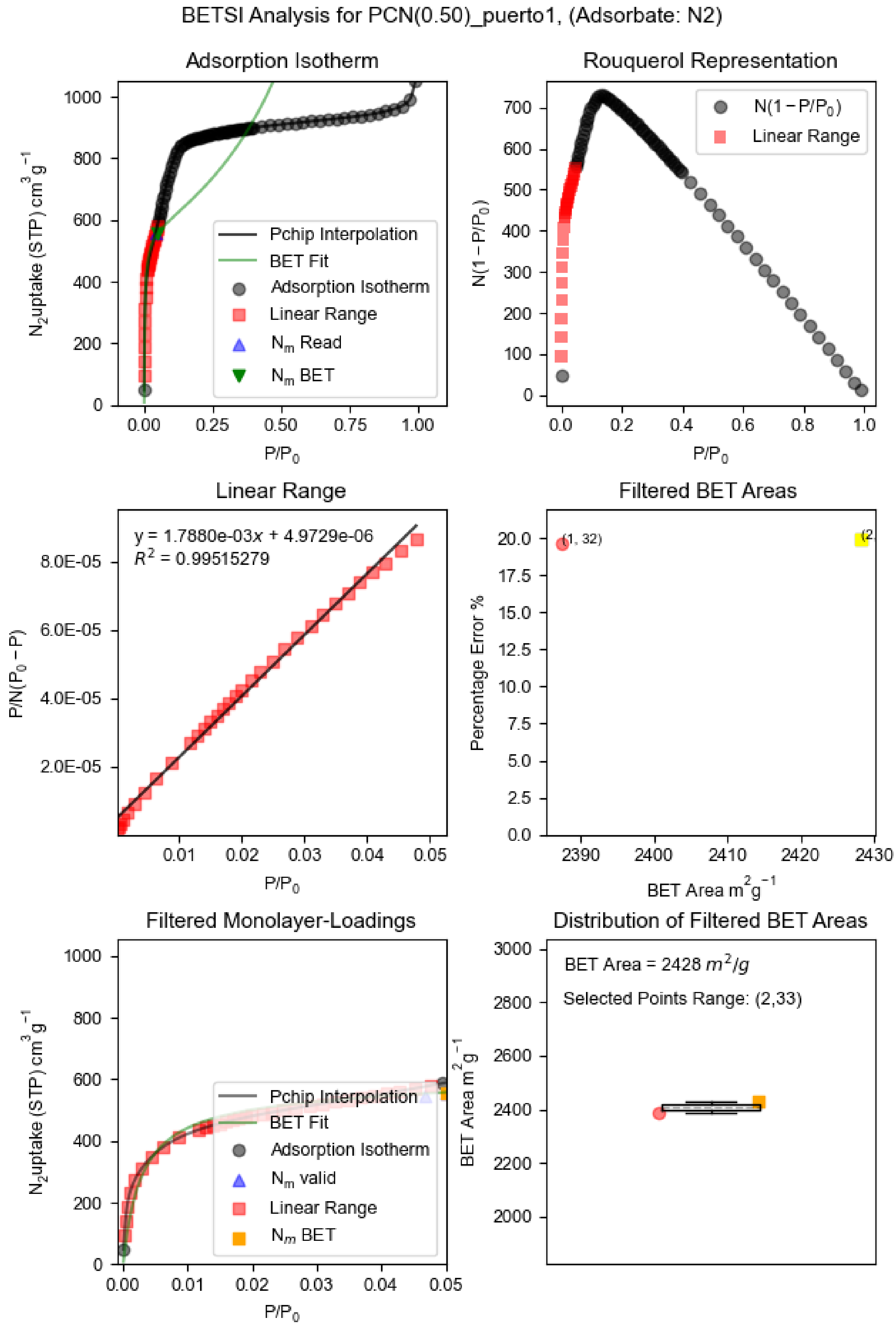

Figure S34. BETSI analysis of Zr-porphyrinic MOF synthesized with L/M 0.50, Mod(AA)/M 560, RT, 1h of reaction and $Zr(OEt)_4$ as precursor.

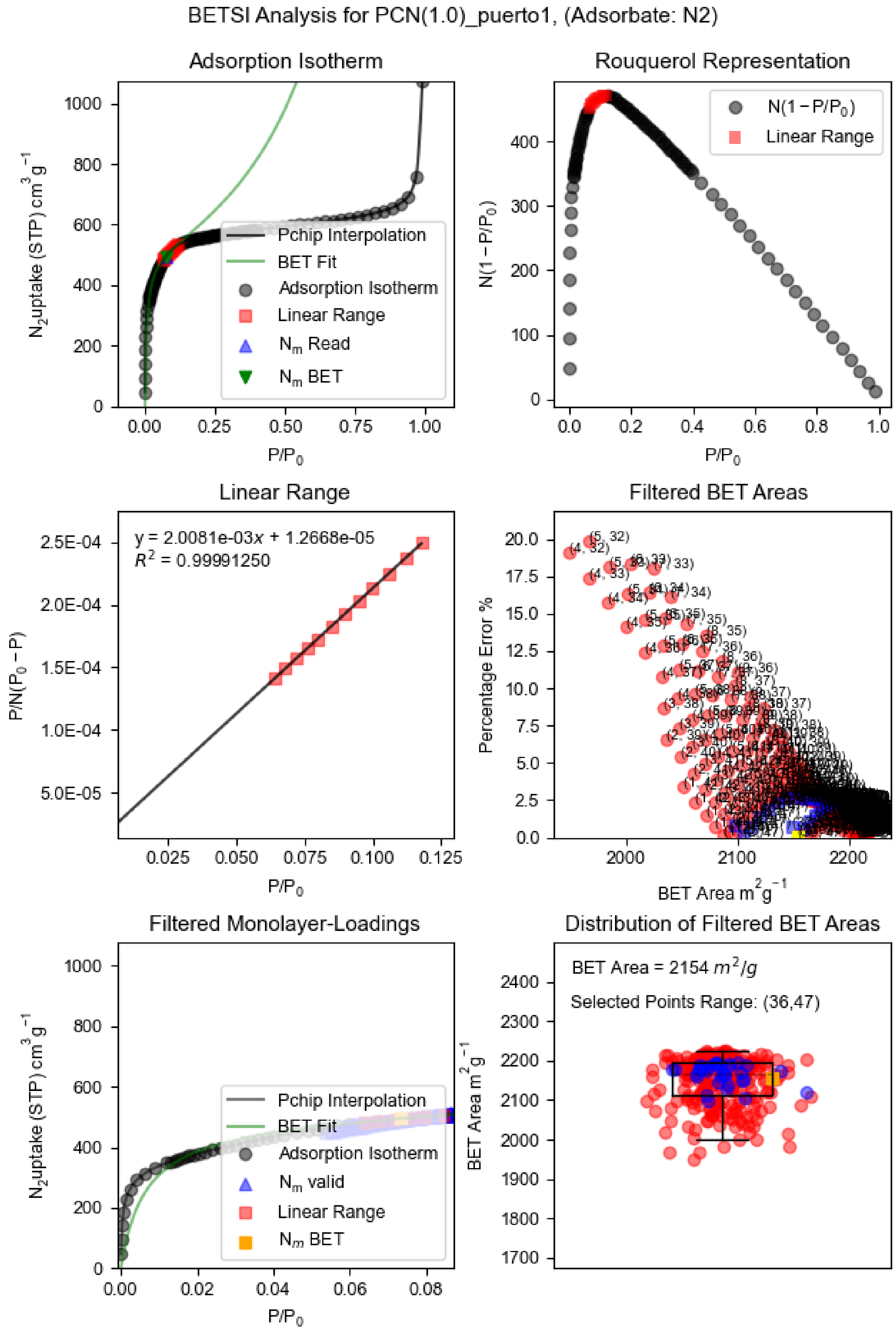

Figure S35. BETSI analysis of Zr-porphyrinic MOF synthesized with L/M 1.00, Mod(AA)/M 560, RT, 1h of reaction and $Zr(OEt)_4$ as precursor.

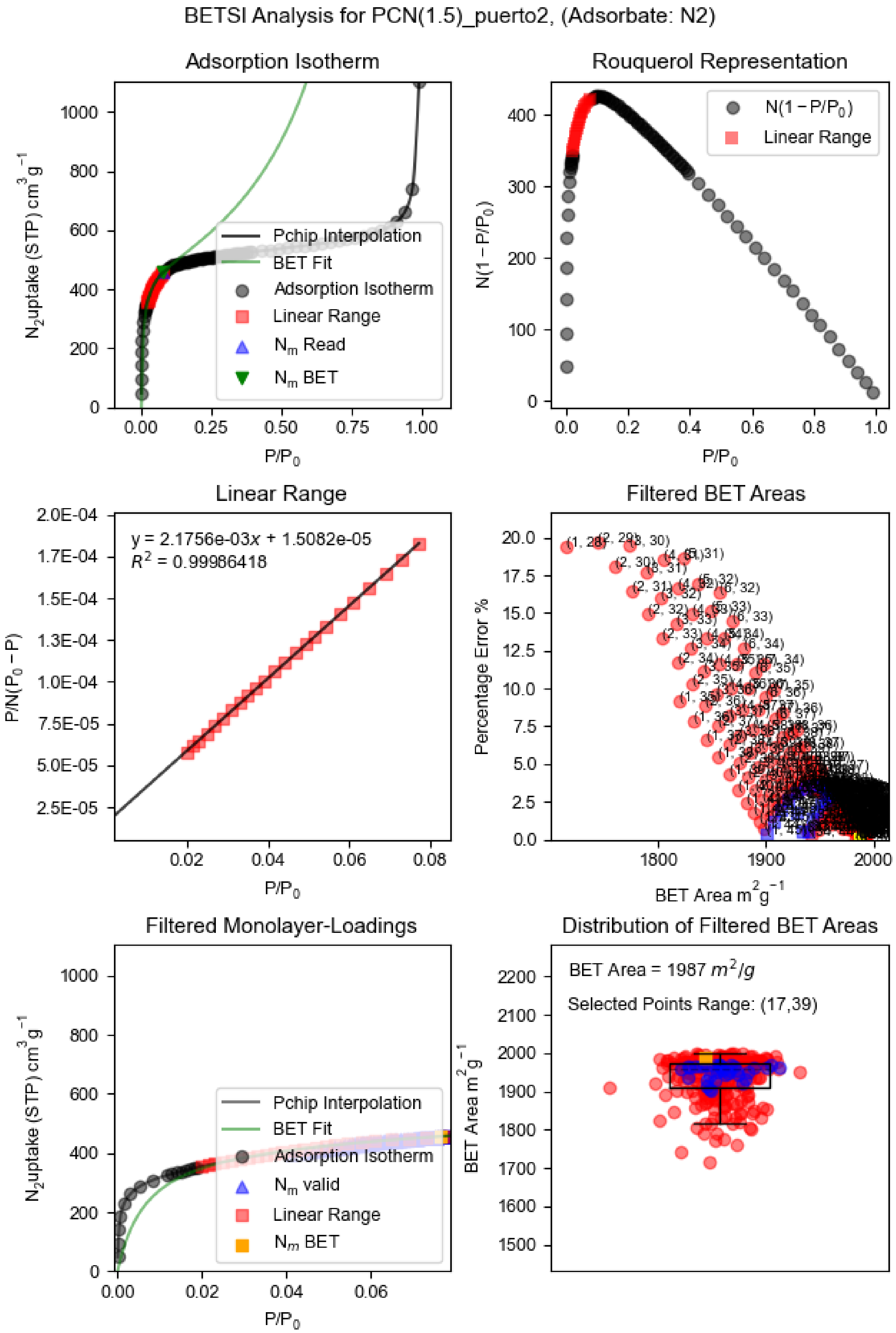

Figure S36. BETSI analysis of Zr-porphyrinic MOF synthesized with L/M 1.50, Mod(AA)/M 560, RT, 1h of reaction and $Zr(OEt)_4$ as precursor.

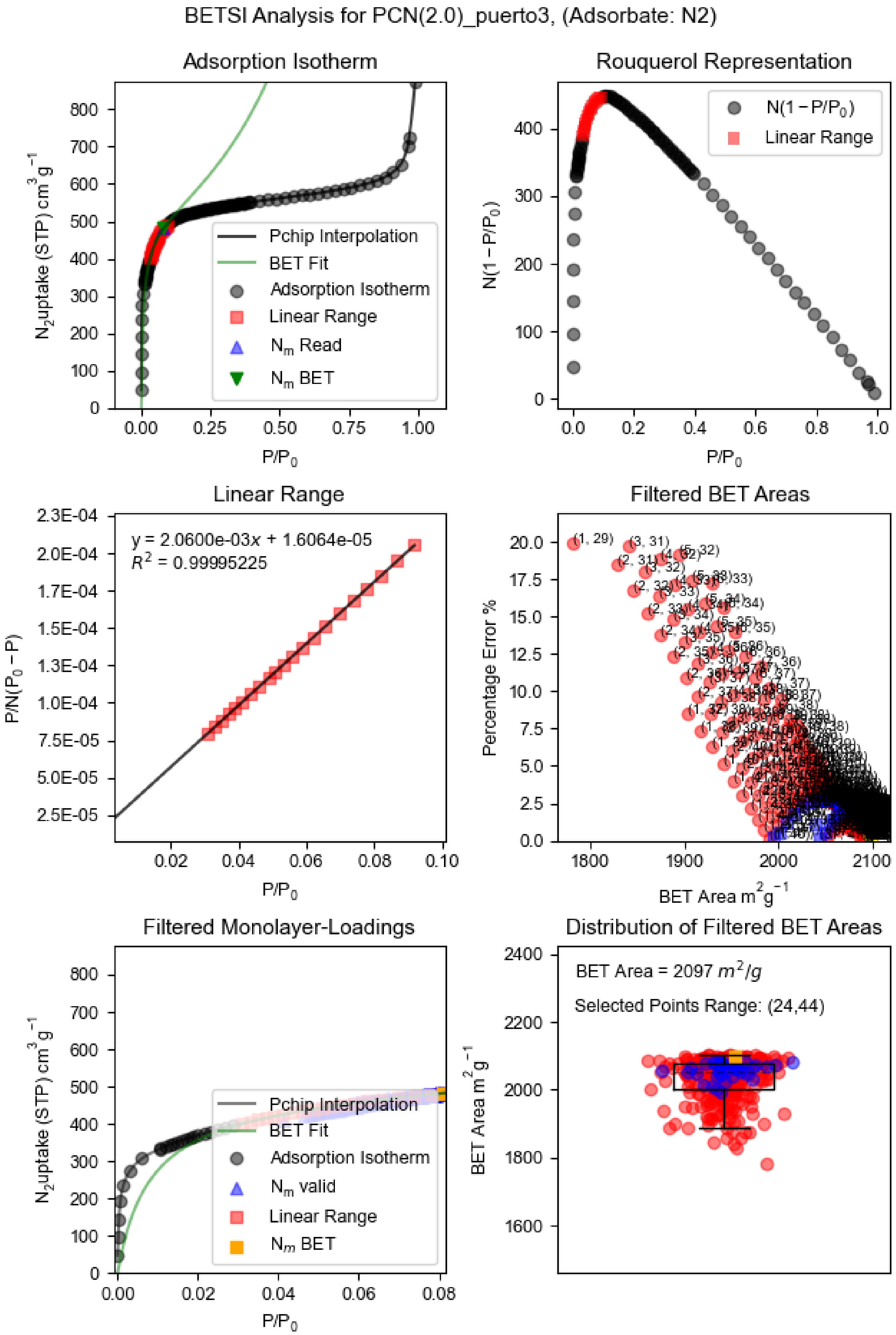

Figure S37. BETSI analysis of Zr-porphyrinic MOF synthesized with L/M 2.00, Mod(AA)/M 560, RT, 1h of reaction and Zr(OEt)4 as precursor.

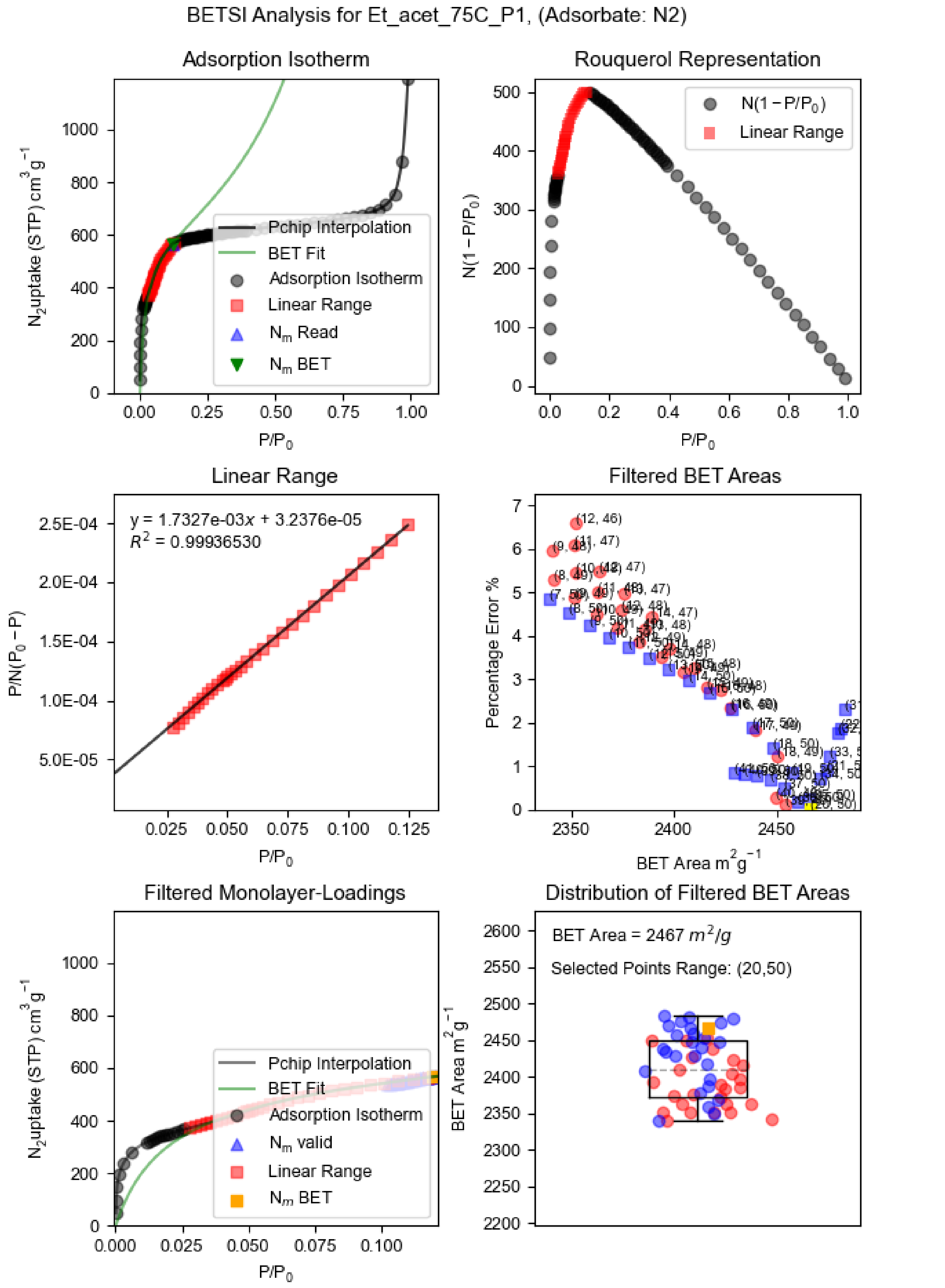

Figure S38. BETSI analysis of Zr-porphyrinic MOF synthesized with L/M 0.25, Mod(AA)/M 250, 75 ºC, 1h of reaction and $Zr(OEt)_4$ as precursor.

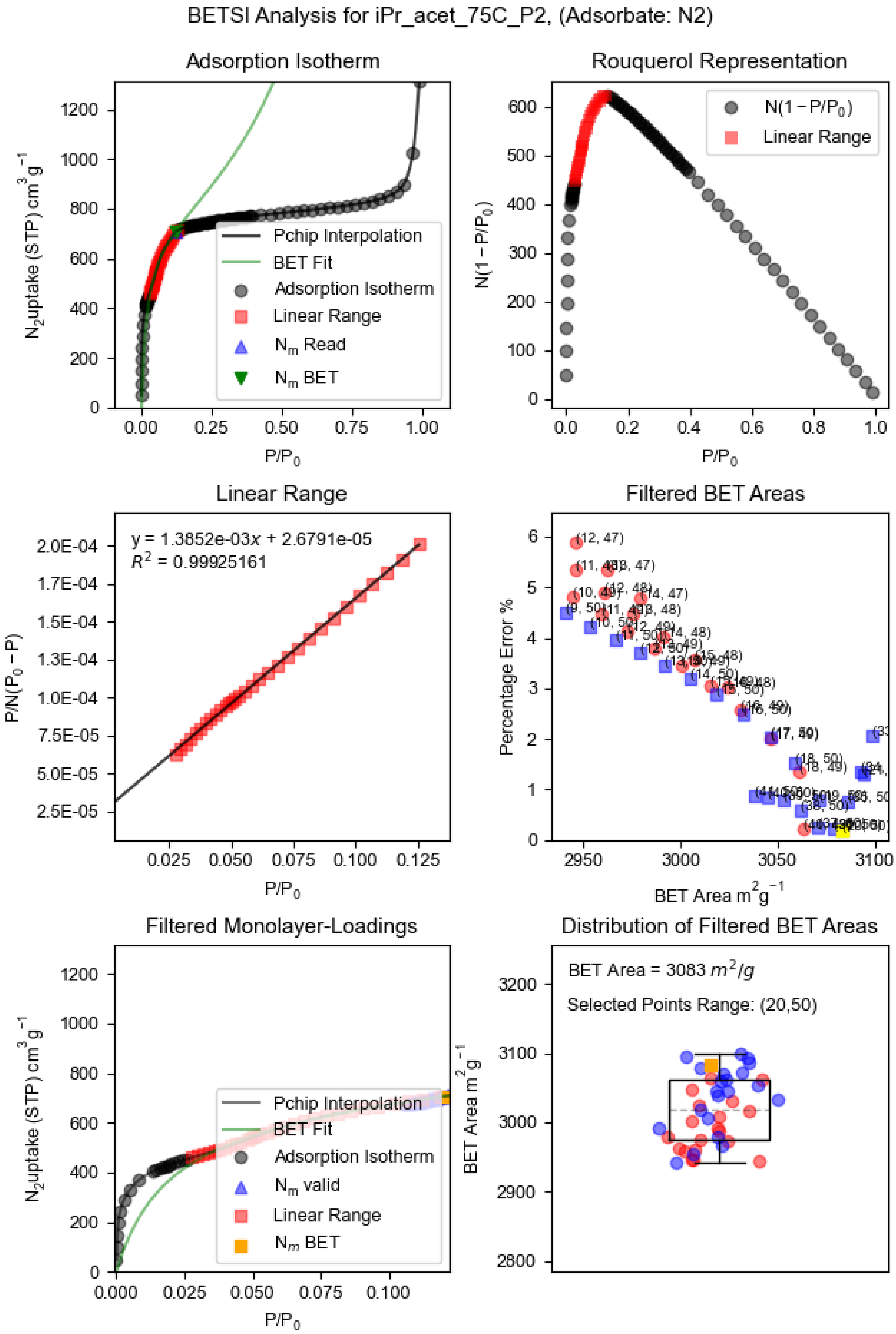

Figure S39. BETSI analysis of Zr-porphyrinic MOF synthesized with L/M 0.25, Mod(AA)/M 250, 75 ºC, 1h of reaction and Zr(OiPr)4 as precursor.

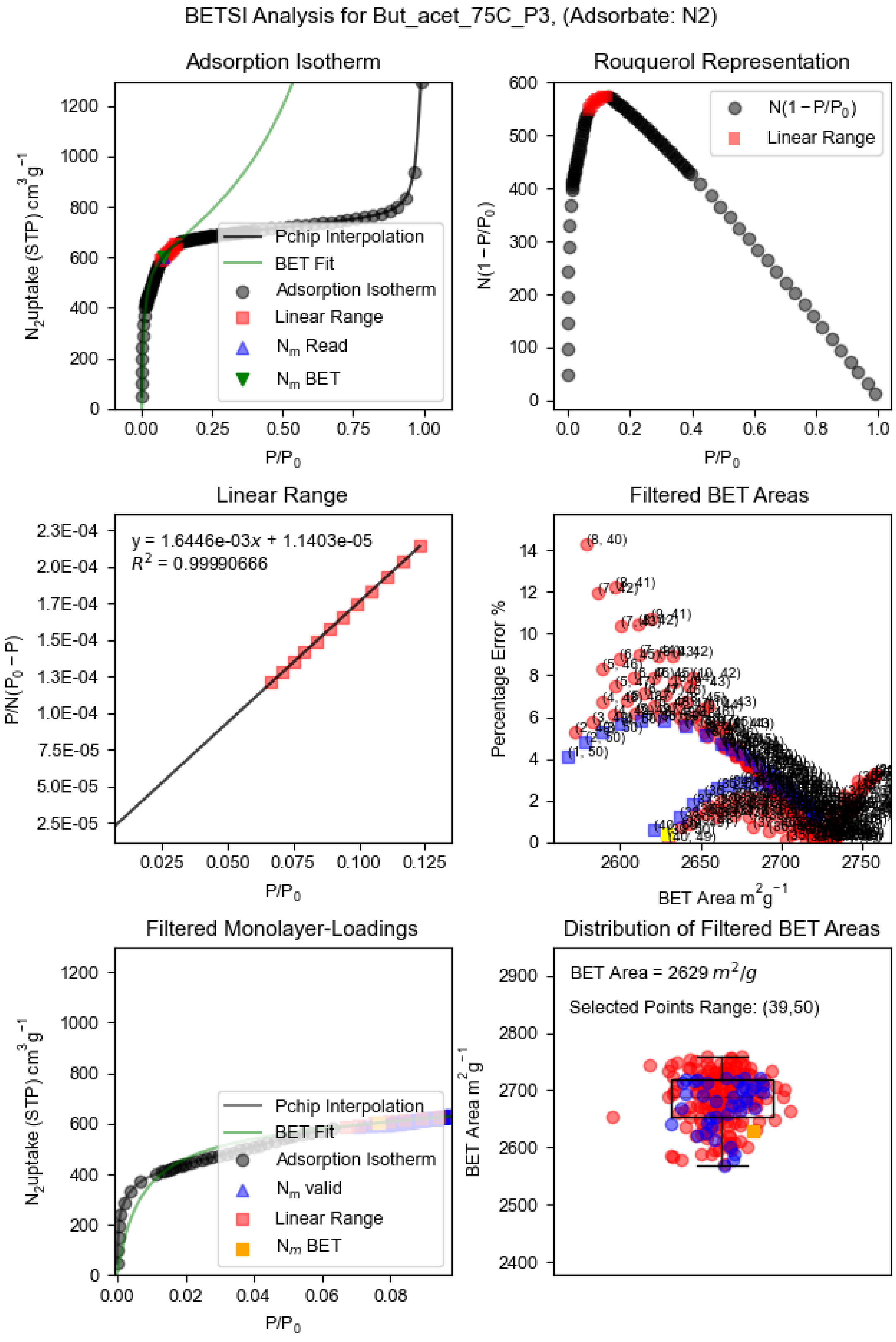

Figure S40. BETSI analysis of Zr-porphyrinic MOF synthesized with L/M 0.25, Mod(AA)/M 250, 75 ºC, 1h of reaction and Zr(OiBu)₄ as precursor.

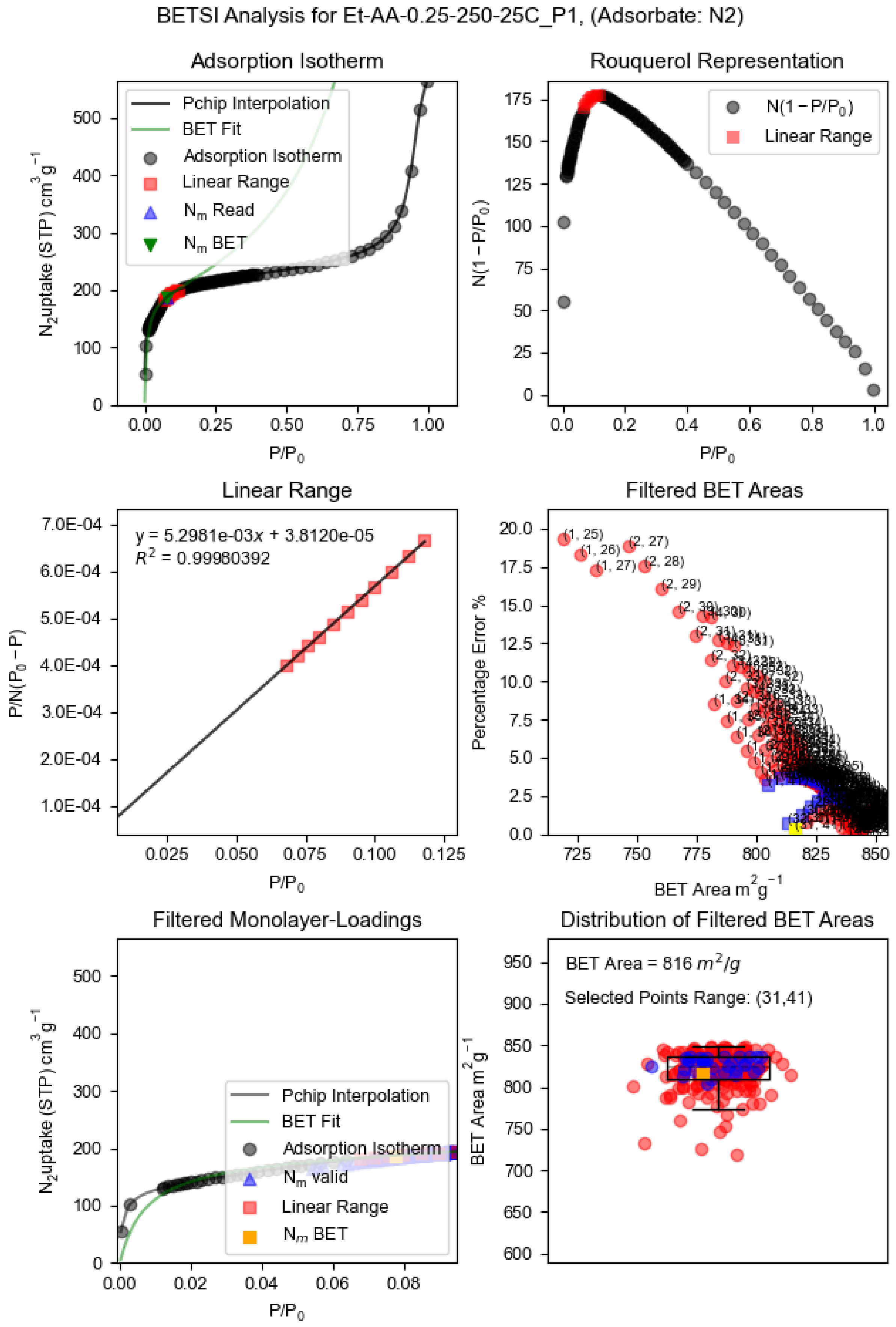

Figure S41. BETSI analysis of Zr-porphyrinic MOF synthesized with L/M 0.25, Mod(AA)/M 250, RT, 1h of reaction and $Zr(OEt)_4$ as precursor.

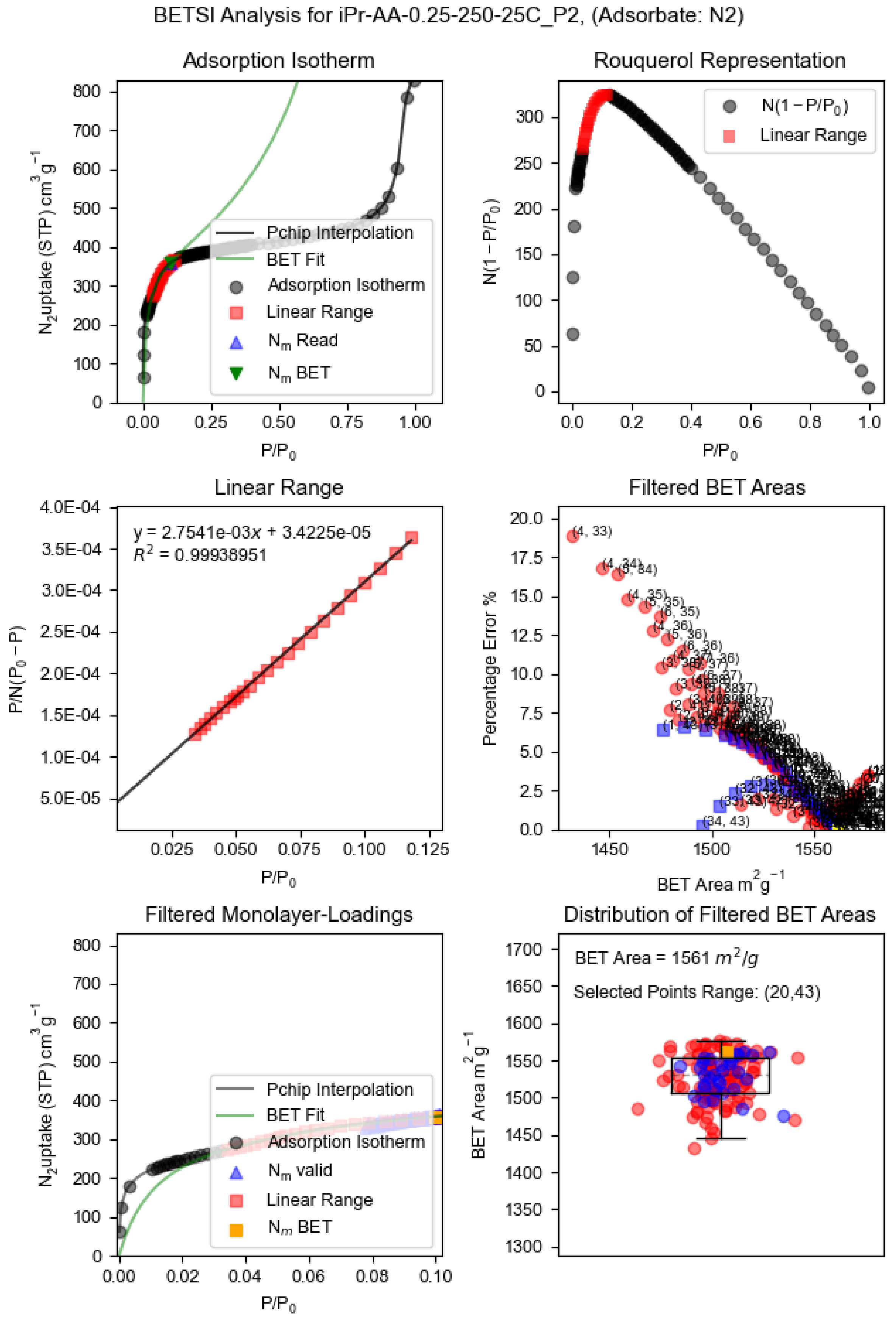

Figure S42. BETSI analysis of Zr-porphyrinic MOF synthesized with L/M 0.25, Mod(AA)/M 250, RT, 1h of reaction and $Zr(OiPr)_4$ as precursor.

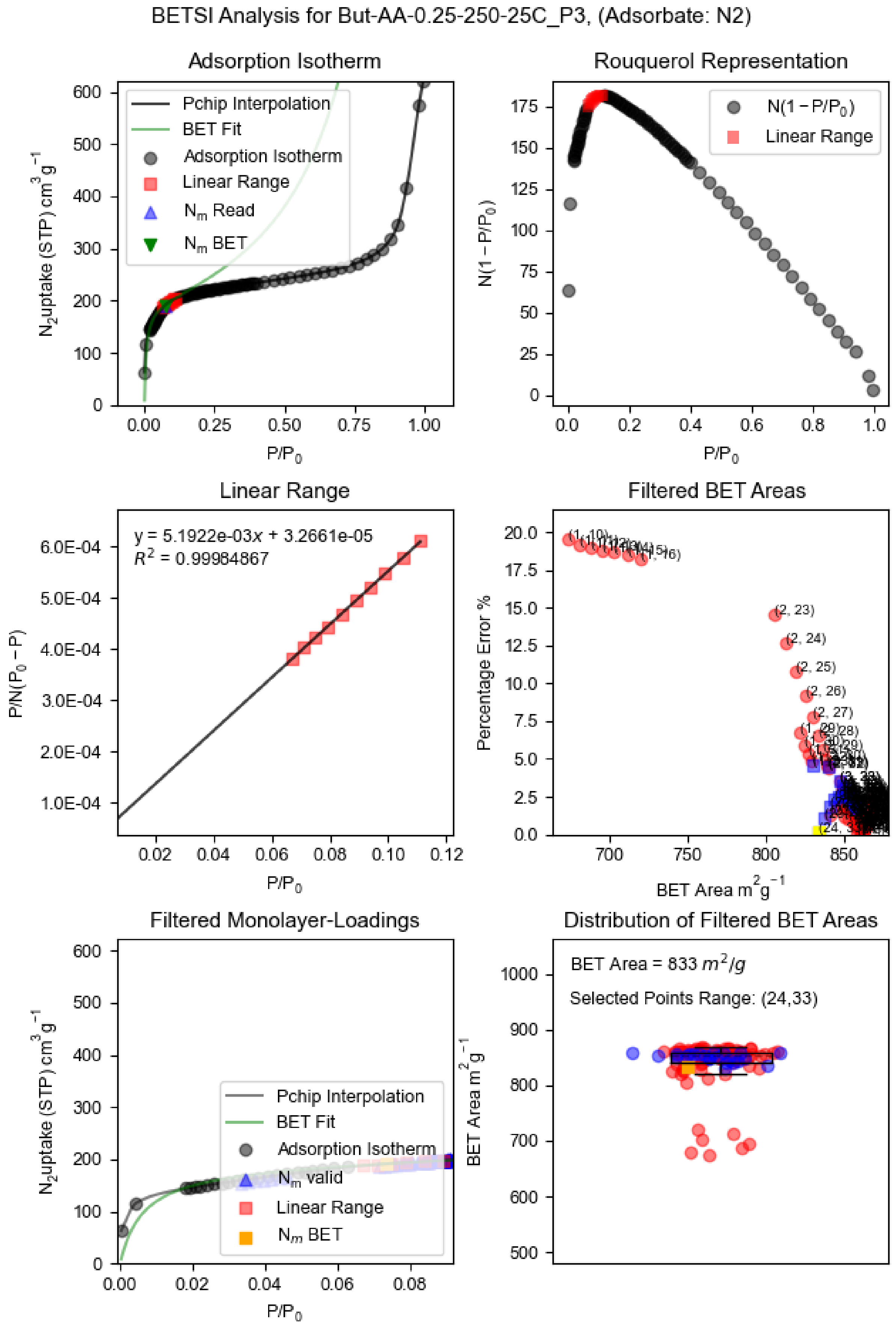

Figure S43. BETSI analysis of Zr-porphyrinic MOF synthesized with L/M 0.25, Mod(AA)/M 250, RT, 1h of reaction and Zr(OiBu)$_4$ as precursor.

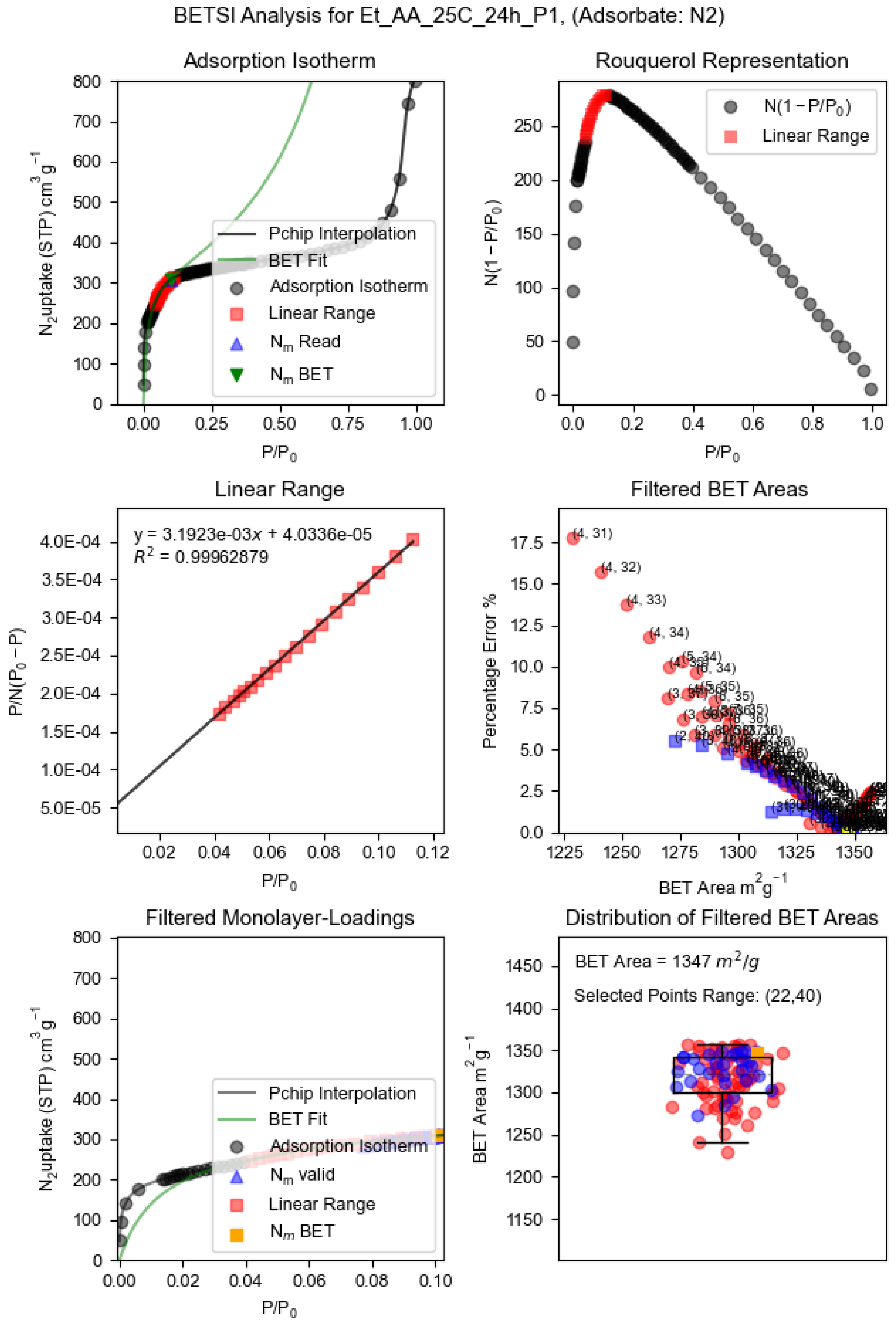

Figure S44. BETSI analysis of Zr-porphyrinic MOF synthesized with L/M 0.25, Mod(AA)/M 250, RT, 24h of reaction and $Zr(OEt)_4$ as precursor.

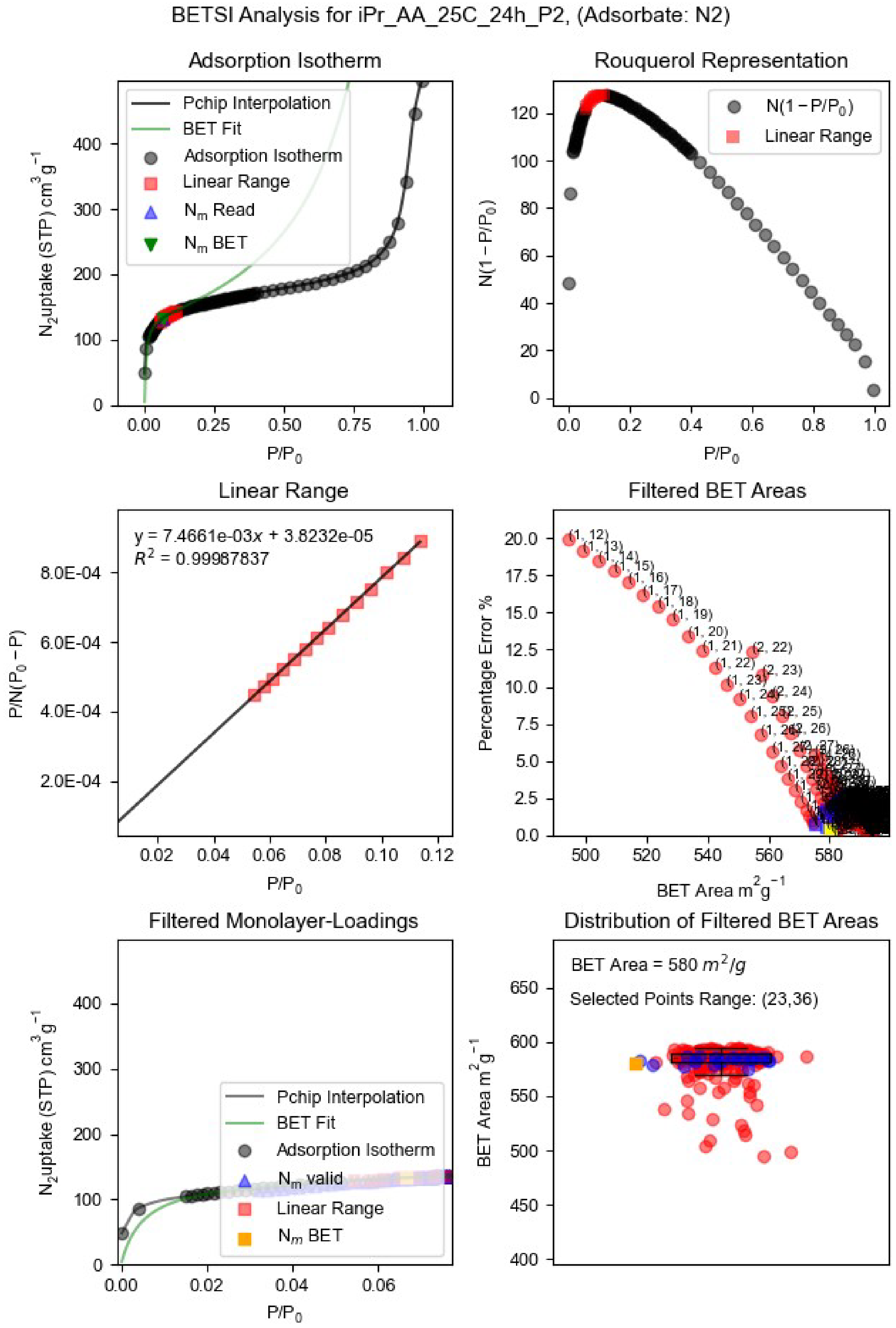

Figure S45. BETSI analysis of Zr-porphyrinic MOF synthesized with L/M 0.25, Mod(AA)/M 250, RT, 24h of reaction and $Zr(OiPr)_4$ as precursor.

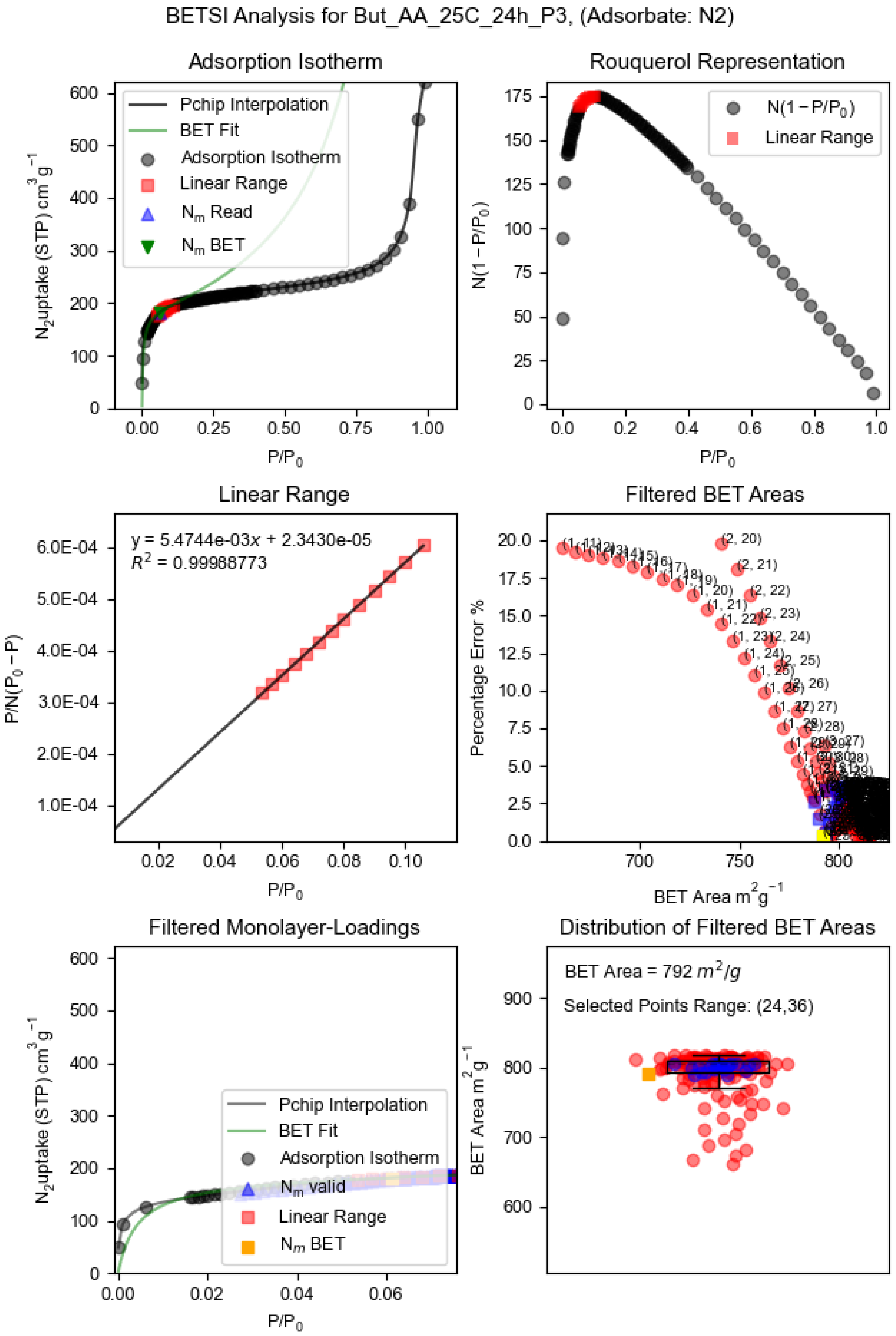

Figure S46. BETSI analysis of Zr-porphyrinic MOF synthesized with L/M 0.25, Mod(AA)/M 250, RT, 24h of reaction and $Zr(OBu)_4$ as precursor.

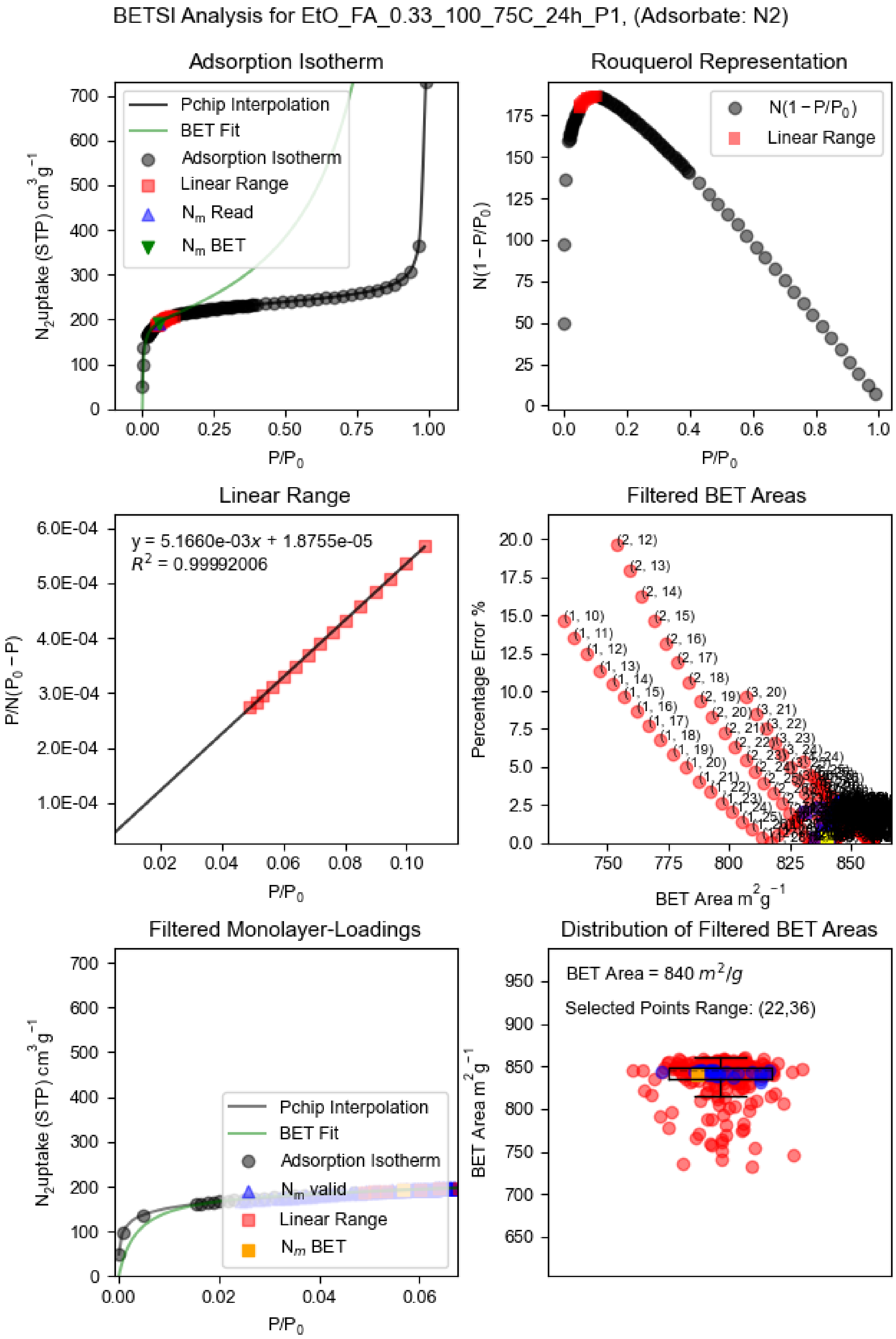

Figure S47. BETSI analysis of Zr-porphyrinic MOF synthesized with L/M 0.33, Mod(FA)/M 100, 75 ºC, 24h of reaction and $Zr(OEt)_4$ as precursor.

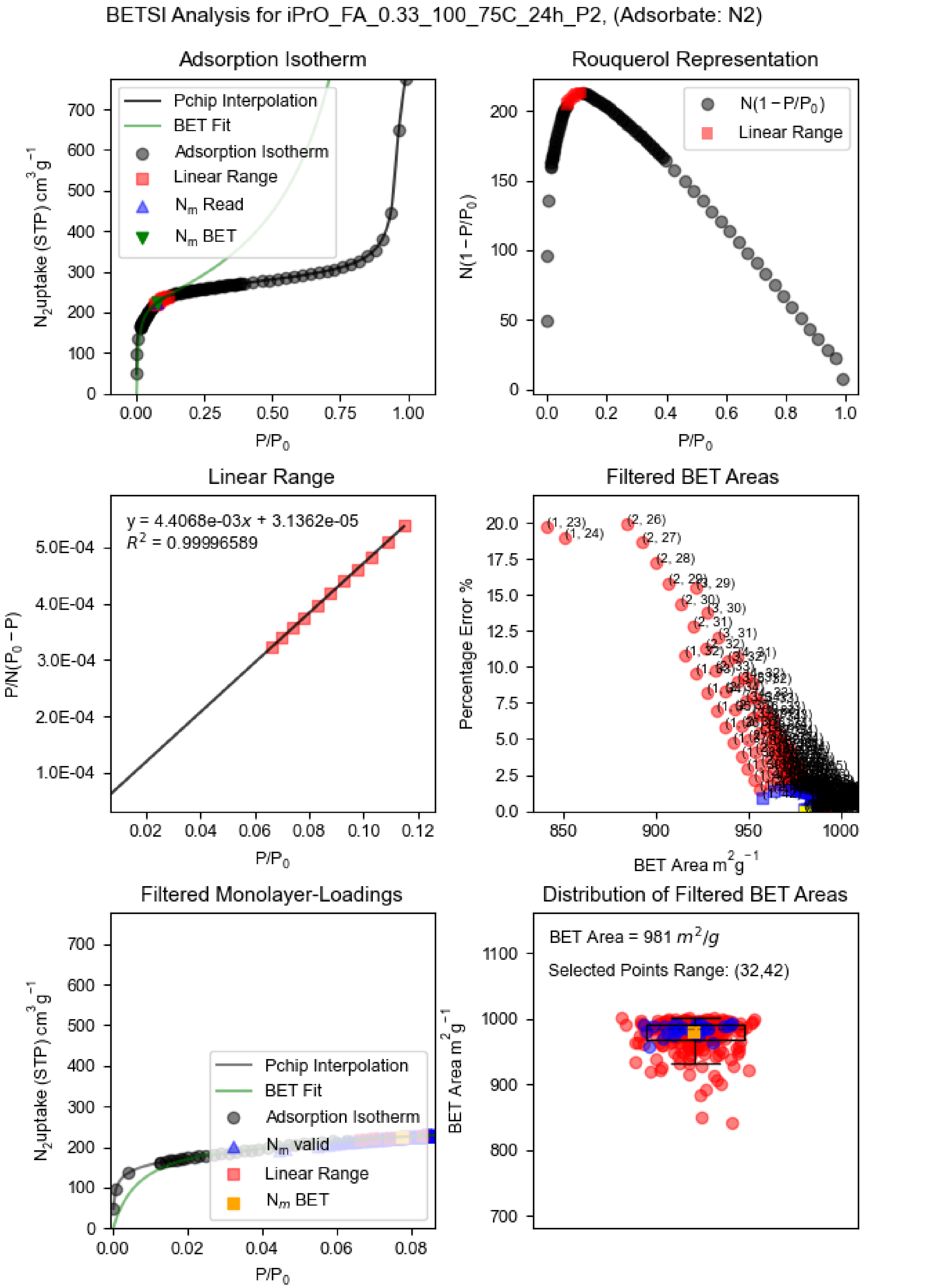

Figure S48. BETSI analysis of Zr-porphyrinic MOF synthesized with L/M 0.33, Mod(FA)/M 100, 75 ºC, 24h of reaction and $Zr(OiPr)_4$ as precursor.

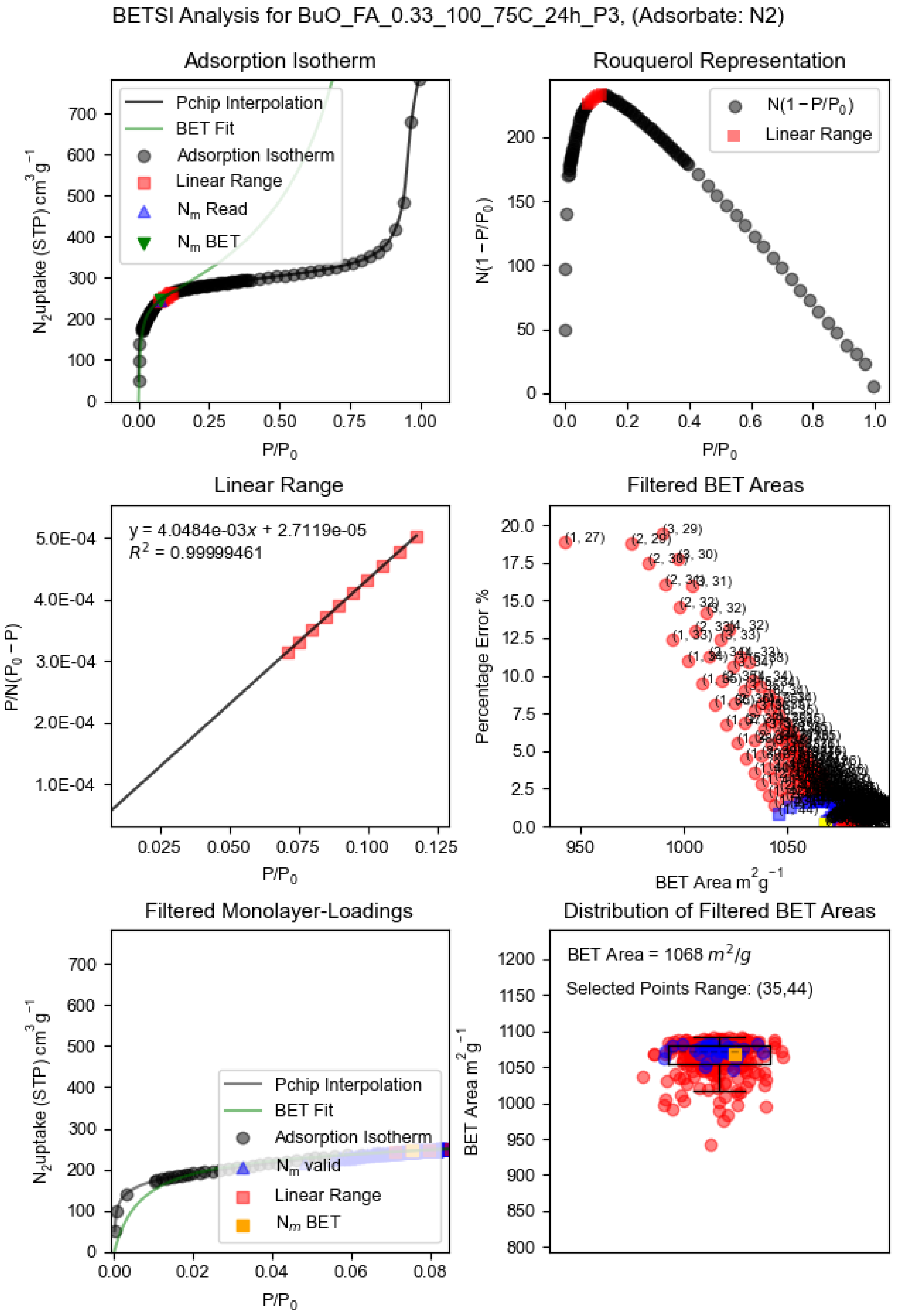

Figure S49. BETSI analysis of Zr-porphyrinic MOF synthesized with L/M 0.33, Mod(FA)/M 100, 75 ºC, 24h of reaction and $Zr(OBu)_4$ as precursor.

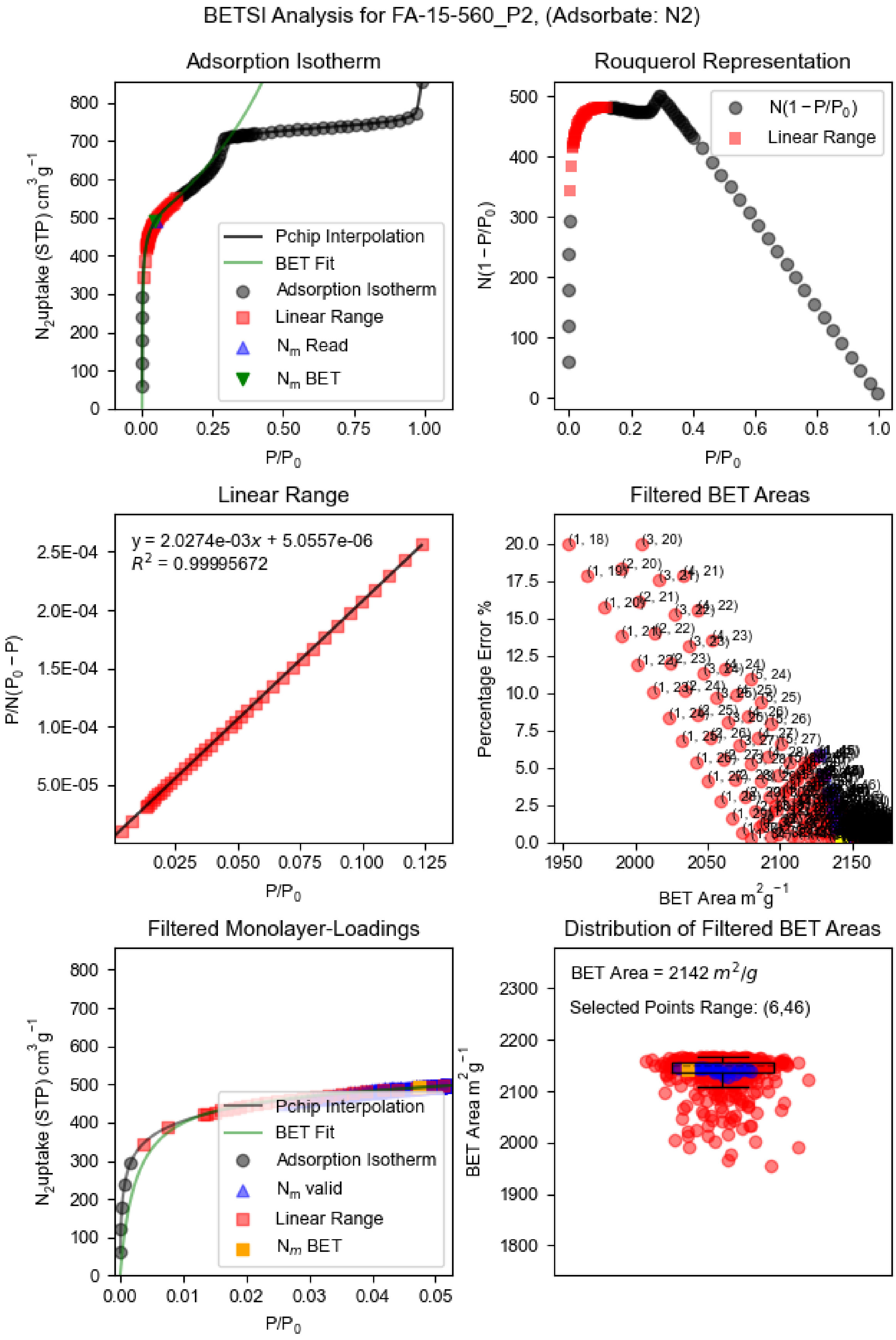

Figure S50. BETSI analysis of Zr-porphyrinic MOF synthesized with L/M 1.50, Mod(FA)/M 560, 75 ºC, 1h of reaction and $Zr(OEt)_4$ as precursor.

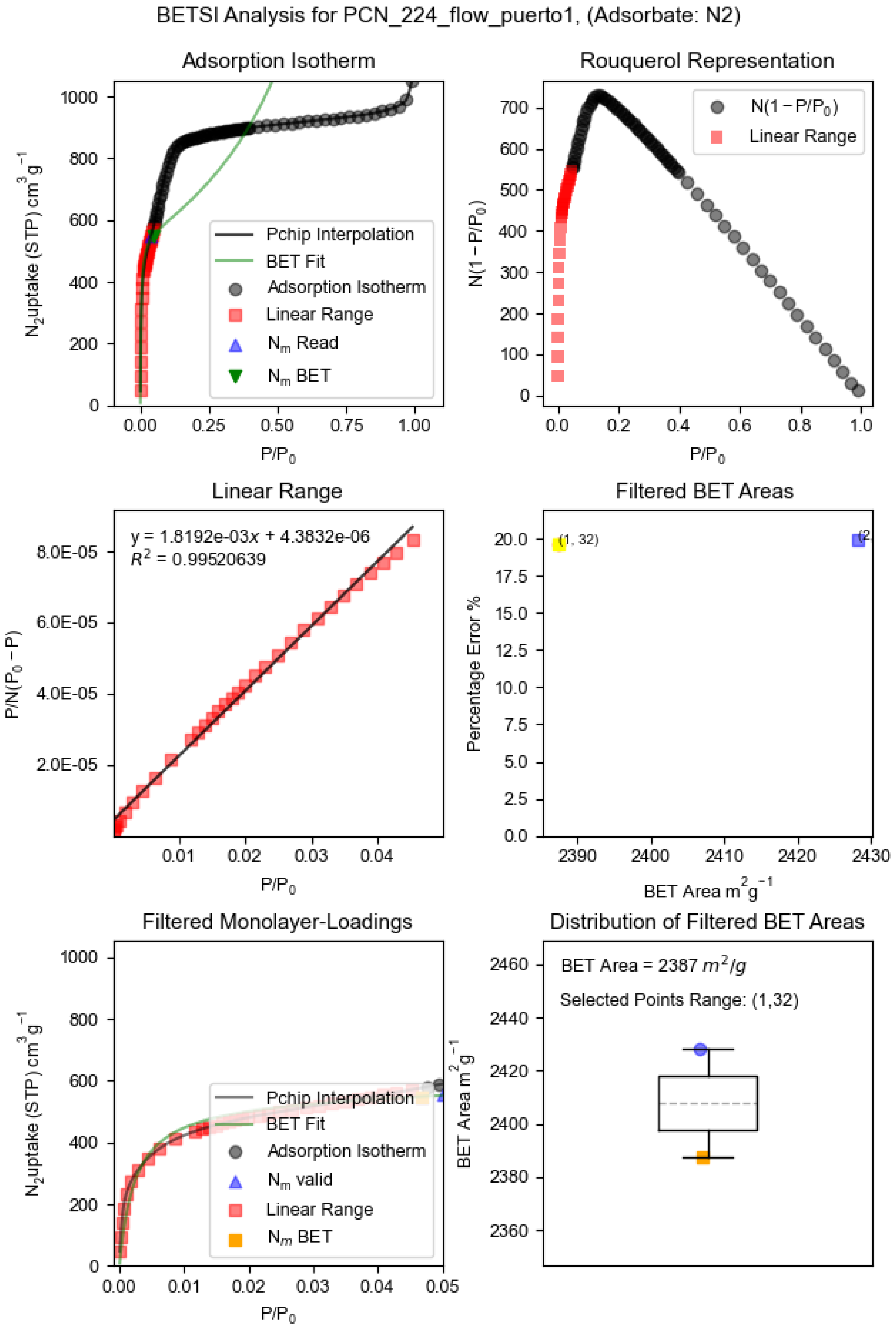

Figure S51. BETSI analysis of Zr-porphyrinic MOF synthesized in flow with L/M 0.25, Mod(AA)/M 560, RT, 25 s of reaction and $Zr(OEt)_4$ as precursor.

References

1. Osterrieth, J.W.M., Rampersad, J., Madden, D., Rampal, N., Skoric, L., Connolly, B., Allendorf, M.D., Stavila, V., Snider, J.L., Ameloot, R., et al. (2022). How Reproducible are Surface Areas Calculated from the BET Equation? Adv. Mater. *34*, 2201502.
2. Chu, J., Ke, F.S., Wang, Y., Feng, X., Chen, W., Ai, X., Yang, H., and Cao, Y. (2020). Facile and reversible digestion and regeneration of zirconium-based metal-organic frameworks. Commun. Chem. *3*, 5.
3. Li, Y., Lo, W.S., Zhang, F., Si, X., Chou, L.Y., Liu, X.Y., Williams, B.P., Li, Y.H., Jung, S.H., Hsu, Y.S., et al. (2021). Creating an Aligned Interface between Nanoparticles and MOFs by Concurrent Replacement of Capping Agents. J. Am. Chem. Soc. *143*, 5182–5190.
4. G, E., K, V., and Nudelman, A. (1997). NRM Chemicals Shifts of common laboratory solvents as traces imputities. J. org. Chem *3263*, 7512–7515.
5. Willems, T.F., Rycroft, C.H., Kazi, M., Meza, J.C., and Haranczyk, M. (2012). Algorithms and tools for high-throughput geometry-based analysis of crystalline porous materials. Microporous Mesoporous Mater. *149*, 134–141.
